# American mastodon mitochondrial genomes suggest multiple dispersal events in response to Pleistocene climate oscillations

**DOI:** 10.1038/s41467-020-17893-z

**Published:** 2020-09-01

**Authors:** Emil Karpinski, Dirk Hackenberger, Grant Zazula, Chris Widga, Ana T. Duggan, G. Brian Golding, Melanie Kuch, Jennifer Klunk, Christopher N. Jass, Pam Groves, Patrick Druckenmiller, Blaine W. Schubert, Joaquin Arroyo-Cabrales, William F. Simpson, John W. Hoganson, Daniel C. Fisher, Simon Y. W. Ho, Ross D. E. MacPhee, Hendrik N. Poinar

**Affiliations:** 1 McMaster Ancient DNA Centre, Departments of Anthropology and Biochemistry, McMaster University, Hamilton, ON L8S 4L9 Canada; 2 Department of Biology, McMaster University, Hamilton, ON L8S 4L8 Canada; 3 Department of Biochemistry, McMaster University, Hamilton, ON L8S 4L8 Canada; 4 Yukon Palaeontology Program, Department of Tourism and Culture, Government of Yukon, Whitehorse, YT Y1A 2C6 Canada; 5 Research and Collections, Canadian Museum of Nature, Ottawa, ON K2P 2R1 Canada; 6 Center of Excellence in Paleontology and Department of Geosciences, East Tennessee State University, Johnson City, TN 37614 USA; 7 Department of Anthropology, McMaster University, Hamilton, ON L8S 4L9 Canada; 8 Arbor Biosciences, Ann Arbor, MI 48103 USA; 9 Quaternary Palaeontology Program, Royal Alberta Museum, Edmonton, T5J 0G2 Canada; 10 Institute of Arctic Biology, University of Alaska Fairbanks, Alaska, AK 99775 USA; 11 Department of Geosciences, University of Alaska Fairbanks, Alaska, AK 99775 USA; 12 University of Alaska Museum, University of Alaska Fairbanks, Alaska, AK 99775 USA; 13 Laboratorio de Arqueozoologia, SLAA, Instituto Nacional de Antropología e Historia, Ciudad de México, 06600 México; 14 Gantz Family Collections Center, Field Museum of Natural History, Chicago, IL 60605 USA; 15 North Dakota Geological Survey, Bismarck, ND 58505 USA; 16 Museum of Paleontology and Department of Earth and Environmental Sciences, University of Michigan, Ann Arbor, MI 48109 USA; 17 School of Life and Environmental Sciences, University of Sydney, Sydney, NSW 2006 Australia; 18 Department of Mammalogy/Vertebrate Zoology, American Museum of Natural History, New York, NY 10024 USA

**Keywords:** Climate-change ecology, Palaeoecology, Palaeontology, Genetic variation

## Abstract

Pleistocene glacial-interglacial cycles are correlated with dramatic temperature oscillations. Examining how species responded to these natural fluctuations can provide valuable insights into the impacts of present-day anthropogenic climate change. Here we present a phylogeographic study of the extinct American mastodon (*Mammut americanum*), based on 35 complete mitochondrial genomes. These data reveal the presence of multiple lineages within this species, including two distinct clades from eastern Beringia. Our molecular date estimates suggest that these clades arose at different times, supporting a pattern of repeated northern expansion and local extirpation in response to glacial cycling. Consistent with this hypothesis, we also note lower levels of genetic diversity among northern mastodons than in endemic clades south of the continental ice sheets. The results of our study highlight the complex relationships between population dispersals and climate change, and can provide testable hypotheses for extant species expected to experience substantial biogeographic impacts from rising temperatures.

## Abstract (web summary)

Pleistocene population dynamics can inform the consequences of current climate change. This phylogeography of 35 complete American mastodon mitochondrial genomes suggests distinct lineages in this species repeatedly expanded northwards and then went locally extinct in response to glacial cycles.

## Introduction

Anthropogenic climate change is causing considerable increases to the earth’s mean surface temperatures^1^ and ecological stress^2^. As a consequence, many species are experiencing demographic declines or extinction, or are shifting their ranges into regions that have become more habitable given new environmental conditions^3–6^. Although human practices have been the primary cause of global temperature changes over the past century, the phenomenon of large-scale, climatically driven environmental change has occurred numerous times, at varying temporal scales, during the Quaternary period (the last 2.6 million years). The largest changes involved the alternation of glacial and interglacial intervals, which for the past 800 thousand years (ky) have operated on cycles of ~100 ky^7^. These cycles resulted in periodic ice-sheet expansion across ~50% of the habitable land in North America^8^ and fluctuations in global mean annual temperatures of greater than 10 °C^9–11^.

The high-magnitude climate changes associated with glacial–interglacial cycles also resulted in dramatic rearrangement of North American terrestrial ecosystems and vegetation zonation^12^. Furthermore, these climate changes had substantial impacts on the amount of habitable land available as glacial formation and advance, coupled with global sea level drop, enabled new continental shelf lands to emerge during Pleistocene cold periods. Inversely, periods of climatic warming, such as those during previous interglaciations, resulted in climatic and biogeographic configurations across the continent similar to those experienced today. Examining the phylogeographic and demographic impacts of these major climatic oscillations on ancient populations can inform our understanding of how species respond to this scale of change and aid in the construction of predictive frameworks.

Ancient DNA recovered from fossil bones and teeth enables us to directly examine the genetics of extinct species over long periods of time. These methods can provide a nuanced understanding of responses (e.g., migration and extinction) to climatic stressors during the Pleistocene, and have already revealed demographic trends that are not easily recovered with traditional palaeontological techniques^13–16^. Most phylogeographic studies of North American Pleistocene taxa have focused on species adapted to grassland or steppe-tundra, and their responses to the arrival of humans and terminal Pleistocene warming^13,17–19^. Yet past climate change, particularly intervals of sharply increasing temperatures such as that which occurred during Marine Isotope Stage (MIS) 5e (Sangamon interglacial) ~125,000 years ago (kya), is also likely to have had substantial impacts on megafaunal populations^14,20,21^. This kind of climate-driven pressure would have especially applied to species adapted to forests and mixed woodland habitats, because these biomes, which greatly expanded during warmer interglacial intervals, were subsequently replaced or rendered inaccessible during subsequent glaciations^12,20^.

American mastodons (*Mammut americanum*) were an iconic part of wooded and swampy habitats in Pleistocene North America^22–24^, with remains recovered from the Central American subtropics to the Arctic latitudes of Alaska and Yukon^20,25^. Stable isotope data, dental morphology, and microwear analyses reveal some regional and chronological variation or flexibility in diet, although C_3_ woody browse vegetation (e.g., spruce trees) seems to have been preferred^25–28^. Like most proboscideans, the mastodon was a keystone species and served an important role in maintaining the integrity and diversity of its preferred habitats^25,29,30^.

According to recent palaeontological investigations, mastodons and mammoths displayed contrasting responses to cyclical glacial–interglacial climatic shifts. Temporal analyses of mastodon distribution patterns within the American midcontinent^31^ and eastern Beringia (present-day unglaciated areas of Alaska and Yukon)^20^ have inferred that American mastodons briefly expanded into high latitudes during the last interglaciation (MIS 5), but underwent regional extirpation when climates became much colder during the last glaciation (i.e., MIS 4 to MIS 2), surviving thereafter only in lower-latitude temperate regions in North America. These extirpations were likely caused by climate-driven changes in vegetation at the onset of glaciation, which, by contrast, favoured the spatial expansion of mammoths and other grazing species adapted to steppe-tundra. However, these arguments remain difficult to test empirically. This is particularly the case for eastern Beringia where there is often little or no chronostratigraphic context for mastodon fossils, and which regularly return age estimates greater than the effective limit of radiocarbon (^14^C) analysis (i.e., >50,000 years ago)^20^. Alternative dating methods, such as optically stimulated luminescence, may eventually prove useful but have yet to be applied to questions like these.

Here, we present an alternative approach to testing models of expansion–extirpation due to glacial–interglacial cycling, using detailed phylogeographic analysis and Bayesian clock dating of American mastodon mitochondrial genomes. Our findings suggest that American mastodons repeatedly expanded into northern latitudes in response to interglacial warming. However, we also note that northern clades have extremely low levels of genetic diversity, highlighting an important consideration for the conservation of modern species exhibiting similar dispersal patterns.

## Results

### Mastodon phylogeography

**Fig. 1:**
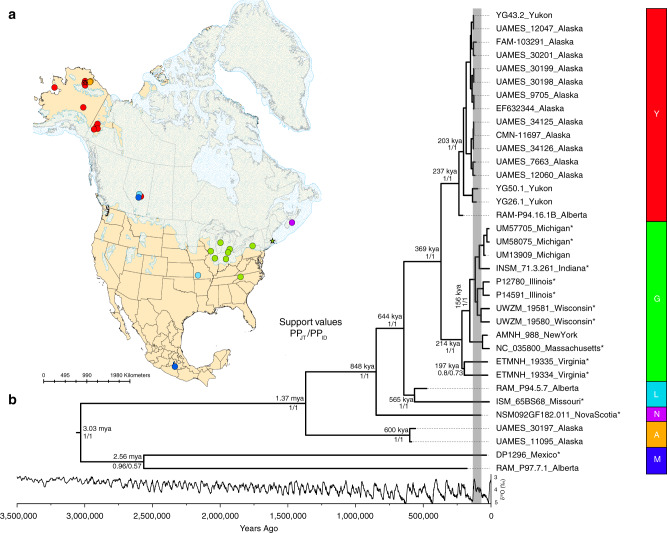
Phylogeographic relationships of American mastodons. **a** Locations of specimens included in this study. Circles indicate specimens for which complete mitochondrial genomes were obtained, coloured according to their clade assignment. Stars indicate previously sequenced specimens (EF632344, Alaska; NC_035800, Massachusetts). The locations of specimens from Alberta and eastern Beringia have been jittered to aid visualisation (see Supplementary Fig. 2 for an unmodified version). **b** Phylogenetic tree inferred by the Joint analysis. Median posterior ages are given for major nodes in the tree. Support values for each node represent posterior probabilities from the Joint (PP_JT_) and Individually Dated (PP_ID_) analyses. The δ^18^O record for the last 3.5 million years is shown below the tree^7^, with the approximate extent of the MIS 5 interglacial period shaded in grey. Clades identified in this study are designated by colour and named where possible by the geographic provenance of their members (i.e., A Alaska, Y Yukon, G Great Lakes, M Mexico, N Nova Scotia, L Alberta/Missouri). Specimens with finite radiometric or geological ages are indicated with asterisks.

Subsamples from fossil bones and teeth of American mastodons were obtained from museums, universities, and government institutions across North America (Supplementary Data 1). Complete mitochondrial genomes were sequenced from 33 of 122 specimens (~27% success rate), with completeness defined as >80% sequence coverage of the *M. americanum* mitochondrial reference (GenBank accession NC_035800), at a minimum coverage depth of 3× (Fig. 1a; Supplementary Fig. 2). Alignments contained 1492–51,113 uniquely mapped reads, with the short inserts (mean fragment sizes 37.26–76.63 bp) and terminal cytosine deamination that are characteristic of authentic ancient DNA (Supplementary Methods—Sequence Authenticity and Map Damage). Partial sequences were also obtained from another 12 specimens, ranging from 46 to 580 uniquely mapped reads, but these were excluded from all subsequent analyses (Supplementary Table 31).

We identified five well supported major clades in the mitogenomic phylogeny (Fig. 1b), through a combination of maximum-likelihood and Bayesian methods (Supplementary Methods—Phylogenetic Analyses). We named the five clades by the approximate geographic provenance of their constituent specimens (i.e., A Alaska, Y Yukon, G Great Lakes, M Mexico, L Alberta/Missouri). We included two specimens from Virginia (southeastern USA; ETMNH 19334 and ETMNH 19335) in Clade G, due to the lower support for their monophyly. We also tentatively assigned a single specimen, NSM092GF182.011 from Nova Scotia (eastern Canada), dated to 74.9 ± 5.0 ky^32^, to a separate Clade N. This specimen is geographically and temporally distinct; given the deep divergence that we estimate for this lineage, it is likely to represent a separate group of east coast mastodons from the Sangamon interglacial of MIS 5.

The phylogenies inferred by Bayesian and maximum-likelihood methods were consistent, except for the placements of the two specimens in Clade M (DP1296 and RAM P97.7.1). When the tree is rooted with a *Mammuthus* (mammoth) outgroup, Clade M is rendered paraphyletic, albeit with low bootstrap support (79%). However, the monophyly of this clade is supported by midpoint-rooted trees inferred using maximum likelihood, as well as by Bayesian methods (Fig. 1b; Supplementary Methods—Phylogenetic Analyses).

We find evidence of broad phylogeographic structure, with mastodons from neighbouring localities generally being more closely related. This trend is also observed in North American mammoths^33^, as well as in African^34^ and Asian elephants^35^, and is due to the matrilocal nature of proboscidean herds. A matrilocal herd structure for mastodons has also previously been argued based on difference in tusk growth profiles between males and females upon reaching sexual maturity^36^, and relationships in preserved trackways^37^. Notably, female proboscidean philopatry also results in deep divergences between clades^38^ and possibly explains the deep divergences that we observe in mastodons.

Despite the limited geographic dispersal expected within proboscidean matrilines, we identify two independent and genetically divergent clades (A and Y) that consist primarily of specimens from eastern Beringia. Clade Y is grouped with Clades G, L, and N, and diverged from Clade A between 1.37 million years ago (95% HPD interval: 857–1881 kya, when the ages of all undated ancient samples are estimated simultaneously in a “Joint” analysis) and 609 kya (95% HPD interval: 335–998 kya, when the ages of undated ancient samples are “Individually Dated”).

Specimens from Alberta are found in three of the five well-defined clades (L, M, and Y; Fig. 1), highlighting a complex ecological and biogeographical history that could not have been recovered from the palaeontological record alone. Notably, central Alberta was the site of maximum convergence of the Laurentide and Cordilleran ice sheets, and contained the earliest deglaciated corridor connecting areas north and south of the ice sheets following latest Pleistocene deglaciation^8^. Previous work on *Bison* has shown that this region was also the site of dynamic range changes in response to this last deglaciation episode, with rapid population expansions into the region from both Beringian and southern areas coinciding with glacial retreat^16,19^. While further research is required to determine whether these findings also apply to other taxa and time periods, the phylogenetically divergent placements of Alberta mastodons suggest that this region was one of immense biological fluidity for this species as well.

### Mastodon sample-age estimation

**Fig. 2:**
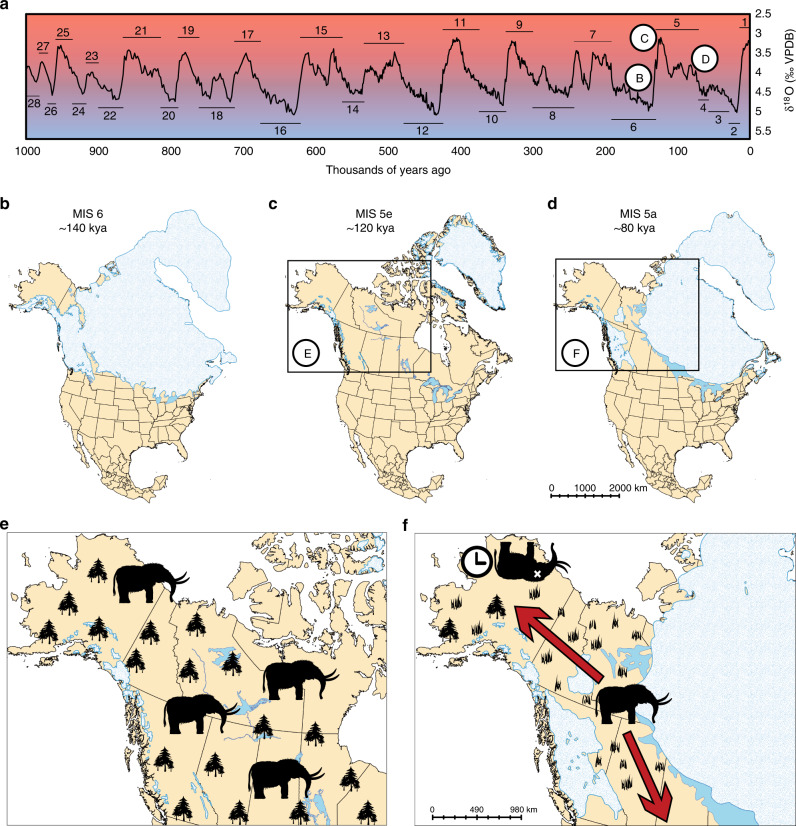
Model of mastodon extirpation and expansion in response to glacial cycles. **a** Global stack of benthic foraminifera δ^18^O for the last 1 million years, which tracks changes in deep-water temperature and global ice volume. The *y*-axis has been inverted so that periods of low ice buildup (and higher temperatures—red) are at the top of the graph, and periods of greater ice buildup (and lower temperatures—blue) are at the bottom. Marine Isotope Stage (MIS) extents are indicated with black bars above (interglacials) or below (glacials) the δ^18^O record. δ^18^O values and MIS terminations can be found in Lisiecki and Raymo^7^. One full glacial cycle is represented, showing the change from glacial (**b**) to interglacial (**c**) conditions, followed by a fall back into another glaciation (**d**). North American ice-sheet cover at each stage (**c**, **d**) is approximated from recorded δ^18^O to similar conditions during the transition out of the last glaciation^8^, or from published simulations where available (**b**)^65^. The ecological implications of these transitions are summarised in (**e**, **f**), with mastodons being able to occupy most of eastern Beringia and Canada during interglacials (**e**), but progressively extirpated from these regions as conditions descend into the next glacial period (**f**). Populations would either need to retract to unglaciated regions south of the ice sheets or north to temporarily unglaciated refugia which would be unlikely to support mastodon populations throughout long glaciations.

To explain the extirpation of American mastodons in eastern Beringia during the last glaciation, Zazula et al.^20^ proposed a palaeoecological model that tied mastodon occupation generally to the MIS 5 interglacial, when the regional vegetation would have been dominated by mixed boreal forests and wetlands^39^. Assuming that mastodon presence in Beringia varied with vegetation type, the question becomes whether there was a repeated pattern of mastodon expansion and extirpation corresponding to the ~100 ky glacial–interglacial cycle (Fig. 2). Evidence for extending the palaeoecological model in this way should include both a temporal signal (i.e., high-latitude mastodon presence should correlate with known interglacial intervals) and a biogeographic signal (i.e., high-latitude populations ought to display lower levels of genetic diversity than southern populations, as a consequence of restricted subsets of matrilocal herds expanding northward as environmental conditions ameliorated).

Mastodon specimens from eastern Beringia and Alberta were determined by radiocarbon analysis to be greater than 50,000 years old, or analytically nonfinite, and are often found out of stratigraphic context^20,40^. In the absence of any other applicable direct-dating method associated with a large date archive, we used a molecular clock analysis^41,42^ to estimate the ages of high-latitude specimens. Although molecular dating tends to produce date estimates that are less precise than those of some radiometric or geological methods, its accuracy has been demonstrated in studies of simulated and molecular data^41^ as well as morphological data^42^. Additionally, in cases where little to no temporal information is available, estimating the ages of undated specimens allows the inclusion of these specimens when they would otherwise need to be excluded from molecular dating analyses. We used two separate approaches to estimate the ages of the undated specimens: one where the dates of all specimens were estimated simultaneously (Joint; JT), and one where we estimated the dates of specimens individually before analysing all of the samples together (Individually Dated; ID).

**Fig. 3:**
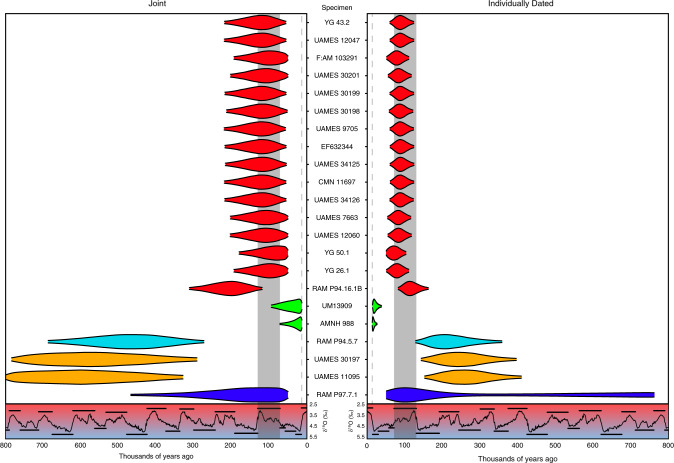
Marginal posterior densities of specimen ages in molecular dating analyses. Specimens are coloured by clade and arranged as in Fig. 1b. Each violin represents the 95% HPD interval of each specimen age estimated in the Joint (left) and Individually Dated (right) analyses. The δ^18^O record for the last 800 thousand years is overlaid below both plots and corresponds to the one in Fig. 2a, with the approximate extent of the MIS 5 interglacial shaded in grey. The dashed grey line at 13 kya represents the minimum bound imposed by the youngest specimen in the analyses.

Median posterior ages for the east Beringian Clade Y mastodons ranged between 98–130 ky (JT) and 74–91 ky (ID) (Fig. 3), falling within the boundaries of the MIS 5 interglacial, the last major extended warm period prior to the Holocene^7^. Although the 95% HPD intervals of the age estimates for individual samples are wide (combined Beringian mastodon 95% HPD range—JT: 50–219 ky; ID: 50–125 ky), their probability density is concentrated around times that correspond well with MIS 5, with the mode of each distribution also being located within the timespan of the MIS 5 interglaciation (Supplementary Table 42). Additionally, while the Joint analysis also includes a small subset of ages corresponding to the previous interglacial (MIS 7; ~191–243 kya)^7^ within some specimens’ 95% HPD interval, these ages are not recovered in the Individually Dated analysis. These findings strongly suggest that mastodon habitation in Eastern Beringia coincided with interglacial periods, as expected under our palaeocological model.

East Beringian mastodons in Clade A had much older posterior age estimates than Beringian mastodons in Clade Y. Median ages for UAMES 11095 were 586 ky (JT 95% HPD interval: 329–800 ky) and 267 ky (ID 95% HPD interval: 152–410 ky), and median ages for UAMES 30197 were 558 ky (JT 95% HPD interval: 292–784 ky) and 254 ky (ID 95% HPD interval: 142–397 ky). However, the 95% HPD intervals of the ages of these specimens were much wider than those of specimens in Clade Y, spanning many more glacial and interglacial periods, and making it difficult to tie them to any specific marine isotope stage. Nevertheless, the ages of both specimens have 95% HPD intervals that do not overlap with those of east Beringian mastodons in Clade Y, which suggests that mastodons in these two clades are temporally distinct. Combined with their divergent positions in the phylogeny, these results are consistent with Clade A mastodons being part of a separate colonisation event during an earlier interglaciation.

The Joint and Individually Dated analyses both estimate young ages for two mastodon individuals in Clade G. AMNH 988 has a median posterior age of 28 ky (JT 95% HPD interval: 13–71 ky) and 17 ky (ID 95% HPD interval: 13–27 ky), while UM13909 has median posterior ages of 43 ky (JT 95% HPD interval: 13–94 ky) and 21 ky (ID 95% HPD interval: 14–38 ky). In all analyses, however, the posterior age densities for these specimens have greater probability mass toward younger values and abut the lower bound at 13 ky, a hard limit based on the age of the youngest specimen in the dataset, INSM 71.3.261. These results are broadly consistent with the expected ages of these two specimens, given the radiometric and geological ages of other mastodons in this clade. Nevertheless, the shapes of the posterior age distributions suggest that these specimens might actually be younger than 13 ky.

Alberta mastodons were estimated at a range of ages, congruent with an interpretation of highly dynamic biogeographic landscape characterised by population turnover. However, we note that the limited number of specimens from this region, the wide 95% HPD intervals of their ages, and their scattered positions across the phylogeny make it difficult to tie them to specific periods. Specimen RAM P94.16.1B had a greater median posterior age (JT: 208 ky; ID: 117 ky) than other mastodons within its clade, although its 95% HPD interval overlapped those of other Clade Y mastodons (JT: 119–311 ky; ID: 82–163 ky). The widths of the 95% HPD intervals and their overlap also varied between the two analyses, and in their association with either the MIS 5 or MIS 7 interglacial. However, should this specimen ultimately be shown to date to MIS 7, it would suggest successive colonisation events from the same or a similar source population.

The Alberta sample RAM P94.5.7 had a median posterior age estimate similar to those of mastodons in Clade A (JT: 474 ky; ID: 221 ky), but also with a wide 95% HPD interval that makes it difficult to associate with any particular marine isotope stage. However, unlike the separation between Beringian mastodons in Clade Y and Clade A, the 95% HPD intervals of the ages of RAM P94.16.1B and RAM P94.5.7 do overlap by 37 ky (JT) and 35 ky (ID), making their separation more uncertain.

The posterior density of the age of sample RAM P97.7.1 had a mode within the MIS 5 interglacial age boundaries (Supplementary Table 42), but with a very wide 95% HPD interval (JT: 50–467 ky; ID: 50–763 ky). As with mastodons in Clades A and L, this pattern is likely to be due to its phylogenetic position and deep divergence from the majority of calibration points in the dataset.

### Genetic diversity within Mastodon clades

Under a model of repeated expansion and extirpation, northern clades of mastodons would be expected to have lower levels of genetic diversity. This pattern would be consistent with repeated expansion of small founder matriarchal herds in response to climatic warming during interglaciations, and the transient nature of their occupation of northern latitudes. Genetic diversity is expected to be higher among samples from regions south of the continental ice sheets that were likely to have been inhabited by populations of mastodons throughout the Pleistocene. We examined levels of nucleotide diversity within our dataset to test this hypothesis.

**Fig. 4:**
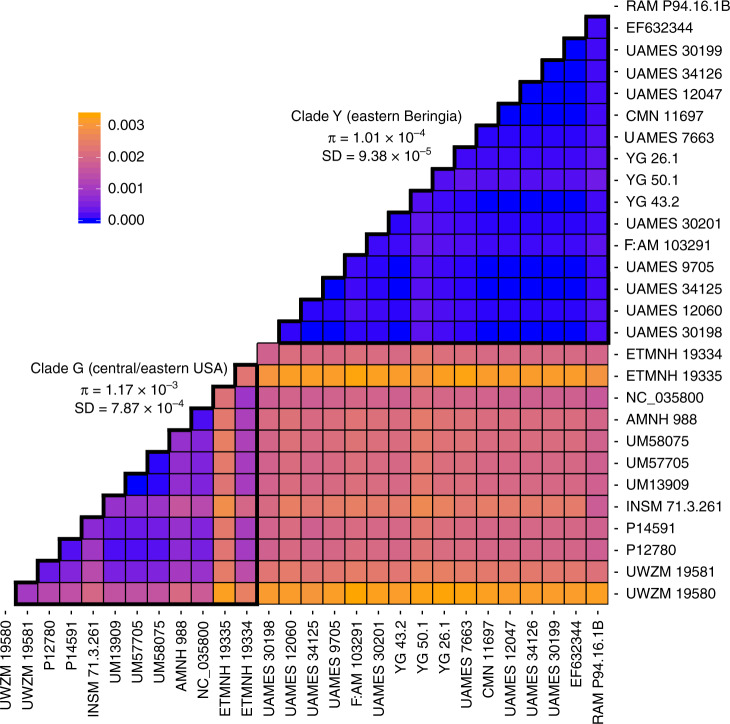
Pairwise genetic distance heatmap for all samples in Clades Y and G. Pairwise genetic distances for all samples in Clade G (including ETMNH 19335 and ETMNH 19334) and Clade Y (including RAM P94.16.1B). Genetic distances are calculated using the F84 model of nucleotide substitution. Nucleotide diversity within each clade (outlined with thick black borders) is indicated. All values are given in substitutions per site.

Clade Y had a low nucleotide diversity (*π*) of *π* = 8.79 × 10^−5^ substitutions per site (standard deviation (SD) = 8.9 × 10^−5^ substitutions per site) or 1.01 × 10^−4^ substitutions per site (SD = 9.38 × 10^−5^ substitutions per site) when including the potentially temporally distinct Alberta specimen (RAM P94.16.1B) (Fig. 4). The genetic distance between the two mastodons in clade A was also quite small (1.24 × 10^−4^ substitutions per site). By comparison, there were much higher levels of nucleotide diversity in clade G, which contains mastodons from south of the ice sheets, their endemic range. This was the case when either including (*π* = 1.17 × 10^−3^ substitutions per site; SD = 7.87 × 10^−4^ substitutions per site) or excluding (*π* = 8.09 × 10^−4^ substitutions per site; SD = 5.31 × 10^−4^ substitutions per site) the two mastodons from Virginia. This is consistent with expectations of small numbers of matrilocal mastodons expanding northward in response to glacial retreat, and also supports previous palaeoecological models of environments inhabited by northern mastodons.

## Discussion

Our sequencing and analysis of 33 mitochondrial genomes of the American mastodon, *Mammut americanum*, provides a framework for interpreting the genetic diversity of the species across space and time. We have identified six mitochondrial clades that span nearly the entire North American continent, from Alaska to Mexico. The two predominantly eastern Beringian clades are likely to have originated from separate expansions of mastodons into the region. We also note that the individuals from Alberta are distributed across the phylogenetic tree, highlighting the dynamic nature of American mastodon dispersal between southern and northern latitudes. Our proposed clade nomenclature may need revision as further mitochondrial genomes are sequenced, and as geographic and temporal gaps are filled. This applies especially to specimens in Clade M, which have a deep coalescence time and may stand as sole representatives of multiple lineages that are deeply divergent from the remaining mastodons.

Our analyses add further support for the model^20^ that proposed that American mastodons only occupied higher latitudes (i.e., Canada and Alaska) during interglacials, when prevailing warm climatic conditions supported the establishment of forests and wetlands. The presence of temporally distinct clades in Alaska and Yukon indicates that the inferred pattern of expansion during warm interglaciations, followed by local extirpations and range contraction to the south during colder times, was likely to have been a recurring scenario. We infer that this was a major, and perhaps widespread, biological response to global glacial-interglacial cycling that affected many species in eastern Beringia (e.g., western camel *Camelops hesternus*;^21^ giant beaver *Castoroides ohioensis*^43^). Similar processes presumably occurred in Eurasia, with warm-adapted species such as hippopotamuses and hyaenas episodically expanding their ranges northward during earlier interglaciations into previously ice-dominated areas like the British Isles and Scandinavia^44–46^. However, this pattern also poses further questions: for example, why were species that had managed to repeatedly expand into the northernmost parts of North America during previous interglacials unable to do so following the return to interglacial conditions after the last glacial maximum (~21 kya)? Were they already in severe decline? More critically, will similar trends be seen in extant browsers?

At present, numerous bird^5^, fish^4^, and mammal communities^47,48^ in northern North America are undergoing rapid restructuring in response to climatic warming. Moose^47^ and beavers^48^, iconic members of present-day northern boreal forest, have expanded their ranges northward by hundreds of kilometres in the last few decades alone. Our data suggest that regional expansion of at least some southern, temperate populations into northern latitudes is a probable outcome of the warmer and wetter conditions of today. However, populations at the expansion front are likely to be a subset of the current global diversity of these species, leaving them vulnerable if more genetically diverse southern populations are eventually lost. The phylogeographical history of Pleistocene megafauna can serve as a useful example for understanding the ecological responses of present-day species, and can generate testable hypotheses about the consequences of anthropogenic environmental impacts.

## Methods

### Sample acquisition and subsampling

Mastodons were subsampled at each of the institutions that contributed specimens to this study (Supplementary Data 1). Subsamples were then sent to the McMaster University Ancient DNA Centre, with all further processing conducted in dedicated ancient DNA clean rooms.

### DNA extraction and processing

Specimens were processed using a variety of wet-lab methodologies in an attempt to maximise the probability of successful DNA extraction, and as new techniques were developed and modified to overcome issues with DNA recovery, inhibition, and poor endogenous preservation. Between 30 and 349.9 mg of material were demineralised and digested in successive rounds with 0.5 M EDTA or a Proteinase K digestion buffer. Supernatants were pooled and extracted using either organic or two different guanidinium-silica based extraction methods^49,50^. UDG-treated and non-UDG-treated libraries were prepared using double-stranded^51,52^ or single-stranded methodologies^53,54^, with some modifications from in-house optimisation. Following indexing, all libraries underwent 1–2 rounds of in-solution enrichment with a comprehensive proboscidean bait set^33^, to increase the relative abundance of the degraded endogenous fraction. Full methods for each sample are given in Supplementary Data 1 and the Supplementary Methods.

### Sequence mapping and curation

Demultiplexed reads were trimmed and merged with leeHom^55^, using the ancient DNA flag, and the double-stranded or single-stranded library adaptor sequences as appropriate. Reads were then mapped against the *M. americanum* mitochondrial reference genome (NC_035800) using a network-aware version of BWA^56^ (https://github.com/mpieva/network-aware-bwa) with common ancient DNA settings: maximum edit distance of 0.01 (−n 0.01), a maximum of two gap openings (−o 2), and seeding effectively disabled (−l 16500). Mapped reads that were either merged or properly paired were extracted using the retrieveMapped_single_and_ProperlyPair programme of libbam (https://github.com/grenaud/libbam). Replication duplicates were then removed based on unique 5′ and 3′ positions (https://bitbucket.org/ustenzel/biohazard/src/master/), and filtered to remove sequences below a minimum length of 24 bp and mapping quality of 30. Specimens with multiple libraries were combined at this point, and underwent additional duplicate removal if they contained the same index pair (Supplementary Methods—Reference Guided Mapping).

Alignments were imported into Geneious v6.1.5 and manually curated to remove any sequencing artefacts. Regions below our requirement of 3× minimum depth coverage were masked with Ns. Due to stacking observed in conserved mitochondrial regions (e.g., 16S rRNA, D-loop), we further masked any positions with coverage depths greater than three standard deviations from the mean that displayed multiallelic variants (Supplementary Methods—Sequence Curation). The variable number tandem repeat (VNTR) region of each sequence was masked with Ns in accordance with the NC_035800 reference. Final consensus sequences were obtained using the majority base call at each position.

### Model selection and phylogenetic analyses

The 33 new mitochondrial genomes were aligned with the only two mastodon mitochondrial genomes previously published, MAS1 (NC_035800) and IK-99-237 (EF632344), using MUSCLE v3.8.31^57^. Model selection was performed using jModelTest v2.1.4^58^ with the corrected Akaike information criterion. The HKY + G model was chosen for all subsequent analyses.

The phylogeny was inferred by maximum-likelihood analysis using IQ-TREE v1.6.6^59^. We used two approaches to root the tree, either by midpoint-rooting or by including an outgroup comprising two mammoth mitochondrial genomes (NC_007596 and NC_015529). In each case, node support was estimated using 1000 bootstrap replicates.

Both of the sequence alignments were also used for Bayesian phylogenetic inference in BEAST v.1.8.0^60^ using an HKY + G4 model, a constant population size tree prior, a strict clock model, and default specifications for all priors (Supplementary Methods—Phylogenetic Analyses). The analysis was run for 10 million steps sampling every 1000 states.

### Sample-age estimation

We compared coalescent tree priors and clock models by calculating their marginal likelihoods using generalised stepping-stone sampling^61^ (Supplementary Methods—Model Testing With GSS) in BEAST v.1.10.5^62^. We ran two separate age-estimation analyses. In the first approach, we jointly estimated the ages of all of the undated specimens in a single analysis (Joint; JT). We specified gamma prior distributions (shape = 1; scale = 200,000) for the ages of the undated samples. The ages of these samples were also bounded at 800 ky (the upper limit of successful ancient DNA recovery) and at 50 ky (the approximate limit of radiocarbon dating), with the exception of AMNH 988 and UM13909 which had a lower limit of 0 ky. In all cases, bounds were scaled relative to the age of the youngest specimen in the dataset (INSM 71.3.261 at 13 ky). Samples with known radiocarbon ages were calibrated using Calib v7.0.4^63^, then the ages of these specimens were treated as point values based on their median ages.

In our second approach, we estimated the age of each specimen individually in an analysis that included the dated specimens. We then used the marginal posterior densities of the ages of the individual samples to specify the priors for the ages of these samples in a combined analysis of all samples (Individually Dated; ID).

Both analyses were run with the HKY + G substitution model and empirical base frequencies. We chose a constant population size tree prior and a strict clock model, with uniform distributions for the associated population size prior (Uniform [1, 1 × 10^6^]) and the substitution rate prior (Uniform [4 × 10^−10^, 8 × 10^−8^]), while all other remaining priors were left at their default distributions. To allow for more efficient sampling of unknown specimen ages, their weight was increased to 5. The chain length was increased to 500 million steps (sampling every 10,000), and all analyses were run in duplicate.

### Nucleotide diversity

Pairwise distances between all American mastodon mitochondrial genomes were calculated using the dist.dna() function in the R package ape^64^. Distances were calculated using the F84 model. Sites with missing data were deleted in a pairwise manner. For clades containing more than two mastodons, nucleotide diversity was calculated as the mean of all pairwise distances between specimens in that clade.

### Reporting summary

Further information on research design is available in the Nature Research Reporting Summary linked to this article.

## Supplementary information

Supplementary Information

Peer Review File

Reporting Summary

Description of Additional Supplementary Files

Supplementary Data 1

## Data Availability

Mastodon specimens examined in this study were obtained from the external institutions listed in Supplementary Data 1. All requests for access to the material should be made to the external institution that houses the material. Final consensus sequences for all complete mastodon specimens have been uploaded to NCBI with GenBank accessions MN616941–MN616973 (Supplementary Table 1). Raw sequencing reads for each complete mitochondrial genome were also uploaded to the SRA (BioProject: PRJNA578413). New radiocarbon dates are reported in Supplementary Table 44. Two previously published American mastodon mitochondrial genomes (GenBank accessions NC_035800 and EF632344) were also analysed in this study. Two mammoth mitochondrial genomes (NC_007596 and NC_015529) were used in some phylogenetic analyses as outgroups.

## Code availability

No new software was generated during the course of this study. Custom scripts used to produce nucleotide diversity graphics are available are available at: https://github.com/ekarpinski/MastoScripts.
