# Supplementary material for: American mastodon mitochondrial genomes suggest multiple dispersal events in response to Pleistocene climate oscillations
Source: Nat Commun. 2020 Sep 1;11:4048. doi: 10.1038/s41467-020-17893-z (PMC7463256; doi:10.1038/s41467-020-17893-z)
Supplement: Supplementary file 1 — Supplementary Information [file 41467_2020_17893_MOESM1_ESM.pdf]

Supplementary Information:

American mastodon mitochondrial genomes suggest multiple dispersal events in response to Pleistocene climate oscillations

Karpinski et al.

Table of Contents

Section	Page
**Supplementary Methods**	
Complete Specimen Catalog Numbers and GenBank/SRA Accessions	3
Extraction Methodologies	4
Library Preparation Methodologies	7
Enrichment Methodologies	13
Pooling, Size Selection, and Sequencing Methodologies	16
Reference Guided Mapping	17
Sequence Curation	17
Sequence Authenticity and Map Damage	21
Phylogenetic Analyses	31
Sequence and Topology Validation	37
Leave-One-Out Analysis	46
Temporal Signal Verification with BETS	52
Model Testing With GSS	52
Specimen Age Estimation	55
Genetic Diversity	75
New Radiocarbon Dates	77
**Appendix A**	78
**Supplementary References**	116

Complete Specimen Catalog Numbers and GenBank/SRA Accessions

Supplementary Table 1. Catalog numbers, specimen alias; GenBank Accession numbers, and SRA BioSample Accessions of each new complete American mastodon specimen in this study (BioProject: PRJNA578413).

Catalog Number	Specimen Aliases	GenBank Accession	BioSample Accession
UAMES 12047	IK-01-321	MN616972	SAMN13061045
NSM092GF182.011	CCM-1	MN616956	SAMN13061043
UAMES 30201	IK10-106	MN616968	SAMN13061046
AMNH 988	-	MN616953	SAMN13061044
CMN 11697	-	MN616957	SAMN13061040
P 12780	-	MN616967	SAMN13061056
UAMES 34126	MAY12-70	MN616970	SAMN13061053
UM13909	-	MN616958	SAMN13061067
UAMES 11095	IK-99-328	MN616960	SAMN13061050
YG 26.1	-	MN616971	SAMN13061066
UAMES 9705	IK-98-963	MN616961	SAMN13061047
UAMES 7663	-	MN616952	SAMN13061062
YG 43.2	-	MN616969	SAMN13061068
UM58075	-	MN616963	SAMN13061065
UM57705	-	MN616959	SAMN13061069
RAM P97.7.1	-	MN616951	SAMN13061064
YG 50.1	-	MN616945	SAMN13061071
UAMES 30198	IK08-127	MN616943	SAMN13061055
UAMES 30197	IK05-3.5	MN616965	SAMN13061041
UAMES 30199	KIG12-15	MN616973	SAMN13061051
RAM P94.16.1B	-	MN616949	SAMN13061061
RAM P94.5.7	-	MN616950	SAMN13061063
UAMES 12060	IK-01-277	MN616954	SAMN13061048
UAMES 34125	MAY12-69	MN616955	SAMN13061052
INSM 71.3.261	Buesching; “Beusching”	MN616944	SAMN13061057
F:AM 103291	-	MN616964	SAMN13061042
P 14591	-	MN616946	SAMN13061070
ETMNH 19335	SV57_A1A4, SV 5/7 A1A4	MN616947	SAMN13061072
ETMNH 19334	SV57_A1A3, SV 5/7 A1A3	MN616966	SAMN13061060
ISM 65BS68	ISM2015-58	MN616941	SAMN13061059
UWZM 19581	ISM2015-53	MN616962	SAMN13061049
UWZM 19580	ISM2015-54	MN616942	SAMN13061058
DP1296	-	MN616948	SAMN13061054

Extraction Methodologies

Over the duration of this study mastodon specimens were processed using a variety of techniques to maximize DNA recovery. Methodologies changed as a result of in-house testing and optimization and as new methodologies were described in the literature.

Extraction Method A

Manually crushed samples were demineralized with 0.75 ml of 0.5 M EDTA for approximately 24 hours at 25°C with shaking at 400 RPM. Samples were spun down for 5 minutes at 16.1 kG, and the supernatant removed. Samples were then digested with 500 µl of a Proteinase K digestion buffer (Supplementary Table 2) for 1.5 hours at 50°C with rotation. Samples were spun down for 5 minutes at 16.1 kG, and the supernatant removed. Samples underwent a second round of both demineralization and digestion with 650 µl 0.5 M EDTA for 24 hours, and 400 µl Proteinase K for 3 hours respectively.

Round 2 demineralization and digestion supernatants were extracted with 2x 900 µl of PCI (phenol/chloroform/isoamyl alcohol) (pH 8) and 2x 600 µl of chloroform separately. Purified aqueous phases were then concentrated over pre-wet (400 µl 1x TE) 30 kDa Amicon ultra 0.5 Centrifugal Filter tubes (EMD Millipore) and washed three times with 1x TE.

Supplementary Table 2. Final concentrations of all components in the proteinase K digestion solution. Samples were digested with 500µl of digestion solution for 1.5 hours at 50°C with rotation. Water (not shown) was used to bring the volume of the reaction up to a desired amount.

ProK Digestion Solution	
**Component**	**Final Concentration**
Tris-Cl (pH 9.0)	0.01 M
Sarcosyl	0.5 %
ProK	0.25 mg/ml
CaCl2	0.005 M
DTT	50 mM
PVP	1 %
PTB	2.55 mM

Extraction Method B

Crushed samples were demineralized twice using 1 ml of 0.5 M EDTA for 16 to 24 hours at 25°C with shaking at 400 RPM. Demineralization was carried out using 400-500 µl of a simplified Proteinase K digestion buffer (Supplementary Table 3) for 1.5 to 3 hours (50°C with rotation). Demineralization and digestion rounds were interspersed, and all supernatant was removed between each round.

Round 2 demineralization and digestion supernatants were extracted using 800 µl of PCI and 500 µl of chloroform, and 500 µl each of PCI and chloroform respectively. Aqueous phases were concentrated on separated 30 kDa Amicon ultra 0.5 Centrifugal Filter tubes and washed three times with 0.1x TE.

Supplementary Table 3. Final concentrations of all components in the proteinase K digestion solution. Samples were digested with 400-500µl of digestion solution for 1.5 to 3 hours at 50°C with rotation. Water (not shown) was used to bring the volume of the reaction up to a desired amount.

ProK Digestion Solution	
**Component**	**Final Concentration**
Tris-Cl (pH 9.0)	0.01 M
Sarcosyl	0.2 %
ProK	0.25 mg/ml
CaCl2	0.005 M

Extraction Method C

Samples underwent an initial demineralization with 0.5 ml of 0.5 M EDTA for 16 to 18 hours (25°C; shaking at 400 RPM). The supernatant was then removed and 1 ml of 0.5 M EDTA was added for 24 to 26 hours (25°C; shaking at 400 RPM). Samples were then digested with the Proteinase K buffer outlines in Supplementary Table 3 (500 µl; 45°C; rotation).

Digestion and round 2 demineralization supernatants were separately extracted using 800 µl of PCI and 500 µl of chloroform. Purified aqueous phases were concentrated on 30 kDa Amicon ultra 0.5 Centrifugal Filter tubes and washed three times with 0.1x TE

Sample 50003/16643 (extraction ID 545) underwent an additional demineralization (700 µl 0.5M EDTA) and digestion round (300 µl; Supplementary Table 3) due to minimal visible reduction in previous rounds. Supernatants from both additional rounds were combined and extracted using 900 µl of PCI and 500 µl of chloroform. The aqueous phase was concentrated on 30 kDa Amicon ultra 0.5 Centrifugal Filter tubes as above.

Extraction Method D

Samples underwent two successive rounds with 500 µl and 1 ml of 0.5 M EDTA respectively (25°C; shaking at 400 RPM). Samples were then digested with 500 µl of a simplified Proteinase K buffer (Supplementary Table 3) for 3 to 4 hours (45°C; rotation).

Round 2 demineralization and digestion supernatants were combined and extracted with 900 µl of PCI and 500 µl of chloroform. Aqueous phases were concentrated over 30 kDa Amicon ultra 0.5 Centrifugal Filter tubes, washing three times with 0.1x TE.

Extraction Method E

Samples underwent a quick initial demineralization with 500 µl of 0.5 M EDTA for 1.5 hours (25°C; shaking at 400 RPM), followed by a 20 hour demineralization with 1 ml of 0.5 M EDTA under identical conditions. Digestion was carried out with 500 µl of a Proteinase K digestion buffer (Supplementary Table 4) at 50°C with rotation for 3 hours.

Demineralization round 2 and digestion supernatants were extracted separately with 900 µl PCI and 600 µl of chloroform. Purified aqueous phases were combined over pre-wet (400 µl 1x TE) 30 kDa Amicon ultra 0.5 Centrifugal Filter tubes (EMD Millipore) and washed three times with 1x TE.

Supplementary Table 4. Final concentrations of all components in the proteinase K digestion solution. Samples were digested with 500µl of digestion solution for 3 hours at 50°C with rotation. Water (not shown) was used to bring the volume of the reaction up to a desired amount.

ProK Digestion Solution	
**Component**	**Final Concentration**
Tris-Cl (pH 9.0)	0.01 M
Sarcosyl	0.2 %
ProK	0.25 mg/ml
CaCl2	0.01 M
DTT	100 mM
PVP	2 %
PTB	5 mM

Extraction Method F

Remaining demineralization and digestion supernatants were combined where multiple unextracted rounds existed per sample. Samples were then purified using 600 µl each of PCI and chloroform, and concentrated over pre-wet (350 µl 1x TE) 30 kDa Amicon ultra 0.5 Centrifugal Filter tubes (EMD Millipore) and washed three times with 1x TE. Concentrated extracts were further purified over MinElute PCR Purification Kit spin columns (Qiagen) using 6:1 volumes of Buffer PB, two washes with 750 µl of PE, and eluted in 15 µl of TEB (Buffer EB with 1 mM EDTA).

Extraction Method G

Samples underwent a quick wash with 300 µl of 0.5 M EDTA for 20 minutes (25°C; shaking at 1000 RPM), to remove residual bone powder and any exterior exogenous material. Samples were then spun down for 5 minutes at 16.1 kG and the supernatant removed.

Samples underwent three alternating rounds each of demineralization and digestion with 750 µl of 0.5 M EDTA (16-24 hours; 25°C; shaking at 1000 RPM) and 750 µl of a Proteinase K digestion buffer (Supplementary Table 5; 1.5-5 hours; 45°C; rotation). Samples were spun down and supernatants were removed at the end of each round.

Supplementary Table 5. Final concentrations of all components in the proteinase K digestion solution. Samples were digested with 750 µl of digestion solution for 1.5 to 5 hours at 45°C with rotation. Water (not shown) was used to bring the volume of the reaction up to a desired amount.

ProK Digestion Solution	
**Component**	**Final Concentration**
Tris-Cl (pH 9.0)	0.01 M
Sarcosyl	0.2 %
ProK	0.25 mg/ml
CaCl2	0.01 M

Extraction was completed with a 5 M guanidinium HCL binding buffer as described in Dabney et al. [1] with the following modifications: 14 ml of the supernatant and binding buffer was continually reapplied to the membrane until demineralization and digestion supernatant was bound; elution was

performed twice with 25 µl of EBT (Buffer EB + 0.05% Tween-20). Supernatants for all rounds were pooled for each sample and extracted over the sample column.

Extraction Method H

Manually pulverized subsamples were washed with 300 µl of 0.5 M EDTA for 20 minutes (25°C; shaking at 1000 RPM), spun down for 5 minutes at 16.1 kG, and supernatant removed.

Samples underwent three rounds of demineralization and digestion with 750 µl each of 0.5 M EDTA (16-24 hours; 25°C; shaking at 1000 RPM) and 750 µl of a Proteinase K digestion buffer (Supplementary Table 5; 1.5-5 hours; 45°C; rotation). Demineralization and digestion rounds were interspersed and all supernatant was removed prior to each new round. Samples DP5247, DP234, DP 885, DP1296, and DP3727 were observed to have minimal dissolution after three rounds of demineralization and digestion. As such they underwent a fourth "long" round of demineralization (750 µl 0.5 M EDTA, 70 hours; 25°C; shaking at 1000 RPM) followed by an additional round of digestion under the above conditions.

Extraction was completed using the Method B gunidinium – silica based extraction outlined in Glock & Meyer [2] with the following modifications: final elution was carried out with 50 µl EBT. Due to extensive inhibitor carry over in this method, samples were further purified over MinElute PCR Purification Kit spin columns with the modifications listed in Extraction Method F. Extracts were combined with 6:1 volumes of Buffer PB and subject to a "cold spin" for approximately 20 minutes (4°C; 22kG) before binding to the silica column. This resulted in substantial pelleting of residual inhibitor carryover. Extracts were washed twice with 750 µl of PE, and eluted in 50 µl of EBT.

Extraction Method I

Samples were treated identically to Extraction Method G with the following modifications: only two alternating rounds of demineralization with digestion; elution was conducted twice with 25 µl (50 µl total elution volume) of DNAse/RNAse free water instead of EBT.

Library Preparation Methodologies

Illumina Library Preparation Method A – UDG Double-stranded library preparation

Double-stranded Illumina sequencing libraries were prepared according to Meyer and Kircher [3] with the MinElute PCR purification and reduced adapter concentration suggested in Kircher et al. [4]. Uracils were removed with the addition of Uracil-DNA glycosylase and Endonuclease 8. Final libraries were purified over MinElute PCR Purification Kit spin columns according to the manufacturer's protocol and eluted twice with 20 µl and 30 µl of TEB. Final concentrations for each step and cycling conditions can be found in Tables A5-A11.

Libraries were constructed from 25 µl of DNA extract. For samples were demineralization and digestion rounds were extracted separately, one of the two extracts was converted into a library depending on which resulted in a higher copy number for a 12S proboscidean qPCR screen.

10 µl of each library was indexed using unique P5 and P7 barcodes in a 35 µl reaction with 150 nM of each primer. Final concentrations of each reagent in the indexing reaction and cycling conditions are

presented in Tables A12 and A13. Indexed libraries were purified over MinElute according to the manufacturer's protocol with an additional PE Buffer wash, and eluted in 20 µl TEB.

Supplementary Table 6. Final concentrations of all components in the Blunt-End Repair mixture of library preparation. A final volume of 50 µl was used for the mixture and template DNA. Water (not listed) was used to bring the volume of the reaction up to a desired amount. The concentration of T4 DNA polymerase is also listed; however, it was added only after the initial 3 hour incubation at 37ºC is finished. In Method C, the T4 DNA Polymerase concentration is increased to 0.2 U/µl.

**Blunt-End Repair Master Mix**	
**Component**	**Final Concentration**
NE Buffer 2	1 x
BSA	0.1 mg/ml
dNTP Mix	100 µM
ATP	1 mM
T4 Polynucleotide Kinase	0.4 U/µl
Uracil-DNA glycosylase	0.1 U/µl
Endonuclease VIII	0.4 U/µl
T4 DNA Polymerase	0.115 U/µl

Supplementary Table 7. Cycling conditions for Blunt End Repair. Tubes were held at 4°C until ready for the following steps. Samples were removed from the thermocycler to add T4 DNA Polymerase, then put in for the remainder of the cycling program.

**Blunt-End Repair Cycling Conditions**	
**Temperature (°C)**	**Time**
37	3 hours
4	Hold
After adding T4 DNA Polymerase	
25	15 minutes
12	15 minutes
4	Hold

Supplementary Table 8. Preparation of the Oligo Hybridization Buffer and Adapter Mix. The Adapter Mix was prepared separately for IS1_adapter_P5.F and IS2_adapter_P7.F, and combined after an incubation at 95°C for 10 seconds, and a ramp from 95°C to 12°C at a rate of 0.1°C /sec.

**Oligo Hybridization Buffer**	
**Component**	**Final Concentration**
NaCl	500mM
Tris-Cl, pH 8.0	10mM
EDTA, pH 8.0	1mM
**Adapter Mix**	
IS1_adapter_P5.F or IS2_adapter_P7.F	200µM
IS3_adapter_P5+P7.R	200µM
Oligo Hybridization Buffer	1X

Supplementary Table 9. Final concentrations of all components in the Adapter Ligation mixture of library preparation. A final volume of 40 µl was used for the mixture and template DNA. Water (not listed) was used to bring the volume of the reaction up to a desired amount. In Method B the final concentration of adapter mix was increased to 0.5 µM and water decreased accordingly.

Adapter Ligation Master Mix	
**Component**	**Final Concentration**
T4 DNA Ligase Buffer	1 x
PEG-4000	5 %
Adapters	0.25 µM
T4 DNA Ligase	0.125 U/µl

Supplementary Table 10. Cycling conditions for Adapter Ligation. Tubes were held at 4°C until ready for the following steps.

Adapter Ligation Cycling Conditions	
**Temperature (°C)**	**Time**
16	14 hours
4	Hold

Supplementary Table 11. Final concentrations of all components in the Adapter Fill-In mixture of library preparation. A final volume of 50 µl was used for the mixture and template DNA. Water (not listed) was used to bring the volume of the reaction up to a desired amount.

Adapter Fill-In Master Mix	
**Component**	**Final Concentration**
ThermoPol Rxn Buffer	1 x
dNTPs	0.25 mM
BST Polymerase	0.4 U/µl

Supplementary Table 12. Cycling conditions for Adapter Fill-In. Tubes were held at 4°C until ready for MinElute purification in the absence of heat deactivation.

Adapter Fill-In Cycling Conditions	
**Temperature (°C)**	**Time**
37	30 minutes
4	Hold

Supplementary Table 13. Primer sequences and PCR master mix used during indexing amplification. The N in each primer sequence represents the unique 7bp long index, specific to each primer. A final reaction volume of 35 µl was used for the assay, with 10 µl of the purified DNA libraries. Water (not listed) was used to bring the volume of the reaction up to a desired amount. In Method B, the primer concentration is decreased to 100 nM and input increased to 20 µl in 45 µl reactions.

Primers	
Forward Primer Sequence	AATGATACGGCGACCACCGAGATCTACAC*NNNNNNN*ACACTCTTTCCCTACACGACGCTCTT
Reverse Primer Sequence	CAAGCAGAAGACGGCATACGAGATTAT*NNNNNNN*ACTGGAGTTCAGACGTGT

Mastermix	
**Component**	**Final Concentration**
Herculase II Reaction Buffer	1 x
dNTP mix	250 µM
EvaGreen Dye	0.5 X
Herculase II Fusion DNA Polymerase	0.025 U/µl
Primers	150 nM (each)

Supplementary Table 14. The PCR cycling protocol used during indexing amplification. The bolded section of the PCR protocol below was repeated for 10 cycles. Fluorescence readings were recorded at the conclusion of the central annealing step, indicated here with two asterisks.

Indexing PCR Protocol	
**Temperature (°C)**	**Time**
95	2 minutes
**95**	**15 seconds**
60	***30 seconds***
**68**	**1 minute**
68	2 minutes
8	30 seconds

Illumina Library Preparation Method B – UDG Double-stranded library preparation

Double-stranded Illumina sequencing libraries were constructed as outlined in Illumina Library Preparation Method A with the following modifications: adapter concentration increased to 0.5 µM (Supplementary Table 9); libraries were heat-deactivated for 20 minutes at 80°C instead of a final MinElute purification (Supplementary Table 15); indexing primer concentration was decreased to 100 nM each primer (Supplementary Table 13). Indexing was carried out with 20 µl of heat-deactivated library in 45 µl indexing reactions using a slightly modified PCR protocol (Supplementary Table 16). Indexed libraries were purified over MinElute according to the manufacturer’s protocol with an additional PE Buffer wash, and eluted in 15 µl TEB or 13 µl EBT.

Supplementary Table 15. Cycling conditions for Adapter Fill-In. Includes a heat-deactivation step instead of a final purification post library construction.

Adapter Fill-In Cycling Conditions	
**Temperature (°C)**	**Time**
37	30 minutes
80	20 minutes
4	Hold

Supplementary Table 16. The PCR cycling protocol used during indexing amplification of the double-stranded libraries used for the proboscidean enrichment. The bolded section of the PCR protocol below was repeated for 10 cycles. Fluorescence readings were recorded at the conclusion of the central annealing step, indicated here with two asterisks.

**Indexing PCR Protocol**	
**Temperature (°C)**	**Time**
98	3 minutes
**98**	**20 seconds**
60	***30 seconds***
**72**	**25 seconds**
72	3 minutes
8	30 seconds

Illumina Library Preparation Method C – UDG Double-stranded library preparation

Double-stranded Illumina sequencing libraries were constructed as outlined in Illumina Library Preparation Method A with the following modifications: T4 DNA polymerase final concentration increased to 0.2 U/µl (Supplementary Table 6); a final volume of 40 µl was used during adapter fill-in; libraries were heat deactived for 20 minutes at 80°C instead of a final MinElute purification (Supplementary Table 15). Indexing was carried out using 10 µl of the heat-deactivated libraries as template in 35 µl of library as specified in Tables A12 and A13. Indexed libraries were purified over MinElute according to the manufacturer's protocol with an additional PE Buffer wash, and eluted in 13 µl TEB.

Illumina Library Preparation Method D – Non-UDG Double-stranded library preparation

Double-stranded libraries were prepared as outlined in Illumina Library Preparation Method A, with a modified End-Repair reaction to account for the lack of uracil excision and the replacement of NE Buffer 2 with NE Buffer 2.1 (Supplementary Table 17). Final volumes for all reaction steps were changed to 40 µl. Libraries were heat deactivated for 20 minutes at 80°C instead of a final MinElute purification (Supplementary Table 15). 12.5 µl of heat-deactivated library was used in 40 µl indexing reactions as specified in Tables A17 and A18. Indexed libraries were purified over MinElute according to the manufacturer's protocol with an additional PE Buffer wash, and eluted in 13 µl EBT.

Supplementary Table 17. Final concentrations of all components in the Blunt-End Repair mixture of library preparation in Method D. A final volume of 40 µl was used for the mixture and template DNA. Water (not listed) was used to bring the volume of the reaction up to a desired amount.

**Blunt-End Repair Master Mix**	
**Component**	**Final Concentration**
NE Buffer 2.1	1 x
DTT	1 mM
dNTP Mix	100 µM
ATP	1 mM
T4 Polynucleotide Kinase	0.5 U/µl
T4 DNA Polymerase	0.1 U/µl

Supplementary Table 18. Primer sequences and PCR master mix used during indexing amplification. The N in each primer sequence represents the unique 7bp long index, specific to each primer. A final reaction volume of 40 µl was used for the assay, with 12.5 µl of the purified DNA libraries. Water (not listed) was used to bring the volume of the reaction up to a desired amount.

Primers	
Forward Primer Sequence	AATGATACGGCGACCACCGAGATCTACAC*NNNNNNN*ACACTCTTTCCCTACACGACGCTCTT
Reverse Primer Sequence	CAAGCAGAAGACGGCATACGAGATTAT*NNNNNNN*ACTGGAGTTCAGACGTGT

Mastermix	
**Component**	**Final Concentration**
KAPA SYBR®FAST qPCR Master Mix (2X)	1 x
Primers	750 nM (each)

Supplementary Table 19. The PCR cycling protocol used during indexing amplification of Method D libraries. The bolded section of the PCR protocol below was repeated for 10 cycles. Fluorescence readings were recorded at the conclusion of the annealing/extension step, indicated here with two asterisks.

Indexing PCR Protocol	
**Temperature (°C)**	**Time**
95	5 minutes
**95**	**30 seconds**
60	***45 seconds***
60	3 minutes

Illumina Library Preparation Method E – Non-UDG Single-stranded library preparation

Non-UDG single-stranded libraries were created using the ssDNA 2.0 protocol outlined in Gansauge & Meyer[5] with the modifications from Gansauge et al.[6]. Libraries were constructed using 5 µl of extract as input. 12.5 µl of the final library was indexed using unique P5 and P7 barcodes in 40 µl indexing reactions (Supplementary Table 18 and 19). Indexed libraries were purified over MinElute according to the manufacturer's protocol with an additional PE Buffer wash, and eluted in 13 µl EBT.

Libraries appended with a "P" (e.g., LFR8-1P) were indexed as above, but pooled over a single MinElute column using the same purification protocol as above. Pools consisted of all P-labelled indexed libraries from a single specimen.

Enrichment Methodologies

In-solution Enrichment Method A

Enrichment was performed as per the round 1 enrichment strategy outlines in Karpinski et al. [7]. All enrichments in each method used the comprehensive proboscidean bait set described in Enk et al. [8].

Hybridization and capture mixes were prepared to the concentrations given in Supplementary Table 20. 10 µl of each library was combined with 1.65 µl of blocking oligos to final concentration of 11.4 µM of each of the four forward blocks ("Bloligos") (Supplementary Table 21). 13 µl of the hybridization mix and 1.35µl of the capture mix were combined and pre-warmed to 50°C, and the entire 14.5 µl volume was added to the library-Bloligo mixture. Capture was performed at 48°C for 38 hours.

20 µl of beads were washed three times using 80 µl binding buffer per wash, before being resuspended in 200 µl binding buffer. Enriched libraries were transferred to the 200 µl bead suspension to allow bait-bead binding for 45 minutes at room temperature with rotation. Beads were then washed with wash buffer 1 and 2 according to the manufacturer's protocol (MYbaits manual v1, Arbor Biosciences), except with 200 µl each and at 48°C for wash buffer 2. Libraries were eluted according to the manufacturer's protocol with an additional purification step over MinElute prior to reamplification. Libraries were reamplified in 30 µl reactions (Supplementary Table 22) using the cycling protocol in Supplementary Table 23.

Supplementary Table 20. Final concentrations of all components in the hybridization and capture mixtures for mitochondrial enrichment. 13.00µl of the hybridization mixture and 1.35µl of the capture mixture were used per enrichment reaction.

Hybridization Mixture	
**Component**	**Final Concentration**
Hyb #1 (20X SSPE)	10.9 x
Hyb #2 (0.5M EDTA)	0.01 M
Hyb #3 (50X Denhardt's)	10.9 x
Hyb #4 (1% SDS)	0.22 %
**Capture Mixture**	
**Component**	**Final Concentration**
Baits	13.33 ng/µl
SUPERase-IN	3.33 U/µl

Supplementary Table 21. Final concentration of the blocking oligonucleotides (Bloligos) in the library-blocker mixture. Bloligos were combined with 10 µl of library per reaction.

Blocker Concentrations	
**Component**	**Final Concentration**
Bloligos (200 µM each)	11.40 µM

Supplementary Table 22. Primer sequences and PCR master mix used during reamplification. A final reaction volume of 30µl was used, including 13µl of the adapter-ligated DNA libraries. Water (not listed) was used to bring the volume of the reaction up to a desired amount.

**Primers**	
Forward Primer Sequence	AATGATACGGCGACCACCGA
Reverse Primer Sequence	CAAGCAGAAGACGGCATACGA
**Mastermix**	
**Component**	**Final Concentration**
Herculase II Reaction Buffer	1 x
dNTP mix	250 µM
EvaGreen Dye	0.5 X
Herculase II Fusion DNA Polymerase	0.025 U/µl
Primers	75nM (each)

Supplementary Table 23. The PCR cycling protocol used during reamplication of the In-solution Enrichment Method A and B libraries. The bolded section of the PCR protocol below was repeated for 15 cycles. Fluorescence readings were recorded at the conclusion of the annealing/extension step, indicated here with two asterisks.

**Indexing PCR Protocol**	
**Temperature (°C)**	**Time**
98	3 minutes
**98**	**20 seconds**
60	***30 seconds***
**72**	**35 seconds**
72	3 minutes
8	30 seconds

In-solution Enrichment Method B

Enrichments were performed as outlined in In-solution Enrichment Method A with the following modifications: Block #1 (Human Cot1 DNA) and Block #2 (Salmon Sperm DNA) (Supplementary Table 24) were added to a final concentration of 0.28 µg/µl each; library volume was decreased to 6.17 µl to accommodate increased block volume; beads were resuspended in 20 µl EBT after washing with Wash Buffer 2; beads were not eluted using NaOH, but instead 10 µl of the suspension was used as template in the reamplification step with no additional purification.

Supplementary Table 24. Final concentration of the blocking oligonucleotides (Bloligos) and Blocks #1 and #2 in the library-block mixture. Library input was decreased to 6.17 µl to accommodate increased block volume.

**Blocker Concentrations**	
**Component**	**Final Concentration**
Bloligos (200 µM each)	11.40 µM
Block #1 (Human Cot1 DNA)	0.28 µg/µl
Block #2 (Salmon Sperm DNA)	0.28 µg/µl

In-solution Enrichment Method C

Enrichment was performed as per the two round Xenarthran enrichment strategy outlined in Karpinski et al.[7].

Enrichments were carried out using 5 µl of indexed library according to the MYbaits manual v3 with the following optimizations: hybridization temperature increased to 55°C and hybridization time decreased to 24 hours; hybridization (Supplementary Table 25) and block master mixes (Supplementary Table 26) modified; final library suspension was in 18.8 µl of EBT, the entire volume of which was used for reamplification using the final PCR master mix concentrations and cycling conditions as for indexing (Supplementary Table 27 and 28), but for 12 cycles; amplified enriched libraries were eluted in 10.75µl of EBT following the first round (entire volume into second round of enrichment), and 15µl of EBT following the second round.

Supplementary Table 25. Final concentrations of all components in the hybridizataion master mix for the In-solution Enrichment Method C and D libraries. A final volume of 14.50µl was used.

Hybridization Mixture	
**Component**	**Final Concentration**
Hyb #1 (20X SSPE)	11.35 x
Hyb #2 (0.5M EDTA)	0.01 M
Hyb #3 (50X Denhardt's)	11.36 x
Hyb #4 (10% SDS)	0.22 %
Baits	8.02 ng/µl
SUPERase-IN	1.61 U/µl

Supplementary Table 26. Final concentrations of the blocks in the Blocker mix. 2.55µl of the Blocker master mix was combined with either 5.75µl of water and 5µl of purified indexed library to serve as input for the first enrichment, or with the full 10.75µl of purified reamplified library following round 1 for the second round of enrichment.

Blocker Concentrations	
**Component**	**Final Concentration**
Block #1 (Human Cot1 DNA)	0.15 µg/µl
Block #2 (Salmon Sperm DNA)	0.15 µg/µl
Block #3 (Illumina bloligos)	1.44 µM

Supplementary Table 27. Primer sequences and PCR master mix used during enrichment reamplification for In-solution Enrichment Method C and D libraries. A final reaction volume of 40µl was used for the assay, with 18.8µl of the adapter ligated DNA libraries.

Primers	
Forward Primer Sequence	AATGATACGGCGACCACCGA
Reverse Primer Sequence	CAAGCAGAAGACGGCATACGA
**Mastermix**	
**Component**	**Final Concentration**
KAPA SYBR®FAST qPCR Master Mix (2X)	1 X
Primers	150 nM (each)

Supplementary Table 28. The PCR cycling protocol used enrichment reamplification of In-solution Enrichment Method C and D libraries. The core of the PCR protocol (denaturation 95ºC-30sec; annealing/extension 60ºC-45sec) shown here in bold and with a heavier border, was repeated for 12 cycles. Fluorescence readings were recorded at the conclusion of the extension step, indicated here with two asterisks.

Indexing PCR Protocol	
**Temperature (ºC)**	**Time**
95	3 min
**95**	**30 sec**
60	***45 sec***
60	3 min

In-solution Enrichment Method D

Enrichments were carried out using 5ul of indexed library as in In-solution Enrichment Method C, except with Block #3 (Illumina Bloligos) replaced with Block #3 (Single-stranded Blocks) to account for the different adapter structure in single-stranded libraries (Supplementary Table 29). Libraries underwent two rounds of enrichment with identical round 2 input and final elution volumes.

Supplementary Table 29. Final concentrations of the blocks in the Blocker mix for In-solution Enrichment Method D libraries. Double stranded illumine blocks were replaced with a modified version to accommodate the different adapter structure of single stranded libraries. 2.55µl of the Blocker master mix was combined with either 5.75µl of water and 5µl of purified indexed library to serve as input for the first enrichment, or with the full 10.75µl of purified reamplified library following round 1 for the second round of enrichment.

Blocker Concentrations	
**Component**	**Final Concentration**
Block #1 (Human Cot1 DNA)	0.15 µg/µl
Block #2 (Salmon Sperm DNA)	0.15 µg/µl
Block #3 (Single-stranded blocks)	1.44 µM

Pooling, Size Selection, and Sequencing Methodologies

Enriched libraries were pooled to contain equal quantities of fragments, and size selected through gel electrophoresis (3% Nusieve GTG Agarose Gel; 100 V for 35 minutes) for molecules in the range of 150bp to ~500-600bp. Gel plugs were purified using the QIAquick Gel Extraction Kit (Qiagen), according to the manufacturer's protocol and sent off for sequencing.

Libraries were sequenced on an Illumina HiSeq 1500 using paired end chemistry at the read lengths indicated in Supplementary Data 1. Double-stranded libraries were sequenced using the standard Illumina sequencing primers, and single-stranded libraries with the single-stranded primer described in Gansauge and Meyer [5]. Reads from sequencing runs before 2016-08-09 were demultiplexed using CASAVA and from that point forwards by bcl2fastq.

Reference Guided Mapping

Demultiplexed reads were trimmed and merged using the ancient DNA settings (--ancientdna) in leeHom [9] with either double stranded library adapter sequences (default) or by manually providing the single-stranded adapter sequences. Reads were aligned to an indexed version of the mastodon mitochondrial reference (*Mammut americanum*-NC_035800) using a network-aware version of BWA [10] (https://github.com/mpieva/network-aware-bwa) to allow for multiprocessing on a dedicated bioinformatics cluster. BWA was run with commonly accepted ancient DNA settings: maximum edit distance of 0.01 (-n 0.01), allowing for a maximum of two gap openings (-o 2), and with seeding effectively disabled (-l 16500). Reads that mapped and were either merged or properly paired were extracted using the retrieveMapped_single_and_ProperlyPair program of libbam (https://github.com/grenaud/libbam). Reads were then collapsed based on unique 5' and 3' positions (https://bitbucket.org/ustenzel/biohazard), and restricted to a minimum length of 24bp and a minimum map quality of 30.

All sequenced libraries for each sample were combined. Libraries with unique indices were combined without further duplicate read removal as they represent all unique reads. Sequences generated from the same indexed libraries went through an additional round of duplicate removal as above. Library information for each sample can be found in Supplementary Data 1.

Final consensus sequences were annotated in Geneious (v6.1.5), and prepared for GenBank submission using GB2sequin [11].

Sequence Curation

Alignments were imported into Geneious (v6.1.5), and manually curated to remove sequencing artefacts. Insertions or deletions which occurred in at least 50% of all reads were retained. Nucleotide positions with less than 3-fold coverage were masked with N's. A repetitive portion of the D-Loop was also masked as in NC_035800 as this region has proven to be difficult to resolve.

During sequence curation, samples ISM2015-58, ISM2015-54, Beusching, P14591, UAMES 7663, and FAM 103291 were observed to have disproportionately high coverage in certain regions; visible as "stacks" (Supplementary Fig. 1). These stacks primarily occurred in conserved regions of the mitochondria such as within the 16S rRNA and D-loop. BLAST analysis suggested these reads likely originate from non-endogenous sources, and map due to their relatively short fragment size and conserved nature of various mitochondrial loci. As an additional step we also masked any region with a depth of coverage greater than 3 standard deviations from the mean if that region contained at least 3 polymorphic reads carrying the same substitution.

Consensus sequences were generated using a 50% strict majority criteria. 33 specimens met our inclusion threshold of >80% coverage of the mastodon mitochondrial reference (NC_035800) at a minimum depth of 3x (Supplementary Fig. 2). Full mapping and coverage information for each sample can be found in Supplementary Table 30 (complete specimens) and Supplementary Table 31 (partial specimens).

Two additional mastodons (NC_035800 and EF632344) were included in the final dataset, as well as both Woolly Mammoth (NC_007596) and Columbian Mammoth (NC_015529) mitochondrial genomes to root the tree. All four sequences were D-loop masked as above.

Supplementary Figure 1. Coverage plot showing depth of coverage before filtering out polymorphic regions greater than 3 standard deviations from the mean (A) and after filtering is applied (B) in F:AM 103291. (C) Close up of the 16S rRNA stack showing the polymorphic nature of the reads in the "stack", with multiple positions showing support for different bases in at least 3 reads. (D) The same section of the alignment as in (C), after filtering has been applied.

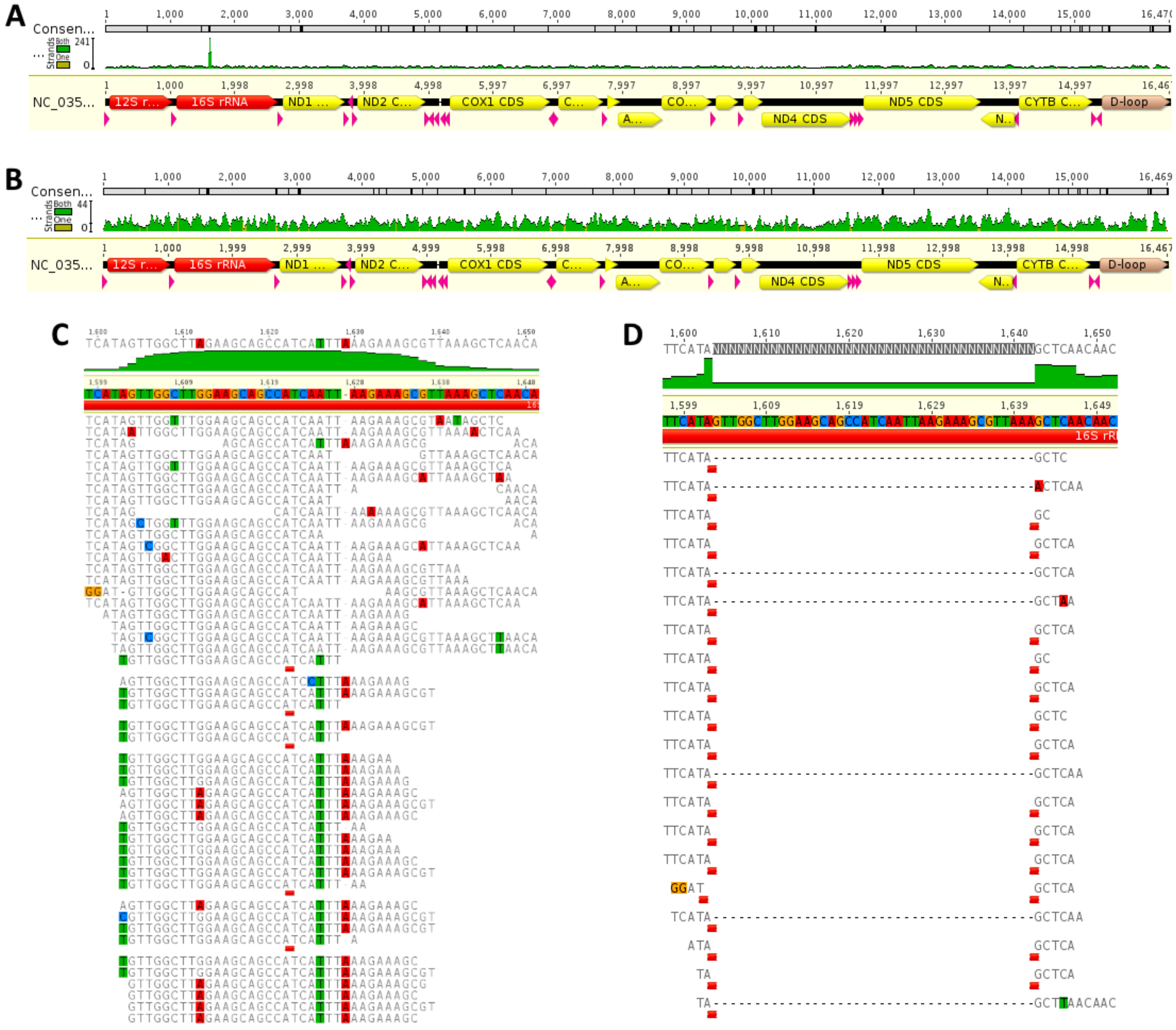

Supplementary Figure 2. Specimen locations analyzed in this study. Specimens without known latitude/longitude coordinates were approximated from listed place of origin. Large circles indicate locations of specimens for which complete mitochondrial genomes were obtained, coloured according to their clade assignment. Crosses indicate samples for which no data was obtained, and small, crossed out brown circles indicate the locations of samples for which partial mitochondrial genomes were recovered. Stars represent previously sequenced specimens.

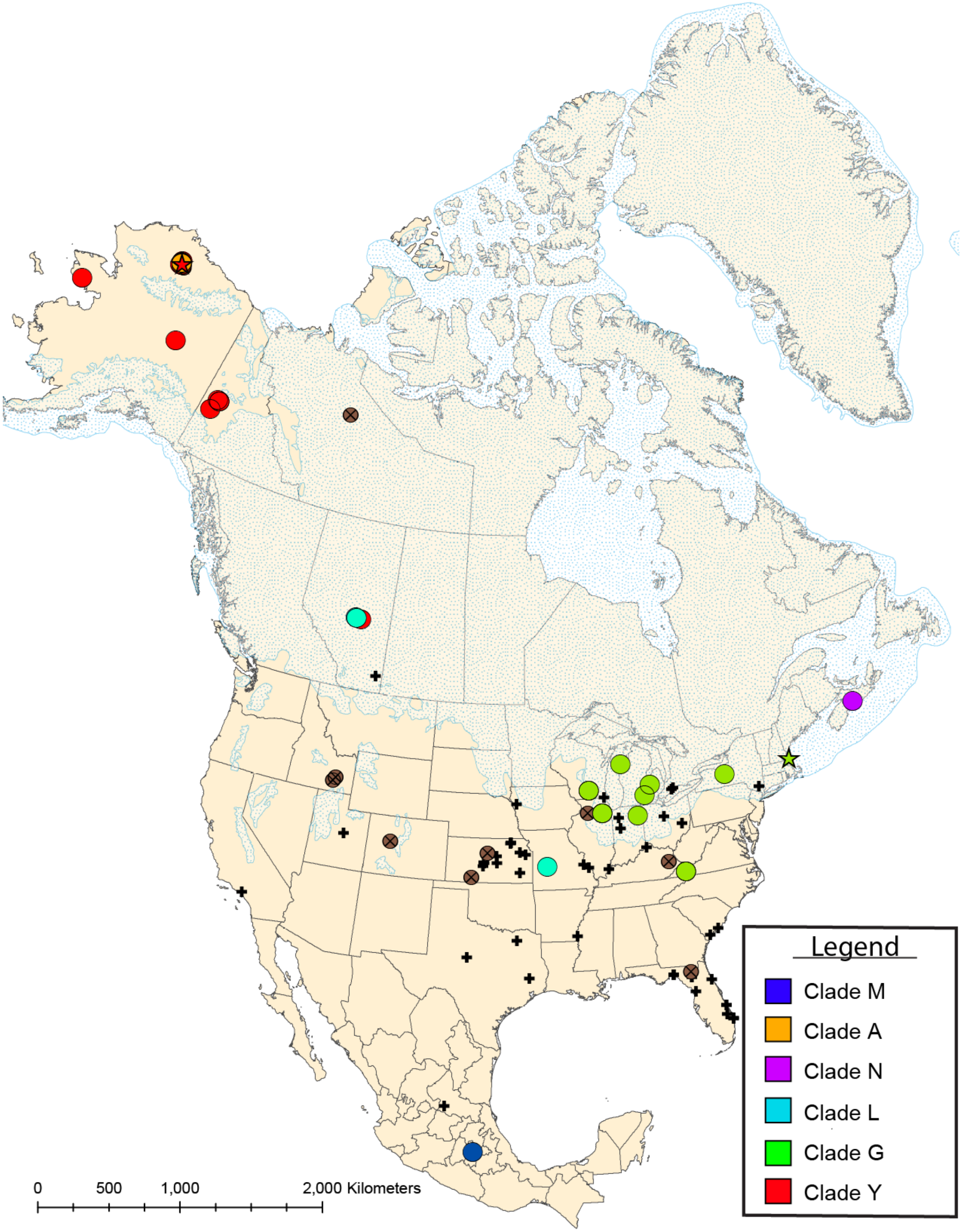

Supplementary Table 30. Mapping statistics for all complete mastodon specimens. All fields represent final values post minimum length, map quality, and coverage depth curation. Fields correspond to the number of reads mapped (Reads Mapped), percent coverage of the NC_035800 reference (% Coverage), average coverage depth of the alignment (Mean Cov. Depth), and the average fragment size of all mapped reads (Mean Frag. Size).

Specimen ID	Reads Mapped	% Coverage	Mean Cov. Depth	Mean Frag. Size
AMNH 988	3,734	97.8	9.1	40.0
Beusching	3,228	86.2	7.1	35.6
CCM-1	4,131	98.3	12.6	50.0
CMN 11697	3,064	97.4	8.8	47.0
DP1296	4,267	92.2	9.9	37.9
F:AM 103291	4,798	99.4	13.1	45.0
IK-01-277	1,983	97.7	8.0	66.5
IK-01-321	3,431	99.6	16.0	60.8
IK05-3.5	24,965	99.8	85.7	56.5
IK08-127	1,480	87.1	4.5	49.0
IK10-106	3,390	99.5	14.8	72.1
IK-98-963	2,422	99.1	9.5	64.5
IK-99-328	2,598	99.0	10.3	65.4
ISM 2015-53	4,108	98.5	13.5	53.9
ISM 2015-54	1,737	85.3	5.4	49.7
ISM 2015-58	1,853	84.3	4.9	42.2
KIG 12-15	26,846	99.8	106.9	65.6
MAY 12-69	1,746	98.7	8.1	76.0
MAY 12-70	10,489	99.6	32.6	51.2
P12780	10,997	99.7	35.0	52.4
P14591	2,143	88.5	5.8	43.6
RAM P94.16.1B	2,172	91.7	6.4	47.9
RAM P94.5.7	2,684	95.4	6.8	41.2
RAM P97.7.1	4,781	96.5	12.6	43.4
ETMNH 19334	51,113	99.4	142.1	45.8
ETMNH 19335	3,129	91.0	7.7	40.0
UAMES 7663	2,481	96.6	8.5	56.1
UM 13909	3,657	98.3	11.2	50.3
UM 57705	4,818	98.1	12.5	42.7
UM 58075	4,630	99.0	14.9	52.8
YG 26.1	6,495	99.7	23.0	58.3
YG 43.2	5,430	99.6	17.0	51.7
YG 50.1	1,678	88.5	4.8	45.6

Supplementary Table 31. Mapping statistics for all partial mastodon specimens. All fields represent final values post minimum length, map quality, but without any coverage depth filtering. Fields correspond to the number of reads mapped (Reads Mapped), percent coverage of the NC_035800 reference (% Coverage), average coverage depth of the alignment (Mean Cov. Depth), and the average fragment size of all mapped reads (Mean Frag. Size).

Specimen ID	Reads Mapped	% Coverage	Mean Cov. Depth	Mean Frag. Size
50003/16643	515	64.8	1.3	40.3
52002/25939	580	53.4	1.4	38.7
66.555	246	34.3	0.5	36.0
AMNH 10666	267	32.2	0.6	39.0
AMNH 22728	46	9.7	0.1	40.2
F:AM 105129	218	17.1	0.5	33.4
L2062.2	161	22.3	0.6	56.6
MAY 12-45	420	77.1	1.6	63.0
RAM P89.13.538	75	11.0	0.2	47.3
ETMNH 6495	128	20.8	0.3	36.0
UKMNH 2859	286	39.1	0.8	44.3
UKMNH 5898	122	23.0	0.3	42.7

Sequence Authenticity and Map Damage

To verify the authenticity of our data we examined cytosine deamination patterns using mapDamage 2.0 [12]. Specimens with both UDG-treated and non-UDG treated libraries, were reprocessed combining all non-UDG libraries to mapDamage analysis, as UDG/Endonuclease VIII treatment removes the cytosine deamination signal.

Specimens with non-UDG treated libraries showed an increase in 5’ C → T and 3’ G → A (double-stranded) or 3’ C → T (single-stranded) substitutions rates as expected for authentic ancient DNA constructed under the two library preparation methodologies (Supplementary Fig. 3). Specimens with libraries that were generated using only UDG-treated methods displayed no evidence of increased substitution rates at either end as a result of the UDG/Endonuclease VIII. Additionally, mapped reads for all specimens were very short (mean 51.54 bp), expected of ancient endogenous DNA.

Extraction blanks contained very few reads that mapped at the above thresholds (minimum fragment size 24 bp; mapping quality of 30) with of <= 1 mapped reads per library (n=10 carried through with all processed sample sets), suggesting minimal evidence of exogenous or cross-contamination.

Supplementary Figure 3. MapDamage plots for all complete American mastodon specimens. Red lines show the frequency of C → T substitutions and blue lines the frequency of G → A substitutions on the 5’ (left graph) and 3’ (right graph) ends of the molecules. Specimens whose libraries were treated with UDG/Endonuclease VIII display no uptick in terminal substitutions as this treatment removes this molecular basis for this signal. Non-UDG double stranded libraries see an uptick in 5’ C → T and 3’ G → A substitutions, whereas non-UDG single-stranded libraries contain an increase in C → T substitutions on both ends. Specimens are grouped such that AMNH 988 through to YG 50.1 contain only UDG-treated libraries, FAM 103291 through to RAM P94.5.7 contain a mix of UDG-treated and non-treated libraries, and DP1296 through to ISM 2015-58 contain only non-treated libraries.

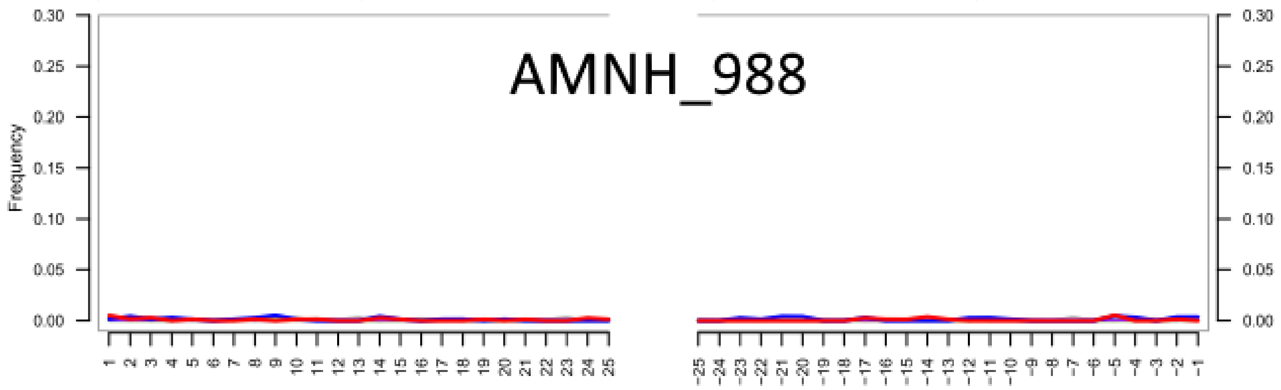

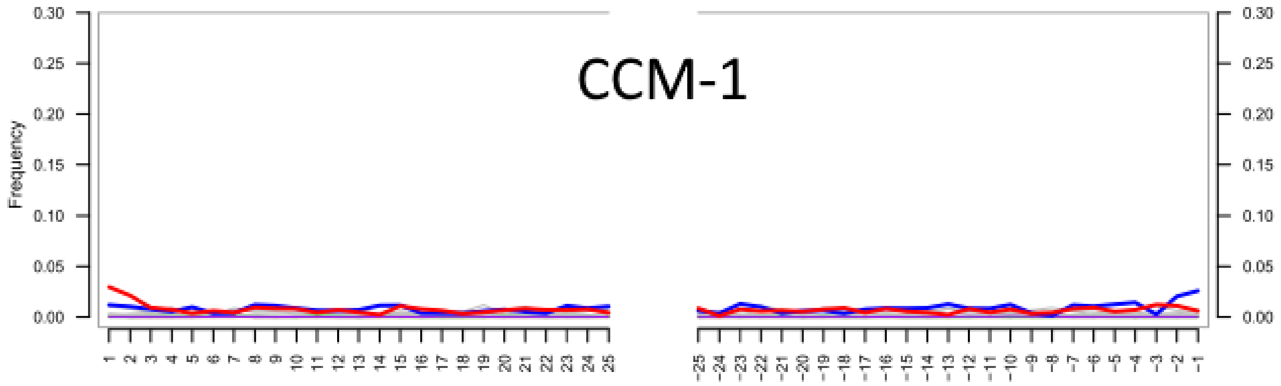

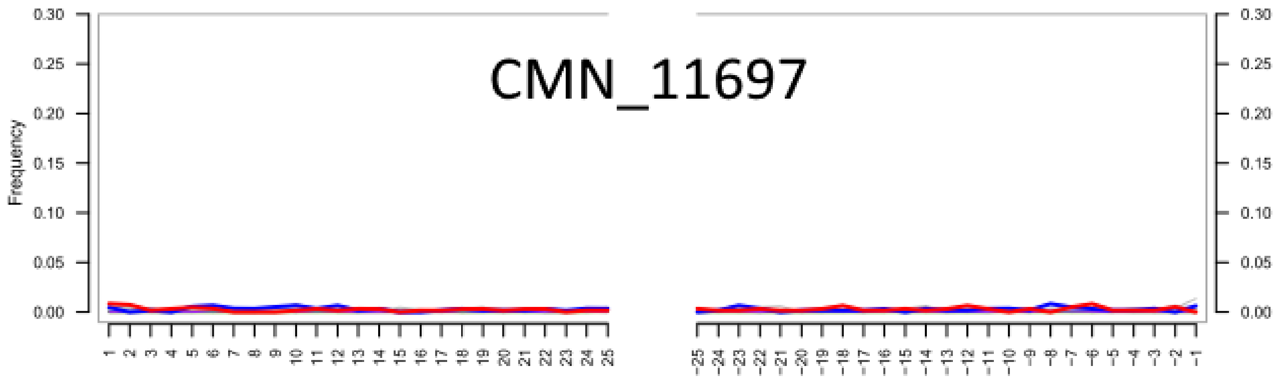

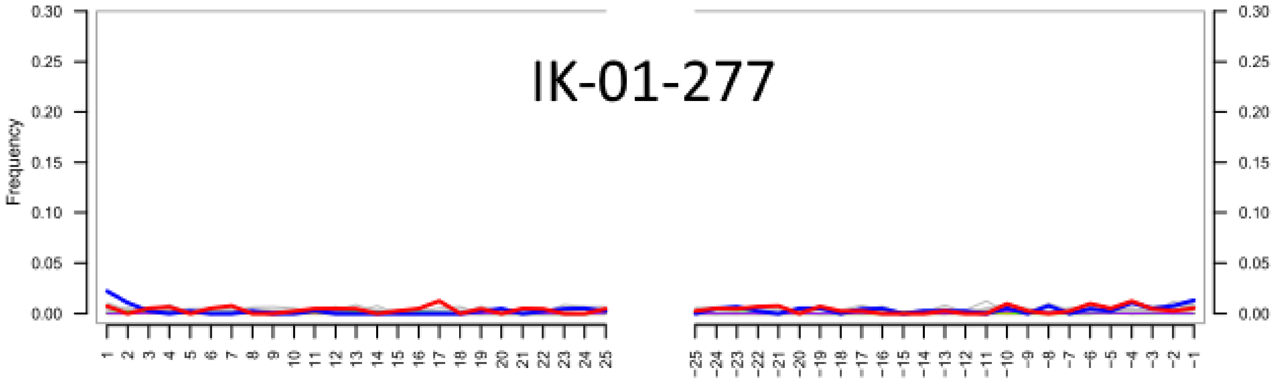
IK-01-277
Frequency
0.00 0.05 0.10 0.15 0.20 0.25 0.30
1 2 3 4 5 6 7 8 9 10 11 12 13 14 15 16 17 18 19 20 21 22 23 24 25
-25 -24 -23 -22 -21 -20 -19 -18 -17 -16 -15 -14 -13 -12 -11 -10 -9 -8 -7 -6 -5 -4 -3 -2 -1

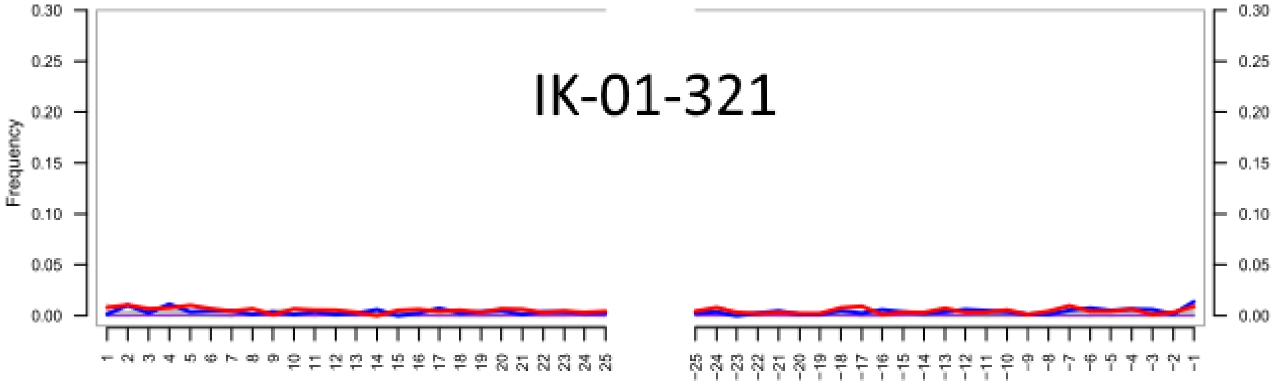
IK-01-321
Frequency
0.00 0.05 0.10 0.15 0.20 0.25 0.30
1 2 3 4 5 6 7 8 9 10 11 12 13 14 15 16 17 18 19 20 21 22 23 24 25
-25 -24 -23 -22 -21 -20 -19 -18 -17 -16 -15 -14 -13 -12 -11 -10 -9 -8 -7 -6 -5 -4 -3 -2 -1

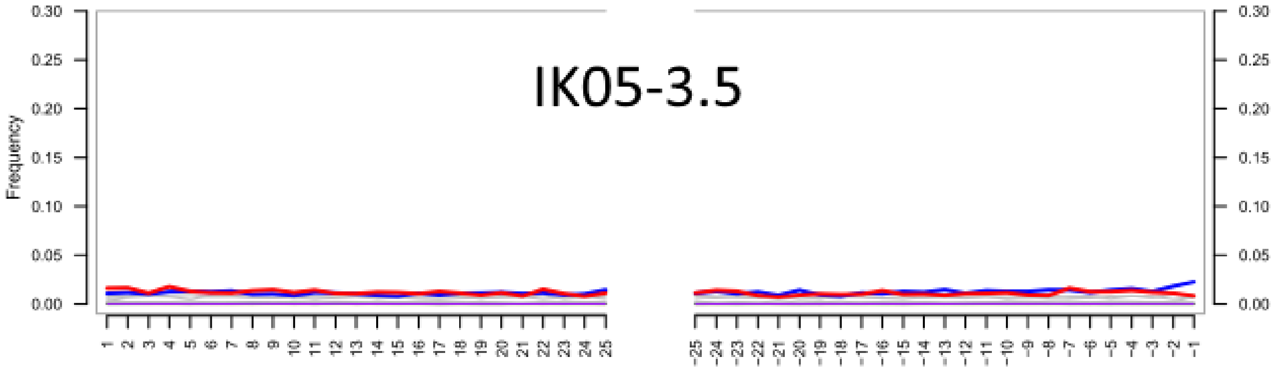
IK05-3.5
Frequency
0.00 0.05 0.10 0.15 0.20 0.25 0.30
1 2 3 4 5 6 7 8 9 10 11 12 13 14 15 16 17 18 19 20 21 22 23 24 25
-25 -24 -23 -22 -21 -20 -19 -18 -17 -16 -15 -14 -13 -12 -11 -10 -9 -8 -7 -6 -5 -4 -3 -2 -1

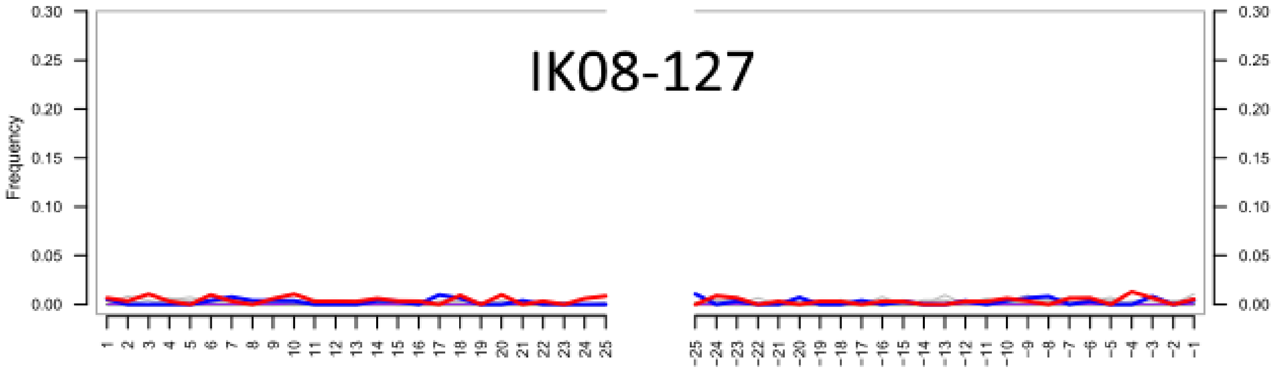
IK08-127
Frequency
0.00 0.05 0.10 0.15 0.20 0.25 0.30
1 2 3 4 5 6 7 8 9 10 11 12 13 14 15 16 17 18 19 20 21 22 23 24 25
-25 -24 -23 -22 -21 -20 -19 -18 -17 -16 -15 -14 -13 -12 -11 -10 -9 -8 -7 -6 -5 -4 -3 -2 -1

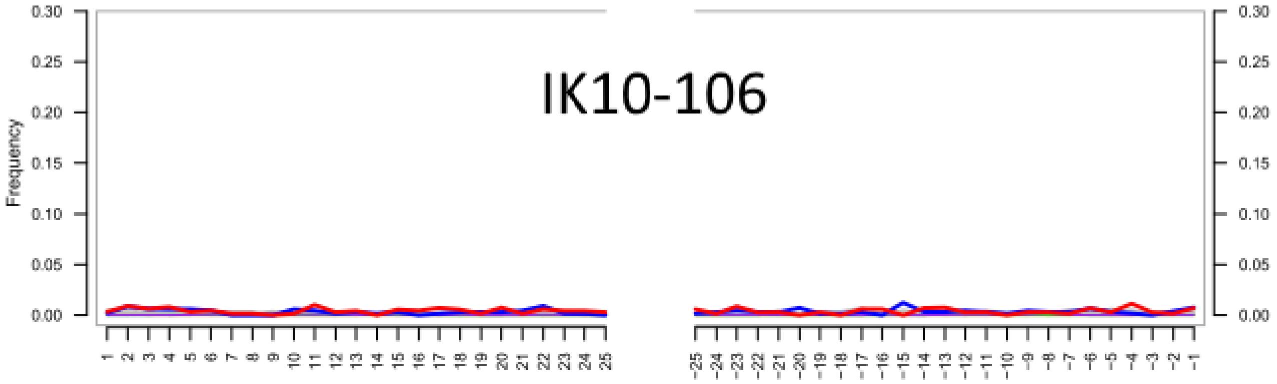
IK10-106
Frequency

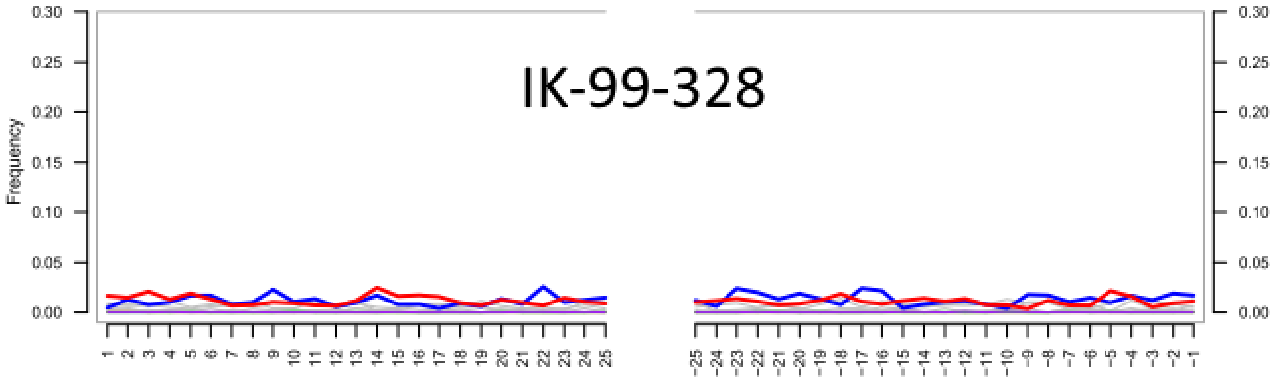
IK-99-328
Frequency

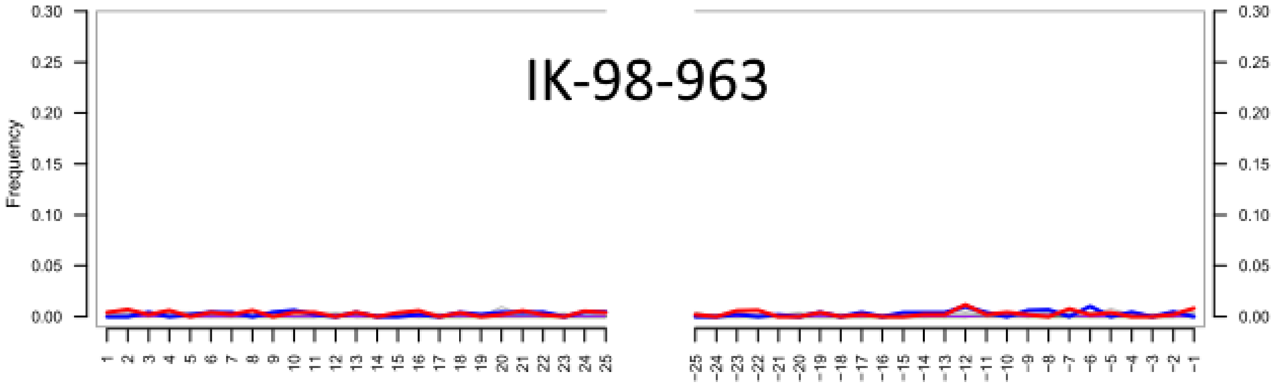
IK-98-963
Frequency

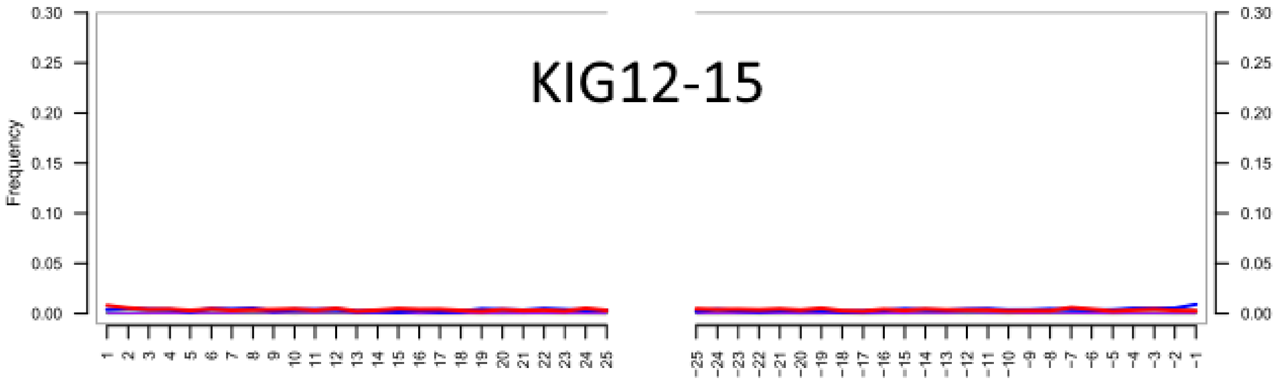
KIG12-15
Frequency

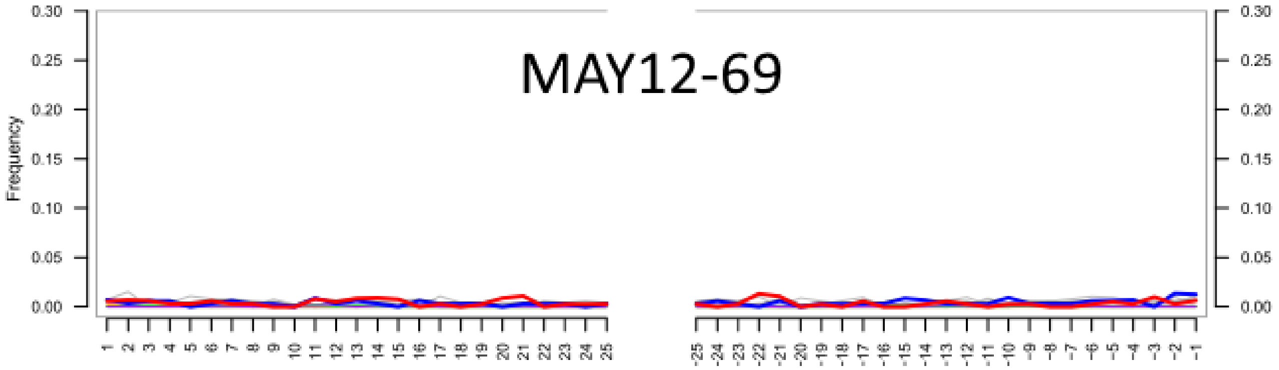
MAY12-69
Frequency
0.30
0.25
0.20
0.15
0.10
0.05
0.00

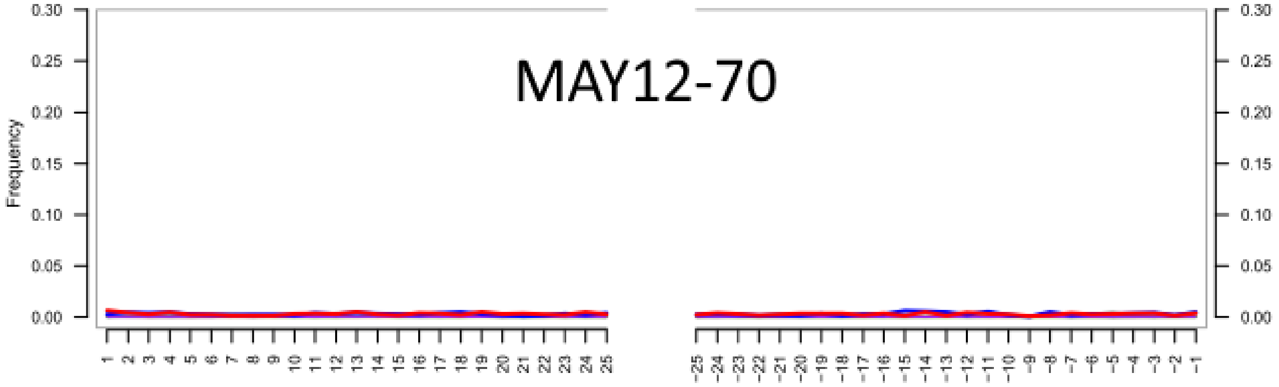
MAY12-70
Frequency
0.30
0.25
0.20
0.15
0.10
0.05
0.00

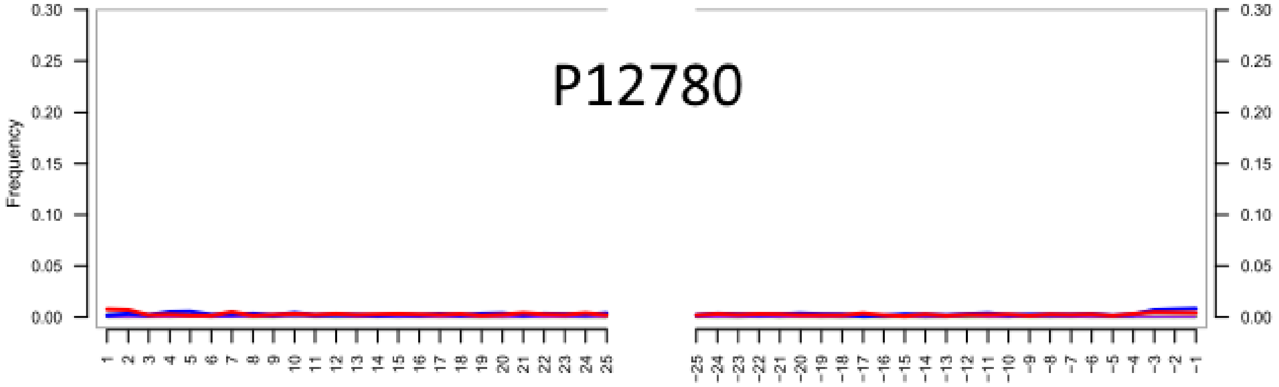
P12780
Frequency
0.30
0.25
0.20
0.15
0.10
0.05
0.00

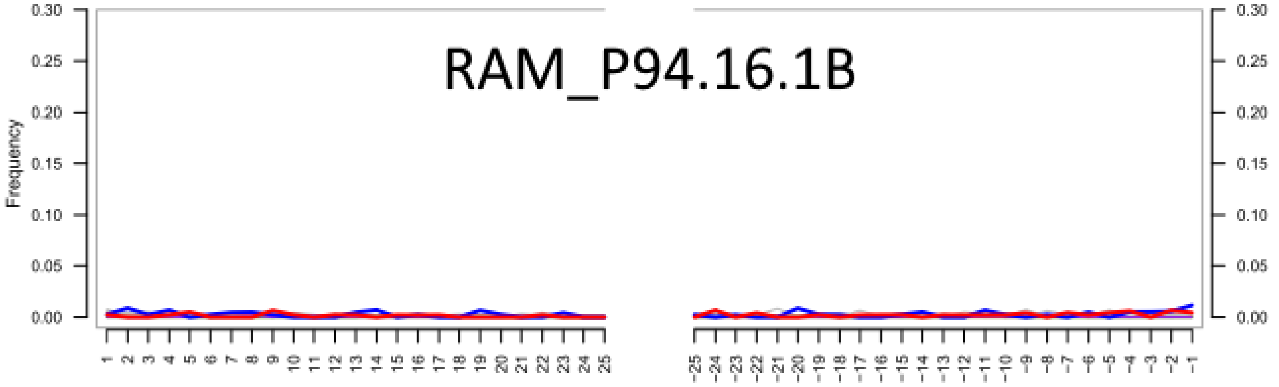
RAM_P94.16.1B
Frequency
0.30
0.25
0.20
0.15
0.10
0.05
0.00

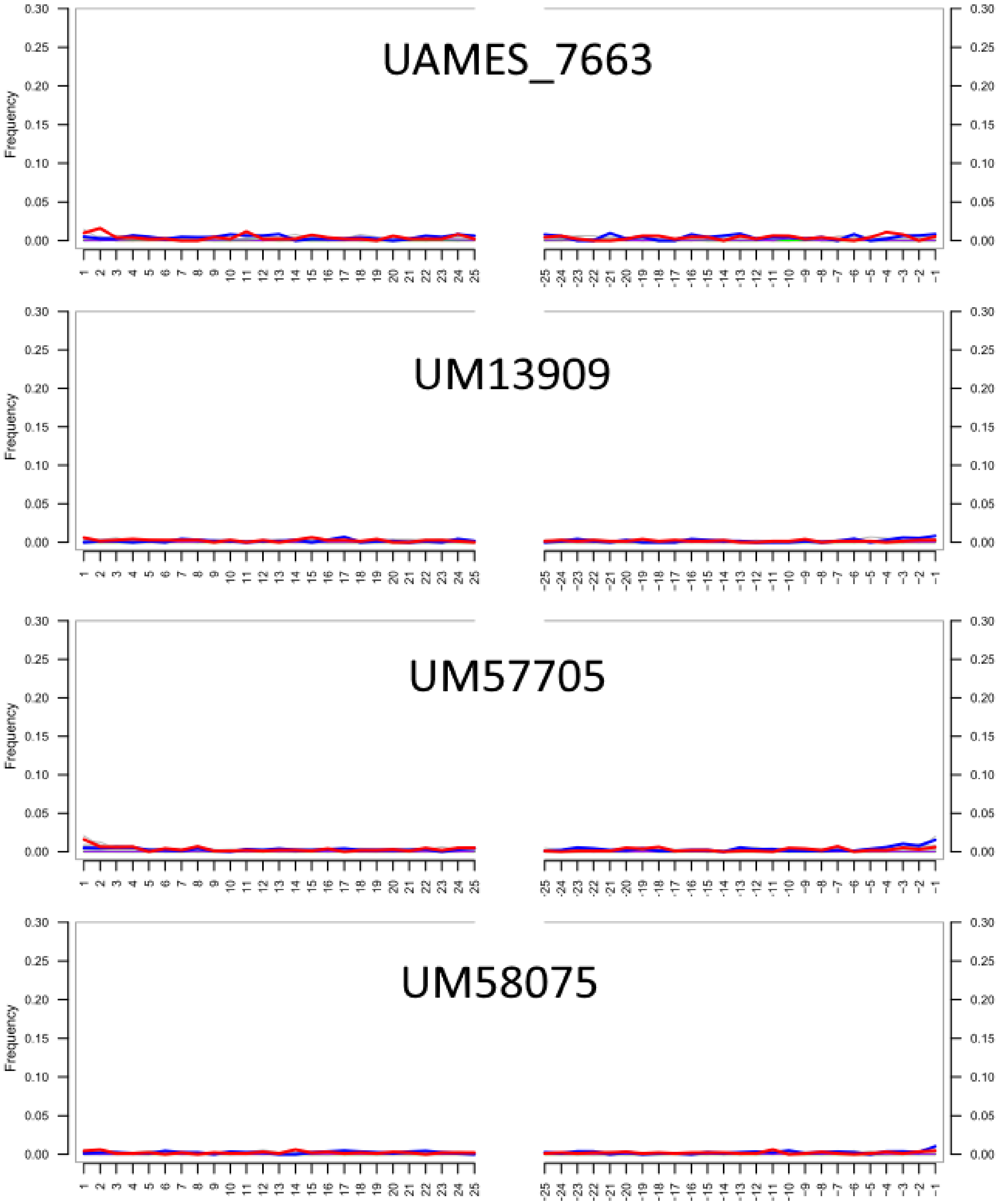
UAMES_7663
UM13909
UM57705
UM58075
Frequency
0.30
0.25
0.20
0.15
0.10
0.05
0.00

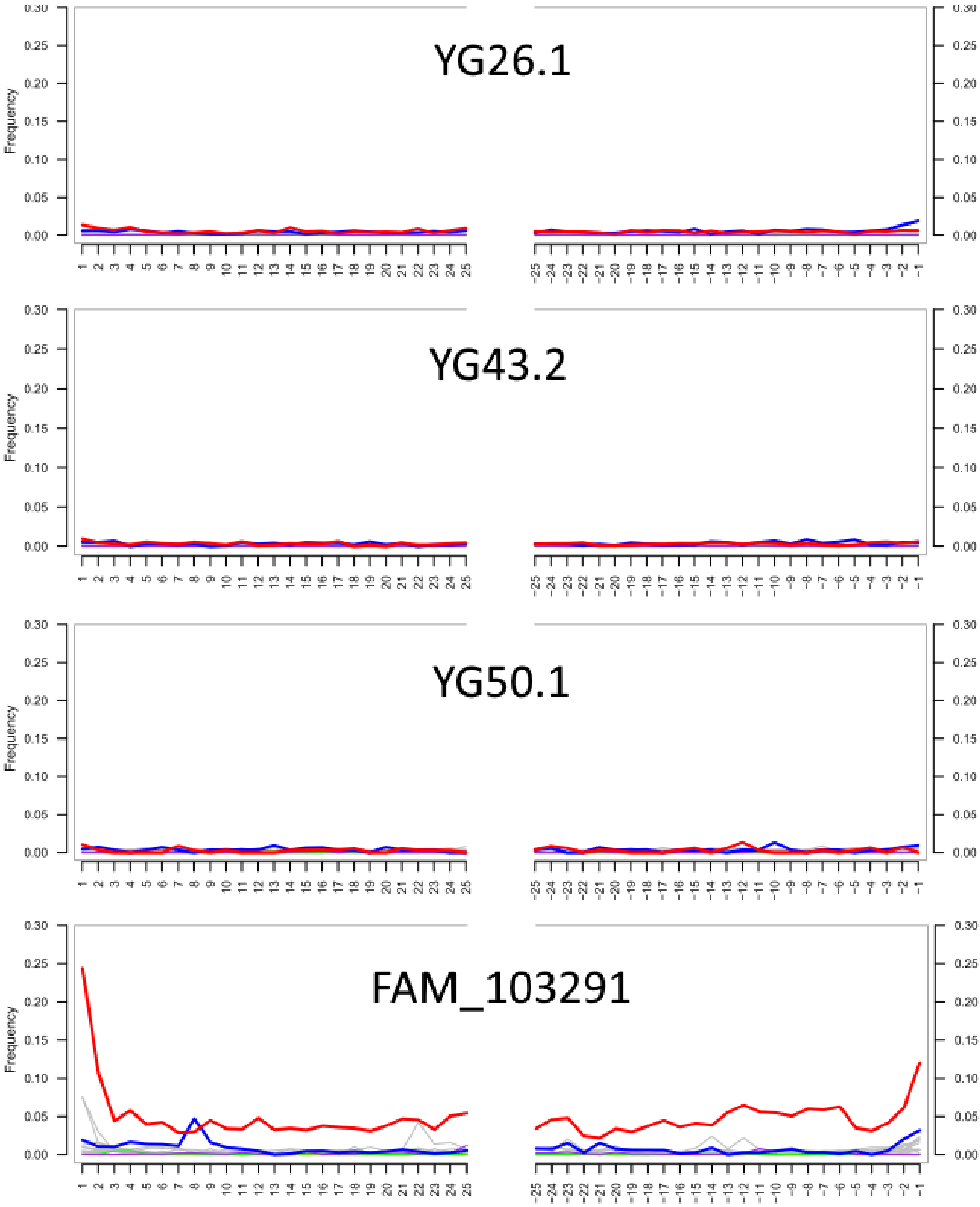
YG26.1
YG43.2
YG50.1
FAM_103291
Frequency

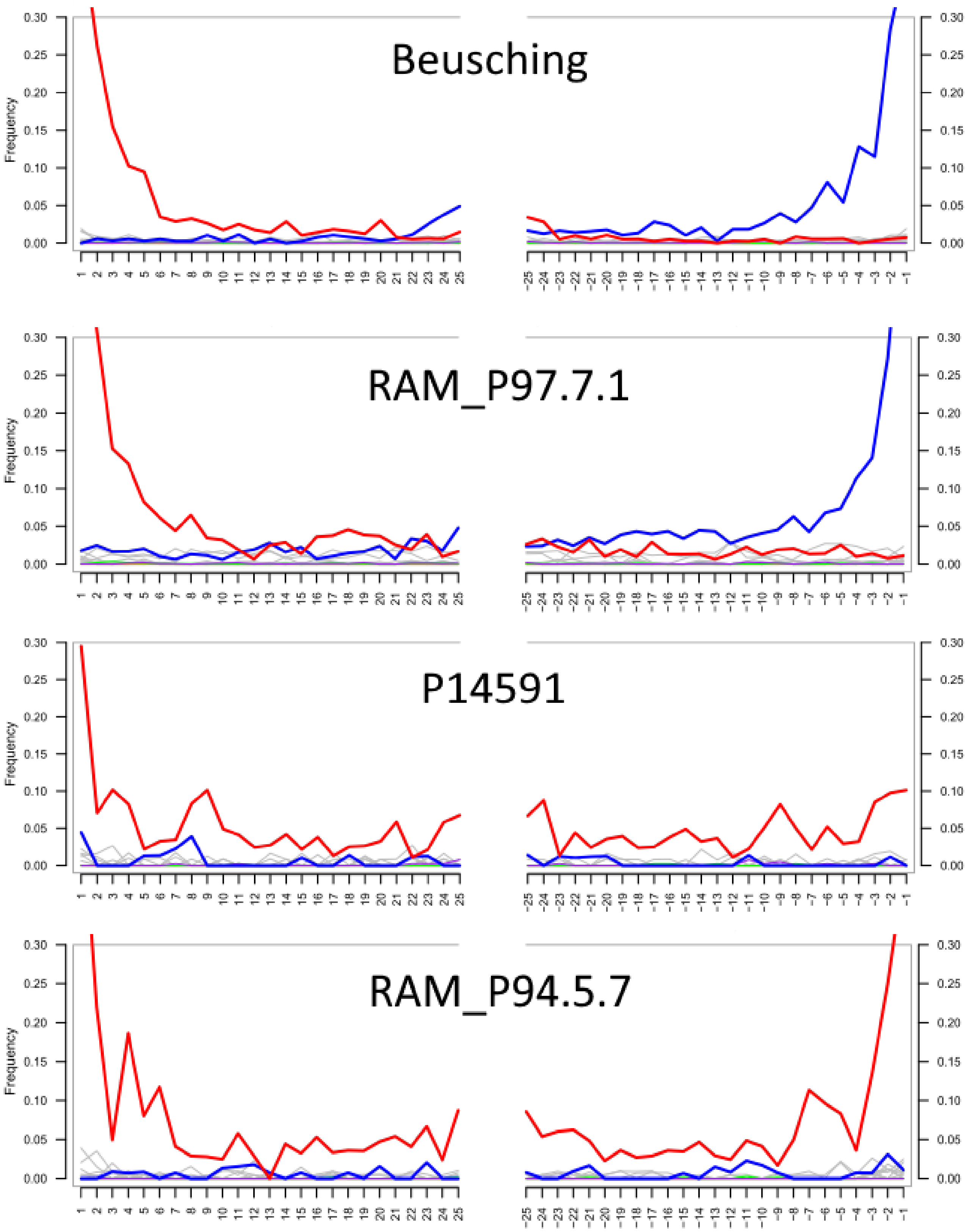
Beusching
RAM_P97.7.1
P14591
RAM_P94.5.7
Frequency
0.30
0.25
0.20
0.15
0.10
0.05
0.00

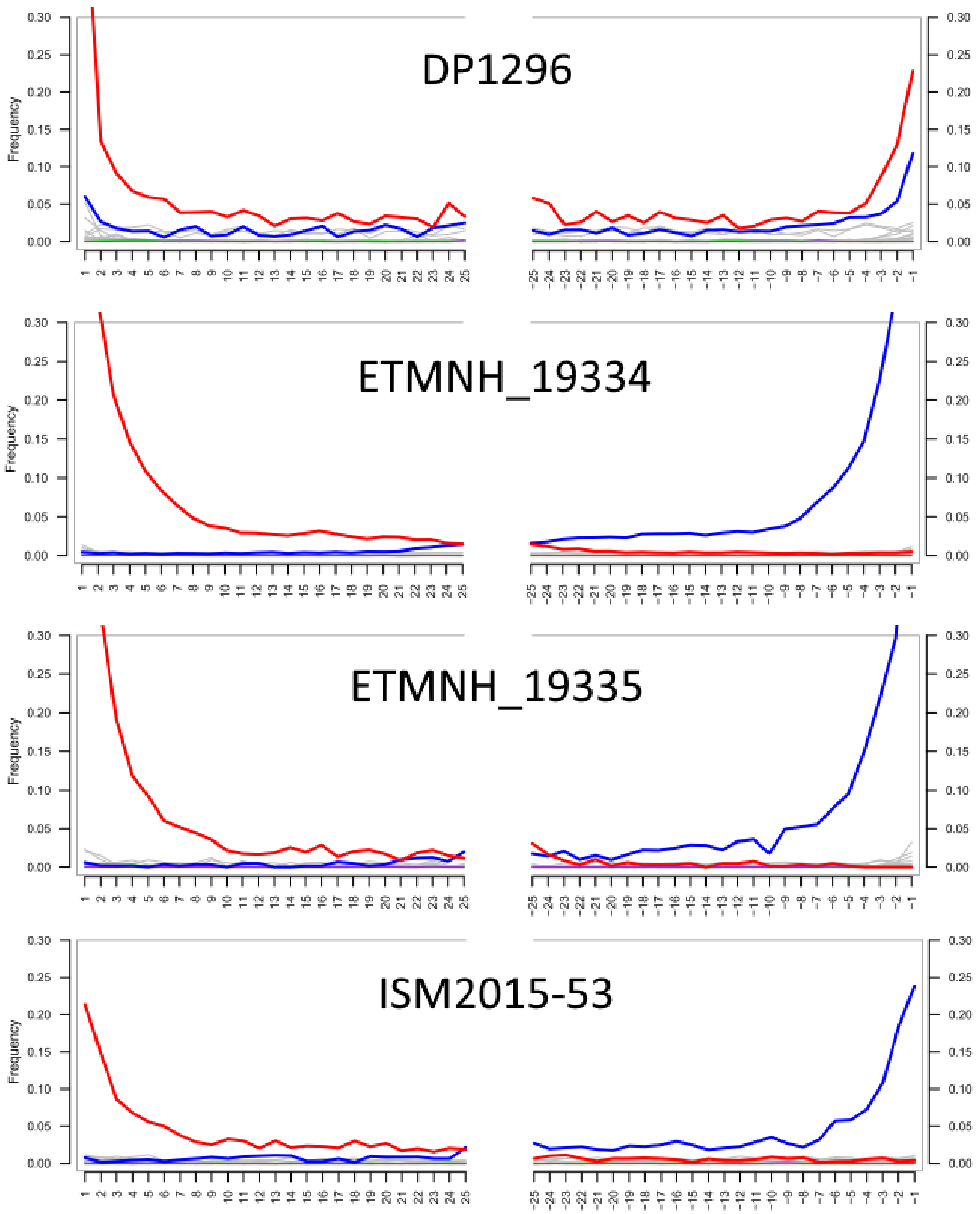
DP1296
ETMNH_19334
ETMNH_19335
ISM2015-53
Frequency

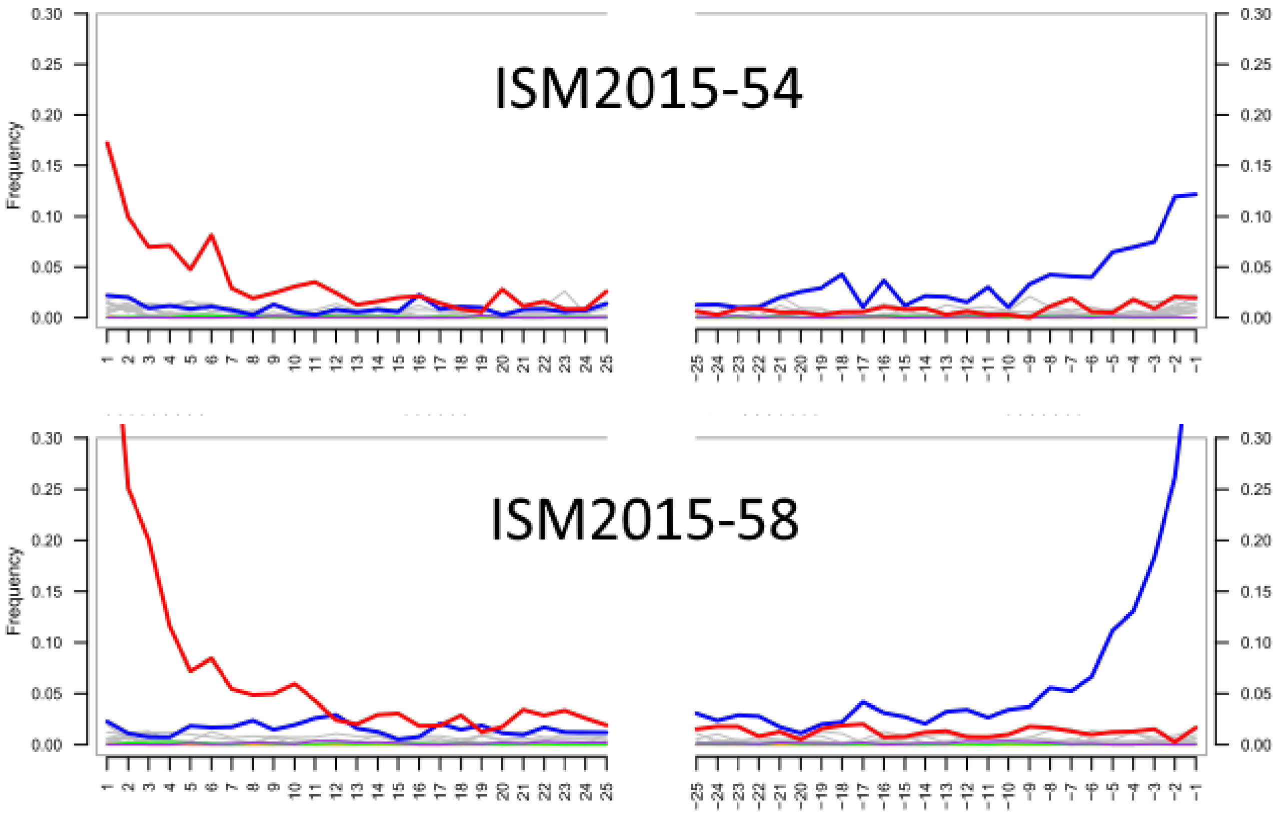
ISM2015-54
ISM2015-58
Frequency
0.30
0.25
0.20
0.15
0.10
0.05
0.00

Phylogenetic Analyses

Final consensus sequences were aligned using MUSCLE v3.8.31[13]. Substitution models were selected with jModelTest v2.1.4 [14] using the corrected Akaike information criterion (AICc) – TIM3+G (with mammoth outgroups) or TPM3uf+G (without outgroups). Rooted and unrooted maximum likelihood phylogenies were inferred in IQ-TREE v1.6.6 [15] using the selected substitution models and with 1000 bootstrap replicates. Phylogenies were rooted using both mammoths as outgroups, or using the midpoint for alignments without the mammoth sequences.

Outgroup-rooted (Supplementary Fig. 4) and midpoint-rooted (Supplementary Fig. 5) phylogenies had generally consistent topologies, with 4–5 well-supported (>95% bootstrap support) clades. The exact relationships between DP1296 and RAM P97.7.1 were variable between the two methods, with the outgroup-rooted phylogenies suggesting that the two specimens are placed in distinct parts of the tree. However, the support for this topology was relatively poor (bootstrap support of 79%). In comparison, the mastodon-only midpoint-rooted phylogeny supports a grouping of DP1296 and RAM P97.7.1 to the exclusion of other mastodons in the dataset (bootstrap support of 100%).

Maximum-likelihood phylogenies were also inferred with an HKY+G substitution model, as this was the best-supported model for the no-outgroup dataset available in BEAST used for molecular clock dating of the undated mastodons. Trees were inferred both with (Supplementary Fig. 6) and without (Supplementary Fig. 7) mammoth outgroups with 1000 bootstrap replicates. In both cases, topologies and support values were consistent with those inferred using the original best-fitting model.

Supplementary Figure 4. Maximum likelihood phylogeny for the mastodon dataset rooted with two mammoth outgroups using the best-selected model-TIM3+G. Phylogenies were constructed with 1000 bootstrap replicates. Support for each major node is shown. Scale bar represents substitutions per site.

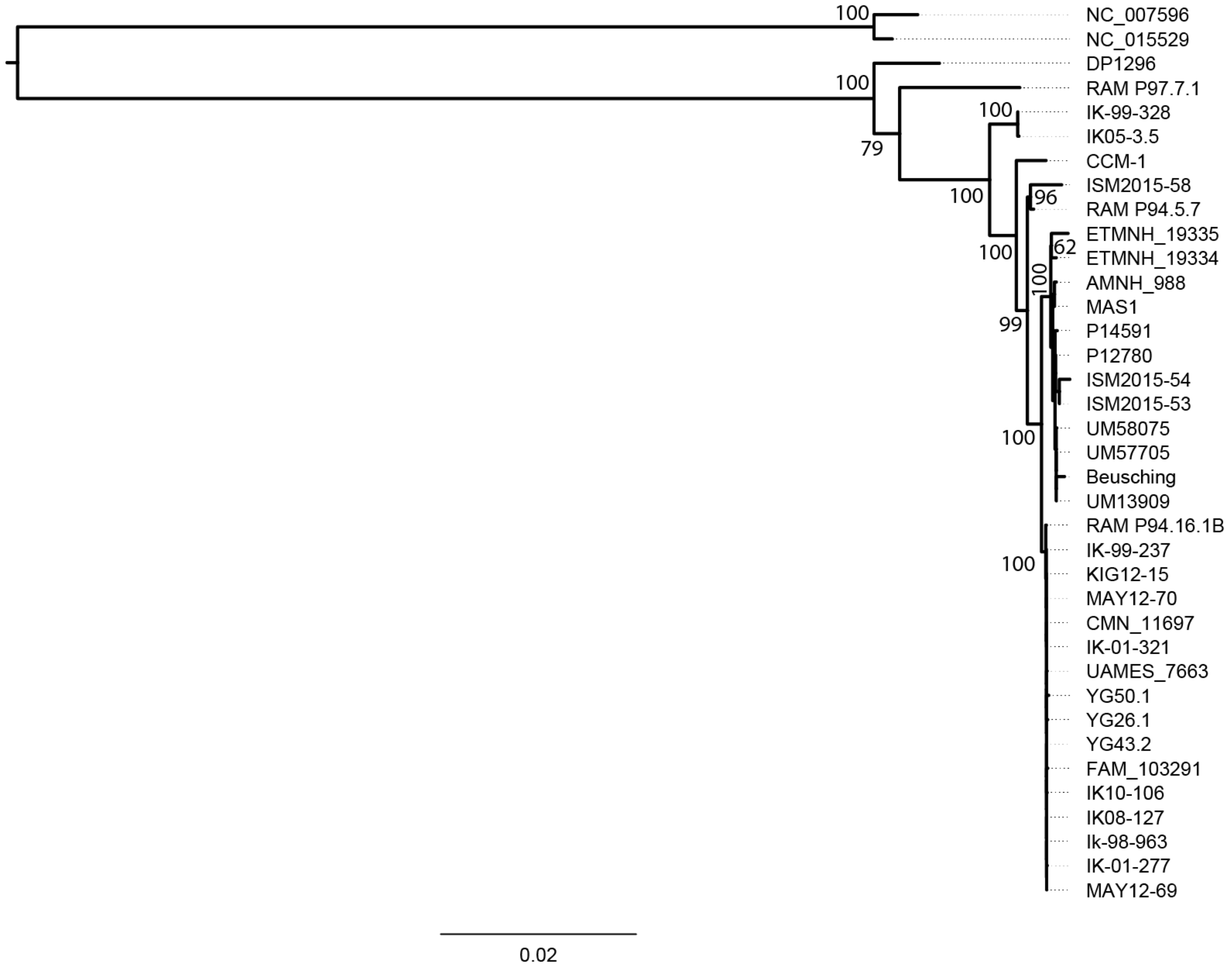

Supplementary Figure 5. Midpoint-rooted maximum-likelihood phylogeny for the mastodon dataset using the best-fitting model TPM3uf+G. Support for each major node is shown, based on 1000 bootstrap replicates. Scale bar represents substitutions per site.

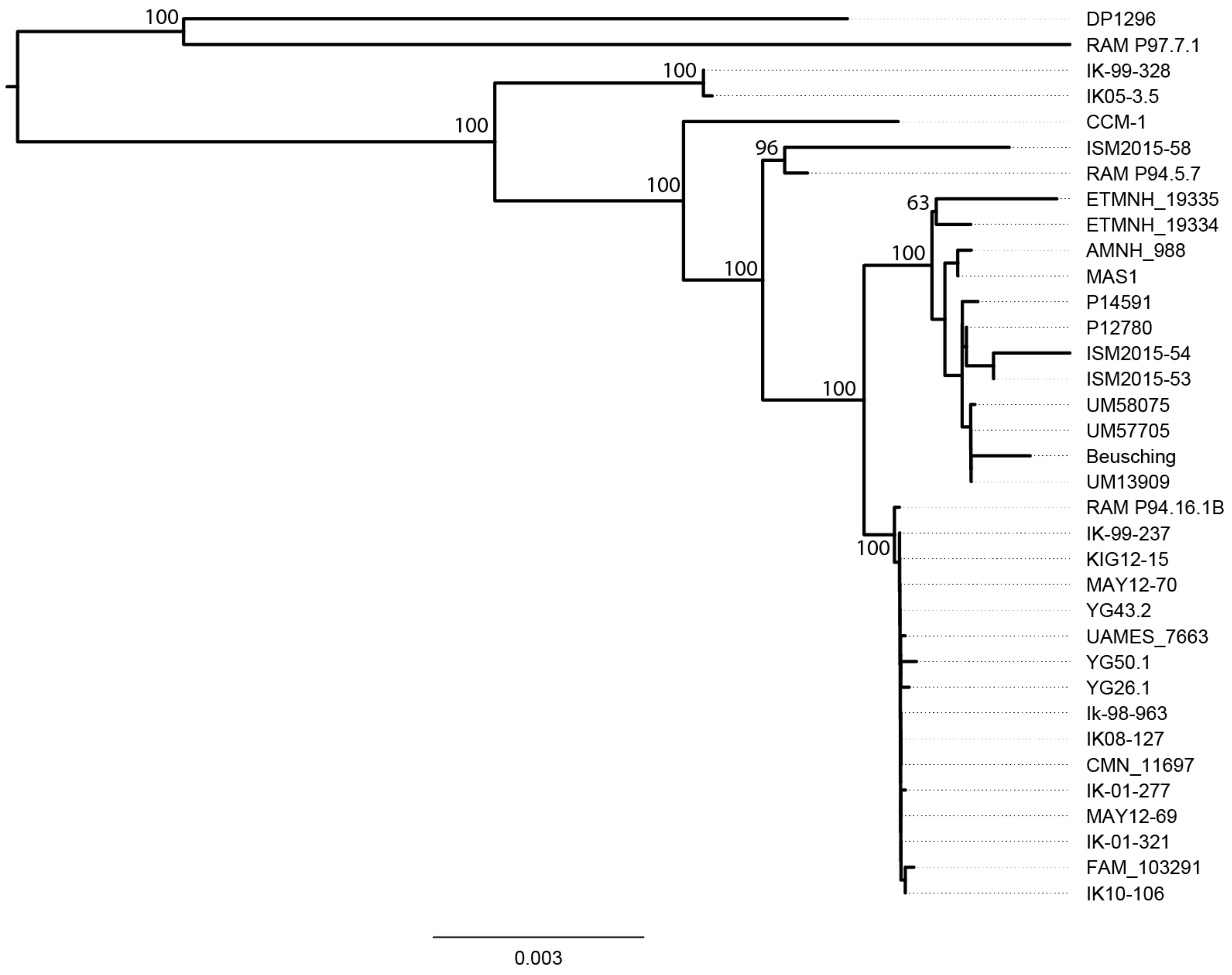

Supplementary Figure 6. Maximum-likelihood phylogeny for the mastodon dataset rooted with two mammoth outgroups using the best-fitting model available in BEAST (v1.8.0) HKY+G4. Support for each major node is shown, based on 1000 bootstrap replicates. Scale bar represents substitutions per site.

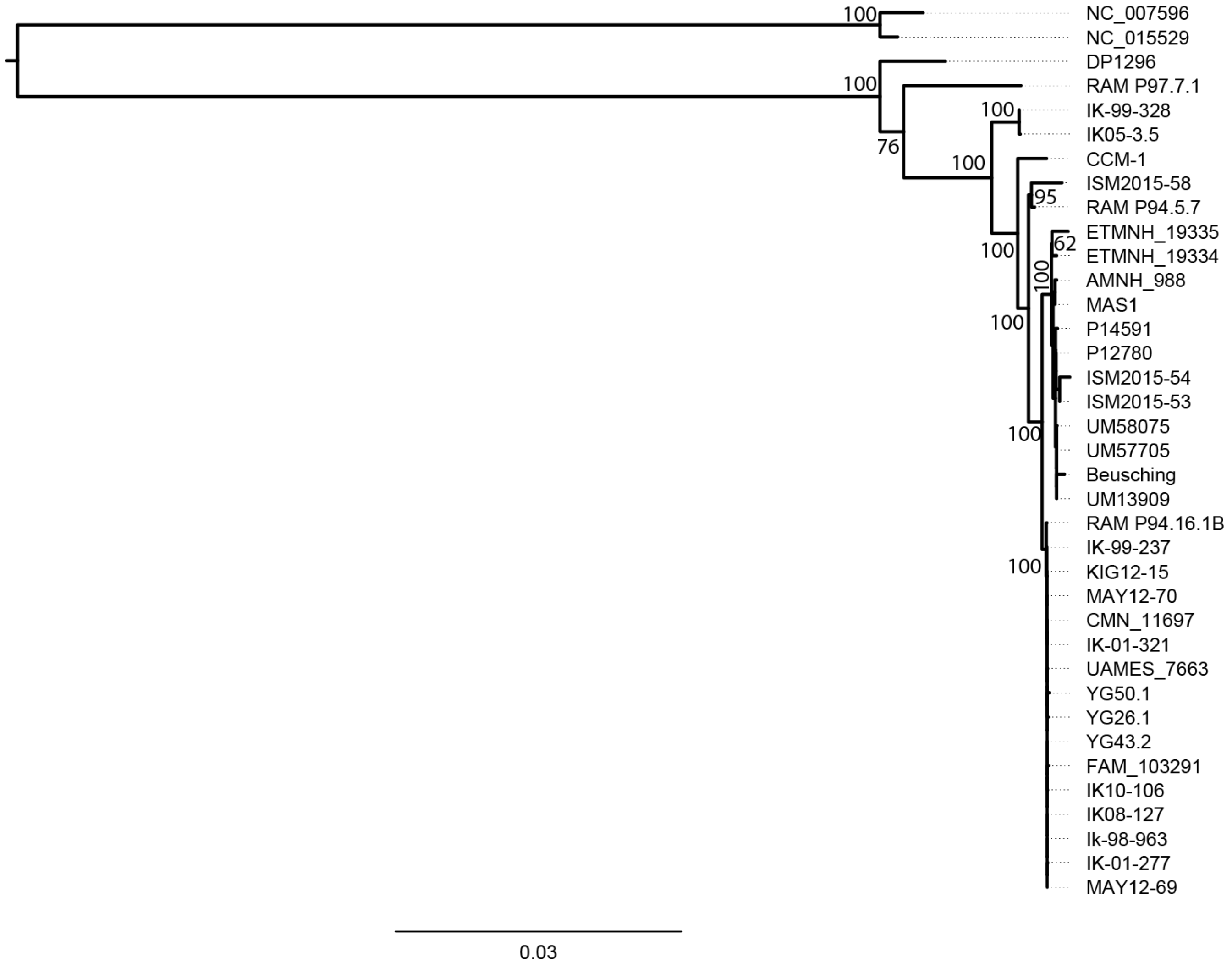

Supplementary Figure 7. Midpoint-rooted maximum-likelihood phylogeny for the mastodon dataset using the best-fitting model available in BEAST (v1.8.0) HKY+G4. Support for each major node is shown, based on 1000 bootstrap replicates. Scale bar represents substitutions per site.

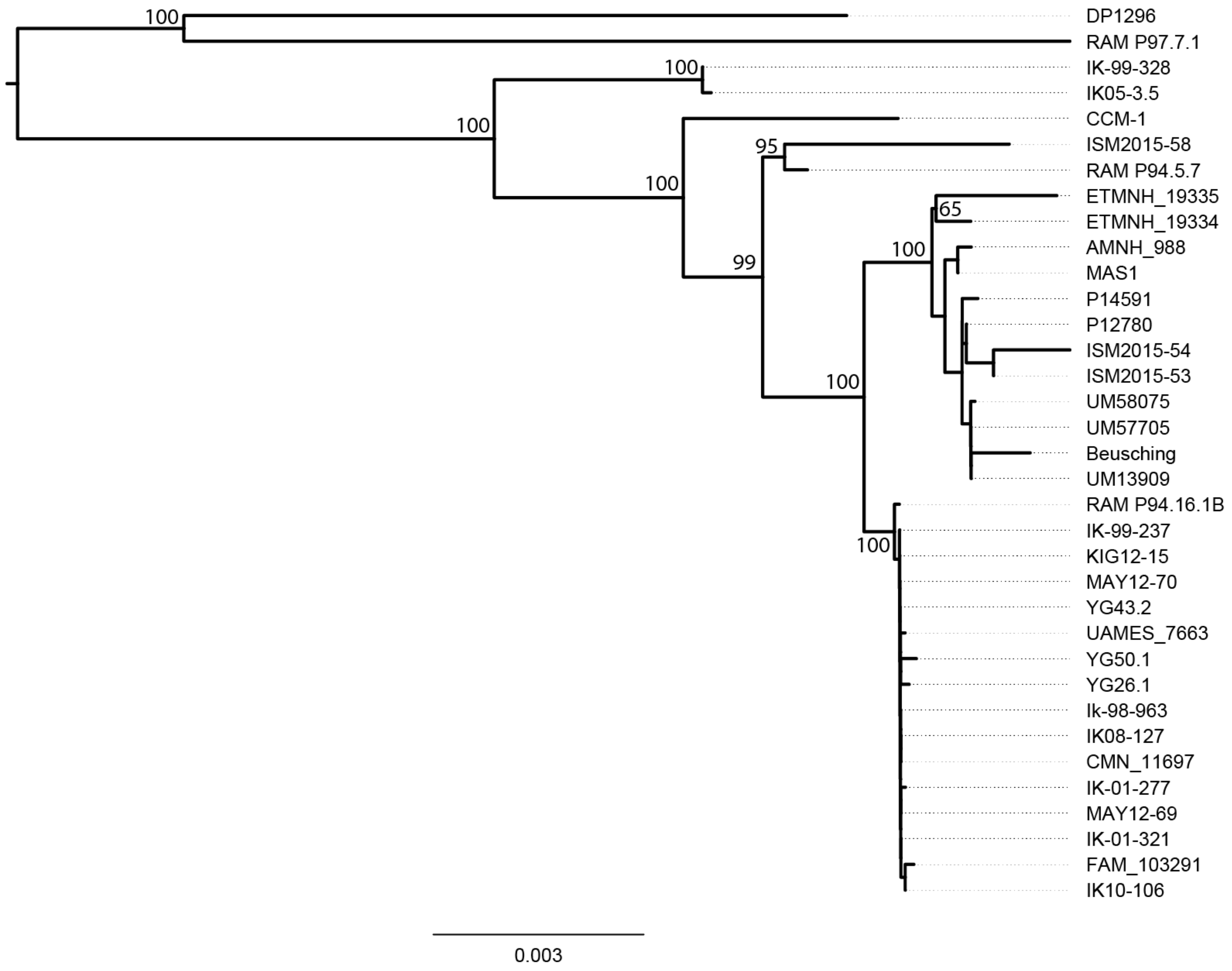

To further examine the relationships of DP1296 and RAM P97.7.1 as well as the rest of the mastodons in our data set, we also inferred phylogenies in BEAST (v1.8.0) [16]. Phylogenies were inferred using an HKY+G4 substitution model and default priors with a chain length of 10 million steps (logging every 1000 steps; 10% burn-in).

Phylogenies inferred both with (Supplementary Fig. 8) and without mammoth outgroup sequences (Supplementary Fig. 9) had identical topologies for all mastodons. Additionally, both trees had a high posterior probability (>0.95) of a monophyletic clade containing DP1296 and RAM P97.7.1.

Supplementary Figure 8. Maximum-clade-credibility tree for the mastodon dataset with two mammoth outgroups inferred in BEAST (v1.8.0) using an HKY+G4 substitution model. Posterior probability for each major node is indicated. Scale bar represents substitutions per site.

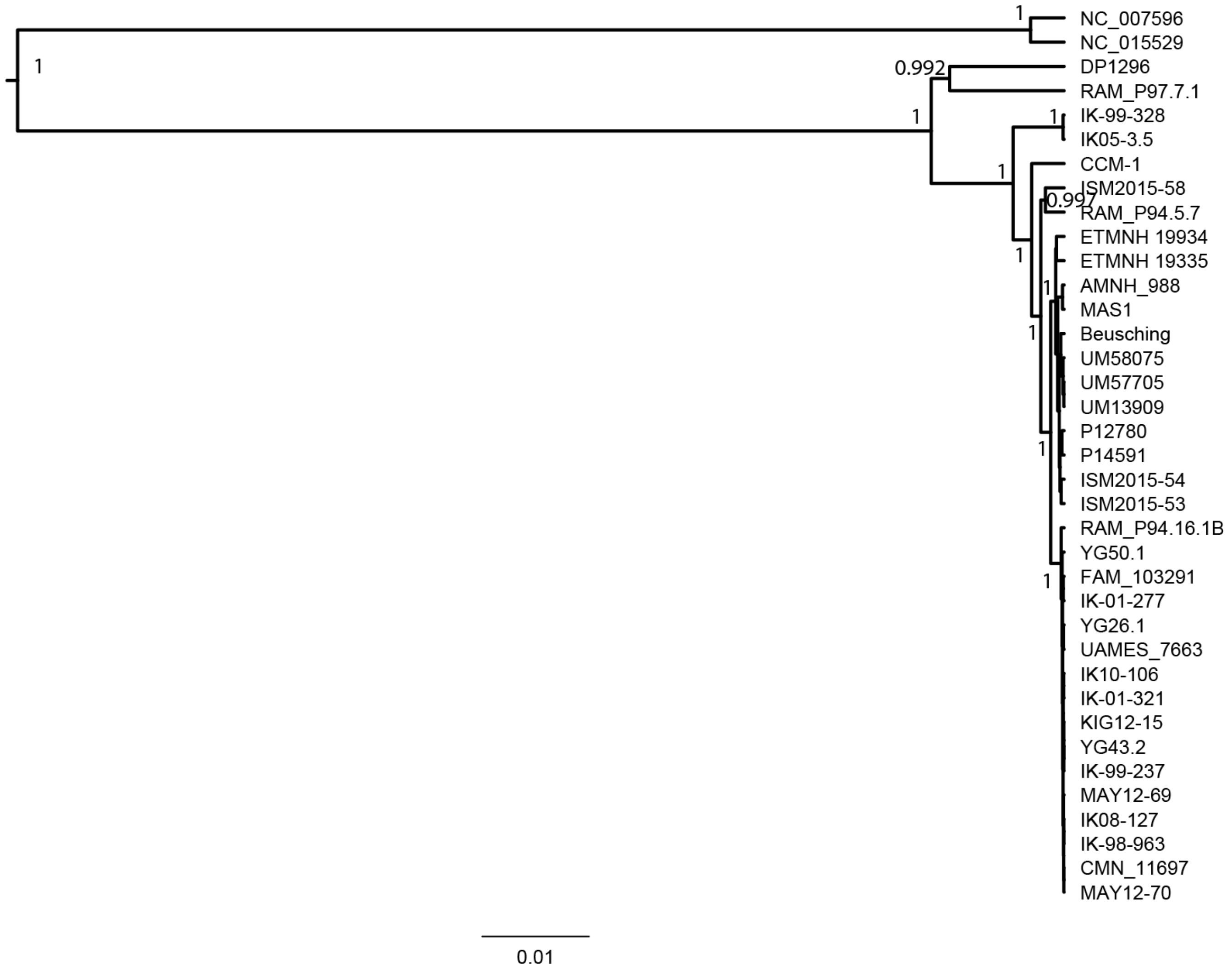

Supplementary Figure 9. Maximum-clade-credibility tree for the mastodon dataset inferred in BEAST (v1.8.0) using an HKY+G4 substitution model. Posterior probability for each major node is indicated. Scale bar represents substitutions per site.

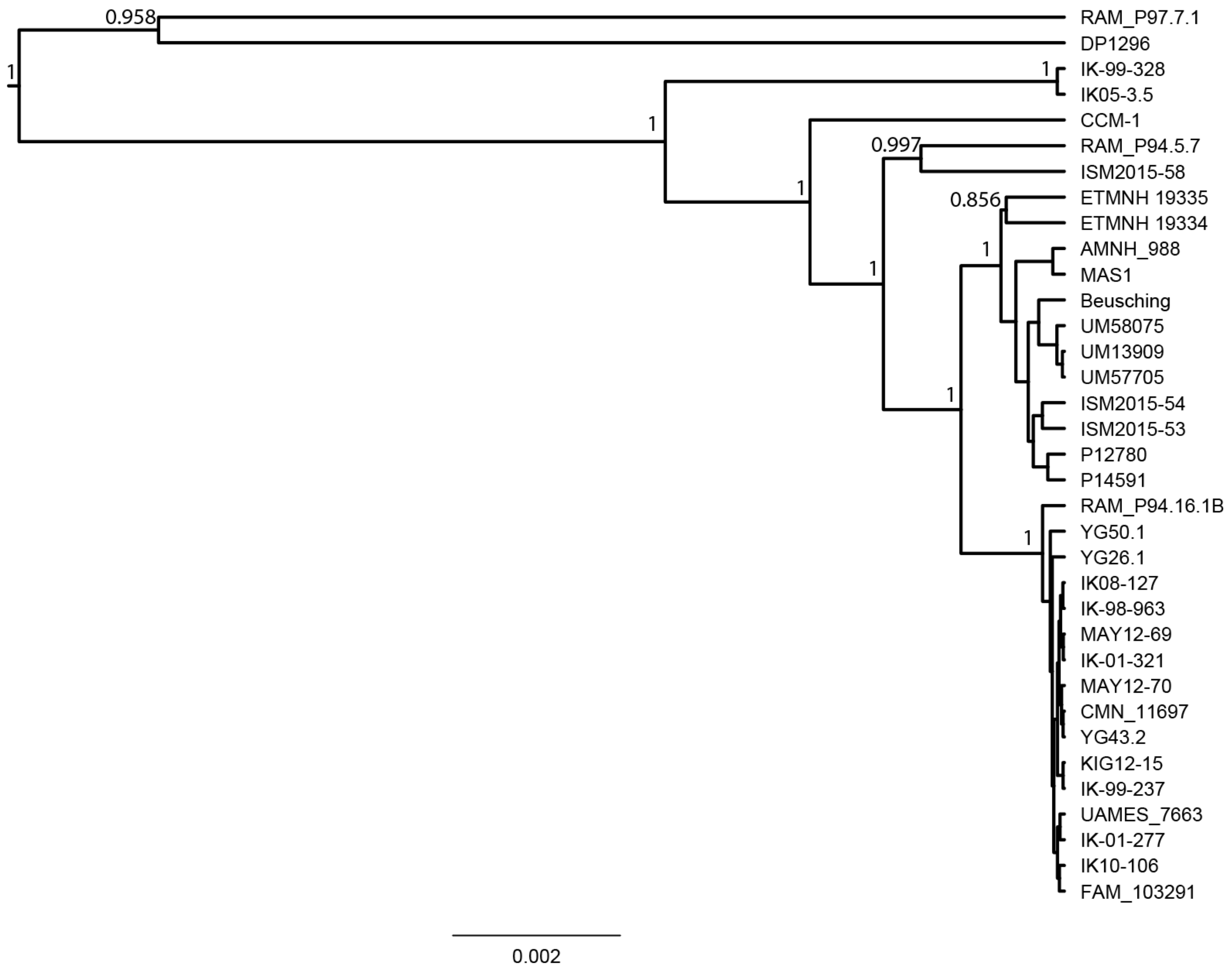

Sequence and Topology Validation

To ensure that the topology of our mastodons was not being affected by the >3 standard deviation coverage area masking, we also generated consensus sequences without masking these regions. Consensus sequences were called using a 50% strict majority criterion and the repetitive portion of the D-loop was masked as before. Model selection and maximum-likelihood phylogenies (with and without outgroups) were inferred as above, except with 100 bootstrap replicates.

Phylogenies inferred both with (Supplementary Fig. 10) and without mammoth outgroups (Supplementary Fig. 11) had identical topologies to their respective standard deviation masked counterparts. Bootstrap supports for the major clades were also relatively consistent between standard deviation masked and non-masked datasets.

Supplementary Figure 10. Maximum-likelihood phylogeny for the mastodon dataset without additional standard deviation filtering, rooted with two mammoth outgroups using the best-fitting model TIM3+G. Support for each major node is shown, based on 1000 bootstrap replicates. Scale bar represents substitutions per site.

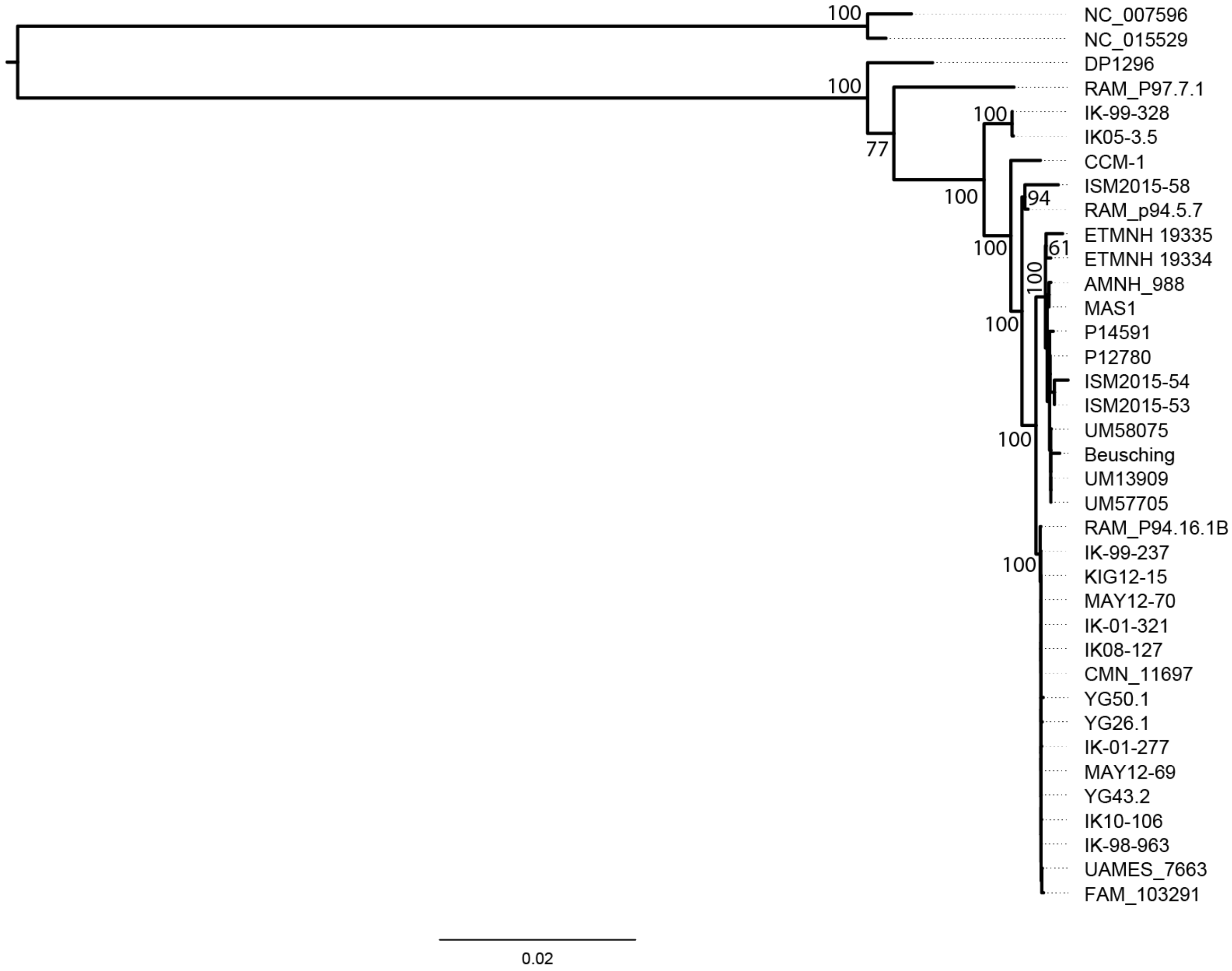

Supplementary Figure 11. Midpoint-rooted maximum-likelihood phylogeny for the mastodon dataset without additional standard deviation filtering using the best-fitting model TPM3uf+G. Support for each major node is shown, based on 1000 bootstrap replicates. Scale bar represents substitutions per site.

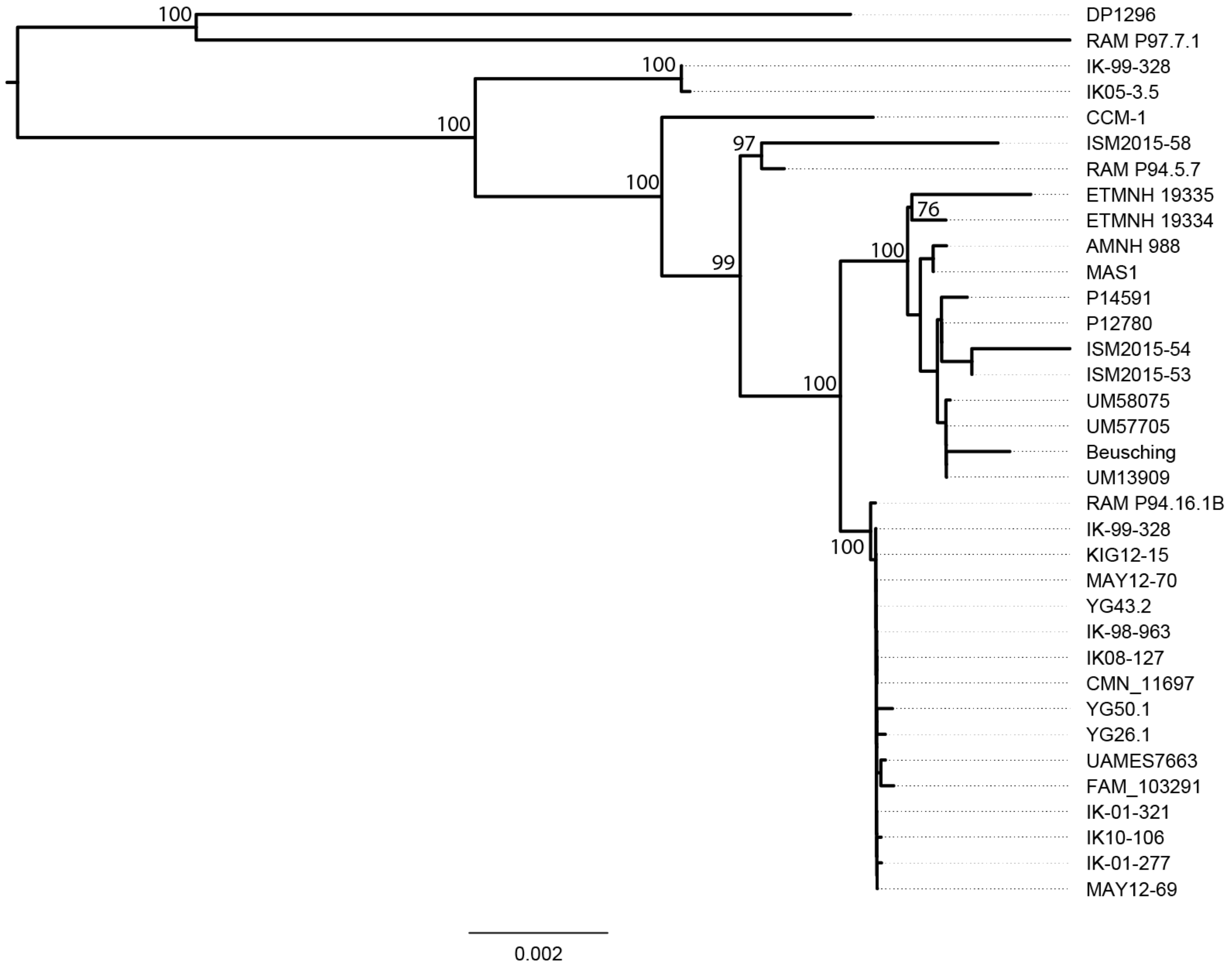

To ensure that our phylogenies were not overly influenced by the presence of low-level exogenous sequences in the previous identified reads, we also called a consensus using only reads that returned a BLAST match within Afrotheria. The top 5 BLAST results for each hit were summarized in MEGAN (v6.12.3) [17], and reads identified as belonging to at least Afrotheria (i.e., Afrotheria or more specific taxonomic assignments within the superorder) were extracted. Consensus sequences, multiple alignment, model selection, and phylogeny construction for BLAST-filtered data were generated identically to the >3 standard deviation masked data, except with 100 bootstrap replicates.

Visual inspection of the BLAST-filtered alignments revealed that the stacks previously observed were filtered out of the final alignments (Figs. A12; A13A). In comparison, samples without observable stacks lost reads in a relatively uniform pattern throughout the alignment (Supplementary Fig. 13B), suggesting that in these cases BLAST is likely filtering out short or damaged endogenous fragments as opposed to exogenous contamination. Percent coverage of the NC_035800 reference and depth of

coverage were largely unchanged for most samples (Supplementary Table 32), although percent coverage did moderately correlate with mean distance from either for the two *Mammut americanum* sequences in GenBank ($R^2 = 0.5492$) (Supplementary Fig. 14).

BLAST-filtering had no effect on the best substitution model selected for the datasets with or without outgroup sequences. Topologies for the no outgroup phylogeny was likewise identical, with only a small decrease in bootstrap support observed for the RAM P94.5.7 and ISM2015-58 clade (Supplementary Fig. 15). In comparison, the outgroup-rooted topology had DP1296 and RAM P97.7.1 grouped together (as observed in the BEAST and no outgroup trees), albeit with poor bootstrap support (46%) (Supplementary Fig. 16).

Supplementary Figure 12. A subset of the mapped reads from sample F:AM 103291 against the NC_035800 *Mammut americanum* reference genome. A portion of the 16S rRNA gene is visible. (A) Visible “stacks” of reads formed by the mapping of short exogenous fragments to a portion of the 16S rRNA gene. (B) The same region as in A, but following BLAST-filtering to retain only reads that returned a result within Afrotheria.

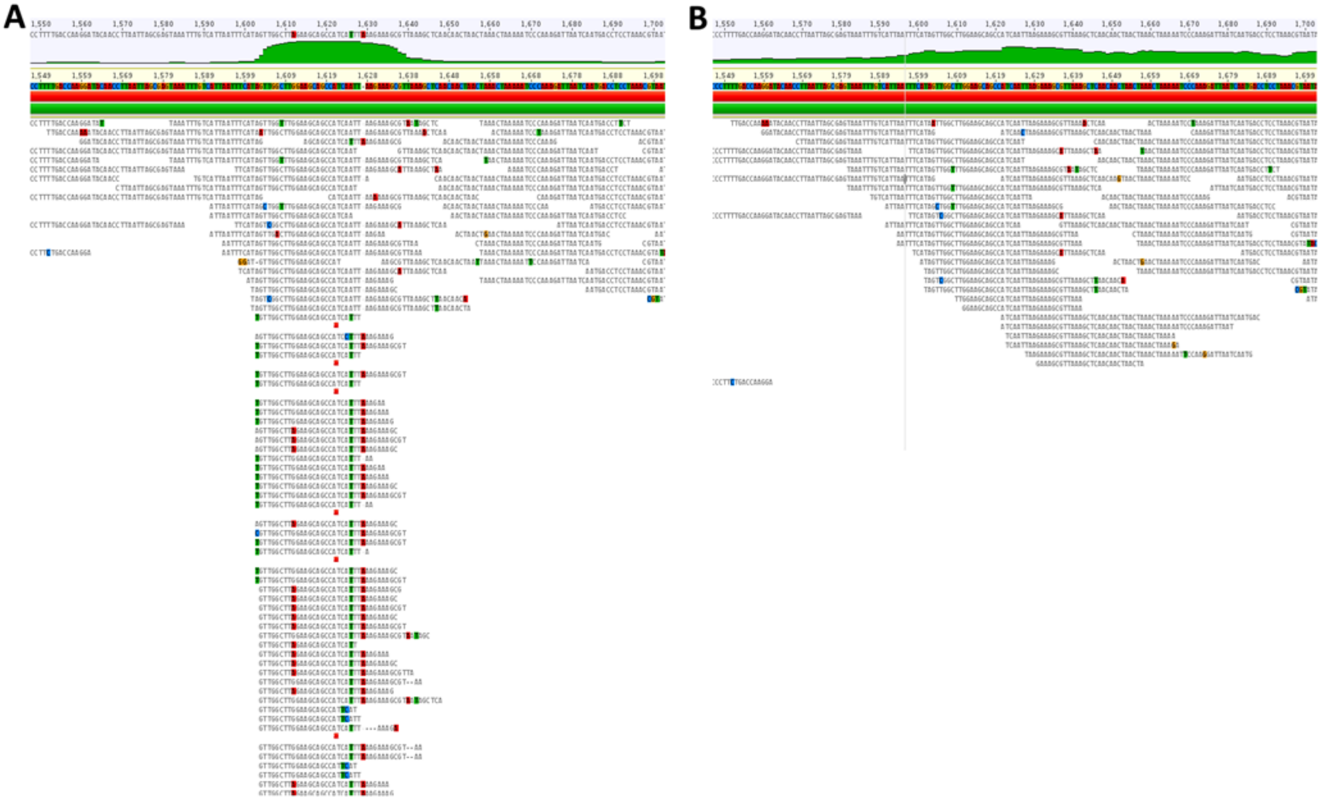

Supplementary Figure 13. Coverage depth across the mitochondrial genome for two American mastodons samples mapped against the NC_035800 reference genome before BLAST-filtering (black), the retained reads that returned a hit within Afrotheria (blue), and the removed reads following BLAST-filtering (red). (A) Coverage depth for F:AM 103291, a clade Y mastodon which contains a previously sequenced mastodon mitochondrial genome (EF632344/IK-99-237). Removed reads primarily correspond to the stack of reads around position 1610, within the 16S rRNA region. (B) Coverage depth for DP1296, a clade M mastodon and one of the most diverged mastodons in our dataset. Unlike for samples with visible stacks, removed reads here span the entirely of the alignment. This suggests that the removed reads are likely the result of spurious hits, possibly as a result of short fragment sizes or damage.

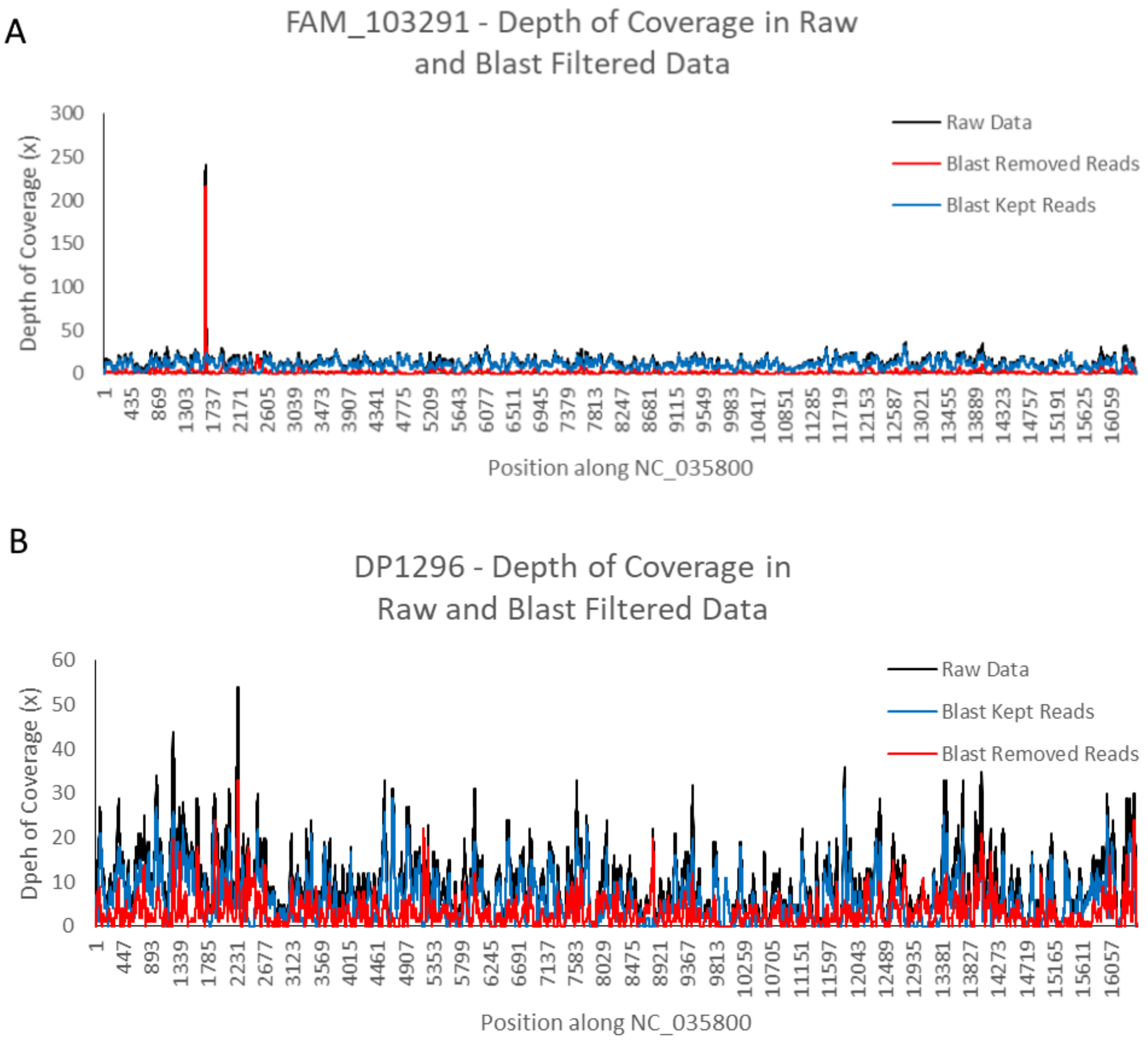

Supplementary Table 32. Mapping statistics for the raw alignment (without Stdev or BLAST-filtering) and for the BLAST-filtered alignment. The % coverage of the NC_035800 reference (% Cov), mean depth of coverage (Depth (x)), the number of mapped reads greater than or equal to 24 bp and with a minimum map quality of 30 (# Reads), and the mean length of mapped reads (Mean Length) are given.

Specimen	Raw ("Non-BLAST")				BLAST-Filtered			
	% Cov	Depth (x)	# Reads	Mean Length	% Cov	Depth (x)	# Reads	Mean Length
AMNH_988	97.8	9.1	3,734	40.40	96.1	8.2	3,228	42.10
Beusching	86.2	7.2	3,275	37.26	80.2	5.6	2,394	39.81
CCM-1	98.3	12.7	4,148	50.56	97.7	11.5	3,542	53.44
CMN_11697	97.4	8.8	3,066	47.27	96.6	8.2	2,754	49.18
DP1296	92.2	10.0	4,279	38.63	76.1	6.3	2,509	42.56
FAM_103291	99.4	13.6	4,974	44.98	98.4	11.1	3,783	48.48
IK05-3.5	99.8	85.7	24,965	56.53	99.8	79.3	22,006	59.35
IK08-127	87.1	4.5	1,492	51.97	86.5	4.3	1,388	53.43
IK10-106	99.5	14.8	3,390	72.11	99.4	14.6	3,286	73.27
IK-01-277	97.7	8.0	1,984	67.08	97.7	7.8	1,887	68.74
IK-01-321	99.6	16.0	4,341	60.82	99.6	15.5	4,083	62.43
IK-98-963	99.1	9.5	2,422	64.74	99.1	9.3	2,339	65.74
IK-99-328	99.0	10.3	2,598	65.61	98.7	9.9	2,409	67.57
ISM2015-53	98.5	13.6	4,111	54.40	98.0	12.6	3,691	56.09
ISM2015-54	85.3	5.9	1,827	54.63	82.9	4.8	1,474	55.89
ISM2015-58	84.3	5.3	1,951	46.64	76.6	4.0	1,471	48.07
KIG12-15	99.8	106.9	26,846	65.56	99.8	104.7	25,864	66.65
MAY12-69	98.7	8.1	1,746	76.63	98.5	8.0	1,707	77.51
MAY12-70	99.6	32.6	10,489	51.23	99.2	31.1	9,700	52.82
P12780	99.7	35.1	10,997	52.53	99.7	33.3	10,111	54.28
P14591	88.5	5.9	2,174	45.41	86.4	5.1	1,798	48.15
RAM_P94.5.7	95.4	6.8	2,685	42.01	91.1	5.7	2,159	44.67
RAM_P94.16.1B	91.7	6.4	2,175	49.13	90.1	5.9	1,919	51.68
RAM_P97.7.1	96.5	12.7	4,790	43.90	87.9	9.5	3,385	46.56
ETMNH 19334	99.4	142.1	51,113	45.78	99.0	127.9	44,982	46.84
ETMNH 19335	91.0	7.7	3,134	41.09	87.9	6.5	2,560	42.56
UAMES_7663	96.6	8.5	2,485	56.92	96.1	8.2	2,304	58.73
UM_13909	98.3	11.2	3,657	50.56	97.9	10.6	3,349	52.19
UM_57705	98.1	12.5	4,818	42.80	97.4	11.1	4,079	45.05
UM_58075	99.0	14.9	4,630	52.89	98.8	14.0	4,198	55.02
YG_26.1	99.7	23.1	6,495	58.53	99.7	22.0	5,995	60.48
YG_43.2	99.6	17.0	5,430	51.71	99.6	16.1	4,906	54.01
YG_50.1	88.5	4.8	1,684	47.99	86.1	4.3	1,474	50.24

Supplementary Figure 14. Regression analysis between mean distance to one of the two *Mammut americanum* reference genomes available in GenBank (NC_035800 and EF632344), and the difference in mapped % coverage of the NC_035800 reference for each specimen before and after BLAST-filtering. R^2 for the regression equals 0.5492. Distances were calculated using the dist.dna function within the R ape package [18].

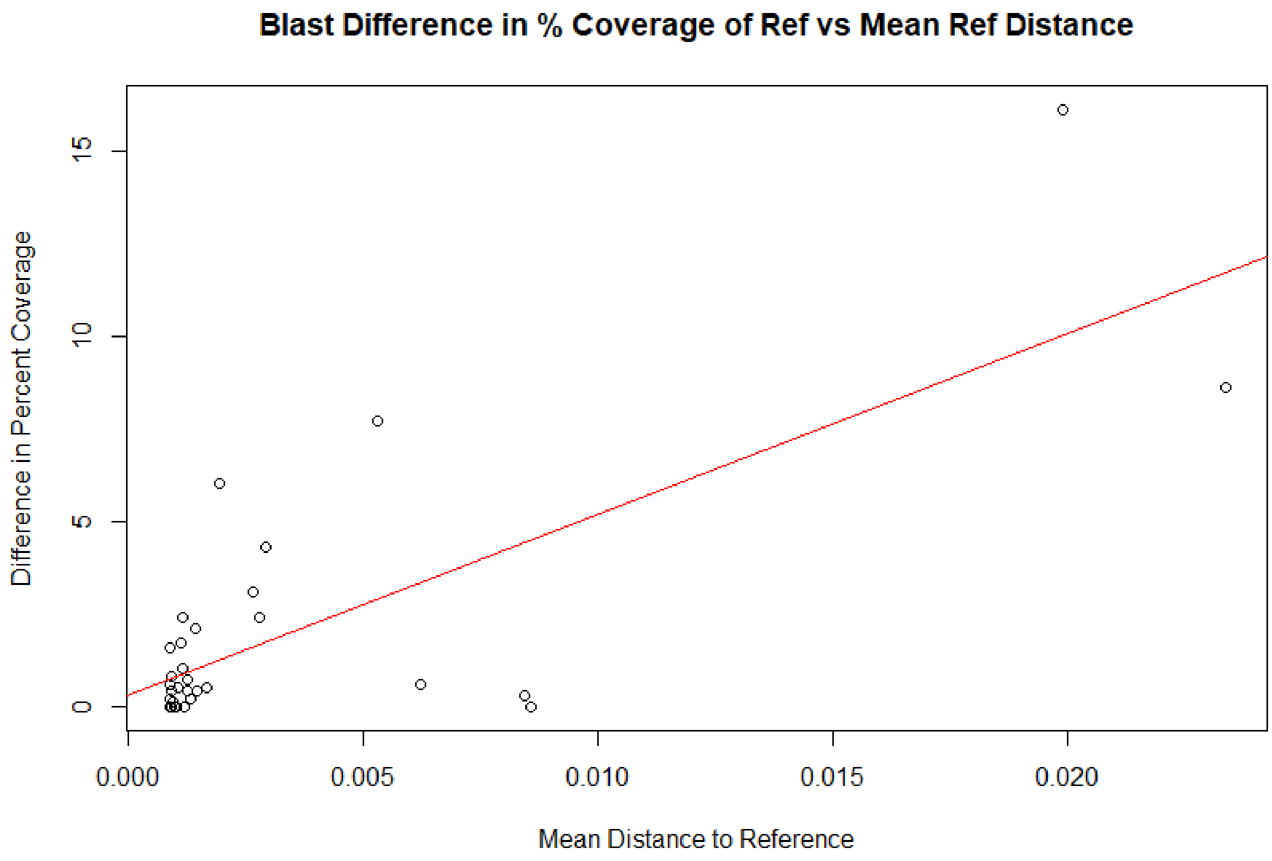

Supplementary Figure 15. Midpoint-rooted maximum-likelihood phylogeny for the BLAST-filtered mastodon dataset using the best-fitting model TPM3uf+G. Support for each major node is shown, based on 1000 bootstrap replicates. Scale bar represents substitutions per site.

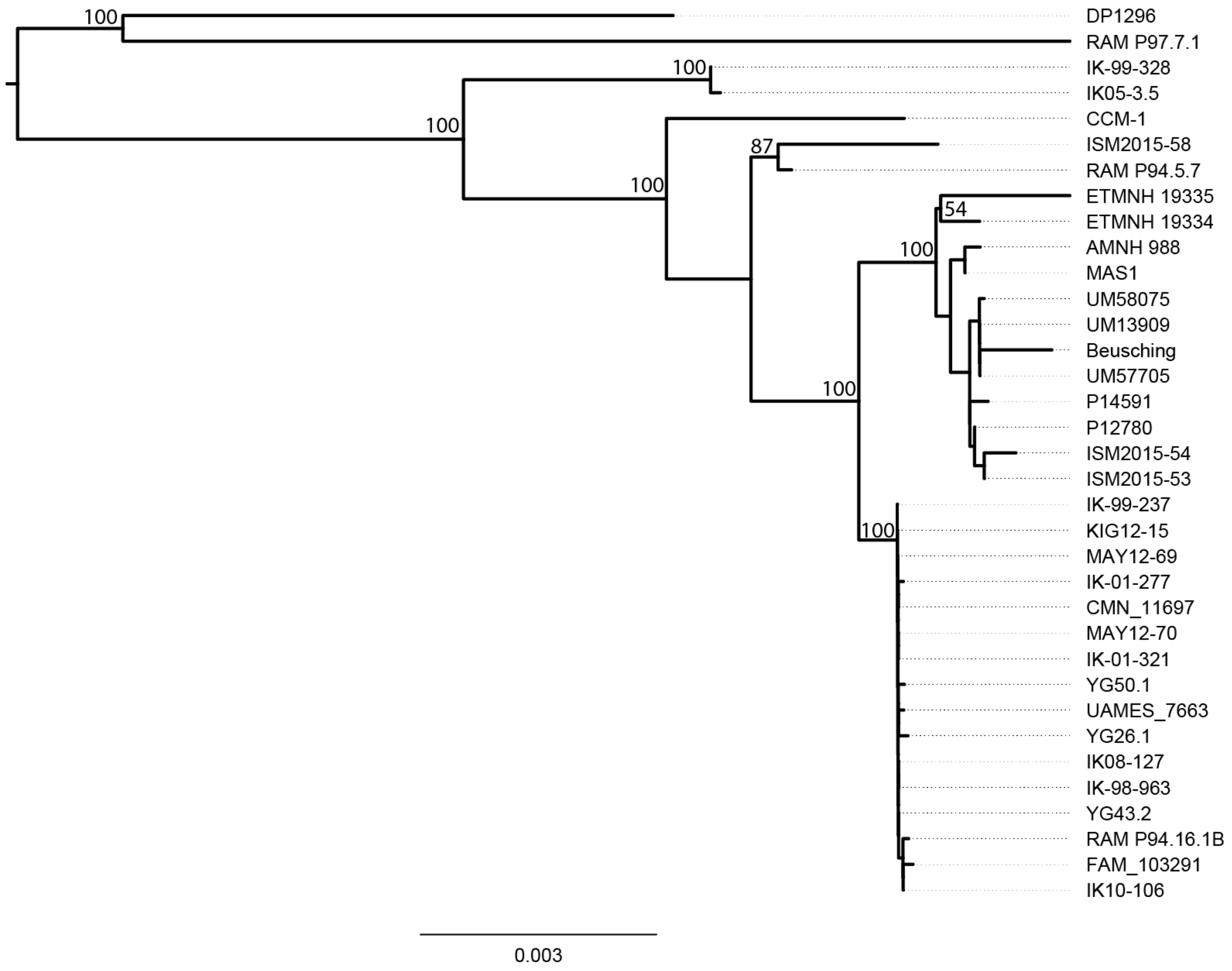

Supplementary Figure 16. Maximum-likelihood phylogeny for the BLAST-filtered mastodon dataset rooted with two mammoth outgroups using the best-fitting model TIM3+G. Support for each major node is shown, based on 1000 bootstrap replicates. Scale bar represents substitutions per site.

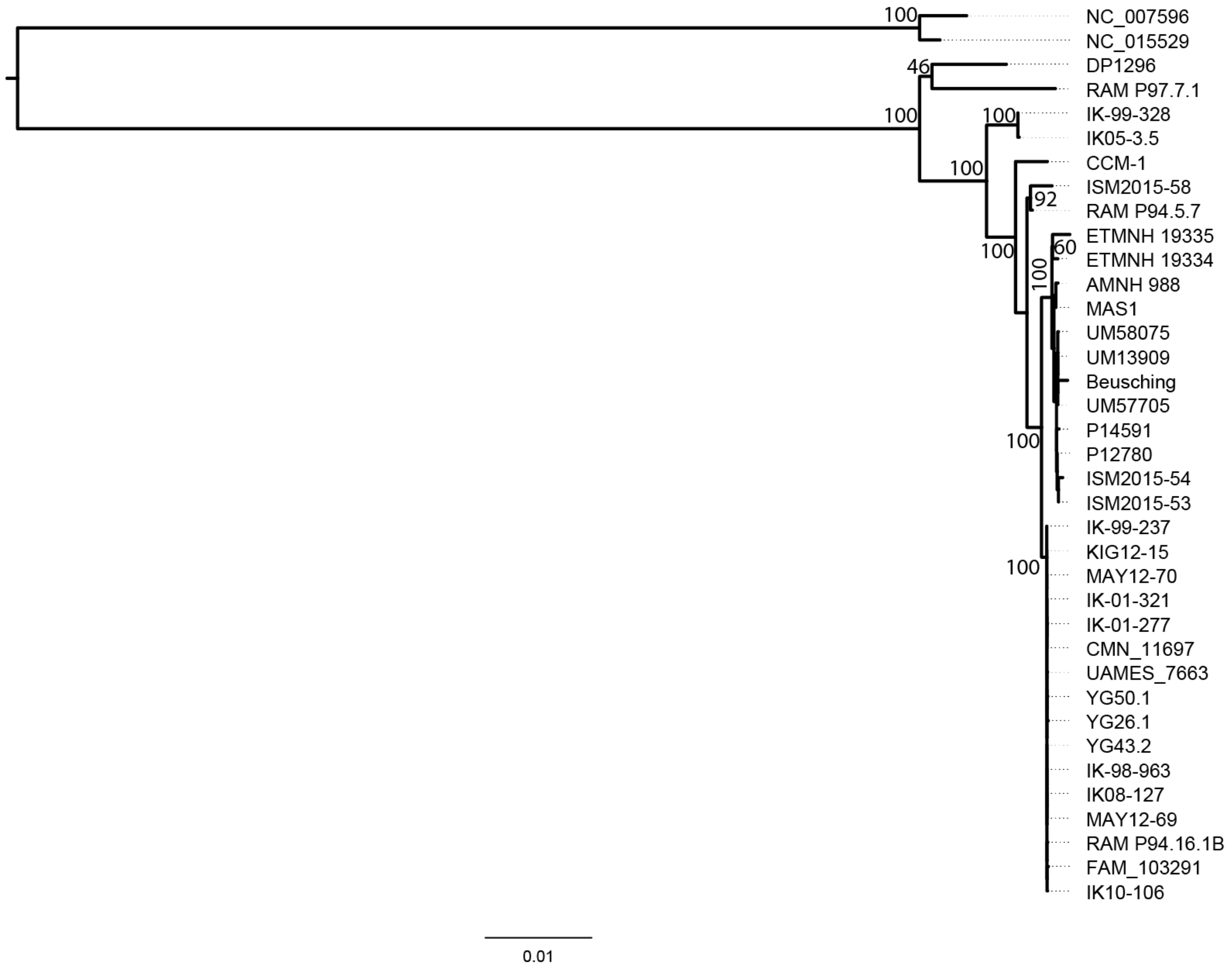

Leave-One-Out Analysis

We conducted a leave-one-out (LOO) analysis on our dated specimens, to see how well our current model estimates the ages of tips for which external calibration was available. All specimens were run using the best-fitting models described above, with both a strict and uncorrelated relaxed lognormal clock in BEAST v1.8.0[16]. The ages of the left-out dated specimens were assigned a diffuse gamma prior (shape = 1; scale = 200,000), with an upper bound at 800,000 and a lower bound at zero, with the exception of CCM-1. This specimen was analysed twice, the second time (CCM-1 V2) being fit with an identical gamma prior truncated between 50,000 and 800,000. Chains were run for 500 million steps (sampling every 10 thousand), with two separate datasets: using all 35 complete specimens and using only dated specimens (n=13).

Similarly to previous molecular dating analyses, LOO analyses resulted in fairly wide 95% HPD intervals using both an uncorrelated relaxed lognormal (Supplementary Table 33) and a strict clock (Supplementary Table 34). Expectedly, 95% HPD intervals estimated using a relaxed clock were almost always larger (range: 1.17–11.02 fold increase), except in one case (DP1296 with only dated specimens) where the relaxed lognormal clock produced a 95% HPD interval 0.98-fold the size of that obtained with the strict clock.

LOO variants produced a 95% HPD interval containing the full 2-sigma range of the left-out specimens in the majority of cases (Supplementary Table 35), with the date range of only three specimens (DP1296, MAS1, and P12780) failing to be recovered in some analyses. Of these, DP1296 falls within the sister clade to all other mastodons in our phylogeny and is genetically the most distant from any other dated specimens. This may partially explain the trouble in analysing this specimen and the very wide (~431 kya and 744 kya) 95% HPD intervals produced for datasets with all 35 mastodon mitochondrial genomes. When we reduced the dataset to include only the dated specimens, the radiocarbon range for DP1296 was recovered in its 95% HPD interval, although the 95% HPD intervals remain very large, likely still as a result of its position relative to other calibration points.

The poor recovery of the ages for MAS1 and P12780 is harder to explain. Shapiro et al.[19] previously observed that in both simulated and empirical datasets they failed to recover the true ages of the specimens in some cases, and outlined some possible sources for this discrepancy. It seems unlikely that the poor recovery of the ages of MAS1 and P12780 is due to errors in the sequences themselves, given that P12780 has one of the best assemblies and highest coverages of our specimens (Supplementary Table 30), and MAS1 is the current *M. americanum* mitochondrial RefSeq entry (NC_035800). Closer examination of the posterior distributions from the LOO analyses of the ages of both of these specimens in the dated-only dataset reveals that the clock.rate parameter abuts the upper bound (Supplementary Fig. 17), suggesting that the prior for this parameter was poorly specified. However, it remains unclear why the true ages of these two specimens fail to be recovered in the 95% HPD for the LOO strict clock analyses when including the entire dataset, where such issues with the clock.rate parameter are not observed. Still these prior misconstraints may have influenced the log marginal likelihoods obtained under path sampling or stepping-stone sampling (Appendix A).

Given the wide 95% HPD intervals that molecular clock dating of tips tends to produce[19], we wanted to compare the relative distances of the maximum (i.e. the mode - age with the highest probability density) and the median of the posterior distribution (Supplementary Table 35) to the dated age. In 47 out of 56 LOO analyses, the mode of the posterior distribution was closer to the mean of the

radiocarbon/ESR/stratigraphic estimate than the median. Among these 47 analyses, the relative differences over the length of the 95% HPD between the dated age and mode versus the median, ranged from about a 1.03 fold (CCM-1 (no bounds) with an uncorrelated lognormal clock using all specimens) to a 745.28 fold (ISM2015-58 with a strict clock and only dated specimens) decrease in distance from the mode to the dated mean. In general the modes were proportionally closer to the dated age in LOO analyses using the uncorrelated lognormal clock, although some of this may be explained by the much smaller 95% HPD intervals obtained using the strict clock.

Analyses with only four specimens (DP1296, Buesching, ISM2015-54, and ETMNH 19335) produced posterior probability distributions where the median age estimate was closer than the mode to the radiocarbon date. As before, issues with DP1296 likely lie with the position of this specimen in our phylogeny and the absence of other calibration points near it. However, as with the successful recovery of the radiocarbon range in the 95% HPD interval, the mode of the posterior distribution is also found to be closer to the radiocarbon mean date when the analysis is restricted to just dated specimens.

The Buesching, ISM2015-54, and ETMNH 19335 specimens all produced distributions with a median being closer than the mode to the mean radiocarbon age, but only in LOO analyses with a strict clock, regardless of dataset. A portion of this is likely being driven by the massive disparity in the size of the 95% HPD intervals between analyses using the uncorrelated lognormal clock and the strict clock, which show an 8.44–11.02 fold increase using the full dataset and a 5.33–8.13 fold increase using only dated specimens. All three of these specimens also produce very young median and mode age estimates across all analyses, but in particular in both strict clock analyses, where the median and mode of these specimens falls firmly in the Holocene (i.e., less than ~12 kya). Two of these specimens (ISM2015-54 and ETHMNH 19335) were revealed as potentially problematic during our TempEst analyses, and all three produce fairly long branches in the maximum-likelihood phylogenies. Together, these results suggest that these values are likely being underestimated due to underlying problems with the sequences, such as DNA damage. However, as previously seen when two out of the three specimens are removed as calibration points in the analysis, the effect of these samples on the overall calibration of the phylogeny and trends between clades is fairly minor (See Appendix A).

Supplementary Table 33. Summary statistics for LOO analyses using the uncorrelated relaxed lognormal clock for with the full mastodon dataset ("all specimens), or only specimens with radiocarbon/stratigraphic/ESR dates ("dated specimens).

Specimen	Relaxed Lognormal Clock (all specimens)			Relaxed Lognormal Clock (dated specimens)		
	Median	95% HPD	Mode	Median	95% HPD	Mode
Buesching	48,820.5	[0.6 - 264,110.0]	13,562.5	27,017.0	[0.1 - 178,990.0]	7,187.5
CCM-1 V1	399,070.0	[358.1 - 740,510.0]	389,687.5	278,070.0	[10.1 - 679,510.0]	175,687.5
CCM-1 V2	417,380.0	[50,126.2 - 744,270.0]	385,437.5	296,210.0	[50,000.2 - 683,790.0]	165,562.5
DP1296	423,870.0	[35,622.3 - 780,340.0]	449,187.5	291,280.0	[38.5 - 705,410.0]	153,812.5
ISM2015-53	72,489.0	[3.3 - 305,130.0]	22,562.5	50,336.0	[3.7 - 221,980.0]	34562.5
ISM2015-54	29,225.1	[0.8 - 185,810.0]	8,187.5	14,063.0	[0.3 - 120,330.0]	3,437.5
ISM2015-58	259,060.0	[8.8 - 628,050.0]	221,062.5	220,340.0	[1.4 - 622,500.0]	141,437.5
MAS1 (NC_035800)	160,050.0	[0.9 - 487,900.0]	85,562.5	140,380.0	[9.2 - 417,420.0]	110,312.5
P12780	126,880.0	[17.7 - 449,940.0]	63,687.5	88,200.4	[6.7 - 296,550.0]	67,812.5
P14591	91,523.5	[1.9 - 401,480.0]	23,687.5	63,237.9	[3.9 - 275,500.0]	14,937.5
ETMNH 19334	160,920.0	[2.8 - 538,980.0]	40,812.5	119,080.0	[0.2 - 396,560.0]	74,937.5
ETMNH 19335	128,820.0	[0.6 - 479,540.0]	31,937.5	84,164.8	[7.3 - 351,940.0]	24,437.5
UM57705	77,457.7	[0.0 - 314,360.0]	24,187.5	58,213.5	[6.9 - 244,820.0]	28,562.5
UM58075	62,855.1	[1.6 - 281,570.0]	15,812.5	42,796.0	[0.1 - 220,420.0]	12,437.5

Supplementary Table 34. Summary statistics for LOO analyses using the strict clock for with the full mastodon dataset ("all specimens), or only specimens with radiocarbon/stratigraphic/ESR dates ("dated specimens).

Specimen	Strict Clock (all specimens)			Strict Clock (dated specimens)		
	Median	95% HPD	Mode	Median	95% HPD	Mode
Buesching	7,075.1	[0.1 - 31,302.4]	1775	6,205.6	[0.1 - 33,579.5]	1,775.0
CCM-1 V1	228,560.0	[19,043.4 - 442,320.0]	217187.5	244,800.0	[1,183.0 - 542,520.0]	221,062.5
CCM-1 V2	233,530.0	[50,206.9 - 432,080.0]	211937.5	251,570.0	[50,008.9 - 547,300.0]	201,562.5
DP1296	654,650.0	[368,240.0 - 800,000.0]	760437.5	338,400.0	[202.0 - 718,240.0]	199,437.5
ISM2015-53	61,041.3	[42.5 - 124,730.0]	56062.5	56,077.2	[39.0 - 190,180.0]	49937.5
ISM2015-54	3,802.1	[0.2 - 17,786.5]	1025	3,349.2	[0.1 - 15,877.8]	925.0
ISM2015-58	48,129.1	[3.5 - 164,410.0]	10687.5	56,358.5	[2.0 - 211,480.0]	13,562.5
MAS1 (NC_035800)	127,870.0	[39,681.7 - 243,580.0]	109937.5	126,050.0	[39,693.7 - 306,600.0]	113,062.5
P12780	101,040.0	[34,028.6 - 188,880.0]	91937.5	85,782.7	[23,562.9 - 255,900.0]	73,062.5
P14591	39,608.7	[0.3 - 118,010.0]	8625	38,157.8	[0.0 - 150,810.0]	7,812.5
ETMNH 19334	115,770.0	[5,410.1 - 226,310.0]	109187.5	123,570.0	[13,453.5 - 316,310.0]	108,812.5
ETMNH 19335	9,523.8	[1.2 - 43,501.8]	2425	8,448.2	[0.2 - 43,268.6]	1,937.5
UM57705	58,786.3	[49.3 - 128,960.0]	54187.5	58,571.7	[3.8 - 195,520.0]	50,437.5
UM58075	37,691.3	[6.7 - 105,210.0]	10125	36,408.9	[0.3 - 141,380.0]	11,687.5

Supplementary Table 35. Performance summary of LOO analyses for each of our dated specimens. The dated mean as well as the full 2 standard deviation range of each specimen is provided for easy reference. Note that Buesching was dated using stratigraphy, and produced a point estimate of 13000, but was fit with a standard deviation of 1 as in previous analyses. For each clock model, we examined whether the full 2σ range of the left out specimen was recovered in the 95% HPD interval ("Within HPD"), and how close the mode of the distribution was to the mean of the dated age of each specimen, relative to the length of the 95% HPD interval ("Rel. Dist. From Max").

Specimen	Date Mean	2σ Range	Lognormal (all)		Lognormal (dated)		Strict (all)		Strict (dated)	
			Within HPD	Rel. Dist. from Mode	Within HPD	Rel. Dist. from Mode	Within HPD	Rel. Dist. from Mode	Within HPD	Rel. Dist. from Mode
Buesching	13000	12998 – 13002	Yes	0.21 %	Yes	3.25 %	Yes	35.86 %	Yes	33.43 %
CCM-1 V1	74900	69900 - 79900	Yes	42.53 %	Yes	14.83 %	Yes	33.62 %	Yes	27.00 %
CCM-1 V2	74900	69900 - 79900	Yes	44.74 %	Yes	14.30 %	Yes	35.89 %	Yes	25.47 %
DP1296	34331.5	33834 - 34829	No	55.71 %	Yes	16.94 %	No	168.17 %	Yes	22.99 %
ISM2015-53	13111	12952 - 13270	Yes	3.10 %	Yes	9.66 %	Yes	34.45 %	Yes	19.37 %
ISM2015-54	13075.5	12883 - 13268	Yes	2.63 %	Yes	8.01 %	Yes	67.75 %	Yes	76.53 %
ISM2015-58	13505	13249 - 13761	Yes	33.05 %	Yes	20.55 %	Yes	1.71 %	Yes	0.03 %
MAS1 (NC_035800)	13410.5	13278 - 13543	Yes	14.79 %	Yes	23.22 %	No	47.34 %	No	37.34 %
P12780	13487.5	13254 - 13721	Yes	11.16 %	Yes	18.32 %	No	50.66 %	No	25.64 %
P14591	13250	13059 - 13441	Yes	2.60 %	Yes	0.61 %	Yes	3.92 %	Yes	3.61 %
ETMNH 19334	24865	24436 - 25294	Yes	2.96 %	Yes	12.63 %	Yes	38.17 %	Yes	27.72 %
ETMNH 19335	26691.5	26208 - 27175	Yes	1.09 %	Yes	0.64 %	Yes	55.78 %	Yes	57.21 %
UM57705	15298	14703 - 15893	Yes	2.83 %	Yes	5.42 %	Yes	30.17 %	Yes	17.97 %
UM58075	13861.5	13570 - 14153	Yes	0.69 %	Yes	0.65 %	Yes	3.55 %	Yes	1.54 %

Supplementary Figure 17. Posterior distributions of the clock.rate parameter for MAS1 (A) and P12780 (B) LOO analyses with a strict clock and all mastodon mitochondrial genomes, and with a strict clock using only dated mastodon mitochondrial genomes – MAS1 (C) and P12780 (D). In analyses using only dated specimens the clock.rate parameter abuts the upper bound specified on the rate prior (8×10^{-9}), suggesting the true value of this parameter might be outside the current bounds and the prior is incorrectly specified.

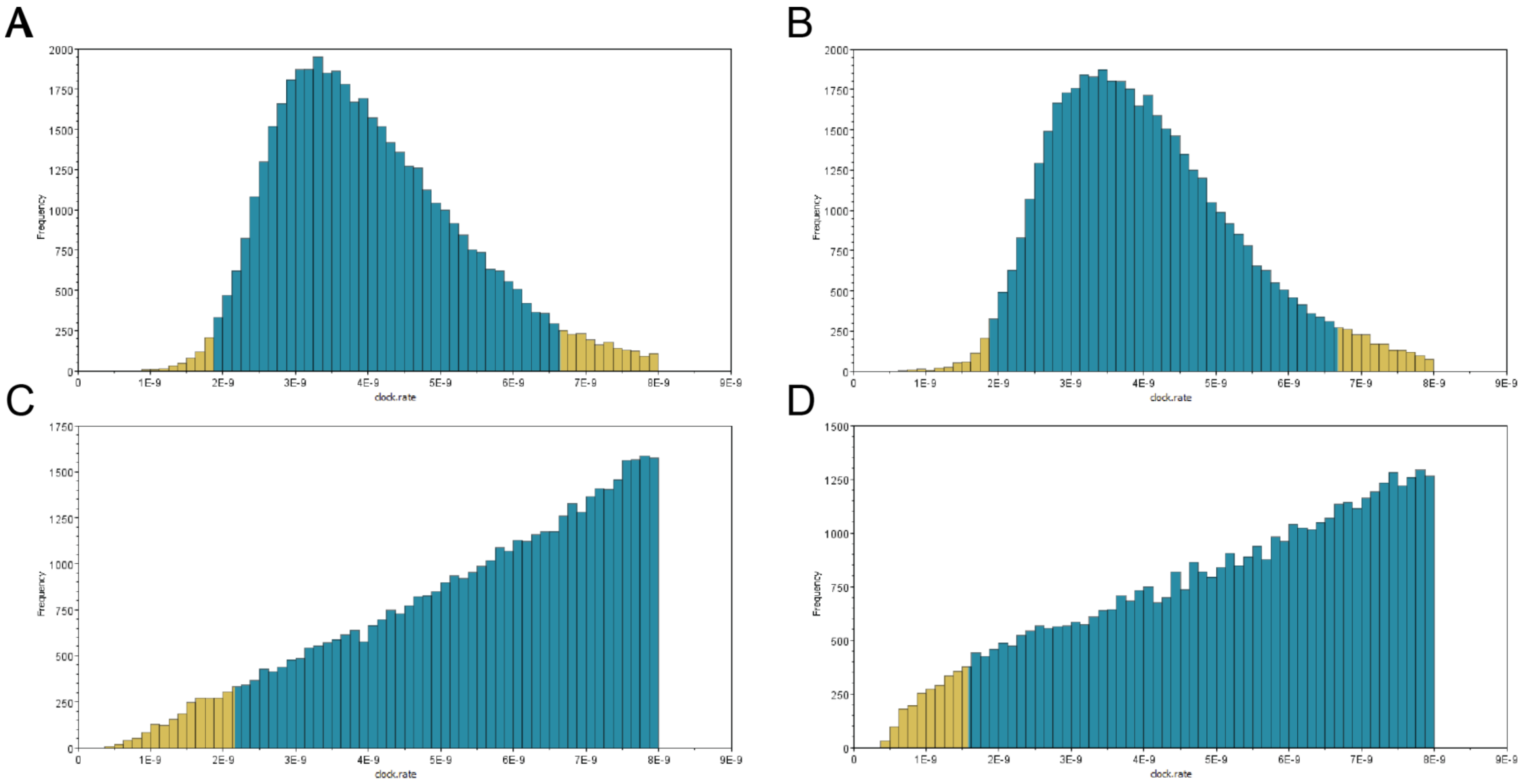

Temporal Signal Verification with BETS

As an additional means of confirming that our data contain sufficient temporal signal for dating, we employed a BETS (Bayesian Evaluation of Temporal Signal)[20] analysis on a phylogeny of our dated specimens in BEAST v1.10.5 (Prerelease #23570d1)[21]. BETS compares the fit of two models, one where specimens are treated as heterochronous (*i.e.* each with their own unique age) and one where specimens are treated as isochronous (*i.e*. all specimens are considered contemporary with no calibrating temporal information), to see whether the inclusion of temporal information improves the log marginal likelihood of the model. Three replicates were run for both the heterochronous and isochronous models (constant size coalescent prior; 50 million steps; sampling every 5000 steps) to test for convergence and reproducibility of the log marginal likelihood as estimated by generalized stepping stone (GSS) sampling[22].

All replicates generated comparable log marginal likelihood values (Supplementary Table 36). Comparison of the average log marginal likelihoods found positive support for the heterochronous model (BF = 3.30)[23], suggesting that the data contain a temporal signal.

Supplementary Table 36. BETS analysis for temporal signal of our dated specimens. Comparison between the heterochronous model (all tips have their own unique calibration) versus the isochronous model (all tips are considered contemporary).

Temporal Model	Log Marginal Likelihood				Log Bayes Factor (2 ln K)
	Rep. 1	Rep. 2	Rep. 3	Average	
Heterochronous	-25174.90	-25175.08	-25175.02	-25175.00	3.303837 (Positive)
Isochronous	-25176.74	-25176.65	-25176.56	-25176.65	

Model Testing With GSS

Given the potential problems with the clock.rate prior highlighted in the LOO analysis, we decided to reconsider the models that we were using for temporal estimation. All analyses were performed in BEAST v1.10.5 (Prerelease #23570d1) as GSS has only been implemented in BEAST v1.8.3 and onwards, and has been shown to outperform PS/SS[22].

During model testing we examined the fit of two clock models (strict and uncorrelated relaxed lognormal), three separate rate priors (the original uniform prior (Uniform [0.4×10^{-9}, 8×10^{-9}], initial = 4.2×10^{-9}), an expanded uniform prior (Uniform [0.4×10^{-9}, 8×10^{-8}], initial = 4.2×10^{-9}), and a CTMC prior), as well as four demographic priors (a constant population size fit with a lognormal prior (LogNormal [1,10], intial=8.7×10^{5}), a constant population size with a very broad (Uniform [1,1×10^{9}], intial=10^{4}) or more restricted uniform prior (Uniform [1,10^{6}], initial = 10^{4}), and a GMRF time-aware skyride demographic prior) (Supplementary Table 37). Each clock, rate, and demographic prior combination was run in duplicate (100 million generations; sampling every 10,000) and compared to check for convergence. Bayes factors were calculated using the mean log marginal likelihood of each model and interpreted using the guidelines of Kass and Raftery[23].

In general, models with a relaxed clock produced greater log marginal likelihoods over models with a strict clock. However, all of these models failed to either converge and/or be adequately sampled after 100 million steps, and all contained at least two parameters that had effective sample size (ESS) values below 200. In comparison only one strict clock model (R114) failed to produce ESS values of at least

200 for a single parameter. Likewise, relaxed clock models tended to produce very large (1.15–3.94) median estimates for the coefficient of variation, unlikely to be observed in intra-species data[24]. Together these results are highly indicative of over-parametrization and suggest that the relaxed clock is not a suitable choice for molecular clock estimation with this dataset. We note that similarly high vales for the coefficient of variation were also observed during original model-selection experiments (median range: 0.714–1.34), suggesting that the uncorrelated lognormal models used previously were also likely over-parameterized and poorly suited for the analysis of these data.

Of the strict clock models, R100_v2 (strict clock, expanded uniform rate prior, and the more restricted uniform demographic prior) had the largest log marginal likelihood. Skyride demographic models were generally not favoured relative to constant population size priors. Likewise, an expanded uniform prior on the rate generally resulted in a better fit for the data than our original uniform prior, as was suggested by the posterior distribution abutting against the upper bound. The very broad uniform prior on the constant population size resulted in a poorer fit from the data than other constant-size priors, although restricting this prior to more reasonable bounds produced positive to very strong support in its favour.

Supplementary Table 37. Model testing with GSS. Priors and their relevant parameters are provided in the run details column for each run. The log marginal likelihood for each of the two replicates for each model is provided as well as the average. Models are ordered by marginal likelihood, with the best-fitting model in bold.

Run	GSS MLE			Run Details
	Replicate 1	Replicate 2	Average	
R121	-25137.27	-25136.07	-25136.67	Uncorrelated Lognormal Clock; CTMC rate prior; Constant Pop Size Lognormal Prior (1,10)
R119_v2	-25141.06	-25135.72	-25138.39	Uncorrelated Lognormal Clock; Uniform rate prior (0.4E-9, 8E-8); Constant Pop Size Uniform Prior (1, 1E6)
R122_v2	-25140.15	-25139.84	-25140	Uncorrelated Lognormal Clock; CTMC rate prior; Constant Pop Size Uniform Prior (1, 1E6)
R119	-25144.24	-25145.74	-25144.99	Uncorrelated Lognormal Clock; Uniform rate prior (0.4E-9, 8E-8); Constant Pop Size Uniform Prior (1,1E9)
R116	-25145.22	-25145.21	-25145.21	Uncorrelated Lognormal Clock; Uniform rate prior (0.4E-9, 8E-9); Constant Pop Size Uniform Prior (1,1E9)
R116_v2	-25146.97	-25146.98	-25146.98	Uncorrelated Lognormal Clock; Uniform rate prior (0.4E-9, 8E-9); Constant Pop Size Uniform Prior (1, 1E6)
R122	-25143.28	-25158.21	-25150.74	Uncorrelated Lognormal Clock; CTMC rate prior; Constant Pop Size Uniform Prior (1,1E9)
**R110_v2**	**-25151.65**	**-25151.72**	**-25151.69**	**Strict Clock; Uniform rate prior (0.4E-9, 8E-8); Constant Pop Size Uniform Prior (1, 1E6)**

R107_v2	-25152.14	-25152.07	-25152.11	Strict Clock; Uniform rate prior (0.4E-9, 8E-9); Constant Pop Size Uniform Prior (1, 1E6)
R118	-25136.52	-25168.4	-25152.46	Uncorrelated Lognormal Clock; Uniform rate prior (0.4E-9, 8E-8); Constant Pop Size Lognormal Prior (1,10)
R113_v2	-25152.46	-25152.51	-25152.48	Strict Clock; CTMC rate prior; Constant Pop Size Uniform Prior (1, 1E6)
R109	-25153.71	-25153.81	-25153.76	Strict Clock; Uniform rate prior (0.4E-9, 8E-8); Constant Pop Size Lognormal Prior (1,10)
R113	-25153.77	-25154.21	-25153.99	Strict Clock; CTMC rate prior; Constant Pop Size Uniform Prior (1,1E9)
R115	-25152.3	-25155.73	-25154.02	Uncorrelated Lognormal Clock; Uniform rate prior (0.4E-9, 8E-9); Constant Pop Size Lognormal Prior (1,10)
R106	-25155.25	-25155.39	-25155.32	Strict Clock; Uniform rate prior (0.4E-9, 8E-9); Constant Pop Size Lognormal Prior (1,10)
R117	-25157.71	-25153.92	-25155.81	Uncorrelated Lognormal Clock; Uniform rate prior (0.4E-9, 8E-9); GMRF Time-aware Skyride
R120	-25159.11	-25156.64	-25157.88	Uncorrelated Lognormal Clock; Uniform rate prior (0.4E-9, 8E-8); GMRF Time-aware Skyride
R107	-25158.06	-25157.9	-25157.98	Strict Clock; Uniform rate prior (0.4E-9, 8E-9); Constant Pop Size Uniform Prior (1,1E9)
R110	-25158.53	-25158.46	-25158.5	Strict Clock; Uniform rate prior (0.4E-9, 8E-8); Constant Pop Size Uniform Prior (1,1E9)
R111	-25174.61	-25172.73	-25173.67	Strict Clock; Uniform rate prior (0.4E-9, 8E-8); GMRF Time-aware Skyride
R108	-25174.26	-25175.21	-25174.74	Strict Clock; Uniform rate prior (0.4E-9, 8E-9); GMRF Time-aware Skyride
R112	-25169.58	-25186.26	-25177.92	Strict Clock; CTMC rate prior; Constant Pop Size Lognormal Prior (1,10)
R114	-25128.34	-26256.55	-25692.45	Strict Clock; CTMC rate prior; GMRF Time-aware Skyride
R123	-25154.86	-1.44E+299	-7.19E+298	Uncorrelated Lognormal Clock; CTMC rate prior; GMRF Time-aware Skyride

Specimen Age Estimation

We attempted to re-estimate the ages of undated specimens in our dataset, using the new best-fitting model identified using GSS. However, due to the much lower rate estimated for datasets when the ages of all undated specimens are estimated together and those that contain only a single undated specimen, we attempted to examine the differences in specimen ages using two separate methods: a combined approach where the ages of all undated specimens were estimated together with similar non-informative priors (Joint; "JT"), and an approach where the undated specimens were analysed individually with all of the dated specimens, and then combined using the pre-estimated posteriors as priors in a final analysis (Individually Dated; "ID").

Joint analyses were conducted using BEAST 1.10.5 (Prerelease #23570d1)[21], using parameters identified by GSS model selection. The ages of undated specimens were assigned broad gamma distributions (shape = 1; scale = 200,000). Specimens with known temporal information were given point ages corresponding to the median of the estimated age, as previous work has shown that incorporating uncertainty in these dates does not substantially change the estimates of the clock rate or node times[25]. However, the use of point estimates prohibits the estimation of ages younger than the youngest specimen included in the dataset (i.e. Buesching at 13,000 kya), resulting in estimates that are relative to the age of the youngest specimen. Accordingly, we modified the bounds of undated specimens to 0–787,000 for those that are likely finite in age, and 37,000-787,000 for undated specimens with ages that are known or presumed to be non-finite. Full prior and operator information for all specimens can be found in Supplementary Table 38. Two independent chains (500 million steps; sampling every 10,000 steps) were run to monitor for convergence then combined for the final analysis, with a 10% burn-in.

Individually Dated analyses were conducted in two phases. In the first phase, the age of each undated specimen was estimated independently with only specimens of known age included in the dataset. The age of each undated specimen was assigned an identical gamma distribution and bounds as in the Joint analyses, and run in duplicate to test for convergence (100 million steps; sampling every 10,000 steps). To minimize computational time, these pre-estimate analyses were run in BEAST 1.8.2[16] as this was the most recent version of BEAST on available computational clusters.

In the second phase, the dataset was extended to include all mastodon mitochondrial genomes together. However, the broad gamma priors fit to the ages of undated specimens in the Joint analyses were instead changed to lognormal distributions based on the marginal posterior distributions obtained during each specimen's pre-estimation (Supplementary Table 38). Lognormal distributions were set using the mean and standard deviation of the combined pre-estimation runs for each individual specimen with a 10% burn-in. The full dataset was then analysed using two independent chains (500 million steps; sampling every 10,000 steps) to monitor for convergence, and then combined for the final analysis (10% burn-in).

Supplementary Table 38. Age priors for specimens of unknown age for the Joint and Individually Dated analyses. For the pre-estimation phase of the Individually Dated analyses, specimen ages were assigned identical gamma distributions as for the Joint analyses, then lognormal analyses corresponding to estimated values for the final second phase. Uniform distributions were manually specified to bound each distribution and are relative to the youngest specimen in the analysis (Buesching @13ky), such that the actual bounds represent 50–800 ky. All values in the lognormal distributions are in real space. The weight on each of the age priors was set to 5 in all analyses to allow for more efficient estimation during each run.

**Specimen**	**Joint**	**Individually Dated**
AMNH-988	Gamma:<br>- Shape: 1<br>- Scale: 200,000<br>- Offset: 0<br>Uniform:<br>- Lower: 0<br>- Upper: 787,000<br>Initial: 7,000	Lognormal:<br>- Mean: 8,343.4034<br>- Stdev: 9,840.0<br>- Offset: 0<br>Uniform:<br>- Lower: 0<br>- Upper: 787,000<br>Initial: 7,000.0
CMN-11697	Gamma:<br>- Shape: 1<br>- Scale: 200,000<br>- Offset: 0<br>Uniform:<br>- Lower: 37,000<br>- Upper: 787,000<br>Initial: 87,000	Lognormal:<br>- Mean: 97,813.5603<br>- Stdev: 54,196.3863<br>- Offset: 0<br>Uniform:<br>- Lower: 37,000<br>- Upper: 787,000<br>Initial: 81,825.4277
FAM-103291	Gamma:<br>- Shape: 1<br>- Scale: 200,000<br>- Offset: 0<br>Uniform:<br>- Lower: 37,000<br>- Upper: 787,000<br>Initial: 87,000	Lognormal:<br>- Mean: 92,166.6687<br>- Stdev: 50,781.5229<br>- Offset: 0<br>Uniform:<br>- Lower: 37,000<br>- Upper: 787,000<br>Initial: 76,986.6196
IK-01-277	Gamma:<br>- Shape: 1<br>- Scale: 200,000<br>- Offset: 0<br>Uniform:<br>- Lower: 37,000<br>- Upper: 787,000<br>Initial: 87,000	Lognormal:<br>- Mean: 94,594.7808<br>- Stdev: 51,782.7134<br>- Offset: 0<br>Uniform:<br>- Lower: 37,000<br>- Upper: 787,000<br>Initial: 79,067.855
IK-01-321	Gamma:<br>- Shape: 1<br>- Scale: 200,000<br>- Offset: 0<br>Uniform:<br>- Lower: 37,000<br>- Upper: 787,000<br>Initial: 87,000	Lognormal:<br>- Mean: 9,9026.5422<br>- Stdev: 55,361.728<br>- Offset: 0<br>Uniform:<br>- Lower: 37,000<br>- Upper: 787,000<br>Initial: 82,529.5147

IK05-3.5	Gamma:<br>- Shape: 1<br>- Scale: 200,000<br>- Offset: 0<br>Uniform:<br>- Lower: 37,000<br>- Upper: 787,000<br>Initial: 87,000	Lognormal:<br>- Mean: 198,040.0<br>- Stdev: 85,595.6954<br>- Offset: 0<br>Uniform:<br>- Lower: 37,000<br>- Upper: 787,000<br>Initial: 176,910.0
IK08-127	Gamma:<br>- Shape: 1<br>- Scale: 200,000<br>- Offset: 0<br>Uniform:<br>- Lower: 37,000<br>- Upper: 787,000<br>Initial: 87,000	Lognormal:<br>- Mean: 90,906.3939<br>- Stdev: 5,0843.1128<br>- Offset: 0<br>Uniform:<br>- Lower: 37,000<br>- Upper: 787,000<br>Initial: 75,613.7289
IK10-106	Gamma:<br>- Shape: 1<br>- Scale: 200,000<br>- Offset: 0<br>Uniform:<br>- Lower: 37,000<br>- Upper: 787,000<br>Initial: 87,000	Lognormal:<br>- Mean: 95,667.4326<br>- Stdev: 53,061.0023<br>- Offset: 0<br>Uniform:<br>- Lower: 37,000<br>- Upper: 787,000<br>Initial: 79,678.0061
IK-98-963	Gamma:<br>- Shape: 1<br>- Scale: 200,000<br>- Offset: 0<br>Uniform:<br>- Lower: 37,000<br>- Upper: 787,000<br>Initial: 87,000	Lognormal:<br>- Mean: 101,260.0<br>- Stdev: 55,535.5386<br>- Offset: 0<br>Uniform:<br>- Lower: 37,000<br>- Upper: 787,000<br>Initial: 84,734.5828
IK-99-328	Gamma:<br>- Shape: 1<br>- Scale: 200,000<br>- Offset: 0<br>Uniform:<br>- Lower: 37,000<br>- Upper: 787,000<br>Initial: 87,000	Lognormal:<br>- Mean: 195,690.0<br>- Stdev: 84,048.8303<br>- Offset: 0<br>Uniform:<br>- Lower: 37,000<br>- Upper: 787,000<br>Initial: 174,970.0
KIG12-15	Gamma:<br>- Shape: 1<br>- Scale: 200,000<br>- Offset: 0<br>Uniform:<br>- Lower: 37,000<br>- Upper: 787,000<br>Initial: 87,000	Lognormal:<br>- Mean: 102,150.0<br>- Stdev: 55,995.2183<br>- Offset: 0<br>Uniform:<br>- Lower: 37,000<br>- Upper: 787,000<br>Initial: 85,399.4858

MAY12-69	Gamma:<br>- Shape: 1<br>- Scale: 200,000<br>- Offset: 0<br>Uniform:<br>- Lower: 37,000<br>- Upper: 787,000<br>Initial: 87,000	Lognormal:<br>- Mean: 110,470.0<br>- Stdev: 61,076.5909<br>- Offset: 0<br>Uniform:<br>- Lower: 37,000<br>- Upper: 787,000<br>Initial: 92,337.6351
MAY12-70	Gamma:<br>- Shape: 1<br>- Scale: 200,000<br>- Offset: 0<br>Uniform:<br>- Lower: 37,000<br>- Upper: 787,000<br>Initial: 87,000	Lognormal:<br>- Mean: 97,963.5469<br>- Stdev: 52,908.9076<br>- Offset: 0<br>Uniform:<br>- Lower: 37,000<br>- Upper: 787,000<br>Initial: 82,770.0859
NC-009574	Gamma:<br>- Shape: 1<br>- Scale: 200,000<br>- Offset: 0<br>Uniform:<br>- Lower: 37,000<br>- Upper: 787,000<br>Initial: 87,000	Lognormal:<br>- Mean: 10,1520.0<br>- Stdev: 55,991.6314<br>- Offset: 0<br>Uniform:<br>- Lower: 37,000<br>- Upper: 787,000<br>Initial: 84,981.5814
RAM-P94.5.7	Gamma:<br>- Shape: 1<br>- Scale: 200,000<br>- Offset: 0<br>Uniform:<br>- Lower: 37,000<br>- Upper: 787,000<br>Initial: 87,000	Lognormal:<br>- Mean: 15,0170.0<br>- Stdev: 75,368.4761<br>- Offset: 0<br>Uniform:<br>- Lower: 37,000<br>- Upper: 787,000<br>Initial: 12,9230.0
RAM-P94.16.1B	Gamma:<br>- Shape: 1<br>- Scale: 200,000<br>- Offset: 0<br>Uniform:<br>- Lower: 37,000<br>- Upper: 787,000<br>Initial: 87,000	Lognormal:<br>- Mean: 93,950.5233<br>- Stdev: 52,616.508<br>- Offset: 0<br>Uniform:<br>- Lower: 37,000<br>- Upper: 787,000<br>Initial: 77,937.2918
RAM-P97.7.1	Gamma:<br>- Shape: 1<br>- Scale: 200,000<br>- Offset: 0<br>Uniform:<br>- Lower: 37,000<br>- Upper: 787,000<br>Initial: 87,000	Gamma:<br>- Shape: 1<br>- Scale: 200,000<br>- Offset: 0<br>Uniform:<br>- Lower: 37,000<br>- Upper: 787,000<br>Initial: 87,000

UAMES-7663	Gamma:<br>- Shape: 1<br>- Scale: 200,000<br>- Offset: 0<br>Uniform:<br>- Lower: 37,000<br>- Upper: 787,000<br>Initial: 87,000	Lognormal:<br>- Mean: 91,057.7442<br>- Stdev: 50,360.2238<br>- Offset: 0<br>Uniform:<br>- Lower: 37,000<br>- Upper: 787,000<br>Initial: 75,395.7648
UM13909	Gamma:<br>- Shape: 1<br>- Scale: 200,000<br>- Offset: 0<br>Uniform:<br>- Lower: 0<br>- Upper: 787,000<br>Initial: 7,000	Lognormal:<br>- Mean: 13,481.7369<br>- Stdev: 13,139.6811<br>- Offset: 0<br>Uniform:<br>- Lower: 0<br>- Upper: 787,000<br>Initial: 13,139.6811
YG26.1	Gamma:<br>- Shape: 1<br>- Scale: 200,000<br>- Offset: 0<br>Uniform:<br>- Lower: 37,000<br>- Upper: 787,000<br>Initial: 87,000	Lognormal:<br>- Mean: 93,090.842<br>- Stdev: 52,086.0704<br>- Offset: 0<br>Uniform:<br>- Lower: 37,000<br>- Upper: 787,000<br>Initial: 77,588.9719
YG43.2	Gamma:<br>- Shape: 1<br>- Scale: 200,000<br>- Offset: 0<br>Uniform:<br>- Lower: 37,000<br>- Upper: 787,000<br>Initial: 87,000	Lognormal:<br>- Mean: 98,857.533<br>- Stdev: 54,720.8737<br>- Offset: 0<br>Uniform:<br>- Lower: 37,000<br>- Upper: 787,000<br>Initial: 82,261.4387
YG50.1	Gamma:<br>- Shape: 1<br>- Scale: 200,000<br>- Offset: 0<br>Uniform:<br>- Lower: 37,000<br>- Upper: 787,000<br>Initial: 87,000	Lognormal:<br>- Mean: 85,849.6457<br>- Stdev: 47,726.8035<br>- Offset: 0<br>Uniform:<br>- Lower: 37,000<br>- Upper: 787,000<br>Initial: 70,940.333

All duplicate pre-estimation chains converged, and after combining both replicates the age of each undated specimen was estimated with a minimum effective sample size of 3126 (RAM P97.7.1). The posterior probability age distributions were also visually inspected to choose prior distributions that would most accurately model the pre-estimated posteriors. In almost all cases a lognormal distribution was chosen with a mean and standard deviation chosen to match the posterior probability distribution of the pre-estimated age (Supplementary Tables 38 and 39). In the case of RAM P97.7.1 the pre-

estimated age posterior distribution contained a long tail spanning almost the entire 750 ky range of the age prior. While the distribution was primarily concentrated at younger age estimates, it also contained a smaller increase in probability mass towards the end of the tail. Due to the difficulty in modelling this specimen's posterior probability distribution, we instead replaced it with the original gamma distribution as the prior in the combined Individually Dated analysis.

Duplicate chains of the Joint and Individually Dated analyses were run for 500 million steps to allow for sufficient sampling of the ages of the undated specimens. In both analyses, duplicate chains appeared to converge and were combined for final analysis. All parameters in the combined Joint and Individually Dated analyses were well estimated, with minimum ESS values of 1195 (JT; Supplementary Table 40) and 1105 (ID; Supplementary Table 41).

In line with earlier observations during the LOO experiments, the two models produced different estimates of the clock.rate parameter, with the median of the Individually Dated analysis estimated at a rate 2.29-fold higher than in the Joint analysis. This probably mostly accounts for the shift towards younger ages observed in the Individually Dated analysis. Despite this, both analyses produced very similar patterns in the age distributions of undated samples within and between clades.

Clade Y mastodons produced age estimates centred on the MIS 5 interglacial (Supplementary Table 42). This was true in both analyses with the exception of specimen RAM P94.16.1B, which was estimated with a median age within the MIS 5 interglacial in the Individually Dated (Supplementary Figs. 18 and 19) analysis, but within the MIS 7 interglacial in the Joint analysis (Supplementary Figs. 20 and 21). However, the 95% HPD posterior probability intervals for all mastodons in Clade Y overlap with one another in both analyses, which makes the association of RAM P94.16.1B with a separate glaciation uncertain.

In comparison to Clade Y, mastodons in Clade A produced older posterior age estimates. In line with patterns observed in the clock.rate parameter, the 95% HPD intervals varied between the two analyses, with the Joint analysis producing older estimates (95% HPD – IK-99-328: 329–800 ky; IK05-3.5: 292–784 ky) in comparison to the Individually Dated analysis (95% HPD – IK-99-328: 152–410 ky; IK05-3.5: 142–397 ky). Within each analysis, the distribution of the 95% HPD intervals for Clade A are non-overlapping with mastodons in Clade Y, with the exception of RAM P94.16.1B, which overlaps IK05-3.5 by 19 ky in the Joint analysis, and both specimens in the Individually Dated analysis by 11 ky (IK-99-328) and 21 ky (IK05-3.5). However, this overlap is minimal in comparison to the full range of the 95% HPD intervals estimated for specimens in Clade A. Combined with the independent phylogenetic positions of Clades A and Y, this is strong evidence suggesting that these two clades represent separate and asynchronous expansions of mastodons into eastern Beringia likely in response to interglacial warming.

Undated mastodons in Clade G were estimated with young median posterior probability ages in both analyses, as would expected given the ages of dated mastodons in Clade G. However, we do note that the posterior probability distributions for each specimen's age in both analyses have a large amount of probability mass towards younger values and abut the lower bound at 13 ky. This suggests that the two undated specimens in this clade may be younger than 13 ky.

Specimen RAM P94.5.7 produced 95% HPD intervals in the two analyses very similar to those of mastodons in Clade A. Like mastodons in Clade A these intervals are wide and span multiple glacial

and interglacial periods, making it difficult to associate the providence of this specimen to any one time period.

The estimated age of specimen RAM P97.7.1 resulted in very wide 95% HPD intervals. The mode of the posterior distribution in both analyses was found within the MIS 5 interglacial, but the wide nature of the 95% HPD interval around this estimate makes this association uncertain. A portion of this uncertainty can probably ascribed to the deep divergence of this specimen from its sister lineage (DP 1296) and its position in the basal-most clade in our phylogeny, far away from most other calibration points.

Supplementary Table 39. Specimen pre-estimate values obtained as part of the Individually Dated analyses. Each undated specimen was analysed independently of other undated specimens, using all 13 specimens of known temporal age to calibrate the rate across the phylogeny. The mean and standard deviation from the output analysis was used to fit lognormal distributions as priors for each undated specimen in the final Individually Dated analysis.

	AMNH-988	CMN-11697	FAM-103291	IK-01-277	IK-01-321	IK-98-963	IK-99-328	IK05-3.5
Mean	8343.403	97813.56	92166.67	94594.78	99026.5422	1.01E+05	1.96E+05	1.98E+05
Stdev	9839.907	54196.39	50781.52	51782.71	55361.728	55535.5386	84048.8303	85595.6954
Median	5233.54	81825.43	76986.62	79067.86	82529.5147	84734.5828	1.75E+05	1.77E+05
95% HPD	[5.05E-01, 2.70E+04]	[3.70E+04, 2.08E+05]	[3.70E+04, 1.95E+05]	[3.70E+04, 1.99E+05]	[3.70E+04, 2.13E+05]	[3.71E+04, 2.15E+05]	[7.85E+04, 3.67E+05]	[7.68E+04, 3.74E+05]

	IK08-127	IK10-106	KIG12-15	MAY12-69	MAY12-70	NC-009574	RAM-P94.16.1B	RAM-P94.5.7
Mean	90906.3939	95667.4326	1.02E+05	1.10E+05	97963.5469	1.02E+05	93950.5233	1.50E+05
Stdev	50843.1128	53061.0023	55995.2183	61076.5909	52908.9076	55991.6314	52616.508	75368.4761
Median	75613.7289	79678.0061	85399.4858	92337.6351	82770.0859	84981.5814	77937.2918	1.29E+05
95% HPD	[3.70E+04, 1.92E+05]	[3.70E+04, 2.05E+05]	[3.71E+04, 2.17E+05]	[3.71E+04, 2.36E+05]	[3.70E+04, 2.06E+05]	[3.71E+04, 2.15E+05]	[3.70E+04, 2.02E+05]	[5.55E+04, 3.04E+05]

	RAM-P97.7.1	UAMES-7663	UM13909	YG26.1	YG43.2	YG50.1
Mean	2.63E+05	91057.7442	13481.7369	93090.842	98857.533	85849.6457
Stdev	2.38E+05	50360.2238	13139.6811	52086.0704	54720.8737	47726.8035
Median	1.26E+05	75398.7648	9568.3253	77588.9719	82261.4387	70940.333
95% HPD	[3.70E+04, 7.03E+05]	[3.70E+04, 1.92E+05]	[4.72E+00, 3.96E+04]	[3.70E+04, 1.98E+05]	[3.70E+04, 2.12E+05]	[3.70E+04, 1.82E+05]

Supplementary Table 40. Data table of all Joint parameters, once both individual chains were combined.

	joint	prior	likelihood	treeModel.rootHeight	age(root)	treeLength	Tmrca (unknowns)	Age (unknowns)
Mean	-28369.9	-1084.42	-27285.5	3.03E+06	3.02E+06	1.24E+07	3.03E+06	3.02E+06
Stderr of mean	0.1962	0.1916	0.0402	10083	10083	42144.92	10082.8	10082.8
Stdev	12.355	10.3891	5.6945	6.31E+05	6.31E+05	2.62E+06	6.31E+05	6.31E+05
Variance	152.6463	107.9339	32.4269	3.98E+11	3.98E+11	6.88E+12	3.98E+11	3.98E+11
Median	-28370.4	-1085.2	-27285.2	3.02E+06	3.00E+06	1.23E+07	3.02E+06	3.00E+06
Value range	[-28424.092, -28298.4506]	[-1121.7839, -1014.5672]	[-27314.9609, -27265.7757]	[8.0892E5, 6.372E6]	[7.9592E5, 6.359E6]	[2.8961E6, 2.5868E7]	[8.0892E5, 6.372E6]	[7.9592E5, 6.359E6]
Geo. mean	n/a	n/a	n/a	2.97E+06	2.95E+06	1.21E+07	2.97E+06	2.95E+06
95% HPD interval	[-28394.0289, -28345.3906]	[-1103.9463, -1063.1311]	[-27296.7143, -27274.5603]	[1.8288E6, 4.2815E6]	[1.8158E6, 4.2685E6]	[7.2922E6, 1.7518E7]	[1.8288E6, 4.2815E6]	[1.8158E6, 4.2685E6]
ACT	2.27E+05	3.06E+05	44816	2.30E+05	2.30E+05	2.32E+05	2.30E+05	2.30E+05
ESS	3964.8	2940.5	20082.6	3914.1	3914.1	3873	3914.2	3914.2
# samples	90002	90002	90002	90002	90002	90002	90002	90002

	constant.popSize	kappa	alpha	clock.rate	meanRate	age(AMNH-988_20000)	age(CMN-11697_100000)	age(FAM-103291_100000)
Mean	7.64E+05	47.2435	0.0553	4.59E-09	4.59E-09	20512.53	1.21E+05	1.03E+05
Stderr of mean	2078.241	0.0272	1.25E-04	2.02E-11	2.02E-11	86.202	496.6546	377.6158
Stdev	1.58E+05	8.1633	0.0371	1.11E-09	1.11E-09	18740.89	43971.2	41823.45
Variance	2.50E+10	66.6388	1.38E-03	1.24E-18	1.24E-18	3.51E+08	1.93E+09	1.75E+09
Median	7.85E+05	46.3871	0.0496	4.39E-09	4.39E-09	15088.65	1.16E+05	96574.55
Value range	[90833.3073, 1E6]	[22.476, 104.8331]	[2.791E-3, 0.3704]	[2.043E-9, 2.0456E-8]	[2.043E-9, 2.0456E-8]	[0.0267, 1.8019E5]	[37022.604, 3.7859E5]	[37000.2303, 3.6709E5]
Geo. mean	7.45E+05	46.5651	0.0414	4.47E-09	4.47E-09	12036.29	1.13E+05	94346.68
95% HPD interval	[4.7398E5, 1E6]	[32.523, 63.5836]	[2.7925E-3, 0.1239]	[2.8255E-9, 6.8034E-9]	[2.8255E-9, 6.8034E-9]	[0.0267, 57943.4349]	[42308.2435, 2.056E5]	[37000.2303, 1.8012E5]
ACT	1.56E+05	10000	10286.55	2.98E+05	2.98E+05	19041.96	1.15E+05	73369.82
ESS	5786.5	90002	87494.9	3022.8	3022.8	47265.1	7838.3	12266.9
# samples	90002	90002	90002	90002	90002	90002	90002	90002

Supplementary Table 40. Continued.

	age(IK-01-277_100000)	age(IK-01-321_100000)	age(IK-98-963_100000)	age(IK-99-328_100000)	age(IK05-3.5_100000)	age(IK08-127_100000)	age(IK10-106_100000)	age(KIG12-15_100000)
Mean	1.11E+05	1.22E+05	1.21E+05	5.61E+05	5.33E+05	1.19E+05	1.11E+05	1.21E+05
Stderr of mean	423.4123	507.3632	481.0199	3933.753	3884.158	472.6924	425.8751	483.1047
Stdev	43054.5	44016.34	43718.26	1.36E+05	1.36E+05	43678.07	42805.94	43563.69
Variance	1.85E+09	1.94E+09	1.91E+09	1.85E+10	1.85E+10	1.91E+09	1.83E+09	1.90E+09
Median	1.06E+05	1.17E+05	1.16E+05	5.73E+05	5.45E+05	1.14E+05	1.06E+05	1.16E+05
Value range	[37003.1463, 3.572E5]	[37012.4336, 3.6392E5]	[37023.5851, 3.8603E5]	[46022.2321, 7.87E5]	[37798.4397, 7.8698E5]	[37022.3624, 3.5659E5]	[37001.436, 3.5883E5]	[37007.3102, 4.1302E5]
Geo. mean	1.03E+05	1.14E+05	1.14E+05	5.41E+05	5.12E+05	1.11E+05	1.03E+05	1.14E+05
95% HPD interval	[37194.1949, 1.9022E5]	[42151.1137, 2.0574E5]	[43626.2202, 2.0635E5]	[3.1629E5, 7.87E5]	[2.7896E5, 7.7093E5]	[38880.5692, 1.9987E5]	[37001.436, 1.8946E5]	[42081.8843, 2.0428E5]
ACT	87045.57	1.20E+05	1.09E+05	7.53E+05	7.32E+05	1.05E+05	89086.82	1.11E+05
ESS	10339.6	7526.4	8260.3	1195.9	1228.7	8538.2	10102.7	8131.3
# samples	90002	90002	90002	90002	90002	90002	90002	90002

	age(MAY12-69_100000)	age(MAY12-70_100000)	age(NC-009574_100000)	age(RAM-P94.16.1B_100000)	age(RAM-P94.5.7_100000)	age(RAM-P97.7.1_100000)	age(UAMES-7663_100000)	age(UM13909_20000)
Mean	1.21E+05	1.22E+05	1.22E+05	1.99E+05	4.66E+05	1.94E+05	1.10E+05	34124.22
Stderr of mean	483.6294	545.4863	492.5869	686.6367	1484.711	616.9746	417.4028	135.8273
Stdev	43590.61	43786.88	43657.99	49720.59	1.06E+05	1.30E+05	43146.94	24829.82
Variance	1.90E+09	1.92E+09	1.91E+09	2.47E+09	1.12E+10	1.70E+10	1.86E+09	6.17E+08
Median	1.16E+05	1.17E+05	1.16E+05	1.95E+05	4.61E+05	1.64E+05	1.05E+05	29771.26
Value range	[37013.4526, 3.5682E5]	[37004.3902, 3.7225E5]	[37047.8353, 3.693E5]	[37024.6859, 4.7176E5]	[1.1858E5, 7.8693E5]	[37005.9657, 7.8695E5]	[37005.1302, 3.6663E5]	[0.0619, 1.6817E5]
Geo. mean	1.13E+05	1.14E+05	1.14E+05	1.92E+05	4.53E+05	1.58E+05	1.02E+05	23002.77
95% HPD interval	[41322.9499, 2.029E5]	[42080.8022, 2.0478E5]	[41966.8411, 2.0424E5]	[1.0581E5, 2.9843E5]	[2.606E5, 6.7361E5]	[37005.9657, 4.5404E5]	[37005.1302, 1.8962E5]	[0.0619, 80737.9399]
ACT	1.11E+05	1.40E+05	1.15E+05	1.72E+05	1.77E+05	20198.73	84230.14	26932.94
ESS	8123.7	6443.4	7855.2	5243.4	5081.8	44558.2	10685.2	33417.1
# samples	90002	90002	90002	90002	90002	90002	90002	90002

Supplementary Table 40. Continued.

	age(YG26.1_100000)	age(YG43.2_100000)	age(YG50.1_100000)	treeLikelihood	branchRates	coalescent
Mean	1.03E+05	1.22E+05	91993.39	-27285.5	0	-495.085
Stderr of mean	371.9327	486.1233	320.6158	0.0402	0	0.1415
Stdev	41629.71	43647.53	39109.07	5.6945	0	8.6234
Variance	1.73E+09	1.91E+09	1.53E+09	32.4269	0	74.3636
Median	96913.49	1.17E+05	84992.14	-27285.2	0	-495.718
Value range	[37001.5074, 3.6767E5]	[37010.1898, 3.9417E5]	[37000.4452, 3.6953E5]	[-27314.9609, -27265.7757]	[0, 0]	[-529.8134, -432.6619]
Geo. mean	94827.12	1.14E+05	84383.67	n/a	n/a	n/a
95% HPD interval	[37001.5074, 1.802E5]	[42573.2182, 2.0527E5]	[37000.4452, 1.6652E5]	[-27296.7143, -27274.5603]	n/a	[-511.2314, -477.4629]
ACT	71842.1	1.12E+05	60488.4	44816	n/a	2.42E+05
ESS	12527.8	8061.6	14879.2	20082.6	n/a	3713
# samples	90002	90002	90002	90002	90002	90002

Supplementary Table 41. MCMC data table of all Individually Dated parameters. Values are after both replicate chains were combined.

	joint	prior	likelihood	treeModel. rootHeight	age(root)	treeLength	Tmrca (unknowns)	Age (unknowns)
Mean	-28308.9022	-1023.4864	-27285.4158	1.51E+06	1.49E+06	5.71E+06	1.51E+06	1.49E+06
Stderr of mean	0.3607	0.3716	0.0407	7845.8427	7845.8427	38415.8257	7845.4495	7845.4495
Stdev	16.816	15.6758	5.8111	4.09E+05	4.09E+05	1.82E+06	4.09E+05	4.09E+05
Variance	282.778	245.7301	33.7685	1.68E+11	1.68E+11	3.32E+12	1.68E+11	1.68E+11
Median	-28308.3483	-1023.3632	-27285.2018	1.43E+06	1.42E+06	5.39E+06	1.43E+06	1.42E+06
Value range	[-28379.7647, -28247.5191]	[-1086.4188, -968.132]	[-27317.4257, -27264.2237]	[5.8683E5, 4.3581E6]	[5.7383E5, 4.3451E6]	[1.7491E6, 1.7616E7]	[5.8683E5, 4.3581E6]	[5.7383E5, 4.3451E6]
Geo. mean	n/a	n/a	n/a	1.46E+06	1.44E+06	5.45E+06	1.46E+06	1.44E+06
95% HPD interval	[-28341.454, -28275.8529]	[-1054.0993, -992.8452]	[-27296.8811, -27274.2516]	[8.4035E5, 2.3508E6]	[8.2735E5, 2.3378E6]	[2.743E6, 9.3904E6]	[8.4035E5, 2.3508E6]	[8.2735E5, 2.3378E6]
ACT	4.14E+05	5.06E+05	44179.878	3.31E+05	3.31E+05	4.00E+05	3.31E+05	3.31E+05
ESS	2173.9	1779.5	20371.7	2722	2722	2251.7	2722.1	2722.1
# samples	90002	90002	90002	90002	90002	90002	90002	90002

Supplementary Table 41. Continued.

	constant.popSize	kappa	alpha	clock.rate	meanRate	age(AMNH-988_20000)	age(CMN-11697_100000)	age(FAM-103291_100000)
Mean	3.43E+05	47.2738	0.0546	1.04E-08	1.04E-08	5121.0024	77823.5491	68541.9115
Stderr of mean	2960.0433	0.0271	1.27E-04	7.33E-11	7.33E-11	15.6181	174.1089	124.9748
Stdev	1.54E+05	8.1256	0.0367	3.18E-09	3.18E-09	4299.6467	16607.5151	16187.3622
Variance	2.36E+10	66.0262	1.34E-03	1.01E-17	1.01E-17	1.85E+07	2.76E+08	2.62E+08
Median	3.14E+05	46.4517	0.0489	1.01E-08	1.01E-08	3890.5588	76471.5698	67196.9305
Value range	[56049.9105, 9.9989E5]	[22.3632, 114.0141]	[2.7911E-3, 0.3003]	[3.0897E-9, 3.1507E-8]	[3.0897E-9, 3.1507E-8]	[102.3519, 54447.4349]	[37002.4235, 1.9601E5]	[37000.3073, 1.8185E5]
Geo. mean	3.12E+05	46.6021	0.0409	9.95E-09	9.95E-09	3779.2346	76083.6373	66677.2533
95% HPD interval	[96773.3031, 6.5379E5]	[32.7974, 63.7195]	[2.7911E-3, 0.1223]	[4.7529E-9, 1.6709E-8]	[4.7529E-9, 1.6709E-8]	[244.8319, 13503.8058]	[47349.3829, 1.115E5]	[37957.4599, 98120.5043]
ACT	3.34E+05	10000	10723.7199	4.78E+05	4.78E+05	11875.447	98921.2827	53647.555
ESS	2694.2	90002	83928	1883.4	1883.4	75788.3	9098.3	16776.5
# samples	90002	90002	90002	90002	90002	90002	90002	90002

	age(IK-01-277_100000)	age(IK-01-321_100000)	age(IK-98-963_100000)	age(IK-99-328_100000)	age(IK05-3.5_100000)	age(IK08-127_100000)	age(IK10-106_100000)	age(KIG12-15_100000)
Mean	73003.64	78136.63	78146.95	2.60E+05	2.48E+05	76178.98	73260.08	78481.27
Stderr of mean	151.0912	185.6613	177.9831	2038.907	1961.431	164.7579	144.3621	188.4482
Stdev	16596.12	16774.14	16577.27	67801.28	66451.09	16736.09	16456.51	16818.92
Variance	2.75E+08	2.81E+08	2.75E+08	4.60E+09	4.42E+09	2.80E+08	2.71E+08	2.83E+08
Median	71752.26	76742.38	76782.23	2.54E+05	2.41E+05	74918.06	71993.1	77056
Value range	[37010.7314, 1.9734E5]	[37011.2788, 1.9499E5]	[37005.8054, 1.8929E5]	[77964.1438, 6.0365E5]	[68362.845, 5.8381E5]	[37022.7658, 1.8406E5]	[37012.8929, 1.8838E5]	[37001.9374, 1.8349E5]
Geo. mean	71147.91	76369.53	76416.14	2.52E+05	2.39E+05	74366.56	71439.68	76714.11
95% HPD interval	[41320.8996, 1.0489E5]	[46869.638, 1.1154E5]	[46585.476, 1.1082E5]	[1.3872E5, 3.9723E5]	[1.2937E5, 3.8382E5]	[44544.606, 1.0921E5]	[41883.6191, 1.0503E5]	[46428.3234, 1.1129E5]
ACT	74597.16	1.10E+05	1.04E+05	8.14E+05	7.84E+05	87225.06	69260.86	1.13E+05
ESS	12065.1	8162.7	8674.9	1105.8	1147.8	10318.4	12994.6	7965.4
# samples	90002	90002	90002	90002	90002	90002	90002	90002

Supplementary Table 41. Continued.

	age(MAY12-69_100000)	age(MAY12-70_100000)	age(NC-009574_100000)	age(RAM-P94.16.1B_100000)	age(RAM-P94.5.7_100000)	age(RAM-P97.7.1_100000)	age(UAMES-7663_100000)	age(UM13909_20000)
Mean	78967.93	78111.99	78460.04	1.06E+05	2.18E+05	3.15E+05	72253.66	10320.74
Stderr of mean	189.9572	187.1016	192.8309	343.5448	1219.051	3923.222	140.5787	30.8279
Stdev	16820.29	16660.08	16773.65	21229.72	62565.06	2.53E+05	16546.86	7345.857
Variance	2.83E+08	2.78E+08	2.81E+08	4.51E+08	3.91E+09	6.39E+10	2.74E+08	5.40E+07
Median	77532.58	76701.29	77107.86	1.04E+05	2.08E+05	1.87E+05	71029.31	8338.976
Value range	[37006.3591, 1.9836E5]	[37031.6802, 2.0235E5]	[37028.306, 2.0948E5]	[37296.121, 2.608E5]	[72892.4779, 7.0775E5]	[37000.2575, 7.8699E5]	[37004.9451, 1.8576E5]	[269.258, 83673.928]
Geo. mean	77211.08	76368.95	76699.81	1.04E+05	2.10E+05	2.12E+05	70392.84	8137.039
95% HPD interval	[46924.2925, 1.1184E5]	[46224.4109, 1.1056E5]	[47081.709, 1.1164E5]	[68535.9666, 1.5002E5]	[1.1474E5, 3.4562E5]	[37008.218, 7.5009E5]	[40412.1537, 1.0347E5]	[901.0853, 25247.8286]
ACT	1.15E+05	1.14E+05	1.19E+05	2.36E+05	3.42E+05	2.17E+05	64962.82	15851.07
ESS	7840.6	7928.6	7566.5	3818.7	2634	4150.6	13854.4	56779.7
# samples	90002	90002	90002	90002	90002	90002	90002	90002

	age(YG26.1_100000)	age(YG43.2_100000)	age(YG50.1_100000)	treeLikelihood	branchRates	coalescent
Mean	68779.16	78231.04	62558.79	-27285.4	0	-463.134
Stderr of mean	125.5006	179.4438	102.6422	0.0407	0	0.3133
Stdev	16133.2	16779.96	15339.33	5.8111	0	13.8518
Variance	2.60E+08	2.82E+08	2.35E+08	33.7685	0	191.8723
Median	67451.01	76797.92	60749.87	-27285.2	0	-463.395
Value range	[37006.4027, 1.6112E5]	[37024.9737, 2.029E5]	[37000.1752, 1.8158E5]	[-27317.4257, -27264.2237]	[0, 0]	[-513.7278, -410.6177]
Geo. mean	66932.63	76469.01	60773.93	n/a	n/a	n/a
95% HPD interval	[37546.2613, 97832.0975]	[46562.5923, 1.1126E5]	[37001.9627, 90473.1954]	[-27296.8811, -27274.2516]	n/a	[-489.7663, -435.2531]
ACT	54463.69	1.03E+05	40299.11	44179.88	n/a	4.60E+05
ESS	16525.1	8744.2	22333.5	20371.7	n/a	1954.8
# samples	90002	90002	90002	90002	90002	90002

Supplementary Table 42. Median, mode, and the 95% HPD interval for the final specimen dating analyses in the Joint and Individually Dated analyses. Note that all ages are relative to the youngest specimen (Buesching – 13kya = t_0).

Analysis		AMNH-988	CMN-11697	FAM-103291	IK-01-277	IK-01-321	IK-98-963	IK-99-328	IK05-3.5
Joint	Median	15088.6456	1.16E+05	96574.5474	1.06E+05	1.17E+05	1.16E+05	5.73E+05	5.45E+05
	Mode	3.73E+03	1.06E+05	8.65E+04	9.68E+04	1.07E+05	1.08E+05	5.87E+05	5.64E+05
	95% HPD	[2.67E-02, 5.79E+04]	[4.23E+04, 2.06E+05]	[3.70E+04, 1.80E+05]	[3.72E+04, 1.90E+05]	[4.22E+04, 2.06E+05]	[4.36E+04, 2.06E+05]	[3.16E+05, 7.87E+05]	[2.79E+05, 7.71E+05]
Individually Dated	Median	3.89E+03	7.65E+04	6.72E+04	7.18E+04	7.67E+04	7.68E+04	2.54E+05	2.41E+05
	Mode	2.14E+03	7.54E+04	6.58E+04	7.02E+04	7.43E+04	7.32E+04	2.46E+05	2.27E+05
	95% HPD	[2.45E+02, 1.35E+04]	[4.73E+04, 1.12E+05]	[3.80E+04, 9.81E+04]	[4.13E+04, 1.05E+05]	[4.69E+04, 1.12E+05]	[4.66E+04, 1.11E+05]	[1.39E+05, 3.97E+05]	[1.29E+05, 3.84E+05]

Analysis		IK08-127	IK10-106	KIG12-15	MAY12-69	MAY12-70	NC-009574	RAM-P94.16.1B	RAM-P94.5.7
Joint	Median	1.14E+05	1.06E+05	1.16E+05	1.16E+05	1.17E+05	1.16E+05	1.95E+05	4.61E+05
	Mode	1.02E+05	9.73E+04	1.07E+05	1.05E+05	1.03E+05	1.07E+05	1.86E+05	4.54E+05
	95% HPD	[3.89E+04, 2.00E+05]	[3.70E+04, 1.89E+05]	[4.21E+04, 2.04E+05]	[4.13E+04, 2.03E+05]	[4.21E+04, 2.05E+05]	[4.20E+04, 2.04E+05]	[1.06E+05, 2.98E+05]	[2.61E+05, 6.74E+05]
Individually Dated	Median	7.49E+04	7.20E+04	7.71E+04	7.75E+04	7.67E+04	7.71E+04	1.04E+05	2.08E+05
	Mode	7.34E+04	7.08E+04	7.46E+04	7.42E+04	7.45E+04	7.40E+04	1.00E+05	1.92E+05
	95% HPD	[4.45E+04, 1.09E+05]	[4.19E+04, 1.05E+05]	[4.64E+04, 1.11E+05]	[4.69E+04, 1.12E+05]	[4.62E+04, 1.11E+05]	[4.71E+04, 1.12E+05]	[6.85E+04, 1.50E+05]	[1.15E+05, 3.46E+05]

Analysis		RAM-P97.7.1	UAMES-7663	UM13909	YG26.1	YG43.2	YG50.1
Joint	Median	1.64E+05	1.05E+05	29771.2595	96913.4883	1.17E+05	84992.1364
	Mode	9.01E+04	9.67E+04	7.08E+03	8.44E+04	1.09E+05	6.67E+04
	95% HPD	[3.70E+04, 4.54E+05]	[3.70E+04, 1.90E+05]	[6.19E-02, 8.07E+04]	[3.70E+04, 1.80E+05]	[4.26E+04, 2.05E+05]	[3.70E+04, 1.67E+05]
Individually Dated	Median	1.87E+05	7.10E+04	8.34E+03	6.75E+04	7.68E+04	6.07E+04
	Mode	8.72E+04	6.98E+04	5.09E+03	6.67E+04	7.42E+04	5.62E+04
	95% HPD	[3.70E+04, 7.50E+05]	[4.04E+04, 1.03E+05]	[9.01E+02, 2.52E+04]	[3.75E+04, 9.78E+04]	[4.66E+04, 1.11E+05]	[3.70E+04, 9.05E+04]

Supplementary Figure 18. KDE plot illustrating the posterior probability of the ages of all undated specimens in the final Individually Dated analysis (when all undated specimens were combined into a single analysis). Note that all ages are relative to the youngest specimen (Buesching – 13kya = t_0).

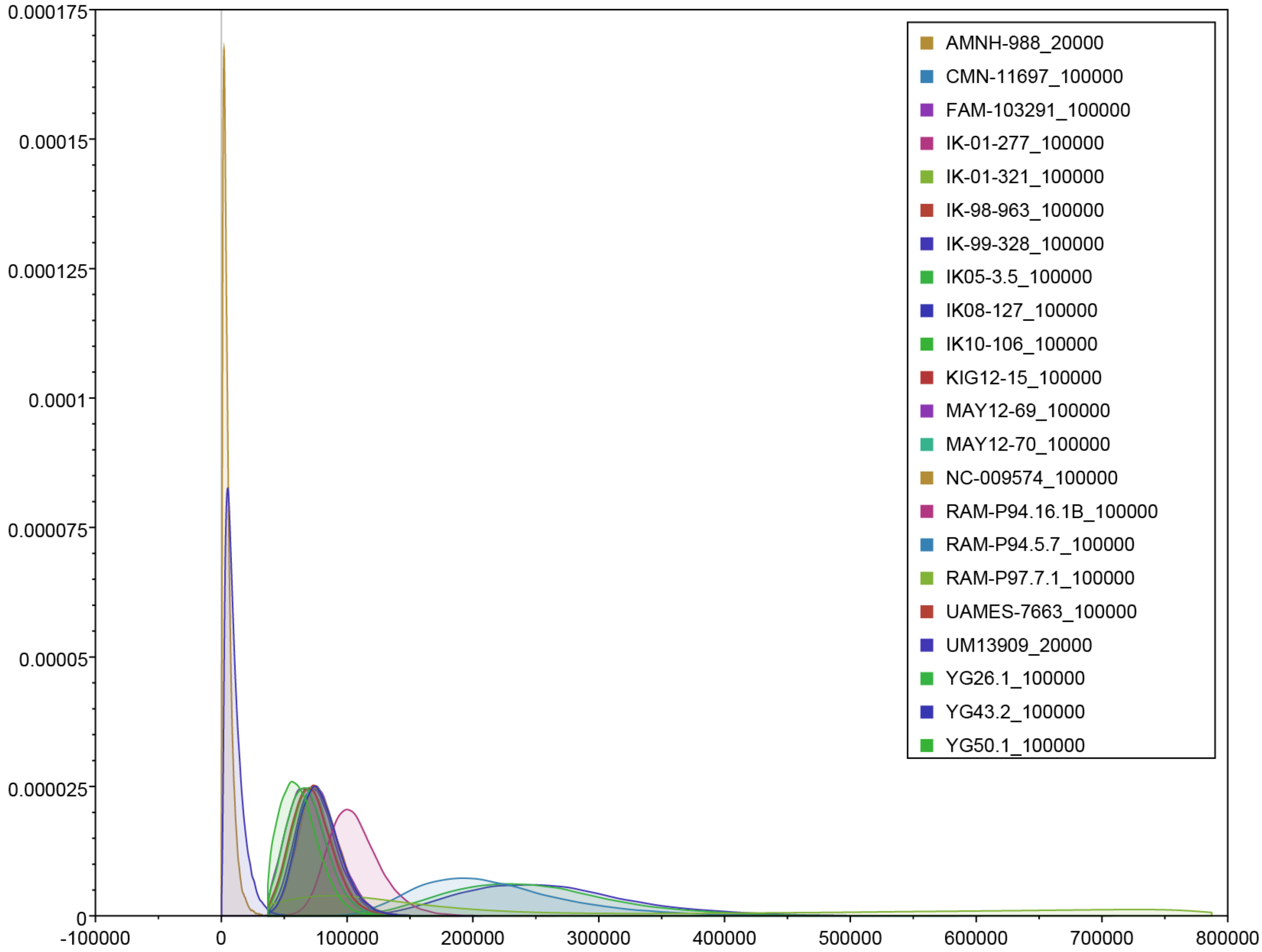

Supplementary Figure 19. Violin plot illustrating the posterior probability of the ages of all undated specimens in the final Individually Dated analysis (when all undated specimens were combined into a single analysis). Note that all ages are relative to the youngest specimen (Buesching – 13kya = t_0).

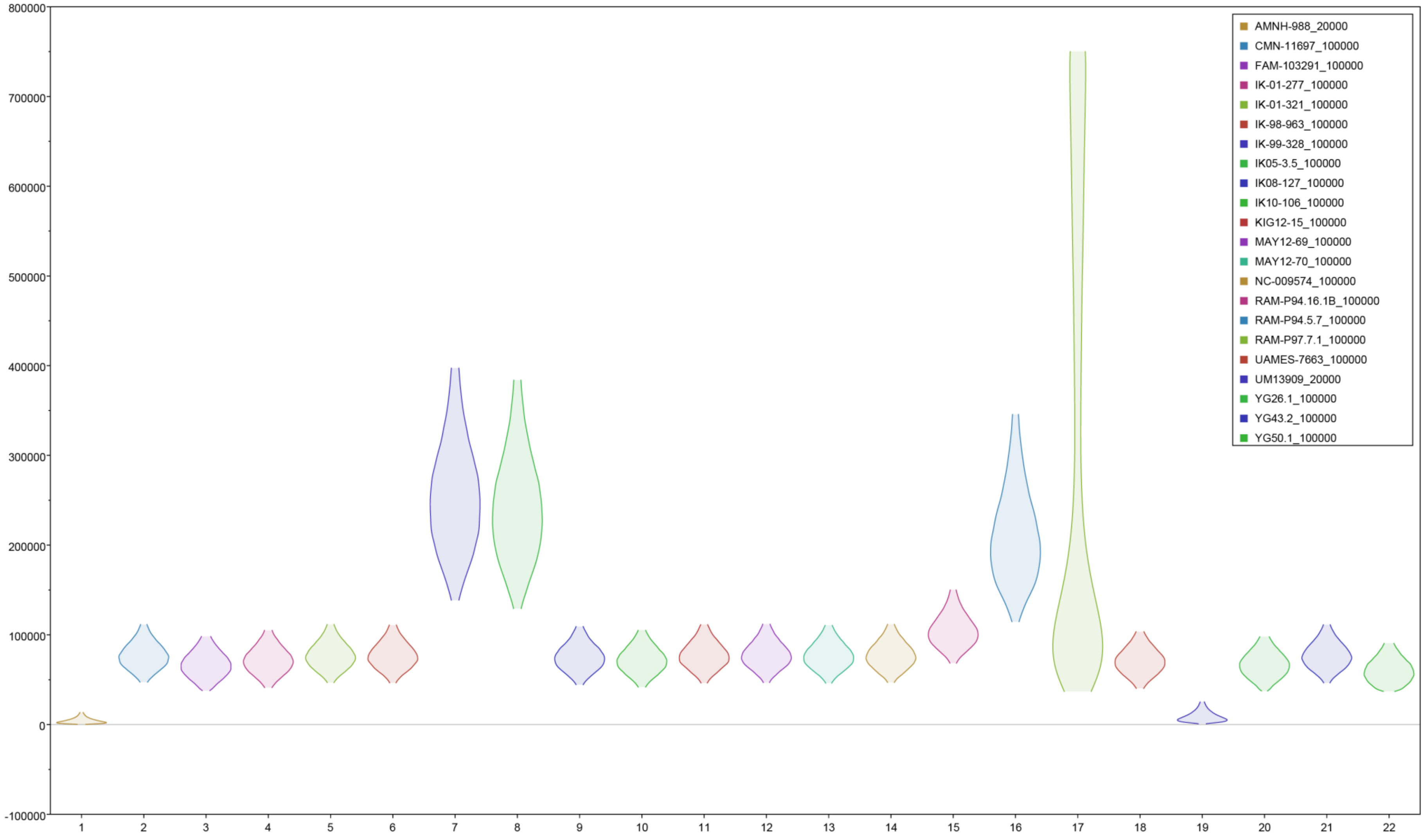

Supplementary Figure 20. KDE plot illustrating the posterior probability of the ages of all undated specimens in the Joint analysis. Note that all ages are relative to the youngest specimen (Buesching – 13kya = t_0).

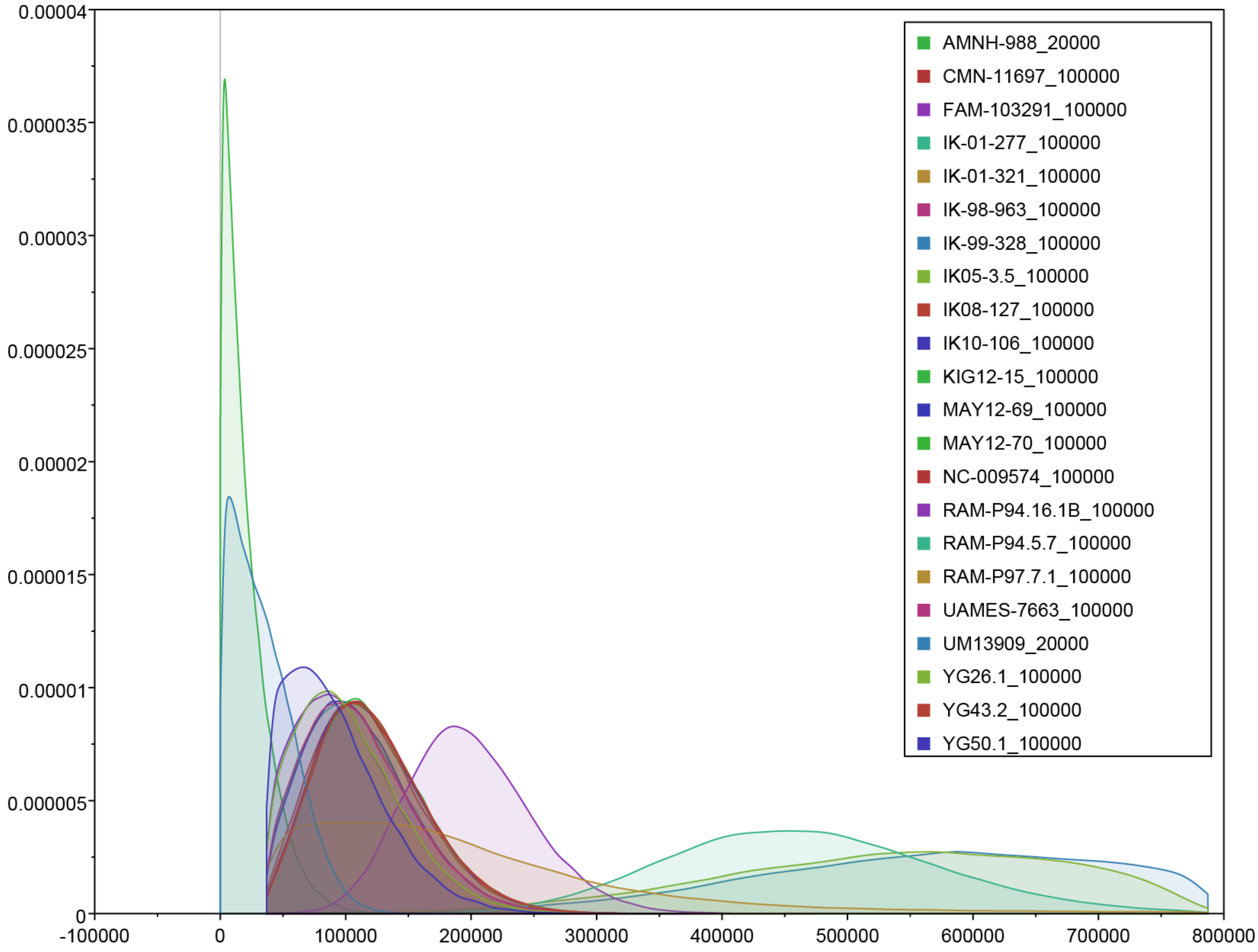

Supplementary Figure 21. Violin plot illustrating the posterior probability distributions of the ages of all undated specimens in the Joint analysis. Note that all ages are relative to the youngest specimen (Buesching – 13kya = t_0).

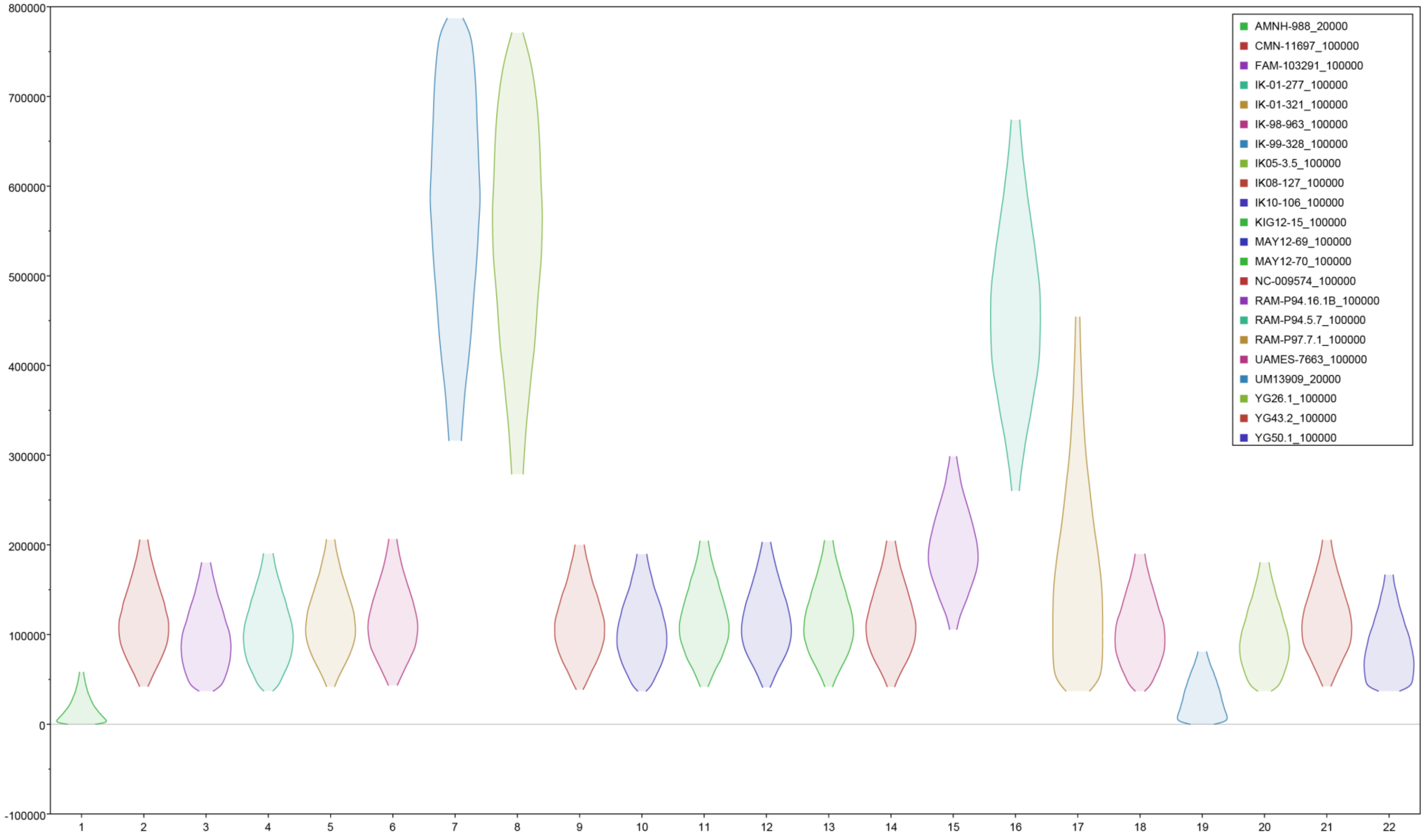

Supplementary Figure 22. MCC tree from the Joint analysis. Node heights are set to the median age of each node and bars illustrate the 95% HPD interval. Listed node values indicate the corrected node height (i.e. true age), while 95% HPD intervals are relative to the youngest specimen (Buesching @ 13kya).

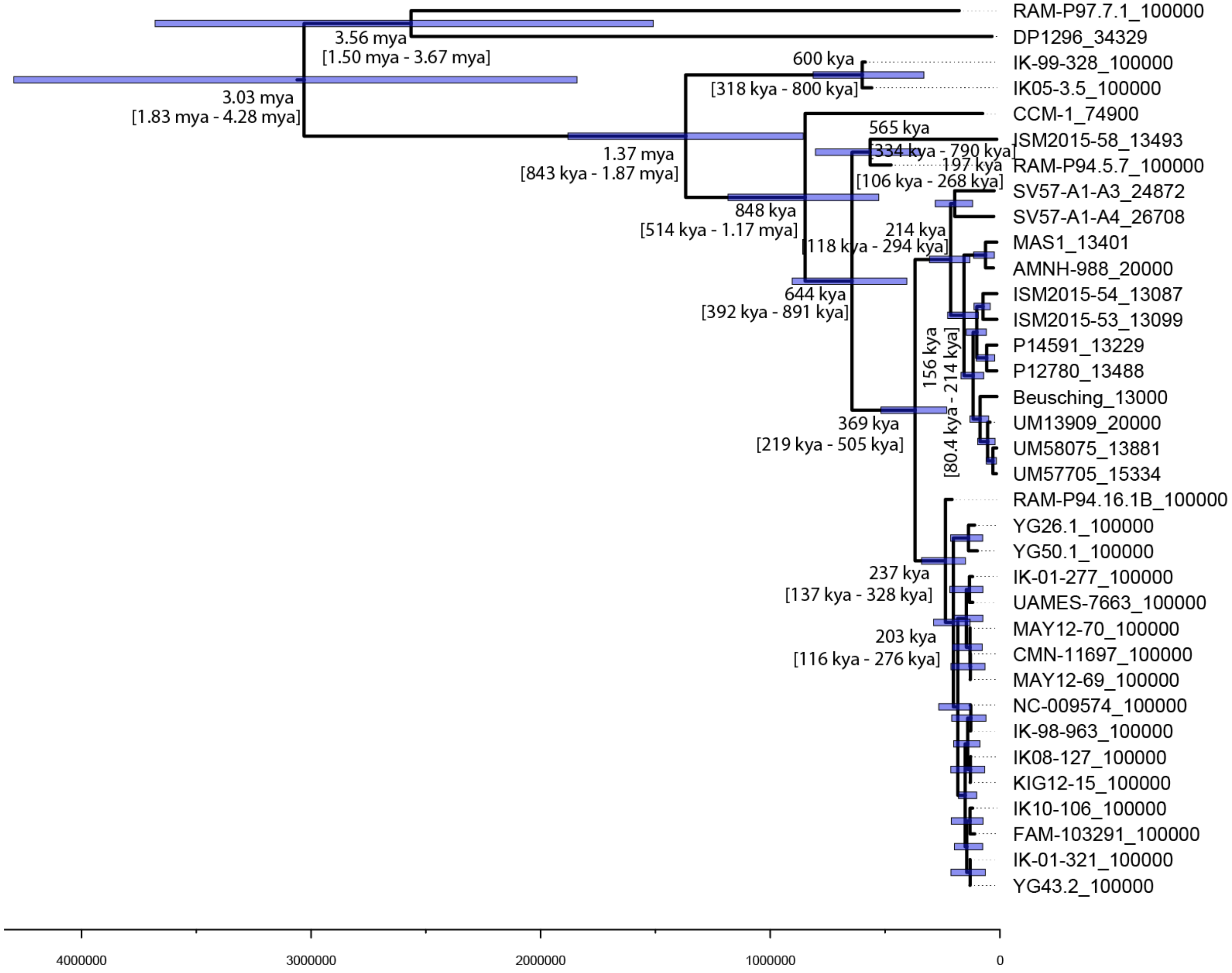

Supplementary Figure 23. MCC tree from the final Individually Dated analysis. Node heights are set to the median age of each node and bars illustrate the 95% HPD interval. Listed node values indicate the corrected node height (i.e. true age), while 95% HPD intervals are relative to the youngest specimen (Bueschİng @ 13kya).

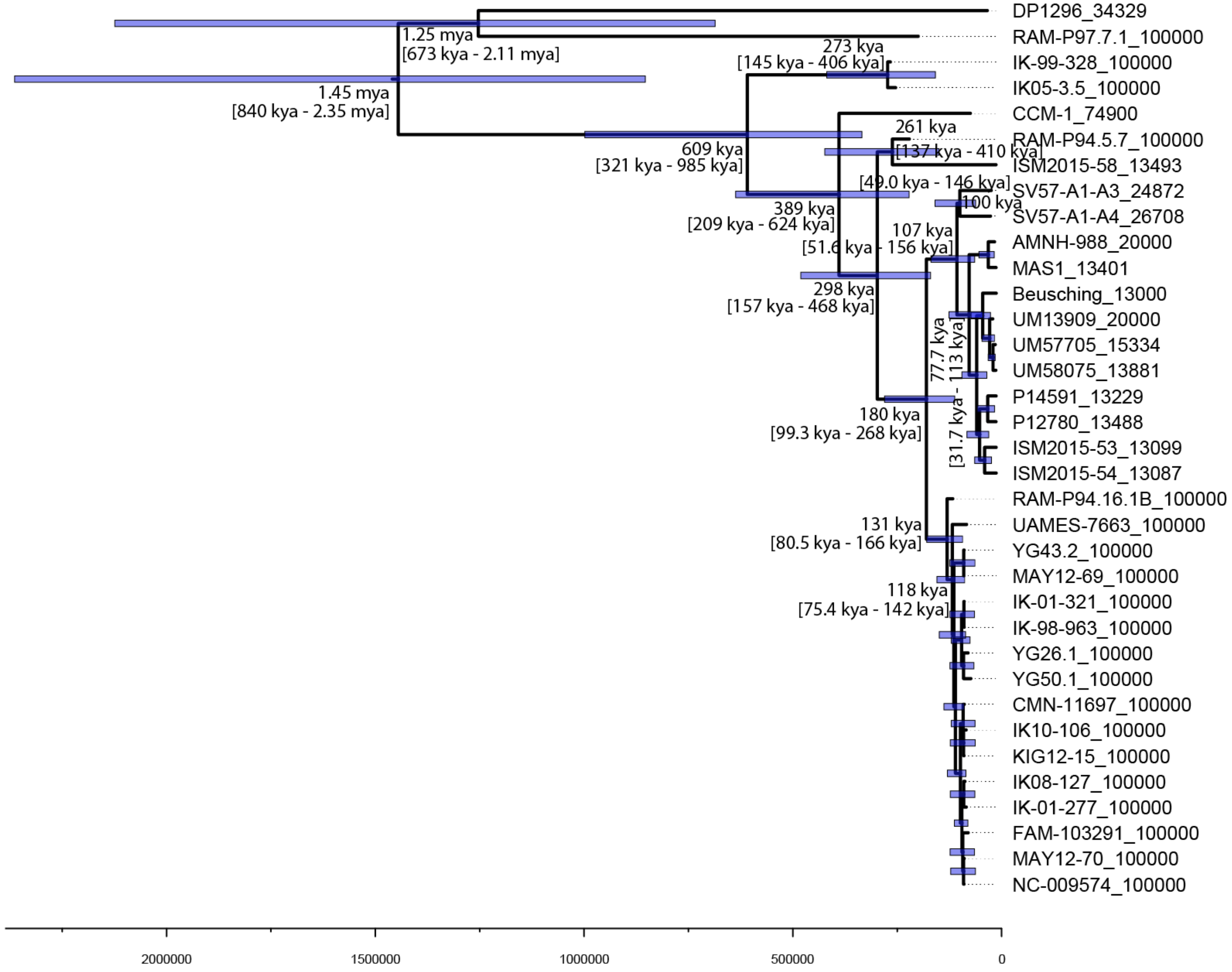

Genetic Diversity

To examine levels of genetic diversity in our dataset, we calculated nucleotide diversity within each of our major clades. Pairwise distances were calculated between all 35 mastodon samples using the dist.dna() function in ape [18], deleting data in a pairwise manner, and outputting as a matrix. A heat map was then generated using the ggplot() function in ggplot2 [26], with blue and red representing small and large distance values between sequences respectively. All distances were calculated using an F84 substitution model as HKY was not available. Nucleotide diversity for clades Y and G was calculated by taking the mean and standard deviation (functions mean() and sd() in R respectively) of all specimens within each clade both with and without RAM P94.16.1B (clade Y) and ETMNH 19335 and ETMNH 19334 (clade G) as these specimens are both temporally and geographically distinct from other mastodons in their respective clades.

Clade Y contains little diversity in comparison to clade G, regardless of whether RAM P96.16.1B ETMNH 19334 and ETMNH 19335 were included in the analysis (Supplementary Table 43; Supplementary Fig. 24; Main Text – Fig. 4). Likewise, the two specimens in clade A are also quite similar with a pairwise distance between them of only 1.24E-04 (2 total SNPs+indels). While only containing two individuals, the close similarity of both mastodons in this clade is consistent with what would be expected under our palaeoecological model.

Supplementary Table 43. Nucleotide diversity and mean combined SNPs and Indels of east Beringian and southern clades. The standard deviation around each estimate also provided. Clade A contains only two American mastodons, but the genetic distance and SNP+indel count is shown for comparison.

Clade	Nucleotide Diversity	SNPs + Indels
Y (excluding RAM P94.16.1B)	8.79E-05 ± 8.90E-05	1.69 ± 1.33
Y (including RAM P94.16.1B)	1.01E-04 ± 9.38E-05	1.83 ± 1.35
G (excluding ETMNH 19334 and 19335)	8.09E-04 ± 5.31E-04	11.09 ± 6.58
G (including ETMNH 19334 and 19335)	1.17E-03 ± 7.87E-04	16.20 ± 10.13
A	1.24E-04	2.00

Supplementary Figure 24. Pairwise distance heatmap for all mastodons in the study. Values represent proportion of sites that differ between sequences under an F84 model of evolution. See also Fig. 4 in the main text for a higher resolution view of clades G and Y.

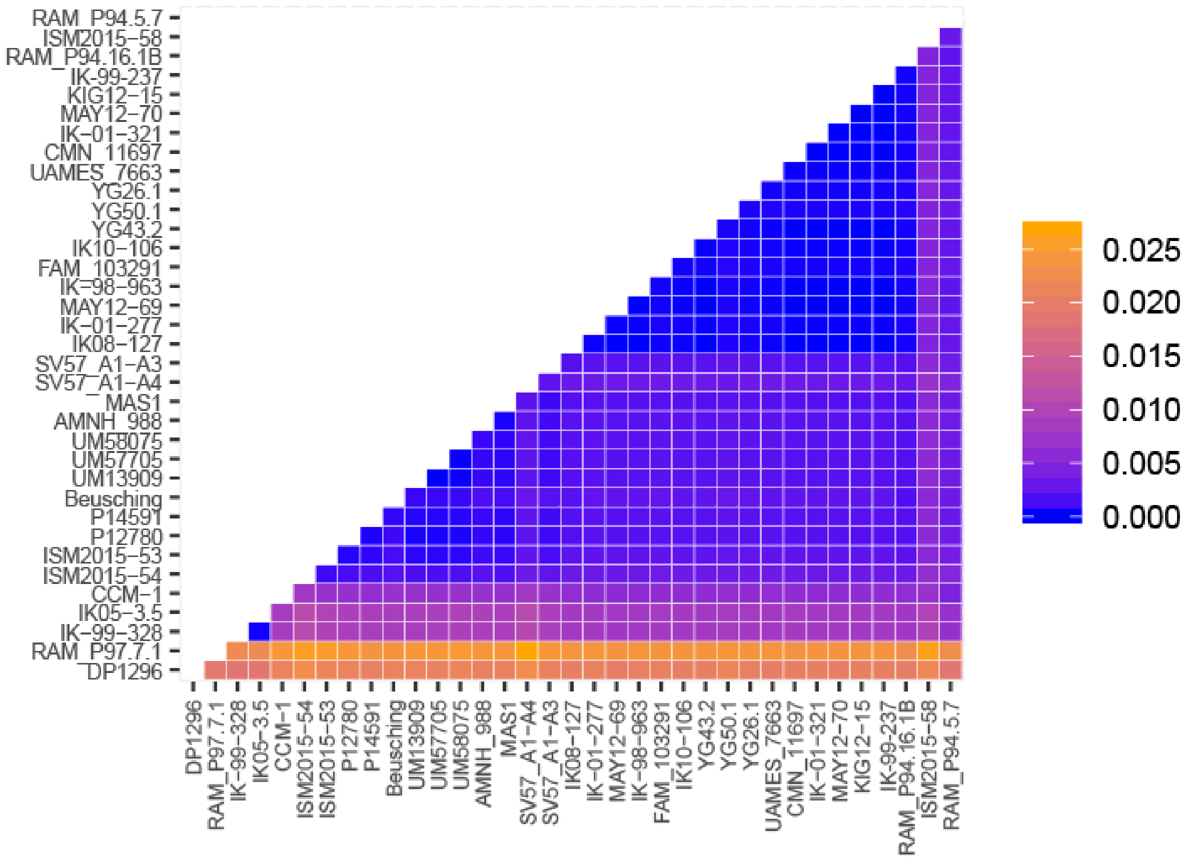

New Radiocarbon Dates

Newly reported radiocarbon dates are reported in Supplementary Table 44.

Specimens DP1296, DP885, DP234, DP3727, and DP5247 were sent to the Keck-CCAMS facility at the University of California, Irvine, where they were decalcified in 1 M HCl, gelatinized at 60°C and pH 2, and ultrafiltered to select a high molecular weight fraction (>30kDa). Only DP1296 produced sufficient collagen for measurement.

Specimens AMNH 982, AMNH 988, AMNH 26834, AMNH 22728, and AMNH 983 were also sent for radiocarbon analysis to the Keck-CCAMS facility at the University of California, Irvine. Bone samples were decalcified using 0.5 M HCl, rinsed with Milli-Q water, hydrolyzed overnight at 60°C with 0.01 M HCl, and the high molecular weight fraction isolated. Cleaned bone samples were additionally sonicated with acetone, methanol, and water to remove unknown consolidants. Only AMNH 22728 and AMNH 983 produced sufficient yields of ultrafiltered collagen for a measurement. Measurements of AMNH 22728 (UCIAMS 88779) and AMNH 983 (UCIAMS 88778) without additional solvent cleanup produced dates several thousand years older than measurements following solvent cleanup, suggesting that the cleanup removed significant amounts of a consolidant that was not completely ^{14}C-dead.

Specimens UAMES 7663 and CCM-1 were sent to the Oxford Radiocarbon Accelerator Unit for radiocarbon analysis. Specimens were prepared and measured as outlined using described in Ramsey et al. [27–29]. An additional hydroxyproline measurement (OxA-X-2457-7) was obtained for UAMES 7663, as a previous measurement was suspected of being artificially young due to the presence of consolidants. OxA-X-2457-7 was noted as being close to the background limit for the method, and should possibly be interpreted as the specimen is greater than the recovered age.

Supplementary Table 44. New ^{14}C results reported in this study. Laboratory numbers correspond to: UCIAMS – Keck Carbon Cycle AMS facility at the University of California, Irvine; OxA – Oxford Radiocarbon Accelerator Unit.

Laboratory number	Specimen	Material	^{14}C age (BP)
UCIAMS 211900	DP1296	Dentine	30320 ± 300
OxA-X-2457-7	UAMES 7663	Bone	43000 ± 2200
OxA-25401	UAMES 7663	Bone	20440 ± 130
OxA-25404	CCM-1	Bone	> 50000
UCIAMS 88777	AMNH 22728	Bone	36220 ± 610
UCIAMS 88778	AMNH 983	Bone	31770 ± 360
UCIAMS 88779	AMNH 22728	Bone	30870 ± 320
UCIAMS 88780	AMNH 983	Bone	35150 ± 550
UCIAMS 88781	AMNH 22728	Bone	37500 ± 720

Appendix A

<u>Note from the Authors:</u>

Following peer review, significant work was done to validate the molecular clock age estimation experiments, which ultimately coincided in a new round of model selection and complete rework of the molecular clock dating analysis. In the interest of full transparency the Supplementary Methods presented below describe the molecular clock dating experiments that were included in the initial submission of this manuscript (the final revised experiments described in the final paper are presented above). However, it is worth noting, that the results described in Model Testing With GSS subsection above suggest that lognormal clock models and skyline demographic priors are poor fits for the data.

Temporal Signal Exploration with TempEst

To explore the temporal signal within our dataset we constructed reduced maximum likelihood phylogenies for data exploration in TempEst (v1.5.1) [30]. Maximum likelihood phylogenies were constructed from all samples (HKY+G; 100 bootstrap replicates) for which some external temporal calibration was available. Samples with radiocarbon dates were calibrated in the desktop version of CALIB (v7.04) [31] (Supplementary Table 45), and median probability values used as input for TempEst.

Analysis of the phylogeny in TempEst identified a positive temporal signal in the data (Correlation Coefficient = 0.4648; $R^2 = 0.216$) in line with what has been observed with other megafauna datasets [30]. However, two samples (ISM2015-54 and ETMNH_19335) were identified as having faster rates of evolution across their branches then their ages would suggest (Supplementary Fig. 25). As such we also repeated TempEst analysis with a reduced dataset of dated samples excluding these two samples from the analysis. This resulted in a higher correlation coefficient (0.653) and R^2 (0.4264). Both datasets were subsequently used for clock and demographic model selection in BEAST.

Supplementary Table 45. Radiocarbon calibration for all samples with finite ages, as well as the final age ranges for two samples with Electronic Spin Resonance and stratigraphy age estimates. For samples with multiple radiocarbon dates, the most recent date was used for calibration. MAS1 produced a 2 sigma range spanning two time intervals, which was combined with a new mean and standard deviation for all subsequent analyses.

Specimen	Age Type	Uncalibrated Age	2 Sigma Range	Median Age	Mean Age	Standard Deviation
CCM-1	ESR	74900 ± 5000	69900-79900	74900	74900	2500
P12780	14C (AA98897UF)	11620 ± 110	13254-13721	13488	13487.5	116.75
UM58075	14C (Beta-23268)	12020 ± 120	13570-14153	13881	13861.5	145.75
UM57705	14C (Beta-1389)	12485 ± 165	14703-15893	15334	15298	297.5
Beusching	Stratigraphy	13000	-	13000	13000	1
P14591	14C (AA98898)	11380 ± 110	13059-13441	13229	13250	95.5
ETMNH 19335	14C (NZA 61419)	22408 ± 180	26208-27175	26708	26691.5	241.75
ETMNH 19334	14C (NZA 61418)	20653 ± 144	24436-25294	24872	24865	214.5
ISM2015-58	14C (AA101549)	11660 ± 130	13249-13761	13493	13505	128
ISM2015-53	14C (AA106381)	11230 ± 70	12952-13270	13099	13111	79.5
ISM2015-54	14C (AA106382)	11220 ± 80	12883-13268	13087	13075.5	96.25
MAS1	14C (BETA-371886)	11570 ± 60	13278-13496<br>13516-13543	13401	13410.5	66.25
DP1296	14C (UCIAMS 211900)	30320 ± 300	33834-34829	34329	34331.5	248.75

Supplementary Figure 25. (A) Tree with branch lengths indicating samples with faster (blue) or slower (red) rates of evolution then would be expected based on the samples age. (B) Plot of residuals showing deviations from the regression line of all samples. ETMNH 19335 (SV57 A1-A4) and ISM2015-54 are indicated by the orange box.

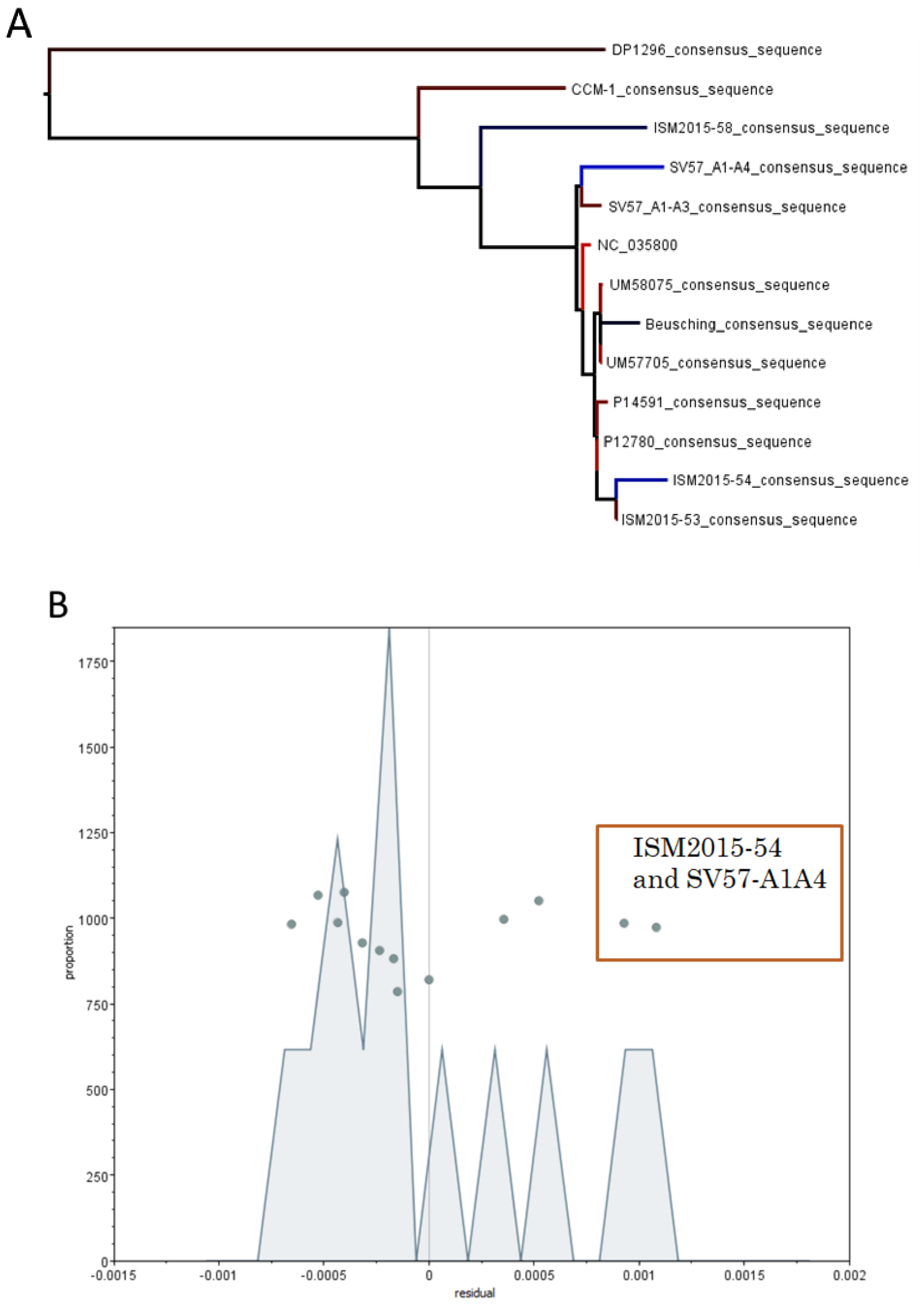

Model Selection with PS/SS

Molecular clock and demographic model selection was done using path sampling/stepping stone in BEAST (v1.8.0) [16,32], on all dated samples as well as on the reduced dated dataset (excludes ISM2015-54 and ETMNH 19335). Tip ages were sampled with individual normal prior distributions using the calibrated 2-sigma distributions as priors on each sample (Supplementary Table 45). For samples where the calibrated age contained multiple distributions, the range was extended to encompass the full 2-sigma range and a new mean calculated to fit a normal prior. Beusching was fit with a normal prior of standard deviation 1, to allow for proposal movement, but to represent its nature as a stratigraphic point estimate. A separate set of analyses was also done using just the calibrated median age (no tip date prior distributions) to ensure consistency between estimates.

Two clock models (strict clock and uncorrelated lognormal relaxed clock) were tested with each dataset and using both tip prior distributions and only median ages. In all cases the relevant rate prior (strict clock – clock.rate; lognormal clock – ucld.mean) was set to a uniform distribution between 0.4E-9 and 8E-9 with an initial value of 4.2E-9 based on previous rates observed for mastodons [33] and mammoths [8].

All dataset, temporal information priors, and clock models were also tested under two demographic models: a constant size model with diffuse lognormal prior (LogNormal [1,10] initial value = 6.25E5); and a skyline model with 5 piecewise-constant groups and a default skyline.popSize prior. All chains were run for 100 million generations (sampling every 10 thousand), with path sampling/stepping stone estimation preformed upon conclusion using 100 path steps at 1 million generations each (logging every 1000). Each analysis was performed in duplicate to ensure consistency among marginal log likelihood estimates. A summary of each run in available in Supplementary Table 46.

Bayes factors from path sampling (Supplementary Fig. 26) and stepping stone (Supplementary Fig. 27) on all dated samples consistently favoured the uncorrelated lognormal relaxed clock regardless of whether prior distributions or median ages were used to calibrate the phylogeny. Marginal likelihood estimates under both the constant population size and skyline demographic models were essentially equal with slightly greater support seen for the constant size model seen in most runs. Both replicates produced consistent estimates (Supplementary Table 46).

Analysis of the reduced dated dataset showed very similar trends, with the uncorrelated lognormal clock being consistently favoured over strict clock models (path sampling – Supplementary Fig. 28; stepping stone – Supplementary Fig. 29). However, support was much lower than observed in using all dated samples (mean all dated BFs PS-38.99 and SS-39.06; mean reduced dataset BFs PS-6.31 and SS-6.32), on the border of strong to positive support for lognormal clock models [23]. As before, both demographic models gave very similar log marginal likelihood estimates, with a slightly increase in support for a constant size prior.

Supplementary Table 46. Path/Stepping stone MLE runs for all dated mastodons. Runs were done in duplicate (denoted by A or B at the end of the run ID) to ensure consistency between analyses. The log marginal likelihood for each run from both Path and Stepping Stone methods is indicated next to each run.

Run	MLE Protocol		Run Details
	Path Sampling	Stepping Stone	
54A	-25167.49	-25167.42	All dated mastodons, strict clock, only median ages, constant population size
54B	-25167.98	-25167.92	
55A	-25148.59	-25148.59	All dated mastodons, lognormal clock, only median ages, constant population size
55B	-25147.79	-25147.77	
56A	-25148.31	-25148.21	All dated mastodons, lognormal clock, age prior distributions, constant population size
56B	-25148.62	-25148.35	
57A	-25168.03	-25167.98	All dated mastodons, strict clock, age prior distributions, constant population size
57B	-25167.35	-25167.38	
58A	-24839.96	-24839.89	Reduced dated set, strict clock, only median ages, constant population size
58B	-24840.03	-24840.00	
59A	-24836.12	-24836.05	Reduced dated set, lognormal clock, only median ages, constant population size
59B	-24837.04	-24836.95	
60A	-24837.23	-24837.22	Reduced dated set, lognormal clock, age prior distributions, constant population size
60B	-24836.08	-24836.09	
61A	-24839.79	-24839.78	Reduced dated set, strict clock, age prior distributions, constant population size
61B	-24840.72	-24840.80	
62A	-25168.39	-25168.36	All dated mastodons, strict clock, only median ages, skyline
62B	-25168.24	-25168.17	
63A	-25150.57	-25150.65	All dated mastodons, lognormal clock, only median ages, skyline
63B	-25148.89	-25148.89	
64A	-25147.94	-25147.87	All dated mastodons, lognormal clock, age prior distributions, skyline
64B	-25148.83	-25148.62	
65A	-25168.68	-25168.72	All dated mastodons, strict clock, age prior distributions, skyline
65B	-25169.34	-25169.24	
66A	-24840.34	-24840.36	Reduced dated set, strict clock, only median ages, skyline
66B	-24839.93	-24839.91	
67A	-24837.10	-24837.21	Reduced dated set, lognormal clock, only median ages, skyline
67B	-24837.28	-24837.15	
68A	-24837.90	-24837.81	Reduced dated set, lognormal clock, age prior distributions, skyline
68B	-24837.46	-24837.68	
69A	-24840.47	-24840.35	Reduced dated set, strict clock, age prior distributions, skyline
69B	-24840.21	-24840.34	

Supplementary Figure 26. Path sampling Bayes factors for all dated mastodons. Run IDs correspond to parameters listed in Supplementary Table 46. Bayes factors were calculated by multiplying the difference between the row and the column log marginal likelihood by two. Colours correspond to the support scheme in Kass and Raftery (1995): Dark Red (BF<-10) indicating very strong support for the column model; Light Red (BF -6 to -10) indicates strong support of the column model; Orange (BF -2 to -6) indicates positive support for the column model; Yellow (BF 0 to -2) indicates marginal support for the column model; Dark Blue (BF 0 to -2) indicates marginal support for the row model; Light Blue (BF 2 to 6) indicates positive support for the row model; Light Green (BF 6 to 10) indicates strong support of the row model; Dark Green (BF >10) indicating very strong support for the row model.

			Constant Population Size								Skyline							
	Path Sampling Bayes Factors_AllDated		54A	54B	55A	55B	56A	56B	57A	57B	62A	62B	63A	63B	64A	64B	65A	65B
			-25167.48823	-25167.97834	-25148.6	-25147.78673	-25148.30661	-25148.6	-25168.03059	-25167.35113	-25168.38888	-25168.2	-25150.6	-25148.9	-25147.9	-25148.8	-25168.7	-25169.3
Constant Population Size	54A	-25167.48823		0.980213501	-37.806	-39.40300078	-38.36323466	-37.7431	1.084713069	-0.274199616	1.801295708	1.503685	-33.8409	-37.2043	-39.0965	-37.323	2.379346	3.704028
	54B	-25167.97834	-0.980213501		-38.7862	-40.38321428	-39.34344816	-38.7233	0.104499568	-1.254413117	0.821082207	0.523472	-34.8211	-38.1845	-40.0768	-38.3032	1.399133	2.723815
	55A	-25148.58524	37.80598692	38.78620042		-1.597013858	-0.557247735	0.062863	38.89069999	37.53178731	39.60728263	39.30967	3.965096	0.6017	-1.29056	0.482999	40.18533	41.51002
	55B	-25147.78673	39.40300078	40.38321428	1.597014		1.039766123	1.659877	40.48771385	39.12880117	41.20429649	40.90669	5.56211	2.198713	0.306455	2.080013	41.78235	43.10703
	56A	-25148.30661	38.36323466	39.34344816	0.557248	-1.039766123		0.620111	39.44794773	38.08903504	40.16453037	39.86692	4.522344	1.158947	-0.73331	1.040247	40.74258	42.06726
	56B	-25148.61667	37.74312411	38.72333761	-0.06286	-1.659876676	-0.620110553		38.82783717	37.46892449	39.54441981	39.24681	3.902233	0.538837	-1.35342	0.420136	40.12247	41.44715
	57A	-25168.03059	-1.084713069	-0.104499568	-38.8907	-40.48771385	-39.44794773	-38.8278		-1.358912685	0.716582638	0.418972	-34.9256	-38.289	-40.1813	-38.4077	1.294633	2.619315
	57B	-25167.35113	0.274199616	1.254413117	-37.5318	-39.12880117	-38.08903504	-37.4689	1.358912685		2.075495323	1.777885	-33.5667	-36.9301	-38.8223	-37.0488	2.653546	3.978228
Skyline	62A	-25168.38888	-1.801295708	-0.821082207	-39.6073	-41.20429649	-40.16453037	-39.5444	-0.716582638	-2.075495323		-0.29761	-35.6422	-39.0056	-40.8978	-39.1243	0.578051	1.902732
	62B	-25168.24007	-1.503685425	-0.523471924	-39.3097	-40.90668621	-39.86692008	-39.2468	-0.418972356	-1.777885041	0.297610283		-35.3446	-38.708	-40.6002	-38.8267	0.875661	2.200343
	63A	-25150.56778	33.84089103	34.82110454	-3.9651	-5.562109747	-4.522343624	-3.90223	34.9256041	33.56669142	35.64218674	35.34458		-3.3634	-5.25566	-3.4821	36.22024	37.54492
	63B	-25148.88609	37.20428734	38.18450084	-0.6017	-2.19871344	-1.158947317	-0.53884	38.28900041	36.93008773	39.00558305	38.70797	3.363396		-1.89226	-0.1187	39.58363	40.90832
	64A	-25147.93996	39.09654606	40.07675956	1.290559	-0.306454718	0.733311405	1.353422	40.18125913	38.82234645	40.89784177	40.60023	5.255655	1.892259		1.773558	41.47589	42.80057
	64B	-25148.82674	37.32298776	38.30320126	-0.483	-2.080013022	-1.040246899	-0.42014	38.40770083	37.04878814	39.12428347	38.82667	3.482097	0.1187	-1.77356		39.70233	41.02702
	65A	-25168.6779	-2.379346422	-1.399132921	-40.1853	-41.7823472	-40.74258108	-40.1225	-1.294633353	-2.653546038	-0.578050714	-0.87566	-36.2202	-39.5836	-41.4759	-39.7023		1.324682
	65B	-25169.34024	-3.704028177	-2.723814676	-41.51	-43.10702896	-42.06726284	-41.4472	-2.619315108	-3.978227793	-1.90273247	-2.20034	-37.5449	-40.9083	-42.8006	-41.027	-1.32468	

Supplementary Figure 27. Stepping stone Bayes factors for all dated mastodons. Run IDs correspond to parameters listed in Supplementary Table 46. Bayes factors were calculated by multiplying the difference between the row and the column log marginal likelihood by two. Colours correspond to the support scheme in Kass and Raftery (1995): Dark Red (BF<-10) indicating very strong support for the column model; Light Red (BF -6 to -10) indicates strong support of the column model; Orange (BF -2 to -6) indicates positive support for the column model; Yellow (BF 0 to -2) indicates marginal support for the column model; Dark Blue (BF 0 to -2) indicates marginal support for the row model; Light Blue (BF 2 to 6) indicates positive support for the row model; Light Green (BF 6 to 10) indicates strong support of the row model; Dark Green (BF >10) indicating very strong support for the row model.

			Constant Population Size								Skyline							
	Stepping Stone Bayes Factors_AllDates		54A	54B	55A	55B	56A	56B	57A	57B	62A	62B	63A	63B	64A	64B	65A	65B
			-25167.42311	-25167.92059	-25148.6	-25147.77063	-25148.2095	-25148.4	-25167.97937	-25167.37725	-25168.35631	-25168.2	-25150.7	-25148.9	-25147.9	-25148.6	-25168.7	-25169.2
Constant Population Size	54A	-25167.42311		0.994959355	-37.6694	-39.30495964	-38.42723033	-38.1462	1.112509353	-0.091720996	1.866402073	1.500163	-33.5453	-37.0684	-39.1044	-37.6034	2.595884	3.636014
	54B	-25167.92059	-0.994959355		-38.6644	-40.29991899	-39.42218969	-39.1411	0.117549998	-1.086680351	0.871442718	0.505203	-34.5403	-38.0633	-40.0994	-38.5984	1.600925	2.641055
	55A	-25148.58841	37.66940567	38.66436502		-1.635553971	-0.757824668	-0.47678	38.78191502	37.57768467	39.53580774	39.16957	4.124084	0.601038	-1.43503	0.065961	40.26529	41.30542
	55B	-25147.77063	39.30495964	40.29991899	1.635554		0.877729303	1.158772	40.41746899	39.21323864	41.17136171	40.80512	5.759638	2.236592	0.200528	1.701515	41.90084	42.94097
	56A	-25148.2095	38.42723033	39.42218969	0.757825	-0.877729303		0.281042	39.53973969	38.33550934	40.29363241	39.92739	4.881908	1.358862	-0.6772	0.823785	41.02311	42.06324
	56B	-25148.35002	38.14618811	39.14114747	0.476782	-1.158771524	-0.281042221		39.25869747	38.05446712	40.01259019	39.64635	4.600866	1.07782	-0.95824	0.542743	40.74207	41.7822
	57A	-25167.97937	-1.112509353	-0.117549998	-38.7819	-40.41746899	-39.53973969	-39.2587		-1.204230349	0.75389272	0.387653	-34.6578	-38.1809	-40.2169	-38.716	1.483375	2.523505
	57B	-25167.37725	0.091720996	1.086680351	-37.5777	-39.21323864	-38.33550934	-38.0545	1.204230349		1.958123069	1.591884	-33.4536	-36.9766	-39.0127	-37.5117	2.687605	3.727735
Skyline	62A	-25168.35631	-1.866402073	-0.871442718	-39.5358	-41.17136171	-40.29363241	-40.0126	-0.75389272	-1.958123069		-0.36624	-35.4117	-38.9348	-40.9708	-39.4698	0.729482	1.769612
	62B	-25168.17319	-1.500162605	-0.50520325	-39.1696	-40.80512224	-39.92739294	-39.6464	-0.387653252	-1.591883601	0.366239468		-35.0455	-38.5685	-40.6046	-39.1036	1.095722	2.135851
	63A	-25150.65045	33.54532185	34.5402812	-4.12408	-5.759637789	-4.881908486	-4.60087	34.6578312	33.45360085	35.41172392	35.04548		-3.52305	-5.55911	-4.05812	36.14121	37.18134
	63B	-25148.88893	37.06836809	38.06332745	-0.60104	-2.236591545	-1.358862242	-1.07782	38.18087745	36.9766471	38.93477017	38.56853	3.523046		-2.03606	-0.53508	39.66425	40.70438
	64A	-25147.8709	39.10443145	40.09939081	1.435026	-0.200528186	0.677201117	0.958243	40.21694081	39.01271046	40.97083353	40.60459	5.55911	2.036063		1.500986	41.70032	42.74045
	64B	-25148.62139	37.6034451	38.59840445	-0.06596	-1.701514538	-0.823785235	-0.54274	38.71595445	37.5117241	39.46984717	39.10361	4.058123	0.535077	-1.50099		40.19933	41.23946
	65A	-25168.72105	-2.59588437	-1.600925015	-40.2653	-41.90084401	-41.0231147	-40.7421	-1.483375017	-2.687605366	-0.729482297	-1.09572	-36.1412	-39.6643	-41.7003	-40.1993		1.04013
	65B	-25169.24112	-3.636014065	-2.64105471	-41.3054	-42.9409737	-42.0632444	-41.7822	-2.523504712	-3.727735061	-1.769611992	-2.13585	-37.1813	-40.7044	-42.7404	-41.2395	-1.04013	

Supplementary Figure 28. Path sampling Bayes factors for the reduced dated mastodon dataset. Run IDs correspond to parameters listed in Supplementary Table 46. Bayes factors were calculated by multiplying the difference between the row and the column log marginal likelihood by two. Colours correspond to the support scheme in Kass and Raftery (1995): Dark Red (BF<-10) indicating very strong support for the column model; Light Red (BF -6 to -10) indicates strong support of the column model; Orange (BF -2 to -6) indicates positive support for the column model; Yellow (BF 0 to -2) indicates marginal support for the column model; Dark Blue (BF 0 to -2) indicates marginal support for the row model; Light Blue (BF 2 to 6) indicates positive support for the row model; Light Green (BF 6 to 10) indicates strong support of the row model; Dark Green (BF >10) indicating very strong support for the row model.

			Constant Population Size								Skyline							
	Path Sampling Bayes Factors_ReducDated		58A	58B	59A	59B	60A	60B	61A	61B	66A	66B	67A	67B	68A	68B	69A	69B
			-24839.9592	-24840.02617	-24836.1	-24837.04015	-24837.23242	-24836.1	-24839.79063	-24840.72206	-24840.3413	-24839.9	-24837.1	-24837.3	-24837.9	-24837.5	-24840.5	-24840.2
Constant Population Size	58A	-24839.9592		0.133938367	-7.68294	-5.83810254	-5.453544818	-7.7684	-0.33713952	1.525733581	0.764206601	-0.05312	-5.71663	-5.3651	-4.12622	-4.9902	1.015962	0.493742
	58B	-24840.02617	-0.133938367		-7.81688	-5.972040908	-5.587483185	-7.90234	-0.471077887	1.391795213	0.630268234	-0.18705	-5.85057	-5.49904	-4.26015	-5.12414	0.882024	0.359804
	59A	-24836.11773	7.682938296	7.816876663		1.844835756	2.229393478	-0.08546	7.345798776	9.208671877	8.447144897	7.629823	1.966305	2.317836	3.556723	2.692738	8.6989	8.17668
	59B	-24837.04015	5.83810254	5.972040908	-1.84484		0.384557723	-1.9303	5.50096302	7.363836121	6.602309142	5.784987	0.121469	0.473	1.711887	0.847902	6.854065	6.331845
	60A	-24837.23242	5.453544818	5.587483185	-2.22939	-0.384557723		-2.31485	5.116405297	6.979278398	6.217751419	5.40043	-0.26309	0.088443	1.32733	0.463344	6.469507	5.947287
	60B	-24836.075	7.768399611	7.902337978	0.085461	1.930297071	2.314854793		7.431260091	9.294133192	8.532606212	7.715284	2.051766	2.403297	3.642184	2.778199	8.784362	8.262142
	61A	-24839.79063	0.33713952	0.471077887	-7.3458	-5.50096302	-5.116405297	-7.43126		1.862873101	1.101346122	0.284024	-5.37949	-5.02796	-3.78908	-4.65306	1.353102	0.830882
	61B	-24840.72206	-1.525733581	-1.391795213	-9.20867	-7.363836121	-6.979278398	-9.29413	-1.862873101		-0.761526979	-1.57885	-7.24237	-6.89084	-5.65195	-6.51593	-0.50977	-1.03199
Skyline	66A	-24840.3413	-0.764206601	-0.630268234	-8.44714	-6.602309142	-6.217751419	-8.53261	-1.101346122	0.761526979		-0.81732	-6.48084	-6.12931	-4.89042	-5.75441	0.251756	-0.27046
	66B	-24839.93264	0.053115202	0.187053569	-7.62982	-5.784987338	-5.400429616	-7.71528	-0.284024318	1.578848783	0.817321803		-5.66352	-5.31199	-4.0731	-4.93709	1.069077	0.546857
	67A	-24837.10088	5.716633114	5.850571481	-1.96631	-0.121469426	0.263088297	-2.05177	5.379493594	7.242366695	6.480839716	5.663518		0.351531	1.590418	0.726433	6.732595	6.210375
	67B	-24837.27665	5.365102211	5.499040578	-2.31784	-0.473000329	-0.088442606	-2.4033	5.027962691	6.890835792	6.129308813	5.311987	-0.35153		1.238887	0.374902	6.381064	5.858844
	68A	-24837.89609	4.126215156	4.260153524	-3.55672	-1.711887384	-1.327329661	-3.64218	3.789075636	5.651948737	4.890421758	4.0731	-1.59042	-1.23889		-0.86399	5.142177	4.619957
	68B	-24837.4641	4.99020059	5.124138957	-2.69274	-0.847901951	-0.463344228	-2.7782	4.653061069	6.51593417	5.754407191	4.937085	-0.72643	-0.3749	0.863985		6.006163	5.483943
	69A	-24840.46718	-1.015962199	-0.882023832	-8.6989	-6.85406474	-6.469507017	-8.78436	-1.353101719	0.509771381	-0.251755598	-1.06908	-6.7326	-6.38106	-5.14218	-6.00616		-0.52222
	69B	-24840.20607	-0.493742114	-0.359803747	-8.17668	-6.331844654	-5.947286932	-8.26214	-0.830881634	1.031991467	0.270464487	-0.54686	-6.21038	-5.85884	-4.61996	-5.48394	0.52222	

Supplementary Figure 29. Stepping stone Bayes factors for the reduced dated mastodon dataset. Run IDs correspond to parameters listed in Supplementary Table 46. Bayes factors were calculated by multiplying the difference between the row and the column log marginal likelihood by two. Colours correspond to the support scheme in Kass and Raftery (1995): Dark Red (BF<-10) indicating very strong support for the column model; Light Red (BF -6 to -10) indicates strong support of the column model; Orange (BF -2 to -6) indicates positive support for the column model; Yellow (BF 0 to -2) indicates marginal support for the column model; Dark Blue (BF 0 to -2) indicates marginal support for the row model; Light Blue (BF 2 to 6) indicates positive support for the row model; Light Green (BF 6 to 10) indicates strong support of the row model; Dark Green (BF >10) indicating very strong support for the row model.

	Stepping Stone Bayes Factors_ReducDated		58A	58B	59A	59B	60A	60B	61A	61B	66A	66B	67A	67B	68A	68B	69A	69B
			-24839.89364	-24840.00136	-24836.1	-24836.95103	-24837.21739	-24836.1	-24839.78312	-24840.80281	-24840.36245	-24839.9	-24837.2	-24837.2	-24837.8	-24837.7	-24840.3	-24840.3
Constant Population Size	58A	-24839.89364		0.215439533	-7.68233	-5.885209353	-5.352495085	-7.61371	-0.221023438	1.818344853	0.937624439	0.028918	-5.36415	-5.47778	-4.16055	-4.4345	0.908337	0.8999
	58B	-24840.00136	-0.215439533		-7.89777	-6.100648886	-5.567934617	-7.82915	-0.43646297	1.60290532	0.722184906	-0.18652	-5.57959	-5.69322	-4.37599	-4.64994	0.692898	0.684461
	59A	-24836.05247	7.682330977	7.897770509		1.797121623	2.329835892	0.068625	7.461307539	9.500675829	8.619955416	7.711249	2.318177	2.204551	3.521785	3.247833	8.590668	8.582231
	59B	-24836.95103	5.885209353	6.100648886	-1.79712		0.532714269	-1.7285	5.664185916	7.703554206	6.822833792	5.914127	0.521055	0.40743	1.724664	1.450711	6.793547	6.78511
	60A	-24837.21739	5.352495085	5.567934617	-2.32984	-0.532714269		-2.26121	5.131471647	7.170839938	6.290119524	5.381413	-0.01166	-0.12528	1.191949	0.917997	6.260832	6.252396
	60B	-24836.08678	7.613706131	7.829145664	-0.06862	1.728496778	2.261211047		7.392682694	9.432050984	8.55133057	7.642624	2.249552	2.135927	3.45316	3.179208	8.522043	8.513607
	61A	-24839.78312	0.221023438	0.43646297	-7.46131	-5.664185916	-5.131471647	-7.39268		2.03936829	1.158647877	0.249941	-5.14313	-5.25676	-3.93952	-4.21347	1.129361	1.120924
	61B	-24840.80281	-1.818344853	-1.60290532	-9.50068	-7.703554206	-7.170839938	-9.43205	-2.03936829		-0.880720414	-1.78943	-7.1825	-7.29612	-5.97889	-6.25284	-0.91001	-0.91844
Skyline	66A	-24840.36245	-0.937624439	-0.722184906	-8.61996	-6.822833792	-6.290119524	-8.55133	-1.158647877	0.880720414		-0.90871	-6.30178	-6.4154	-5.09817	-5.37212	-0.02929	-0.03772
	66B	-24839.9081	-0.028917988	0.186521545	-7.71125	-5.914127341	-5.381413073	-7.64262	-0.249941426	1.789426865	0.908706451		-5.39307	-5.5067	-4.18946	-4.46342	0.879419	0.870982
	67A	-24837.21156	5.364154054	5.579593587	-2.31818	-0.521055299	0.011658969	-2.24955	5.143130616	7.182498907	6.301778493	5.393072		-0.11363	1.203608	0.929656	6.272491	6.264055
	67B	-24837.15475	5.477779581	5.693219113	-2.20455	-0.407429773	0.125284496	-2.13593	5.256756143	7.296124434	6.41540402	5.506698	0.113626		1.317234	1.043281	6.386117	6.37768
	68A	-24837.81336	4.160545781	4.375985314	-3.52179	-1.724663572	-1.191949303	-3.45316	3.939522344	5.978890634	5.09817022	4.189464	-1.20361	-1.31723		-0.27395	5.068883	5.060446
	68B	-24837.67639	4.434498173	4.649937705	-3.24783	-1.450711181	-0.917996912	-3.17921	4.213474735	6.252843026	5.372122612	4.463416	-0.92966	-1.04328	0.273952		5.342835	5.334399
	69A	-24840.34781	-0.908337155	-0.692897623	-8.59067	-6.793546509	-6.26083224	-8.52204	-1.129360593	0.910007698	0.029287284	-0.87942	-6.27249	-6.38612	-5.06888	-5.34284		-0.00844
	69B	-24840.34359	-0.899900452	-0.684460919	-8.58223	-6.785109805	-6.252395536	-8.51361	-1.120923889	0.918444401	0.037723987	-0.87098	-6.26405	-6.37768	-5.06045	-5.3344	0.008437	

Molecular Clock Dating

We attempted to estimate the ages of undated and non-finite specimens in our phylogeny using BEAST v1.8.0 [16]. Specimen ages were sampled with individual normal prior distributions as above (Supplementary Table 45), and samples with unknown or non-finite ages were fit with a gamma distribution (shape = 1; scale = 200,000), with an upper bound at 800,000 (approximate age of oldest recovered ancient DNA) and a lower bound for of 50,000 ya (approximate limit of radiocarbon dating) for known or presumed non-finite samples. Full age priors and operators for all samples can be found in Supplementary Table 47.

Supplementary Table 47. BEAST tip date priors and operators manually set for each specimen. Molecular dating analysis was done both with and without ISM2015-54 and ETMNH 19335 to calibrate the tree. For analyses where the calibrating information for these two specimens was removed, it was replaced with a diffuse gamma distribution as for other samples of unknown age.

Specimen	Distribution	Initial Value	Additional Parameters
ISM2015-58	Normal	13493.0	Mean = 13505.0<br>Stdev = 128.0<br>Weight = 1
ISM2015-54	Normal	13087.0	Mean = 13075.5<br>Stdev = 96.25<br>Weight = 1
	Gamma	20000.0	Shape = 1.0<br>Scale = 200000.0<br>Offset = 0.0<br>Bound = [0.0, 800000.0]<br>Weight = 5
IK08-127	Gamma	100000.0	Shape = 1.0<br>Scale = 200000.0<br>Offset = 50000.0<br>Bound = [50000.0, 800000.0]<br>Weight = 5
Beusching	Normal	13000.0	Mean = 13000.0<br>Stdev = 1.0<br>Weight = 1
YG50.1	Gamma	100000.0	Shape = 1.0<br>Scale = 200000.0<br>Offset = 50000.0<br>Bound = [50000.0, 800000.0]<br>Weight = 5
P14591	Normal	13229.0	Mean = 13250.0<br>Stdev = 95.5<br>Weight = 1
ETMNH 19335	Normal	26708.0	Mean = 26691.5<br>Stdev = 241.75<br>Weight = 1
	Gamma	20000.0	Shape = 1.0<br>Scale = 200000.0<br>Offset = 50000.0

			Bound = [50000.0, 800000.0]<br>Weight = 5
DP1296	Normal	34329.0	Mean = 34331.5<br>Stdev = 248.75<br>Weight = 1
RAM_P94.16.1B	Gamma	100000.0	Shape = 1.0<br>Scale = 200000.0<br>Offset = 50000.0<br>Bound = [50000.0, 800000.0]<br>Weight = 5
RAM_P94.5.7	Gamma	100000.0	Shape = 1.0<br>Scale = 200000.0<br>Offset = 50000.0<br>Bound = [50000.0, 800000.0]<br>Weight = 5
RAM_P97.7.1	Gamma	100000.0	Shape = 1.0<br>Scale = 200000.0<br>Offset = 50000.0<br>Bound = [50000.0, 800000.0]<br>Weight = 5
UAMES_7663	Gamma	100000.0	Shape = 1.0<br>Scale = 200000.0<br>Offset = 50000.0<br>Bound = [50000.0, 800000.0]<br>Weight = 5
IK-01-277	Gamma	100000.0	Shape = 1.0<br>Scale = 200000.0<br>Offset = 50000.0<br>Bound = [50000.0, 800000.0]<br>Weight = 5
AMNH_988	Gamma	20000.0	Shape = 1.0<br>Scale = 200000.0<br>Offset = 0.0<br>Bound = [0.0, 800000.0]<br>Weight = 5
MAY12-69	Gamma	100000.0	Shape = 1.0<br>Scale = 200000.0<br>Offset = 50000.0<br>Bound = [50000.0, 800000.0]<br>Weight = 5
CCM-1	Normal	74900.0	Mean = 74900.0<br>Stdev = 2500.0<br>Weight = 1
CMN_11697	Gamma	100000.0	Shape = 1.0<br>Scale = 200000.0<br>Offset = 50000.0<br>Bound = [50000.0, 800000.0]<br>Weight = 5

UM13909	Gamma	20000.0	Shape = 1.0<br>Scale = 200000.0<br>Offset = 0.0<br>Bound = [0.0, 800000.0]<br>Weight = 5
UM57705	Normal	15334.0	Mean = 15298.0<br>Stdev = 297.5<br>Weight = 1
IK-99-328	Gamma	100000.0	Shape = 1.0<br>Scale = 200000.0<br>Offset = 50000.0<br>Bound = [50000.0, 800000.0]<br>Weight = 5
IK-98-963	Gamma	100000.0	Shape = 1.0<br>Scale = 200000.0<br>Offset = 50000.0<br>Bound = [50000.0, 800000.0]<br>Weight = 5
ISM2015-53	Normal	13099.0	Mean = 13111.0<br>Stdev = 79.5<br>Weight = 1
UM58075	Normal	13881.0	Mean = 13861.5<br>Stdev = 145.75<br>Weight = 1
FAM_103291	Gamma	100000.0	Shape = 1.0<br>Scale = 200000.0<br>Offset = 50000.0<br>Bound = [50000.0, 800000.0]<br>Weight = 5
IK05-3.5	Gamma	100000.0	Shape = 1.0<br>Scale = 200000.0<br>Offset = 50000.0<br>Bound = [50000.0, 800000.0]<br>Weight = 5
ETMNH 19334	Normal	24872.0	Mean = 24865.0<br>Stdev = 214.5<br>Weight = 1
IK10-106	Gamma	100000.0	Shape = 1.0<br>Scale = 200000.0<br>Offset = 50000.0<br>Bound = [50000.0, 800000.0]<br>Weight = 5
YG43.2	Gamma	100000.0	Shape = 1.0<br>Scale = 200000.0<br>Offset = 50000.0<br>Bound = [50000.0, 800000.0]<br>Weight = 5
MAY12-70	Gamma	100000.0	Shape = 1.0

			Scale = 200000.0<br>Offset = 50000.0<br>Bound = [50000.0, 800000.0]<br>Weight = 5
YG26.1	Gamma	100000.0	Shape = 1.0<br>Scale = 200000.0<br>Offset = 50000.0<br>Bound = [50000.0, 800000.0]<br>Weight = 5
P12780	Normal	13448.0	Mean = 13487.5<br>Stdev = 116.75<br>Weight = 1
IK-01-321	Gamma	100000.0	Shape = 1.0<br>Scale = 200000.0<br>Offset = 50000.0<br>Bound = [50000.0, 800000.0]<br>Weight = 5
MAS1	Normal	13401.0	Mean = 13410.5<br>Stdev = 66.25<br>Weight = 1
KIG12-15	Gamma	100000.0	Shape = 1.0<br>Scale = 200000.0<br>Offset = 50000.0<br>Bound = [50000.0, 800000.0]<br>Weight = 5
IK-99-237	Gamma	100000.0	Shape = 1.0<br>Scale = 200000.0<br>Offset = 50000.0<br>Bound = [50000.0, 800000.0]<br>Weight = 5

Phylogenies were calibrated using both all dated samples and using only the reduced dated dataset for calibration. In accordance with the results of the model selection (Supplementary Methods – Model Selection), runs were done with an uncorrelated lognormal clock under a constant population size demographic prior. However, we also redid this analysis with a strict clock to examine the differences in estimates produced, and in the case of the reduced dated dataset, because the Bayes factor indicated a smaller difference between the fit of the lognormal versus strick clock models than it did for the entire dataset. As before the respective clock rate priors (strict clock – clock.rate; lognormal clock – ucld.mean) was set to a uniform distribution between 0.4E-9 and 8E-9 with an initial value of 4.2E-9.

All BEAST runs used an HKY+G4 substitution model, the most supported model implemented in BEAST v1.8.0. The constant.popSize prior was fit with a diffuse lognormal (LogNormal [1,10] initial value = 8.7E5). Chains were increased in length to 500 million generations (sampling every 10 thousand). Operator weights on undated samples were also increased from 1 to 5 to ensure sufficient sampling. All chains were run in triplicate to ensure consistency of estimates and subsequently combined for the final analyses.

Clade Y estimates using the lognormal clock (all dated samples for calibration; constant population size) produced median estimates between 141.13-168.42 kya (Supplementary Table 48) which is broadly consistent with the MIS 5 interglacial (130-71 kya), with the posterior probability distribution maximum for 10 of the 15 samples is located within the MIS 5 time period. However, the 95% HPD intervals surrounding these estimates are broad (combined HPD: 50-419.8 kya; Supplementary Fig. 30) making this association difficult to address directly, likely as a result of the large amount of rate variation (median ucld.mean = 2.044E-9; median ucld.stdev = 1.50) favored by BEAST and the lack of calibration points in this clade.

Specimen RAM P94.16.1B displays a flatter posterior probability distribution with a median of 341.340 kya (95% HPD: 50-686kya), much older that what is observed for other specimens in clade Y. Although the large and overlapping 95% HPD makes it difficult to say this specimen is indeed temporally distinct for other mastodons in clade Y, if true this would suggest multiple northward expansions from a single source population, possibly correlating to earlier interglacial periods.

Mastodons in clade A also produce median ages that are much older than those in clade Y, however these samples also exhibit much broader 95% HPD intervals, a trend consistent with all remaining non-finite samples within our phylogeny. This prevents us from decisively identifying these samples as belonging to a separate temporal period in Eastern Beringia, despite the large amount of the probability distribution that lies outside that assigned to specimens in clade Y.

Both other undated Alberta mastodons (RAM P97.7.1 and RAM P94.5.7) produce similar distributions to those observed in clade A specimens, with older median estimates, but very wide 95% HPD intervals indicating a high degree of uncertainty. Despite their uncertain temporal provenance, the close geographic proximity of these specimens and RAM P94.16.1B, but drastically different phylogenetic positions within our tree, is perhaps indicative of this the highly dynamic nature of this region with respect to mastodon movement, with multiple very distinct lineages occupying this region.

Our two undated mastodons in clade G produce relatively young median ages (AMNH 988 - 57.2 kya; UM13909 - 54.2 kya), although older than the dated specimens in this clade. As in clade Y, the median age of these mastodons is being influenced by the long tails observed in the posterior probability distribution and the associated large 95% HPDs, whereas their maximuma are relatively close in age to the other specimens within that clade. Notably, this also highlights the effect the high degree of rate variation has on the data, as this clade contains 10 other closely related specimens all with finite radiocarbon dates.

Analyses using a strict clock (all dated samples; constant population size) produce the same pattern as above, but with greater separation between the major clades (Supplementary Table 48; Supplementary Fig. 31). Additionally, the 95% HPD intervals around estimates tend to be narrower, as would be expected when rate variation is removed. Age estimates also tend be younger than under a lognormal clock for specimens near calibration points (i.e., Clade Y and undated specimens within clades G and M) and older estimates for specimens more distant from calibration points (i.e., Clade A).

Clade Y mastodons produce median age estimates within the MIS 5 interglacial or within 1000 years of its onset, and with all mastodons' individual probability maximum being within MIS 5 limits. RAM P94.16.1B produces a much more concentrated distribution centered on 216 kya (MIS 7 interglacial), although again with some overlap in the 95% HPDs between it and the other mastodons in clade Y. Unlike with the lognormal clock, clade A mastodons produce much older median estimates and non-

overlaping 95% HPD intervals with clade Y mastodons, strongly suggesting they are in fact from two separate temporal periods.

Substituting a skyline demographic prior for the constant population size prior has little effect on age estimates, as would be expected given the results of the PS/SS model selection (Supplementary Table 48; Supplementary Fig. 32; Supplementary Fig. 33). Median ages tend to be fairly consistent with their respective constant population size analyses, as was how concentrated or diffuse the 95% HPD intervals were surrounding those estimates.

Repeating the analysis with the reduced dated mastodon set for calibration (i.e., omitting ISM2015-54 and ETMNH 19335 as calibration points) has little effect on the estimated ages of most mastodons (Supplementary Table 48). Median age estimates with both strict and lognormal clocks tend to be within a few thousand years of estimates generated using all dated mastodons for calibration. 95% HPD intervals likewise followed a similar pattern to what was observed when calibrating with the full dataset for both the lognormal (Supplementary Fig. 34) and strict (Supplementary Fig. 35) clocks. Notably, we do not observe a large difference in the amount of rate variation of the lognormal clock with the reduced dataset (median ucld.mean = 1.3946E-9; median ucld.stdev = 1.5185). This possibly indicates that the observed rate variation is not derived entirely from true variations in the mitochondrial mutation rate, but as a result of the MCMC’s inability to properly assess the likelihood of rate changes at deeper points in our phylogeny relative to the temporal depth of our calibration points.

As before substituting a skyline demographic prior for a constant size population one has a marginal effect on the median age estimates and 95% HPD intervals (Supplementary Table 48; lognormal clock – Supplementary Fig. 36; strict clock – Supplementary Fig. 37).

Supplementary Table 48. Median, mean, and maximum of all American mastodons samples in the analysis from the molecular clock dating BEAST runs. Clock model, whether all dated mastodons (all dated) or the reduced dated mastodon dataset (reduc dated) was used for calibration, and the demographic prior used are provided in the Run column.

Run		ISM2015-58	ISM2015-54	IK08-127	Beusching	YG50.1	P14591	ETMNH 19335	DP1296
Log clock, all dated, constant pop size	Median	13505.28	13074.31	1.66E+05	13000	1.41E+05	13250.42	26691.54	34332.96
	Maximum	13513.25	13062.56	107312.5	13000	72687.5	13259.81	26672.25	34345.25
	95% HPD	[13255.9992, 13756.0092]	[12884.0525, 13259.9566]	[50003.5312, 4.1549E5]	[12998.0453, 13001.95]	[50001.4825, 3.8204E5]	[13059.8393, 13434.21]	[26218.6747, 27160.4487]	[33853.5684, 34829.6194]
Strict clock, all dated, constant pop size	Median	13505.33	13074.42	1.28E+05	13000	98019.92	13249.76	26688.49	34332.46
	Maximum	13511.75	13065.38	116812.5	12999.93	74937.5	13248.38	26689.75	34301.75
	95% HPD	[13252.7976, 13755.2798]	[12888.7974, 13265.4511]	[50086.1249, 2.209E5]	[12998.0596, 13001.9634]	[50000.206, 1.8494E5]	[13064.2643, 13438.9473]	[26215.9279, 27167.0378]	[33853.8402, 34828.8768]
Log clock, all dated, Skyline	Median	13505.08	13075.84	1.72E+05	13000	1.21E+05	13249.84	26691.61	34331.46
	Maximum	13499.25	13080.38	122562.5	13000.04	66562.5	13251.13	26703.25	34309.75
	95% HPD	[13253.1815, 13754.244]	[12889.4182, 13266.2439]	[50003.1999, 3.8573E5]	[12998.0497, 13001.9812]	[50000.9457, 3.0622E5]	[13062.8074, 13436.6548]	[26210.7987, 27157.7985]	[33841.2777, 34818.7957]
Strict clock, all dated, Skyline	Median	13503.82	13073.98	139580	1.30E+04	97538.14	13249.42	26686.06	34332.08
	Maximum	13504.25	13071.69	133937.5	13000.01	84562.5	13247.19	26675.25	34323.25
	95% HPD	[13253.7772, 13754.7573]	[12884.462, 13260.0992]	[61177.6059, 2.2696E5]	[12998.0287, 13001.9442]	[50000.4164, 1.7407E5]	[13063.3936, 13436.7745]	[26219.5956, 27166.3742]	[33846.6775, 34821.6274]
Log clock, reduc dated, constant pop size	Median	13504.61	33458.28	1.72E+05	13000	1.46E+05	13249.71	1.38E+05	34331.76
	Maximum	13498.75	7187.5	86812.5	12999.97	71937.5	13249.31	30187.5	34349.25
	95% HPD	[13259.2178, 13761.3733]	[0.3694, 1.9291E5]	[50000.4368, 4.5244E5]	[12998.0491, 13001.9664]	[50000.5465, 4.1602E5]	[13063.9938, 13438.1086]	[5.0943, 5.2272E5]	[33840.311, 34810.653]
Strict clock, reduc dated, constant pop size	Median	13504.31	3676.979	1.21E+05	13000	93740.77	13250.57	8754.692	34333.06
	Maximum	13500.75	831.25	108312.5	13000.08	71062.5	13257.63	2225	34338.75
	95% HPD	[13252.5634, 13754.9988]	[0.0894, 17001.3727]	[50307.4809, 2.0539E5]	[12998.0585, 13001.972]	[50000.155, 1.7205E5]	[13061.6966, 13436.7124]	[0.3164, 40273.4687]	[33849.3301, 34823.5265]
Log clock, reduc dated, Skyline	Median	13505.31	35523.72	1.76E+05	13000	1.24E+05	13250.4	1.38E+05	34332.45
	Maximum	13499.75	7562.5	81312.5	12999.94	67187.5	13245.94	30062.5	34340.75
	95% HPD	[13259.3002, 13760.0304]	[0.1236, 1.6512E5]	[50002.281, 4.0822E5]	[12998.0558, 13001.9576]	[50000.4202, 3.2669E5]	[13062.8276, 13438.5092]	[1.9984, 5.867E5]	[33849.3736, 34829.7143]
Strict clock, reduc dated, Skyline	Median	13505.23	3309.282	1.25E+05	13000	92014.99	13249.6	6684.14	34332.88
	Maximum	13514.75	756.25	118562.5	12999.95	84375	13249.06	1725	34331.75
	95% HPD	[13251.1754, 13753.6694]	[0.1205, 15375.2823]	[60678.9315, 2.008E5]	[12998.022, 13001.9387]	[50000.6503, 1.5497E5]	[13063.3448, 13436.1198]	[3.5422E-3, 30387.9966]	[33854.9081, 34827.7901]

Supplementary Table 48. Table continued from previous page.

Run		RAM_P94.16.1B	RAM_P94.5.7	RAM_P97.7.1	UAMES_7663	IK-01-277	AMNH_988	MAY12-69	CCM-1
Log clock, all dated, constant pop size	Median	3.41E+05	2.82E+05	3.13E+05	1.53E+05	1.54E+05	57130.0706	1.67E+05	74928.8047
	Maximum	304062.5	97187.5	185937.5	79937.5	76687.5	12312.5	110562.5	74707.5
	95% HPD	[50000.1484, 6.8602E5]	[50006.9528, 6.6906E5]	[50017.703, 7.0007E5]	[50002.3635, 3.9799E5]	[50000.6818, 3.9813E5]	[0.4354, 3.0116E5]	[50001.8963, 4.1618E5]	[70055.3802, 79841.0784]
Strict clock, all dated, constant pop size	Median	2.16E+05	5.05E+05	1.79E+05	1.18E+05	1.19E+05	16859.8528	1.30E+05	75012.3967
	Maximum	201687.5	469687.5	133562.5	108562.5	107812.5	3625	115312.5	74787.5
	95% HPD	[1.1773E5, 3.5153E5]	[2.9778E5, 7.8191E5]	[50010.035, 4.8414E5]	[50000.8238, 2.1017E5]	[50004.0285, 2.1094E5]	[0.3462, 69685.5244]	[50475.0174, 2.244E5]	[70123.4514, 79912.707]
Log clock, all dated, Skyline	Median	3.56E+05	3.67E+05	4.05E+05	1.44E+05	1.44E+05	69143.7974	1.75E+05	74908.9744
	Maximum	343937.5	87812.5	405937.5	71812.5	71562.5	14312.5	128062.5	74847.5
	95% HPD	[50027.7473, 6.8133E5]	[50032.7335, 7.1743E5]	[50005.4504, 7.3246E5]	[50001.3391, 3.446E5]	[50000.0982, 3.4537E5]	[0.4847, 2.9571E5]	[50002.7811, 3.9114E5]	[70041.5527, 79841.7678]
Strict clock, all dated, Skyline	Median	204580	4.96E+05	2.52E+05	1.27E+05	1.27E+05	1.80E+04	141870	7.50E+04
	Maximum	191687.5	428687.5	215812.5	119062.5	123312.5	4025	131937.5	75032.5
	95% HPD	[1.1521E5, 3.2684E5]	[2.8802E5, 7.8647E5]	[50048.824, 5.4456E5]	[50012.1006, 2.0585E5]	[50573.2531, 2.0733E5]	[0.0633, 74957.5735]	[65293.3952, 2.3071E5]	[70169.6997, 79963.0412]
Log clock, reduc dated, constant pop size	Median	3.35E+05	2.74E+05	2.84E+05	1.57E+05	1.57E+05	74058.0454	1.73E+05	74910.3189
	Maximum	286687.5	92812.5	94187.5	74062.5	75437.5	16687.5	97312.5	74867.5
	95% HPD	[50013.9694, 6.9975E5]	[50004.4209, 6.7659E5]	[50002.5143, 6.9529E5]	[50001.5631, 4.3247E5]	[50004.4249, 4.3821E5]	[0.5008, 3.6802E5]	[50004.2262, 4.5509E5]	[69961.71, 79713.5425]
Strict clock, reduc dated, constant pop size	Median	1.93E+05	4.30E+05	1.67E+05	1.13E+05	1.13E+05	14424.8131	1.23E+05	75033.6341
	Maximum	175437.5	362312.5	119687.5	102062.5	104812.5	3225	114437.5	75107.5
	95% HPD	[1.0609E5, 3.1973E5]	[2.4558E5, 7.3471E5]	[50000.7204, 4.8271E5]	[50024.8893, 1.9612E5]	[50024.7951, 1.9714E5]	[0.2577, 60132.1457]	[52237.5753, 2.0996E5]	[70171.8776, 79975.7234]
Log clock, reduc dated, Skyline	Median	3.63E+05	3.58E+05	3.88E+05	1.47E+05	1.47E+05	78787.1781	1.79E+05	74903.5671
	Maximum	360687.5	87812.5	87687.5	72062.5	71812.5	17437.5	85312.5	74622.5
	95% HPD	[50020.0509, 6.9628E5]	[50017.5999, 7.1958E5]	[50007.2901, 7.3032E5]	[50002.1487, 3.6313E5]	[50000.1277, 3.6495E5]	[0.2661, 3.3475E5]	[50000.4887, 4.1087E5]	[70086.7346, 79848.7694]
Strict clock, reduc dated, Skyline	Median	1.71E+05	3.85E+05	2.19E+05	1.16E+05	1.16E+05	14056.7194	1.27E+05	75063.3277
	Maximum	156937.5	337062.5	107312.5	109825	110437.5	3225	120075	75142.5
	95% HPD	[1.0192E5, 2.7891E5]	[2.2357E5, 6.9113E5]	[50002.96, 5.0705E5]	[53086.7566, 1.8575E5]	[52839.4111, 1.8686E5]	[0.6596, 55693.4337]	[63854.0586, 2.0387E5]	[70144.6118, 79937.2975]

Supplementary Table 48. Table continued from previous page.

Run		CMN_11697	UM13909	UM57705	IK-99-328	IK-98-963	ISM2015-53	UM58075	FAM_103291
Log clock, all dated, constant pop size	Median	1.67E+05	54065.4681	15297.0173	3.18E+05	1.67E+05	13111.042	13860.9699	1.45E+05
	Maximum	115937.5	14187.5	15291.25	233312.5	110937.5	13108.6875	13870.75	73812.5
	95% HPD	[50001.4055, 4.198E5]	[1.5944, 2.5289E5]	[14719.0151, 15882.3693]	[50020.433, 6.8574E5]	[50000.6418, 4.1679E5]	[12954.1232, 13264.7231]	[13573.9997, 14143.9279]	[50002.9985, 3.8395E5]
Strict clock, all dated, constant pop size	Median	1.30E+05	39759.9849	15301.0851	5.64E+05	1.30E+05	13110.923	13862.1815	1.09E+05
	Maximum	117812.5	19875	15280.75	569312.5	116937.5	13110.8125	13863.25	91812.5
	95% HPD	[50223.7027, 2.2322E5]	[6.8305, 1.051E5]	[14726.3542, 15889.5041]	[2.9932E5, 7.9998E5]	[51550.791, 2.2497E5]	[12953.8031, 13266.2849]	[13573.3426, 14143.4919]	[50001.6555, 1.9886E5]
Log clock, all dated, Skyline	Median	1.76E+05	97263.6306	15297.9585	3.19E+05	1.76E+05	13111.1202	13860.7536	1.30E+05
	Maximum	115812.5	21812.5	15275.75	305062.5	118812.5	13107.6875	13855.25	69187.5
	95% HPD	[50002.6206, 3.908E5]	[2.21, 3.3247E5]	[14716.0334, 15880.6198]	[50018.2577, 6.6285E5]	[50000.473, 3.9148E5]	[12955.1626, 13264.9074]	[13573.7486, 14145.2564]	[50000.5376, 3.2205E5]
Strict clock, all dated, Skyline	Median	141670	4.60E+04	15300.1834	498740	1.42E+05	1.31E+04	13861.6634	114330
	Maximum	134687.5	20175	15270.75	496437.5	135937.5	13119.9375	13864.25	107687.5
	95% HPD	[66900.2757, 2.3178E5]	[2.593, 1.2348E5]	[14726.0342, 15893.5155]	[2.472E5, 7.8822E5]	[67346.1021, 2.3178E5]	[12955.8415, 13267.5276]	[13577.6327, 14148.3094]	[50002.4657, 1.935E5]
Log clock, reduc dated, constant pop size	Median	1.73E+05	70871.2478	15298.827	2.87E+05	1.74E+05	13110.4327	13862.4688	1.50E+05
	Maximum	86187.5	16937.5	15317.25	110437.5	88187.5	13108.5625	13869.25	72312.5
	95% HPD	[50000.9541, 4.5649E5]	[0.2228, 3.2122E5]	[14730.0896, 15895.3596]	[50015.3844, 6.7136E5]	[50001.2301, 4.5433E5]	[12956.3938, 13267.2879]	[13578.9318, 14148.9495]	[50001.1681, 4.2111E5]
Strict clock, reduc dated, constant pop size	Median	1.23E+05	35173.5528	15304.7363	5.39E+05	1.23E+05	13111.4644	13861.3694	1.05E+05
	Maximum	113812.5	19937.5	15273.25	512687.5	115312.5	13110.8125	13856.75	93687.5
	95% HPD	[52238.4789, 2.1009E5]	[0.0243, 90553.2028]	[14724.8663, 15887.3936]	[3.031E5, 7.9996E5]	[52940.4441, 2.0967E5]	[12957.3425, 13267.9035]	[13569.8388, 14141.4983]	[50009.1545, 1.8589E5]
Log clock, reduc dated, Skyline	Median	1.79E+05	1.11E+05	15297.5173	3.03E+05	1.80E+05	13110.9038	13862.6157	1.33E+05
	Maximum	83812.5	22812.5	15282.25	97062.5	87312.5	13112.1875	13861.25	68812.5
	95% HPD	[50000.5542, 4.103E5]	[0.2625, 3.608E5]	[14720.7279, 15883.3704]	[50007.6178, 6.5788E5]	[50000.7096, 4.107E5]	[12953.0671, 13264.4387]	[13573.9842, 14144.2187]	[50000.6211, 3.4001E5]
Strict clock, reduc dated, Skyline	Median	1.27E+05	36572.2462	15306.1717	4.66E+05	1.27E+05	13111.182	13862.1124	1.06E+05
	Maximum	121062.5	21375	15328.75	433562.5	122025	13111.9375	13870.25	103325
	95% HPD	[63799.4466, 2.0483E5]	[14.5489, 90530.6709]	[14730.4017, 15892.003]	[2.2639E5, 7.5771E5]	[64642.3777, 2.0422E5]	[12956.4975, 13267.9263]	[13576.0493, 14147.2351]	[50001.6218, 1.7225E5]

Supplementary Table 48. Table continued from previous page.

Run		IK05-3.5	ETMNH 19334	IK10-106	YG43.2	MAY12-70	YG26.1	P12780	IK-01-321
Log clock, all dated, constant pop size	Median	2.51E+05	24865.5382	1.53E+05	1.68E+05	1.68E+05	1.46E+05	13487.2094	1.67E+05
	Maximum	88687.5	24867.25	78437.5	106562.5	112312.5	73187.5	13489.375	112187.5
	95% HPD	[50005.0207, 6.1966E5]	[24448.2561, 25289.4811]	[50001.5025, 3.9587E5]	[50004.3696, 4.1756E5]	[50001.0577, 4.1736E5]	[50000.6049, 3.8667E5]	[13256.6226, 13715.4685]	[50001.6084, 4.1704E5]
Strict clock, all dated, constant pop size	Median	5.33E+05	24864.6437	1.19E+05	1.30E+05	1.31E+05	1.10E+05	13488.1477	1.31E+05
	Maximum	545312.5	24857.75	105437.5	116437.5	118437.5	95312.5	13487.125	116312.5
	95% HPD	[2.5357E5, 7.8017E5]	[24447.3396, 25290.1604]	[50008.009, 2.1131E5]	[51194.7174, 2.239E5]	[51258.9378, 2.2559E5]	[50004.6681, 1.999E5]	[13259.6153, 13716.5704]	[53077.4245, 2.2683E5]
Log clock, all dated, Skyline	Median	2.36E+05	24864.6066	1.45E+05	1.76E+05	1.76E+05	1.31E+05	13486.9667	1.76E+05
	Maximum	82937.5	24859.25	72312.5	115812.5	138687.5	67687.5	13477.25	112187.5
	95% HPD	[50003.9486, 5.8292E5]	[24450.086, 25290.7062]	[50001.7727, 3.4758E5]	[50001.2544, 3.8938E5]	[50001.3502, 3.9056E5]	[50001.6255, 3.2324E5]	[13255.739, 13713.2151]	[50000.5602, 3.9004E5]
Strict clock, all dated, Skyline	Median	4.67E+05	2.49E+04	127820	1.42E+05	1.42E+05	1.15E+05	1.35E+04	142260
	Maximum	461562.5	24860.75	120812.5	132812.5	136312.5	107125	13488.75	135187.5
	95% HPD	[2.0409E5, 7.468E5]	[24445.3322, 25287.266]	[50391.3814, 2.0766E5]	[65580.0501, 2.3111E5]	[66544.4299, 2.3103E5]	[50002.5337, 1.9365E5]	[13259.1201, 13717.578]	[66413.9279, 2.3087E5]
Log clock, reduc dated, constant pop size	Median	2.22E+05	24865.9545	1.58E+05	1.73E+05	1.73E+05	1.51E+05	13487.2657	1.73E+05
	Maximum	84437.5	24856.25	74187.5	102562.5	82812.5	72562.5	13484.75	86562.5
	95% HPD	[50001.7084, 6.0207E5]	[24446.3774, 25285.0805]	[50000.4603, 4.3279E5]	[50001.8076, 4.5272E5]	[50000.4759, 4.5517E5]	[50000.5585, 4.2243E5]	[13258.1554, 13716.2347]	[50000.3851, 4.5247E5]
Strict clock, reduc dated, constant pop size	Median	5.14E+05	24866.8256	1.14E+05	1.24E+05	1.23E+05	1.05E+05	13488.1824	1.23E+05
	Maximum	498062.5	24872.25	105812.5	113562.5	111937.5	93687.5	13487.75	108687.5
	95% HPD	[2.6213E5, 7.7278E5]	[24447.601, 25287.6465]	[50021.0086, 1.9699E5]	[53986.4136, 2.1201E5]	[53157.0386, 2.114E5]	[50000.2119, 1.8635E5]	[13258.541, 13715.2948]	[52758.35, 2.1052E5]
Log clock, reduc dated, Skyline	Median	2.13E+05	24865.3092	1.48E+05	1.80E+05	1.79E+05	1.33E+05	13487.834	1.79E+05
	Maximum	81187.5	24863.75	71812.5	100062.5	96312.5	68187.5	13481.875	86937.5
	95% HPD	[50001.0702, 5.7697E5]	[24451.1597, 25292.3007]	[50000.0211, 3.6644E5]	[50000.96, 4.1227E5]	[50000.0085, 4.111E5]	[50000.1243, 3.4256E5]	[13264.1128, 13720.6724]	[50004.3101, 4.1131E5]
Strict clock, reduc dated, Skyline	Median	4.42E+05	24866.5349	1.17E+05	1.28E+05	1.28E+05	1.06E+05	13489.178	1.28E+05
	Maximum	415687.5	24873.75	107312.5	123062.5	121312.5	101725	13486.75	121437.5
	95% HPD	[2.0091E5, 7.2626E5]	[24445.9044, 25284.6631]	[53723.2484, 1.8821E5]	[64711.9901, 2.0472E5]	[63437.7357, 2.0263E5]	[50001.2108, 1.726E5]	[13258.791, 13713.9294]	[63031.1137, 2.0318E5]

Supplementary Table 48. Table continued from previous page.

Run		MAS1	KIG12-15	IK-99-237	CCM-1
Log clock, all dated, constant pop size	Median	13410.5615	1.68E+05	1.67E+05	74928.8047
	Maximum	13408.3125	103562.5	107187.5	74707.5
	95% HPD	[13277.8794, 13537.5241]	[50001.3774, 4.1709E5]	[50000.1366, 4.1648E5]	[70055.3802, 79841.0784]
Strict clock, all dated, constant pop size	Median	13410.6836	1.30E+05	1.31E+05	75012.3967
	Maximum	13410.8125	120562.5	117312.5	74787.5
	95% HPD	[13280.142, 13539.9312]	[52535.9673, 2.2618E5]	[52238.3439, 2.2635E5]	[70123.4514, 79912.707]
Log clock, all dated, Skyline	Median	13410.6713	1.76E+05	1.76E+05	74908.9744
	Maximum	13410.5625	133062.5	121187.5	74847.5
	95% HPD	[13281.7943, 13541.9021]	[50003.6885, 3.911E5]	[50005.6279, 3.9193E5]	[70041.5527, 79841.7678]
Strict clock, all dated, Skyline	Median	1.34E+04	142000	1.42E+05	7.50E+04
	Maximum	13403.9375	133812.5	131437.5	75032.5
	95% HPD	[13279.4816, 13539.1249]	[65031.5712, 2.2917E5]	[66206.1326, 2.3095E5]	[70169.6997, 79963.0412]
Log clock, reduc dated, constant pop size	Median	13411.0566	1.74E+05	1.74E+05	74910.3189
	Maximum	13414.0625	87562.5	91437.5	74867.5
	95% HPD	[13280.7941, 13539.6296]	[50001.3672, 4.5515E5]	[50000.1489, 4.5562E5]	[69961.71, 79713.5425]
Strict clock, reduc dated, constant pop size	Median	13410.9221	1.23E+05	1.23E+05	75033.6341
	Maximum	13415.0625	112312.5	112062.5	75107.5
	95% HPD	[13281.4163, 13541.2394]	[53225.9881, 2.1041E5]	[52308.6047, 2.1017E5]	[70171.8776, 79975.7234]
Log clock, reduc dated, Skyline	Median	13410.0748	1.80E+05	1.80E+05	74903.5671
	Maximum	13405.5625	85437.5	78562.5	74622.5
	95% HPD	[13279.0573, 13539.0263]	[50001.9817, 4.1061E5]	[50002.4817, 4.1253E5]	[70086.7346, 79848.7694]
Strict clock, reduc dated, Skyline	Median	13410.6435	1.28E+05	1.28E+05	75063.3277
	Maximum	13404.0625	121437.5	120312.5	75142.5
	95% HPD	[13281.391, 13540.5744]	[65254.3001, 2.052E5]	[64789.9857, 2.0521E5]	[70144.6118, 79937.2975]

Supplementary Figure 30. Posterior probability distributions of mastodons dated using a lognormal clock, all known temporal provenience sample for calibration, and a constant population size prior. (A) Kernel Density Estimate of undated mastodons. Samples with known dates were excluded to better visualize the probability density along the y-axis. (B) Violin plot showing the 95% HPD of all mastodons in the analysis. Samples appear in order according to the legend.

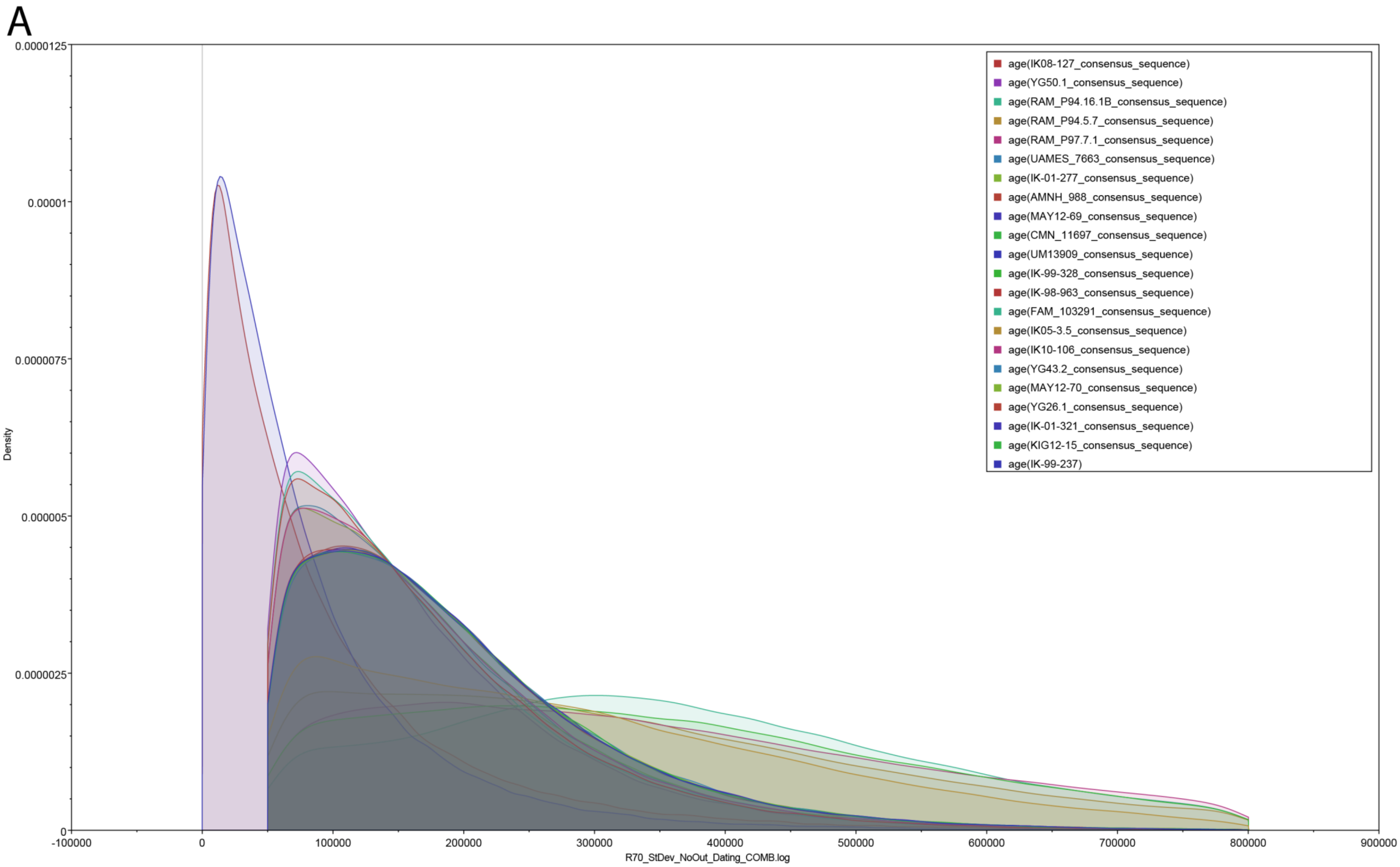

B

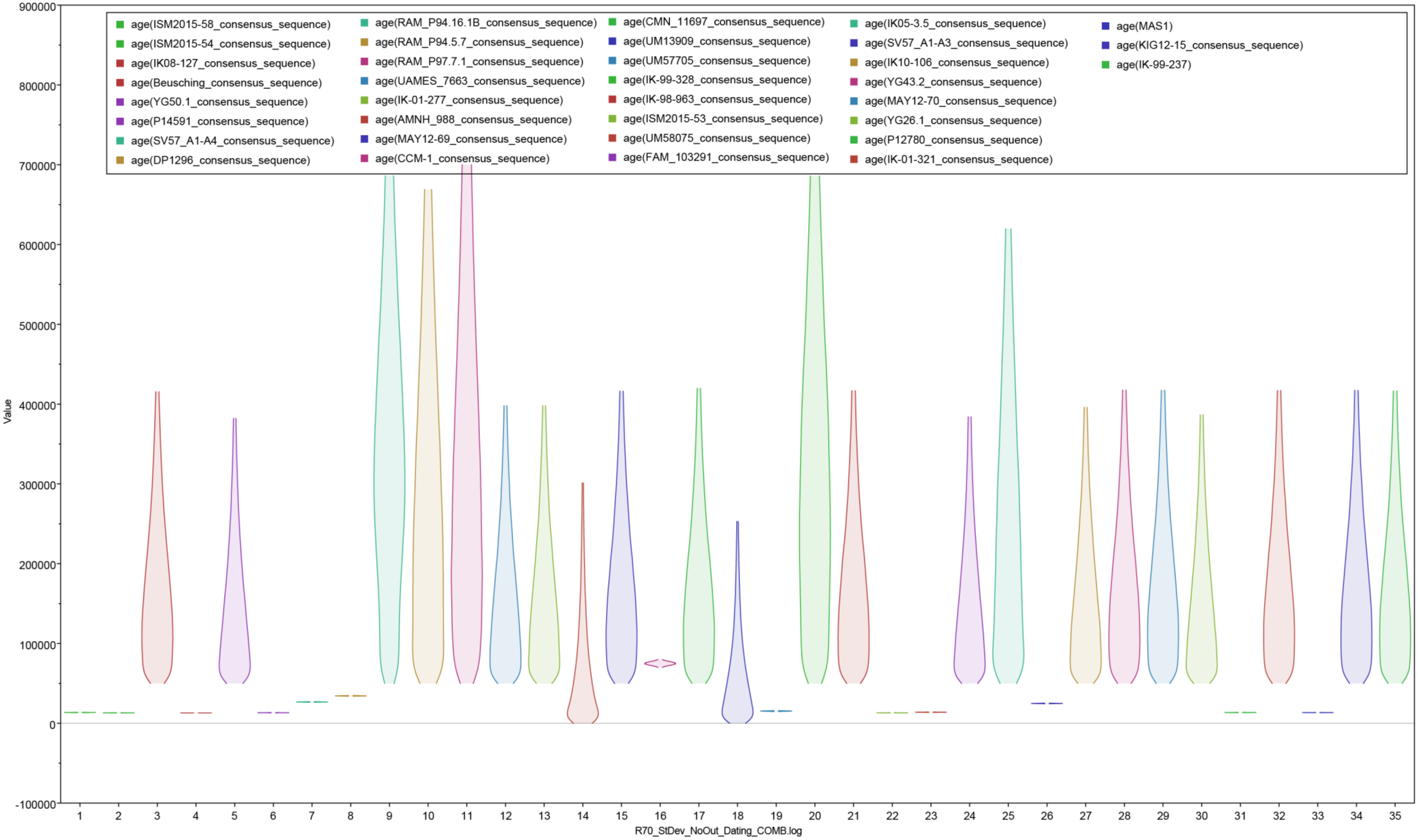
age(ISM2015-58_consensus_sequence)
age(ISM2015-54_consensus_sequence)
age(IK08-127_consensus_sequence)
age(Beusching_consensus_sequence)
age(YG50.1_consensus_sequence)
age(P14591_consensus_sequence)
age(SV57_A1-A4_consensus_sequence)
age(DP1296_consensus_sequence)
age(RAM_P94.16.1B_consensus_sequence)
age(RAM_P94.5.7_consensus_sequence)
age(RAM_P97.7.1_consensus_sequence)
age(UAMES_7663_consensus_sequence)
age(IK-01-277_consensus_sequence)
age(AMNH_988_consensus_sequence)
age(MAY12-69_consensus_sequence)
age(CCM-1_consensus_sequence)
age(CMN_11697_consensus_sequence)
age(UM13909_consensus_sequence)
age(UM57705_consensus_sequence)
age(IK-99-328_consensus_sequence)
age(IK-98-963_consensus_sequence)
age(ISM2015-53_consensus_sequence)
age(UM58075_consensus_sequence)
age(FAM_103291_consensus_sequence)
age(IK05-3.5_consensus_sequence)
age(SV57_A1-A3_consensus_sequence)
age(IK10-106_consensus_sequence)
age(YG43.2_consensus_sequence)
age(MAY12-70_consensus_sequence)
age(YG26.1_consensus_sequence)
age(P12780_consensus_sequence)
age(IK-01-321_consensus_sequence)
age(MAS1)
age(KIG12-15_consensus_sequence)
age(IK-99-237)
Value
900000
800000
700000
600000
500000
400000
300000
200000
100000
0
-100000
1 2 3 4 5 6 7 8 9 10 11 12 13 14 15 16 17 18 19 20 21 22 23 24 25 26 27 28 29 30 31 32 33 34 35
R70_StDev_NoOut_Dating_COMB.log

Supplementary Figure 31. Posterior probability distributions of mastodons dated using a strict clock, all known temporal provenience sample for calibration, and a constant population size prior. (A) Kernel Density Estimate of undated mastodons. Samples with known dates were excluded to better visualize the probability density along the y-axis. (B) Violin plot showing the 95% HPD of all mastodons in the analysis. Samples appear in order according to the legend.

A

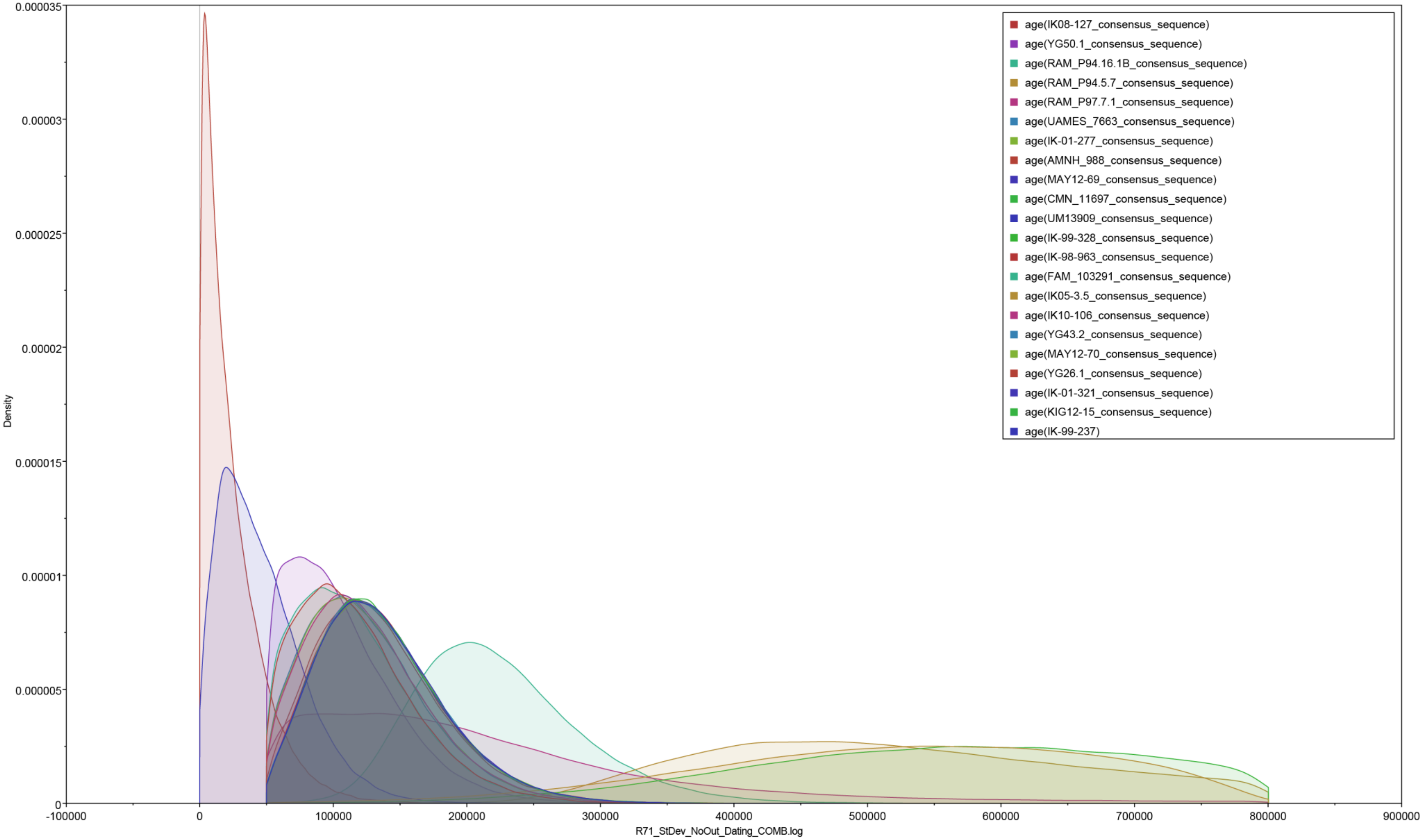

B

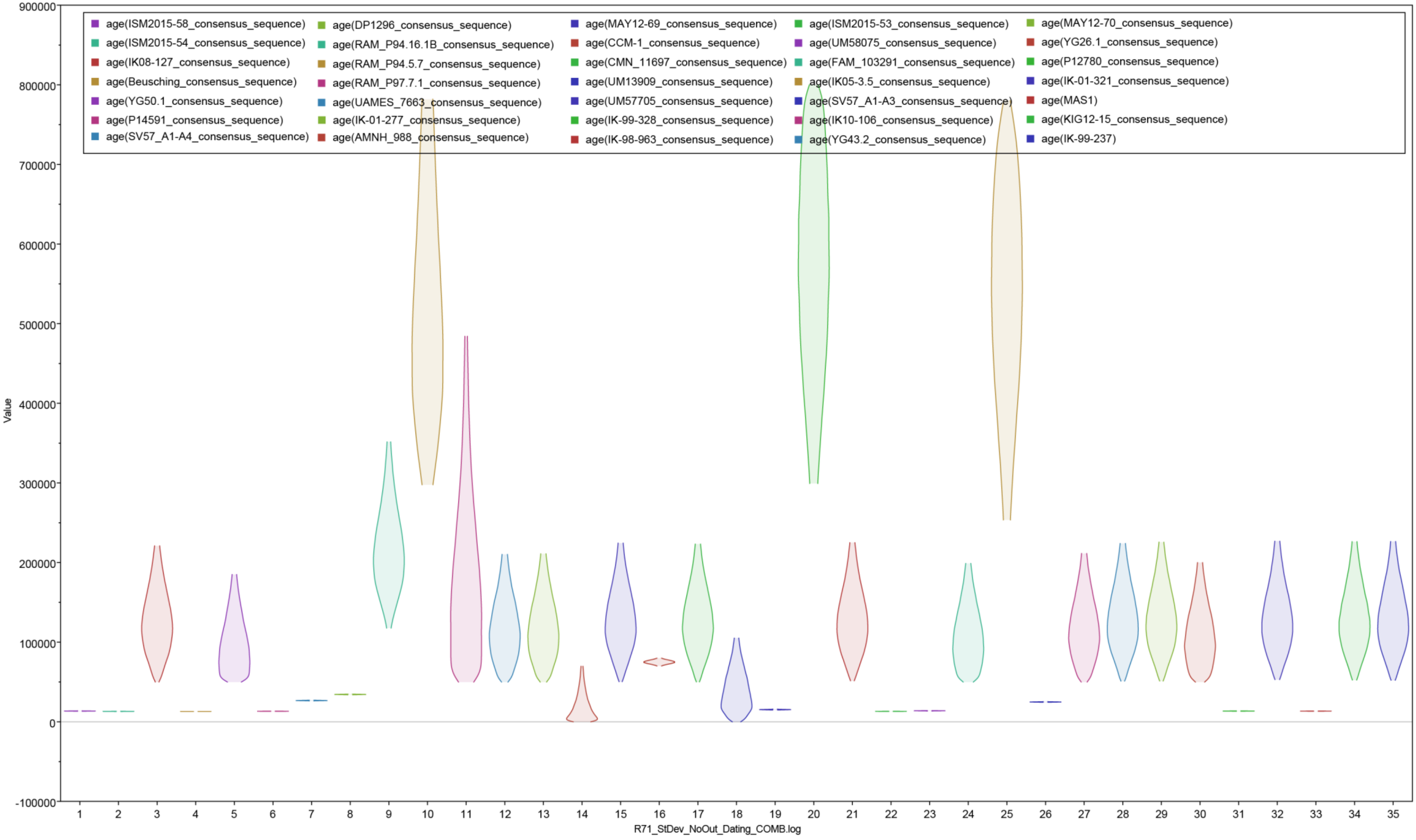

age(ISM2015-58_consensus_sequence)
age(ISM2015-54_consensus_sequence)
age(IK08-127_consensus_sequence)
age(Beusching_consensus_sequence)
age(YG50.1_consensus_sequence)
age(P14591_consensus_sequence)
age(SV57_A1-A4_consensus_sequence)
age(DP1296_consensus_sequence)
age(RAM_P94.16.1B_consensus_sequence)
age(RAM_P94.5.7_consensus_sequence)
age(RAM_P97.7.1_consensus_sequence)
age(UAMES_7663_consensus_sequence)
age(IK-01-277_consensus_sequence)
age(AMNH_988_consensus_sequence)
age(MAY12-69_consensus_sequence)
age(CCM-1_consensus_sequence)
age(CMN_11697_consensus_sequence)
age(UM13909_consensus_sequence)
age(UM57705_consensus_sequence)
age(IK-99-328_consensus_sequence)
age(IK-98-963_consensus_sequence)
age(ISM2015-53_consensus_sequence)
age(UM58075_consensus_sequence)
age(FAM_103291_consensus_sequence)
age(IK05-3.5_consensus_sequence)
age(SV57_A1-A3_consensus_sequence)
age(IK10-106_consensus_sequence)
age(YG43.2_consensus_sequence)
age(MAY12-70_consensus_sequence)
age(YG26.1_consensus_sequence)
age(P12780_consensus_sequence)
age(IK-01-321_consensus_sequence)
age(MAS1)
age(KIG12-15_consensus_sequence)
age(IK-99-237)
900000
800000
700000
600000
500000
400000
300000
200000
100000
0
-100000
Value
1 2 3 4 5 6 7 8 9 10 11 12 13 14 15 16 17 18 19 20 21 22 23 24 25 26 27 28 29 30 31 32 33 34 35
R71_StDev_NoOut_Dating_COMB.log

Supplementary Figure 32. Posterior probability distributions of mastodons dated using a lognormal clock, all known temporal provenience sample for calibration, and a skyline demographic prior with 13 piecewise-constant groups. (A) Kernel Density Estimate of undated mastodons. Samples with known dates were excluded to better visualize the probability density along the y-axis. (B) Violin plot showing the 95% HPD of all mastodons in the analysis. Samples appear in order according to the legend.

A

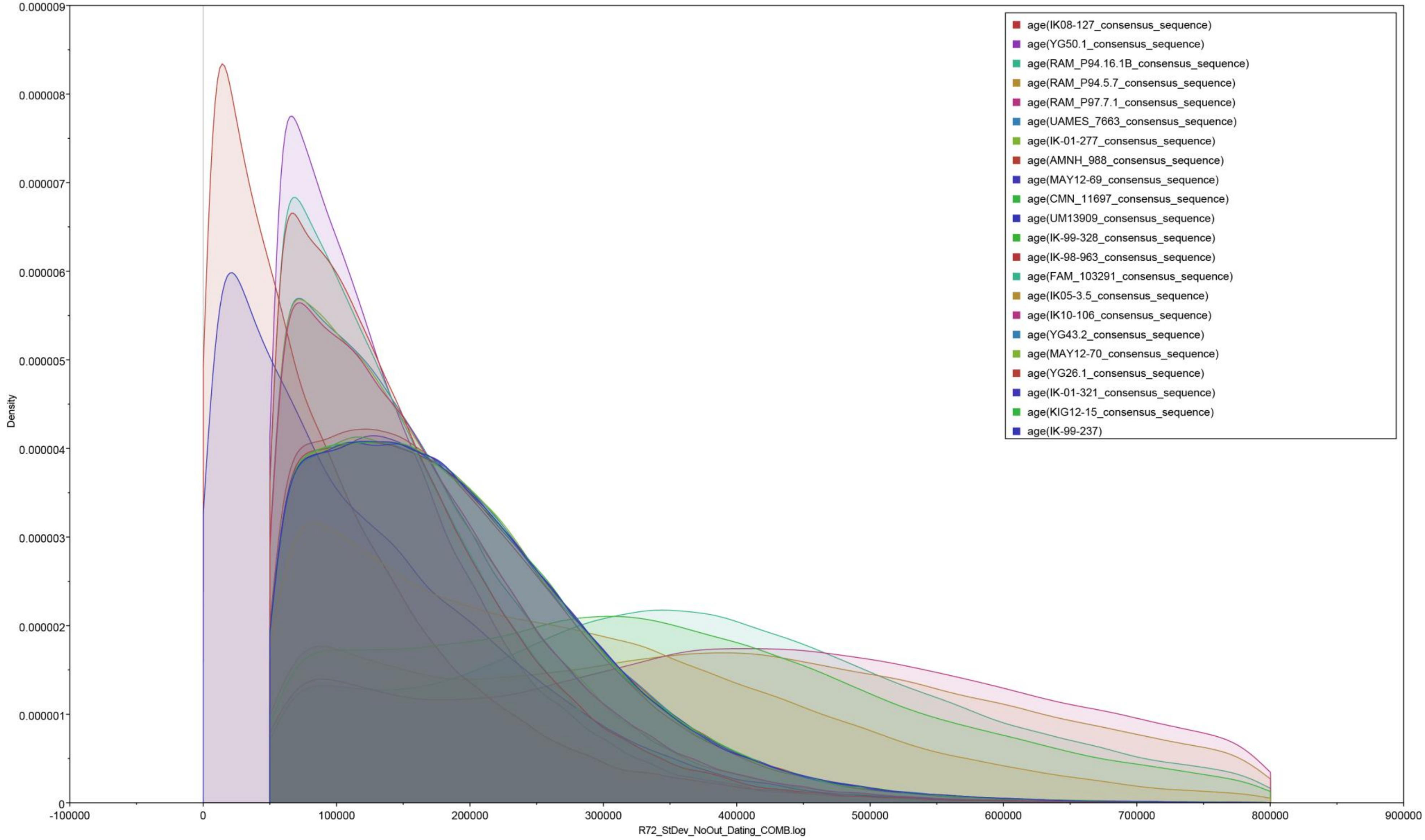

B

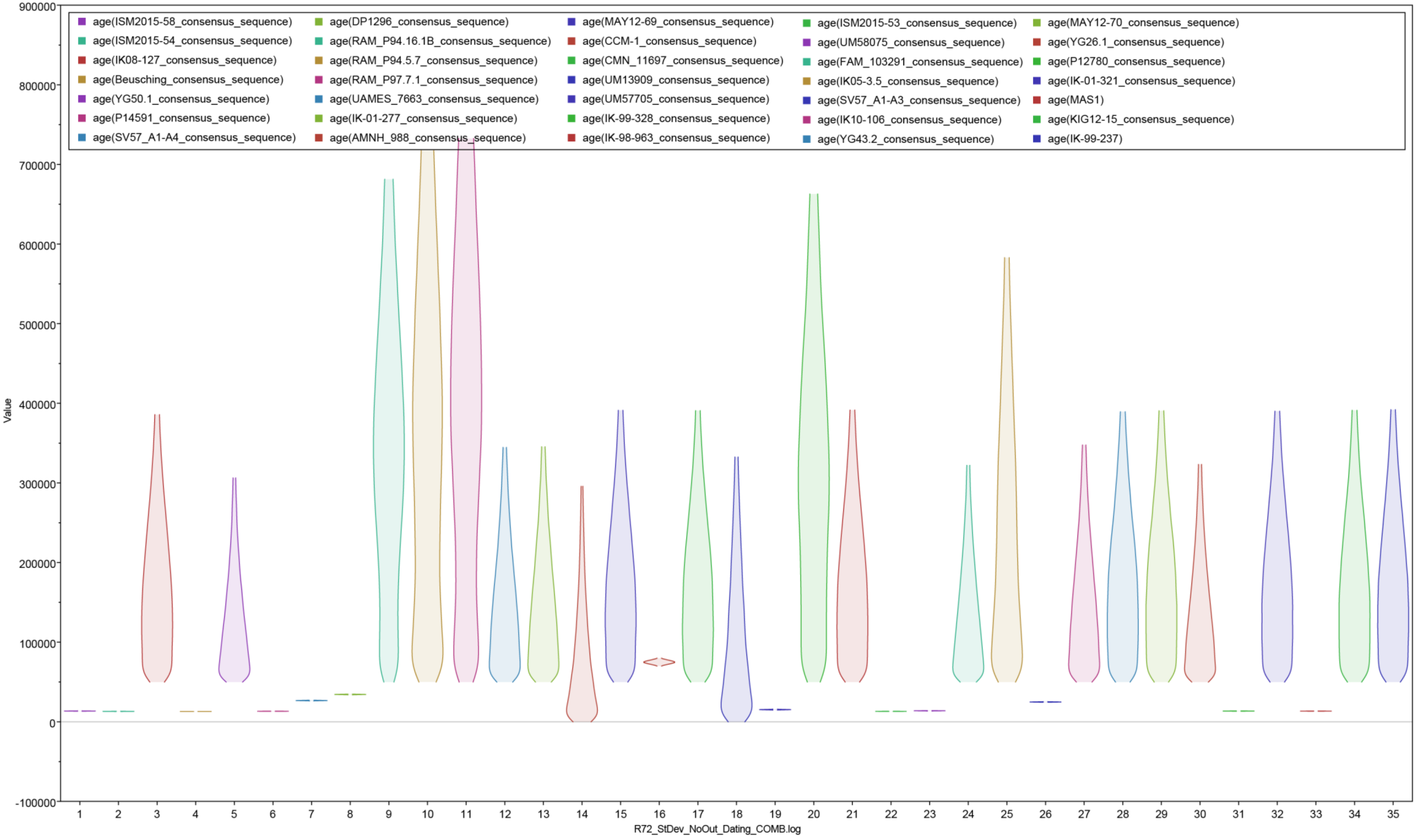

900000
800000
700000
600000
500000
400000
300000
200000
100000
0
-100000
Value
age(ISM2015-58_consensus_sequence)
age(ISM2015-54_consensus_sequence)
age(IK08-127_consensus_sequence)
age(Beusching_consensus_sequence)
age(YG50.1_consensus_sequence)
age(P14591_consensus_sequence)
age(SV57_A1-A4_consensus_sequence)
age(DP1296_consensus_sequence)
age(RAM_P94.16.1B_consensus_sequence)
age(RAM_P94.5.7_consensus_sequence)
age(RAM_P97.7.1_consensus_sequence)
age(UAMES_7663_consensus_sequence)
age(IK-01-277_consensus_sequence)
age(AMNH_988_consensus_sequence)
age(MAY12-69_consensus_sequence)
age(CCM-1_consensus_sequence)
age(CMN_11697_consensus_sequence)
age(UM13909_consensus_sequence)
age(UM57705_consensus_sequence)
age(IK-99-328_consensus_sequence)
age(IK-98-963_consensus_sequence)
age(ISM2015-53_consensus_sequence)
age(UM58075_consensus_sequence)
age(FAM_103291_consensus_sequence)
age(IK05-3.5_consensus_sequence)
age(SV57_A1-A3_consensus_sequence)
age(IK10-106_consensus_sequence)
age(YG43.2_consensus_sequence)
age(MAY12-70_consensus_sequence)
age(YG26.1_consensus_sequence)
age(P12780_consensus_sequence)
age(IK-01-321_consensus_sequence)
age(MAS1)
age(KIG12-15_consensus_sequence)
age(IK-99-237)
1 2 3 4 5 6 7 8 9 10 11 12 13 14 15 16 17 18 19 20 21 22 23 24 25 26 27 28 29 30 31 32 33 34 35
R72_StDev_NoOut_Dating_COMB.log

Supplementary Figure 33. Posterior probability distributions of mastodons dated using a strict clock, all known temporal provenience sample for calibration, and a skyline demographic prior with 13 piecewise-constant groups. (A) Kernel Density Estimate of undated mastodons. Samples with known dates were excluded to better visualize the probability density along the y-axis. (B) Violin plot showing the 95% HPD of all mastodons in the analysis. Samples appear in order according to the legend.

A

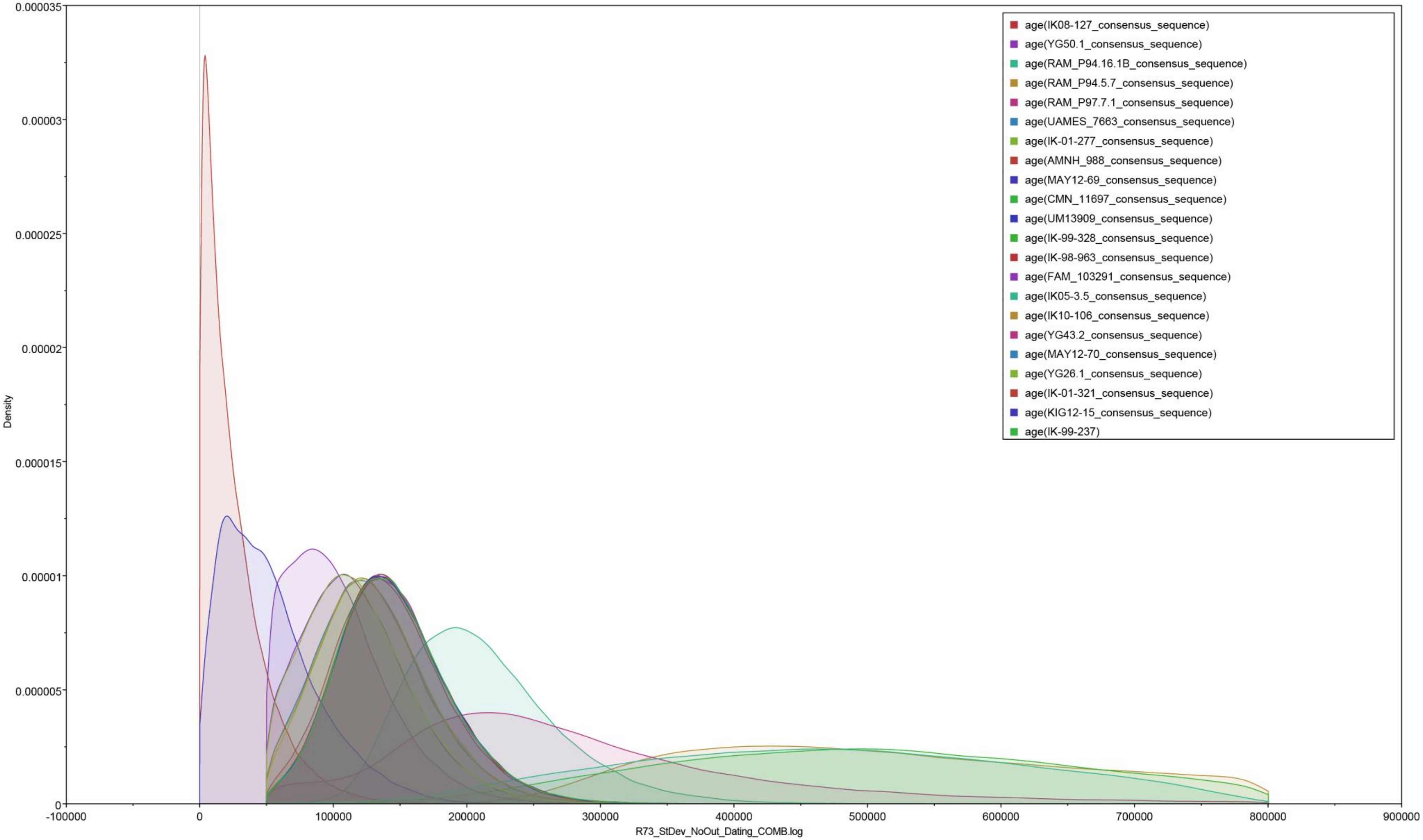

B

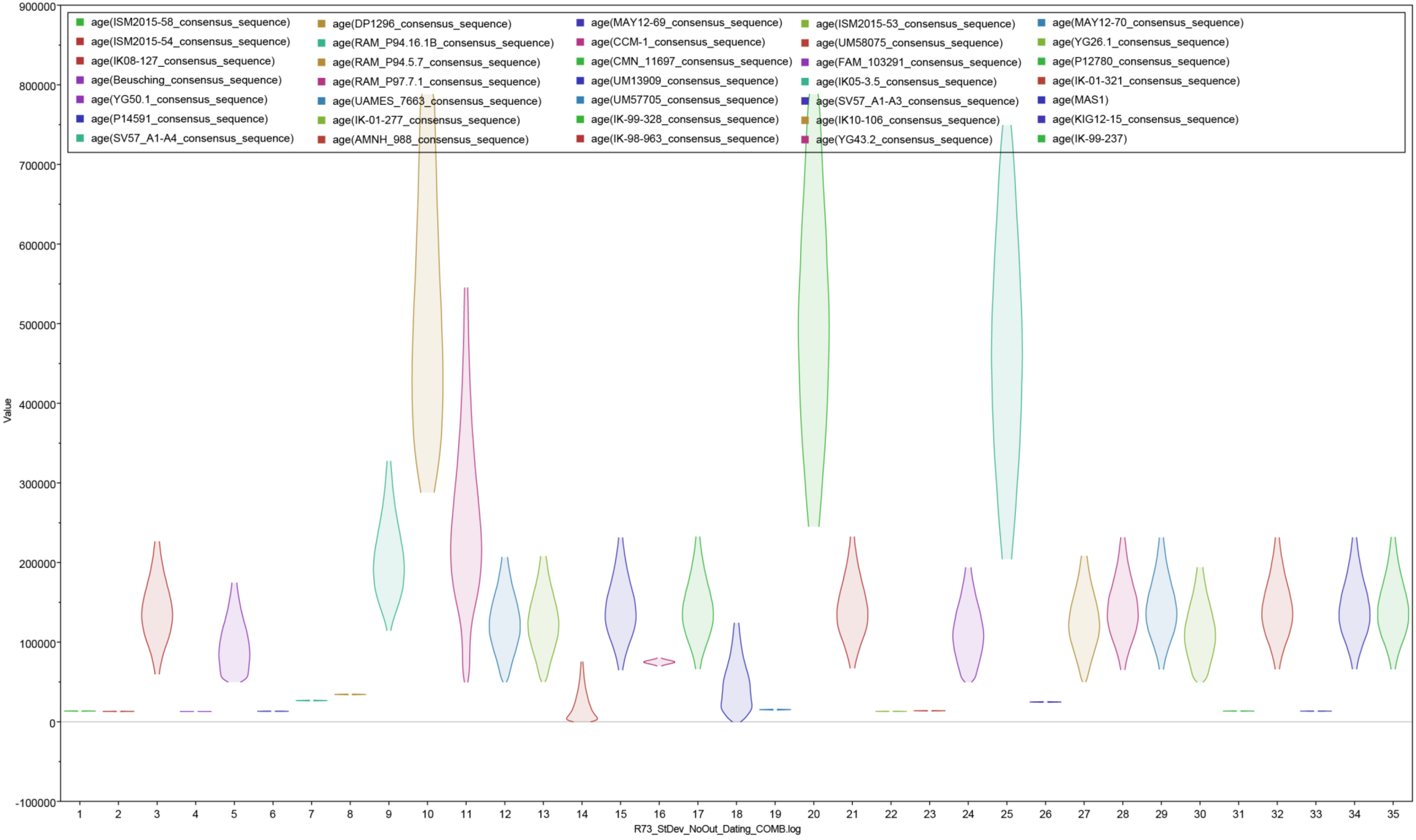

age(ISM2015-58_consensus_sequence)
age(ISM2015-54_consensus_sequence)
age(IK08-127_consensus_sequence)
age(Beusching_consensus_sequence)
age(YG50.1_consensus_sequence)
age(P14591_consensus_sequence)
age(SV57_A1-A4_consensus_sequence)
age(DP1296_consensus_sequence)
age(RAM_P94.16.1B_consensus_sequence)
age(RAM_P94.5.7_consensus_sequence)
age(RAM_P97.7.1_consensus_sequence)
age(UAMES_7663_consensus_sequence)
age(IK-01-277_consensus_sequence)
age(AMNH_988_consensus_sequence)
age(MAY12-69_consensus_sequence)
age(CCM-1_consensus_sequence)
age(CMN_11697_consensus_sequence)
age(UM13909_consensus_sequence)
age(UM57705_consensus_sequence)
age(IK-99-328_consensus_sequence)
age(IK-98-963_consensus_sequence)
age(ISM2015-53_consensus_sequence)
age(UM58075_consensus_sequence)
age(FAM_103291_consensus_sequence)
age(IK05-3.5_consensus_sequence)
age(SV57_A1-A3_consensus_sequence)
age(IK10-106_consensus_sequence)
age(YG43.2_consensus_sequence)
age(MAY12-70_consensus_sequence)
age(YG26.1_consensus_sequence)
age(P12780_consensus_sequence)
age(IK-01-321_consensus_sequence)
age(MAS1)
age(KIG12-15_consensus_sequence)
age(IK-99-237)
Value
900000
800000
700000
600000
500000
400000
300000
200000
100000
0
-100000
1 2 3 4 5 6 7 8 9 10 11 12 13 14 15 16 17 18 19 20 21 22 23 24 25 26 27 28 29 30 31 32 33 34 35
R73_StDev_NoOut_Dating_COMB.log

Supplementary Figure 34. Posterior probability distributions of mastodons dated using a lognormal clock, the reduced dated mastodon set for calibration, and a constant population size prior. (A) Kernel Density Estimate of undated mastodons. Samples with known dates were excluded to better visualize the probability density along the y-axis. (B) Violin plot showing the 95% HPD of all mastodons in the analysis. Samples appear in order according to the legend.

A

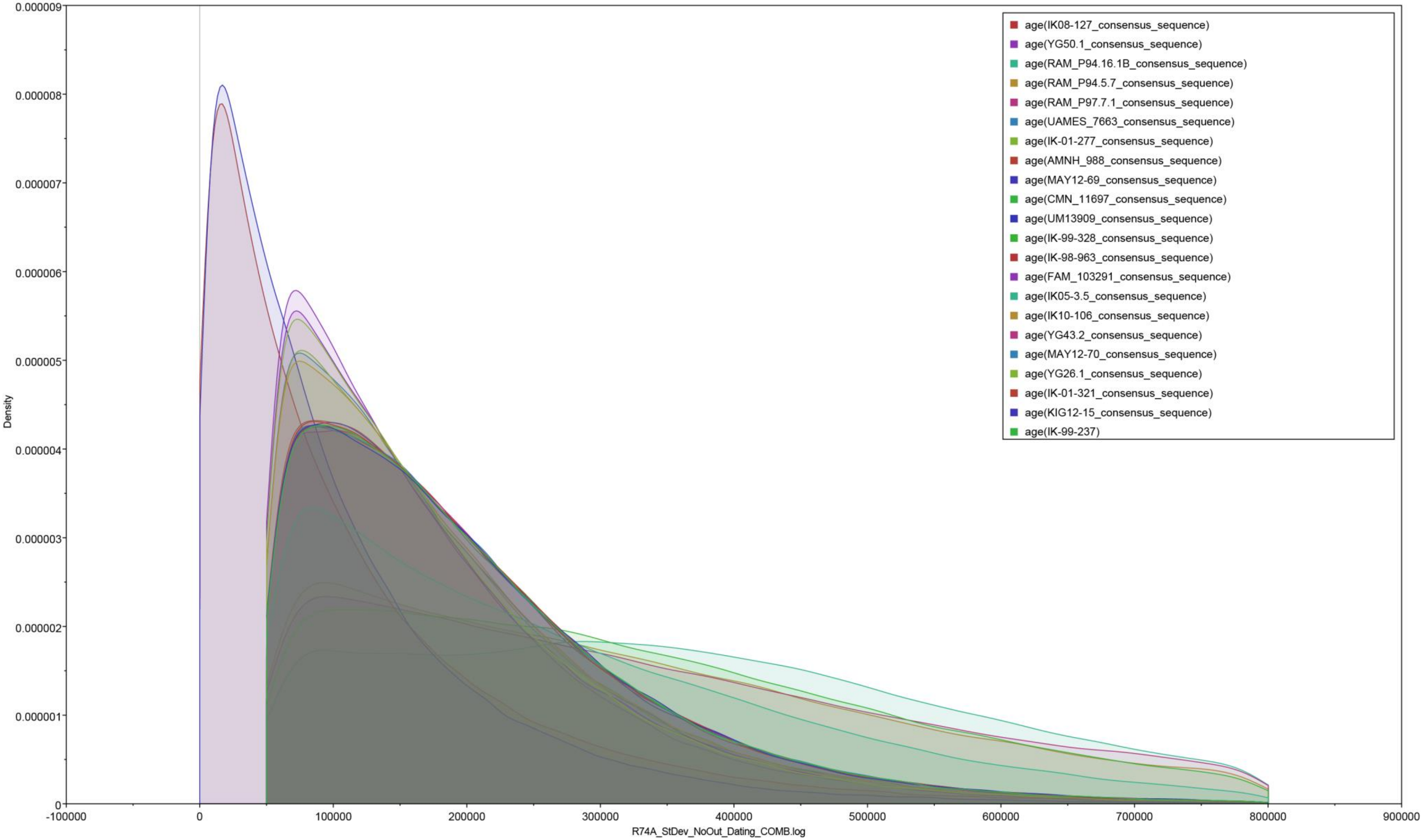

B

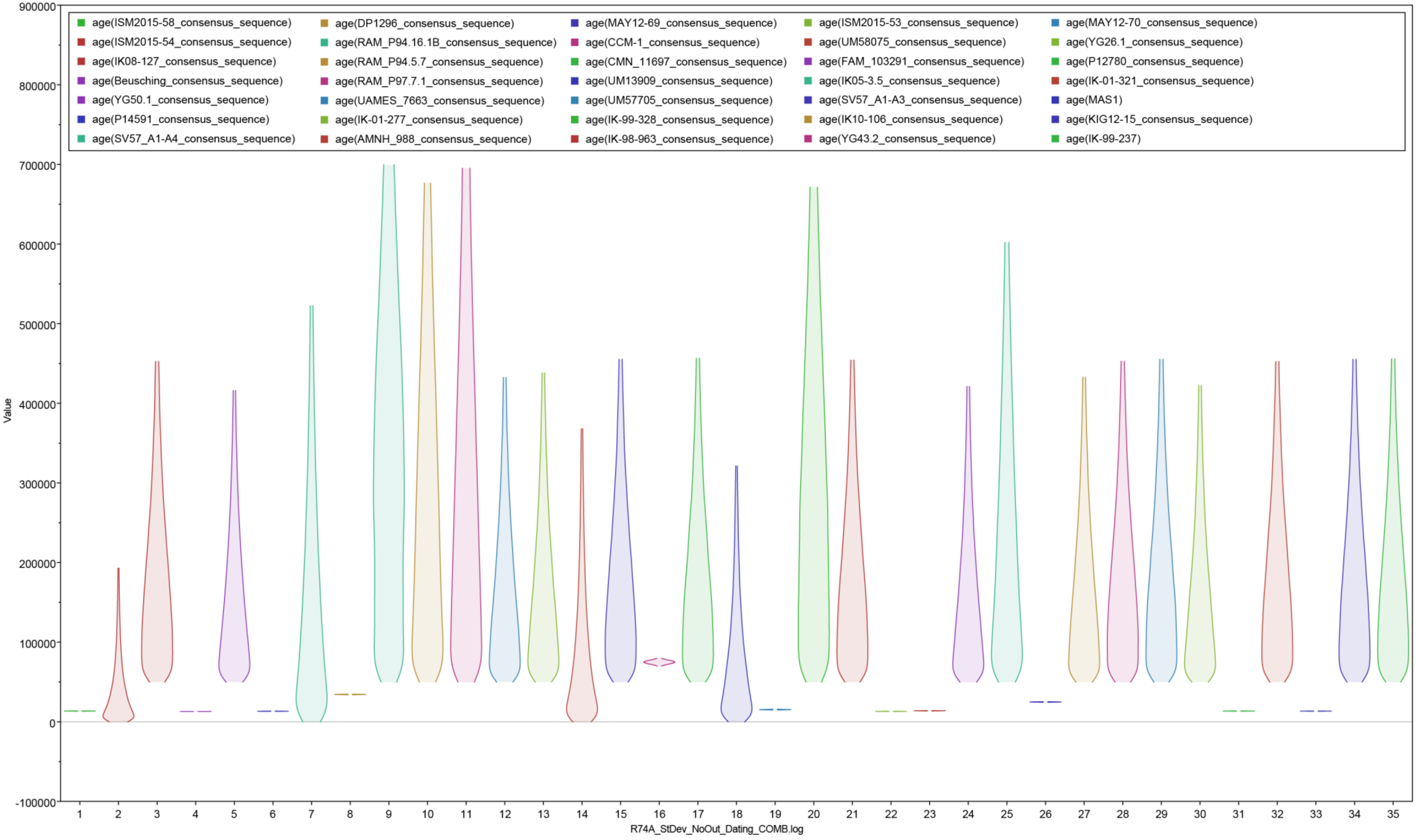
age(ISM2015-58_consensus_sequence)
age(ISM2015-54_consensus_sequence)
age(IK08-127_consensus_sequence)
age(Beusching_consensus_sequence)
age(YG50.1_consensus_sequence)
age(P14591_consensus_sequence)
age(SV57_A1-A4_consensus_sequence)
age(DP1296_consensus_sequence)
age(RAM_P94.16.1B_consensus_sequence)
age(RAM_P94.5.7_consensus_sequence)
age(RAM_P97.7.1_consensus_sequence)
age(UAMES_7663_consensus_sequence)
age(IK-01-277_consensus_sequence)
age(AMNH_988_consensus_sequence)
age(MAY12-69_consensus_sequence)
age(CCM-1_consensus_sequence)
age(CMN_11697_consensus_sequence)
age(UM13909_consensus_sequence)
age(UM57705_consensus_sequence)
age(IK-99-328_consensus_sequence)
age(IK-98-963_consensus_sequence)
age(ISM2015-53_consensus_sequence)
age(UM58075_consensus_sequence)
age(FAM_103291_consensus_sequence)
age(IK05-3.5_consensus_sequence)
age(SV57_A1-A3_consensus_sequence)
age(IK10-106_consensus_sequence)
age(YG43.2_consensus_sequence)
age(MAY12-70_consensus_sequence)
age(YG26.1_consensus_sequence)
age(P12780_consensus_sequence)
age(IK-01-321_consensus_sequence)
age(MAS1)
age(KIG12-15_consensus_sequence)
age(IK-99-237)
Value
900000
800000
700000
600000
500000
400000
300000
200000
100000
0
-100000
1 2 3 4 5 6 7 8 9 10 11 12 13 14 15 16 17 18 19 20 21 22 23 24 25 26 27 28 29 30 31 32 33 34 35
R74A_StDev_NoOut_Dating_COMB.log

Supplementary Figure 35. Posterior probability distributions of mastodons dated using a strict clock, the reduced dated mastodon set for calibration, and a constant population size prior. (A) Kernel Density Estimate of undated mastodons. Samples with known dates were excluded to better visualize the probability density along the y-axis. (B) Violin plot showing the 95% HPD of all mastodons in the analysis. Samples appear in order according to the legend.

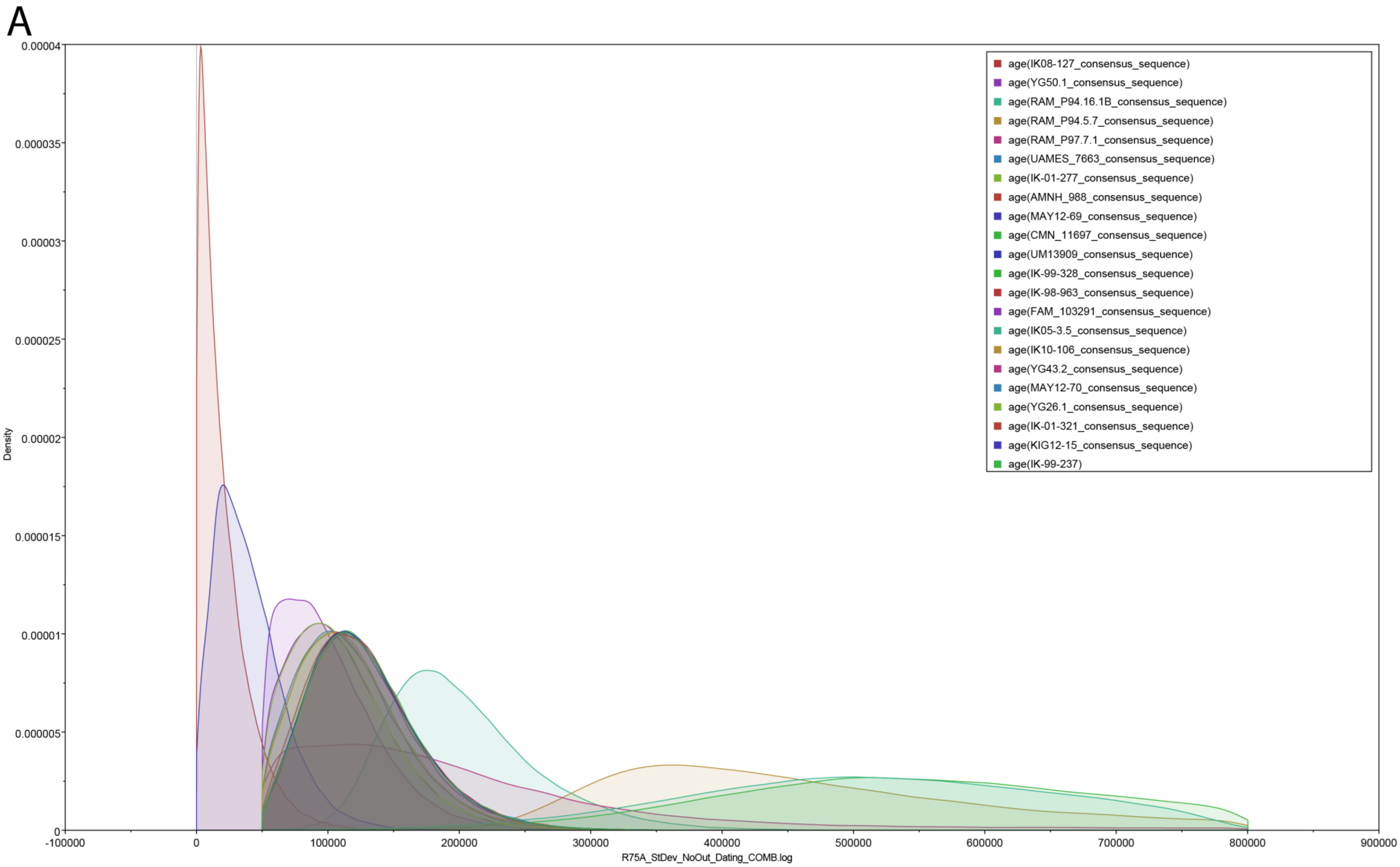

B

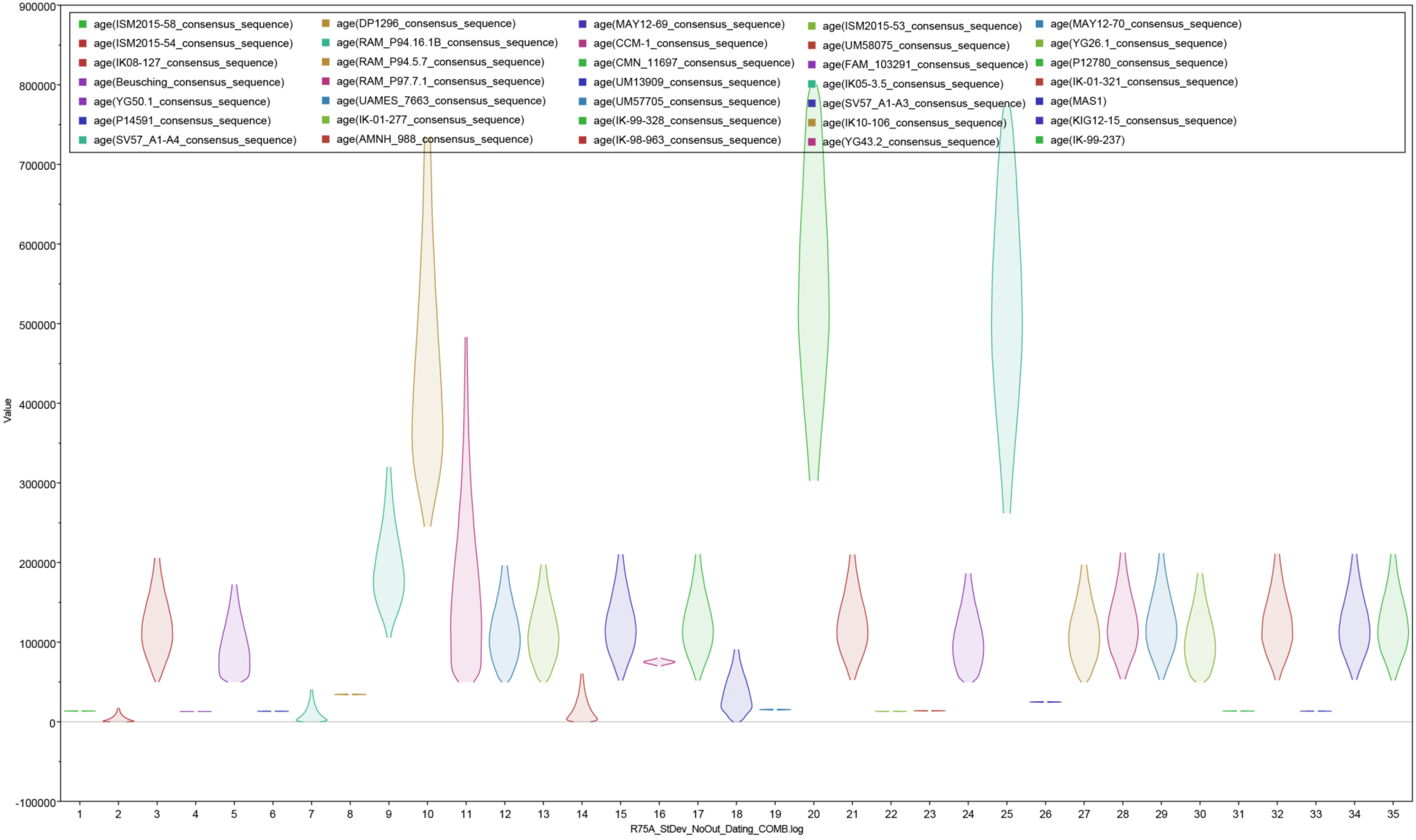
900000
800000
700000
600000
500000
400000
300000
200000
100000
0
-100000
Value
age(ISM2015-58_consensus_sequence)
age(ISM2015-54_consensus_sequence)
age(IK08-127_consensus_sequence)
age(Beusching_consensus_sequence)
age(YG50.1_consensus_sequence)
age(P14591_consensus_sequence)
age(SV57_A1-A4_consensus_sequence)
age(DP1296_consensus_sequence)
age(RAM_P94.16.1B_consensus_sequence)
age(RAM_P94.5.7_consensus_sequence)
age(RAM_P97.7.1_consensus_sequence)
age(UAMES_7663_consensus_sequence)
age(IK-01-277_consensus_sequence)
age(AMNH_988_consensus_sequence)
age(MAY12-69_consensus_sequence)
age(CCM-1_consensus_sequence)
age(CMN_11697_consensus_sequence)
age(UM13909_consensus_sequence)
age(UM57705_consensus_sequence)
age(IK-99-328_consensus_sequence)
age(IK-98-963_consensus_sequence)
age(ISM2015-53_consensus_sequence)
age(UM58075_consensus_sequence)
age(FAM_103291_consensus_sequence)
age(IK05-3.5_consensus_sequence)
age(SV57_A1-A3_consensus_sequence)
age(IK10-106_consensus_sequence)
age(YG43.2_consensus_sequence)
age(MAY12-70_consensus_sequence)
age(YG26.1_consensus_sequence)
age(P12780_consensus_sequence)
age(IK-01-321_consensus_sequence)
age(MAS1)
age(KIG12-15_consensus_sequence)
age(IK-99-237)
1 2 3 4 5 6 7 8 9 10 11 12 13 14 15 16 17 18 19 20 21 22 23 24 25 26 27 28 29 30 31 32 33 34 35
R75A_StDev_NoOut_Dating_COMB.log

Supplementary Figure 36. Posterior probability distributions of mastodons dated using a lognormal clock, the reduced dated mastodon set for calibration, and a skyline demographic prior with 13 piecewise-constant groups. (A) Kernel Density Estimate of undated mastodons. Samples with known dates were excluded to better visualize the probability density along the y-axis. (B) Violin plot showing the 95% HPD of all mastodons in the analysis. Samples appear in order according to the legend.

A

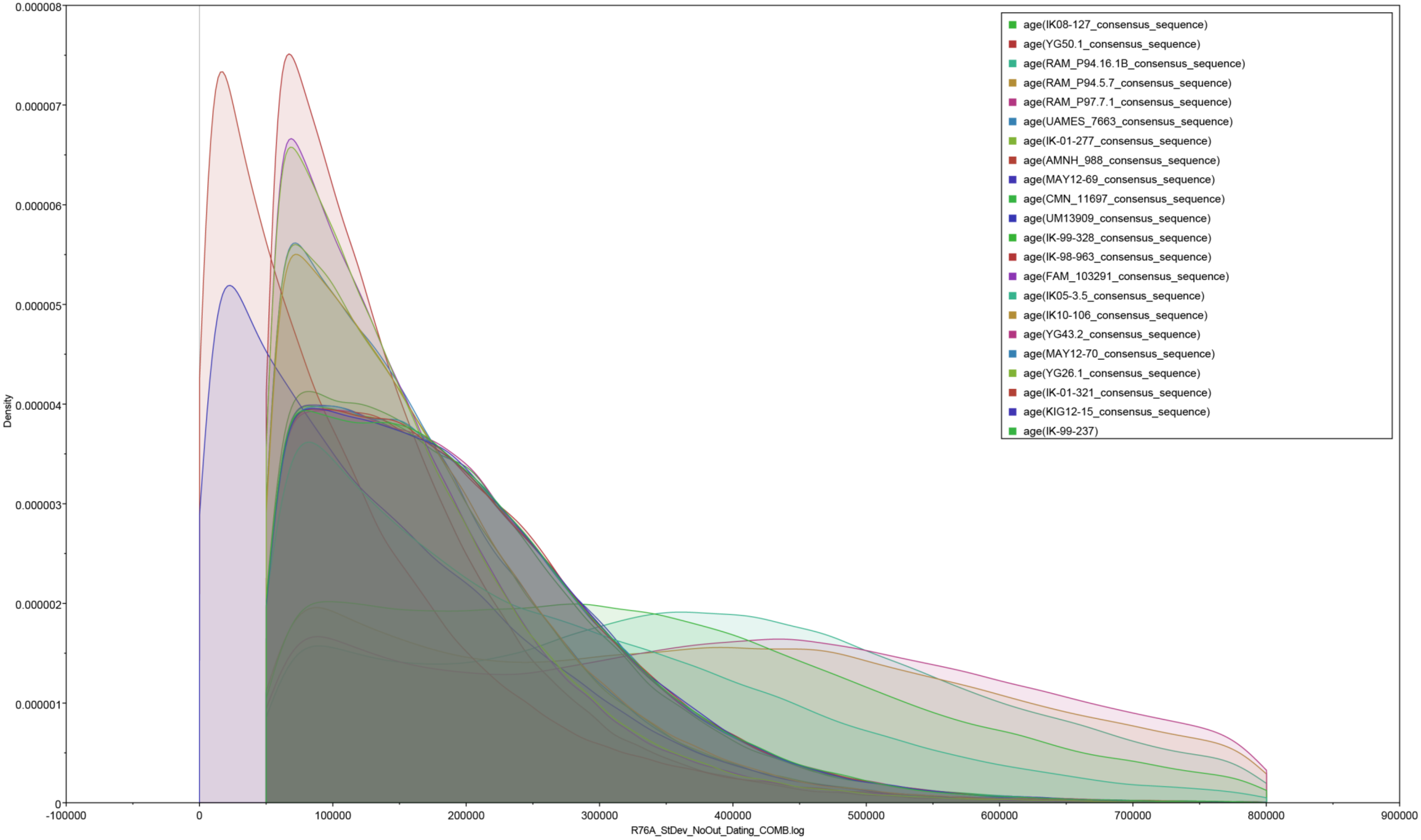

B

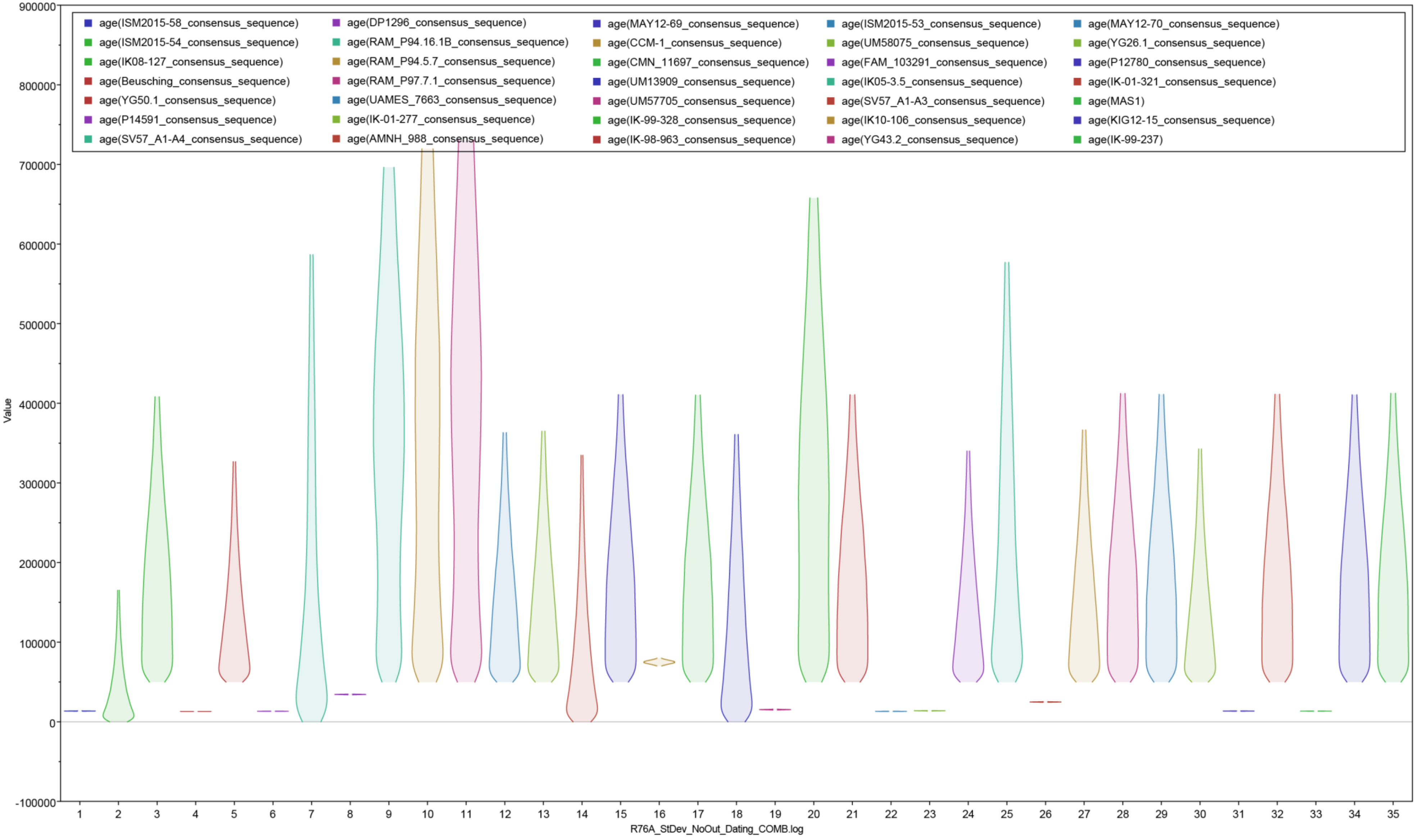
age(ISM2015-58_consensus_sequence)
age(DP1296_consensus_sequence)
age(MAY12-69_consensus_sequence)
age(ISM2015-53_consensus_sequence)
age(MAY12-70_consensus_sequence)
age(ISM2015-54_consensus_sequence)
age(RAM_P94.16.1B_consensus_sequence)
age(CCM-1_consensus_sequence)
age(UM58075_consensus_sequence)
age(YG26.1_consensus_sequence)
age(IK08-127_consensus_sequence)
age(RAM_P94.5.7_consensus_sequence)
age(CMN_11697_consensus_sequence)
age(FAM_103291_consensus_sequence)
age(P12780_consensus_sequence)
age(Beusching_consensus_sequence)
age(RAM_P97.7.1_consensus_sequence)
age(UM13909_consensus_sequence)
age(IK05-3.5_consensus_sequence)
age(IK-01-321_consensus_sequence)
age(YG50.1_consensus_sequence)
age(UAMES_7663_consensus_sequence)
age(UM57705_consensus_sequence)
age(SV57_A1-A3_consensus_sequence)
age(MAS1)
age(P14591_consensus_sequence)
age(IK-01-277_consensus_sequence)
age(IK-99-328_consensus_sequence)
age(IK10-106_consensus_sequence)
age(KIG12-15_consensus_sequence)
age(SV57_A1-A4_consensus_sequence)
age(AMNH_988_consensus_sequence)
age(IK-98-963_consensus_sequence)
age(YG43.2_consensus_sequence)
age(IK-99-237)
Value
900000
800000
700000
600000
500000
400000
300000
200000
100000
0
-100000
1 2 3 4 5 6 7 8 9 10 11 12 13 14 15 16 17 18 19 20 21 22 23 24 25 26 27 28 29 30 31 32 33 34 35
R76A_StDev_NoOut_Dating_COMB.log

Supplementary Figure 37. Posterior probability distributions of mastodons dated using a strict clock, the reduced dated mastodon set for calibration, and a skyline demographic prior with 13 piecewise-constant groups. (A) Kernel Density Estimate of undated mastodons. Samples with known dates were excluded to better visualize the probability density along the y-axis. (B) Violin plot showing the 95% HPD of all mastodons in the analysis. Samples appear in order according to the legend.

A

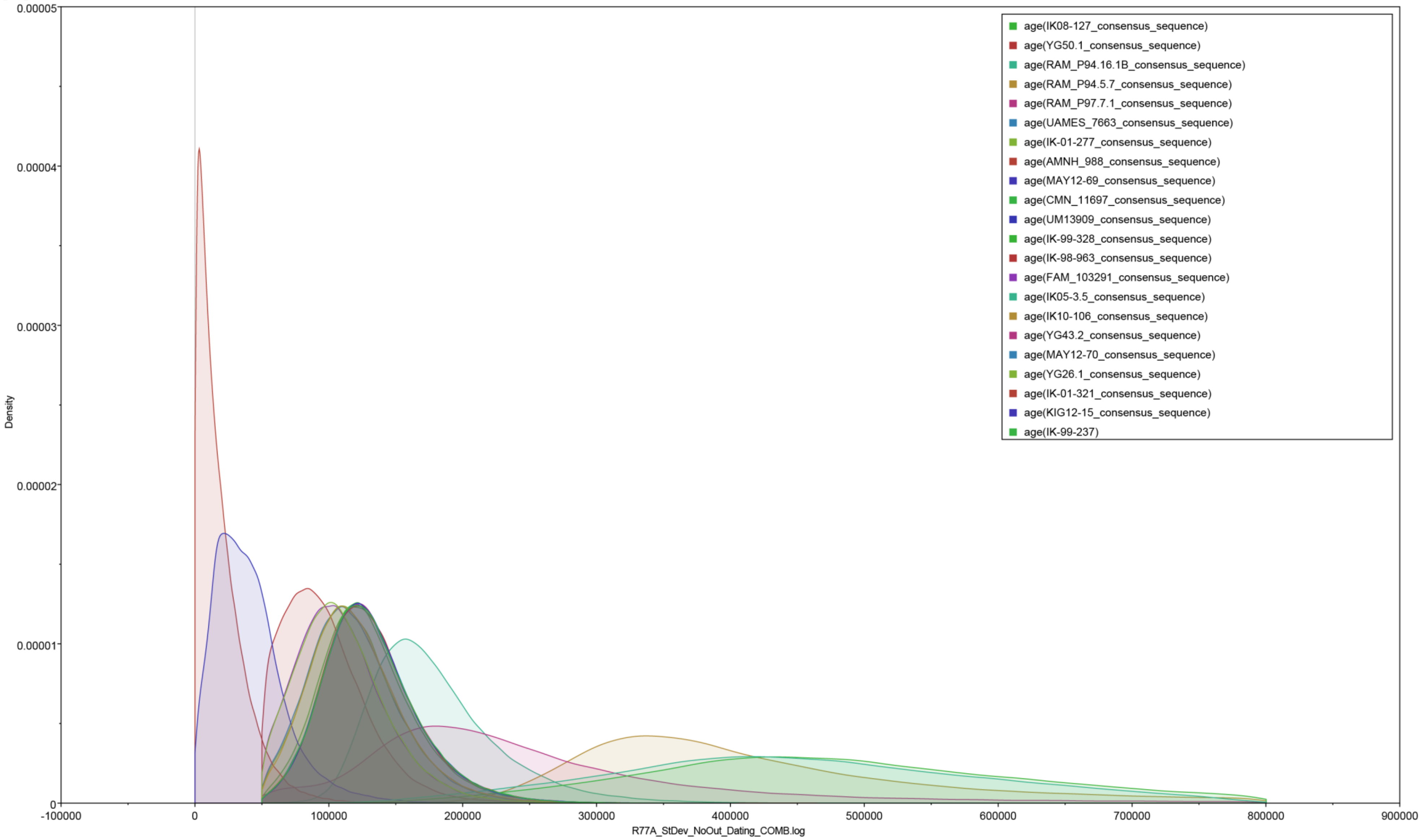

B

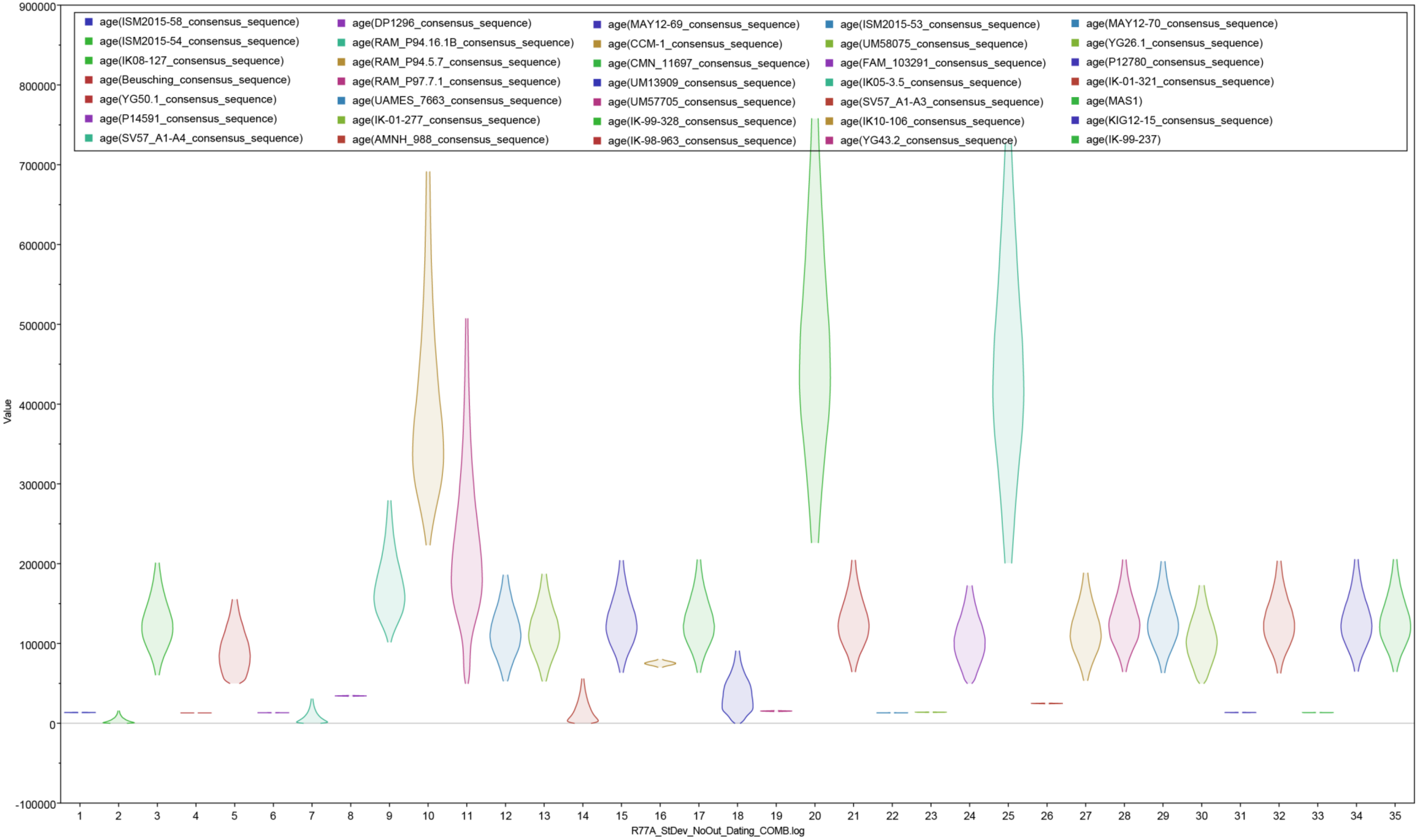
age(ISM2015-58_consensus_sequence)
age(ISM2015-54_consensus_sequence)
age(IK08-127_consensus_sequence)
age(Beusching_consensus_sequence)
age(YG50.1_consensus_sequence)
age(P14591_consensus_sequence)
age(SV57_A1-A4_consensus_sequence)
age(DP1296_consensus_sequence)
age(RAM_P94.16.1B_consensus_sequence)
age(RAM_P94.5.7_consensus_sequence)
age(RAM_P97.7.1_consensus_sequence)
age(UAMES_7663_consensus_sequence)
age(IK-01-277_consensus_sequence)
age(AMNH_988_consensus_sequence)
age(MAY12-69_consensus_sequence)
age(CCM-1_consensus_sequence)
age(CMN_11697_consensus_sequence)
age(UM13909_consensus_sequence)
age(UM57705_consensus_sequence)
age(IK-99-328_consensus_sequence)
age(IK-98-963_consensus_sequence)
age(ISM2015-53_consensus_sequence)
age(UM58075_consensus_sequence)
age(FAM_103291_consensus_sequence)
age(IK05-3.5_consensus_sequence)
age(SV57_A1-A3_consensus_sequence)
age(IK10-106_consensus_sequence)
age(YG43.2_consensus_sequence)
age(MAY12-70_consensus_sequence)
age(YG26.1_consensus_sequence)
age(P12780_consensus_sequence)
age(IK-01-321_consensus_sequence)
age(MAS1)
age(KIG12-15_consensus_sequence)
age(IK-99-237)
Value
900000
800000
700000
600000
500000
400000
300000
200000
100000
0
-100000
1 2 3 4 5 6 7 8 9 10 11 12 13 14 15 16 17 18 19 20 21 22 23 24 25 26 27 28 29 30 31 32 33 34 35
R77A_StDev_NoOut_Dating_COMB.log

Bayesian Skylines

To test whether our data contains any signals of past demographic changes we also constructed Bayesian Skyline plots in BEAST v1.8.0. The same runs were used ass for tip dating (Supplementary Methods – Molecular Clock Dating) and under all parameters (i.e., using both clock models and both datasets for calibration, except with the tree prior changed to a piecewise-constant Bayesian Skyline model with 13 groups). All analyses were run in triplicate to ensure consistency, and subsequently combined for the final analyses.

All skyline plots show a similar trend with essentially no substantial change in the population history regardless of the clock model or whether all mastodons or the reduced dated mastodon datasets were used for calibration (Supplementary Fig. 38). Despite some movement in the median estimated effective population size, estimates remain within or very close to the 95% HPD interval.

Supplementary Figure 38. Bayesian skyline plots reconstructing the demographic history of American mastodons in this study. The blue line shows median effective population size through time, while the shaded area indicates the 95% HPD interval. Skyline plots using all dated mastodons from calibration with a lognormal (A) or strict (B) clock. Skyline plots using the reduced dated mastodon dataset for calibration with a lognormal (C) or strict (D) clock.

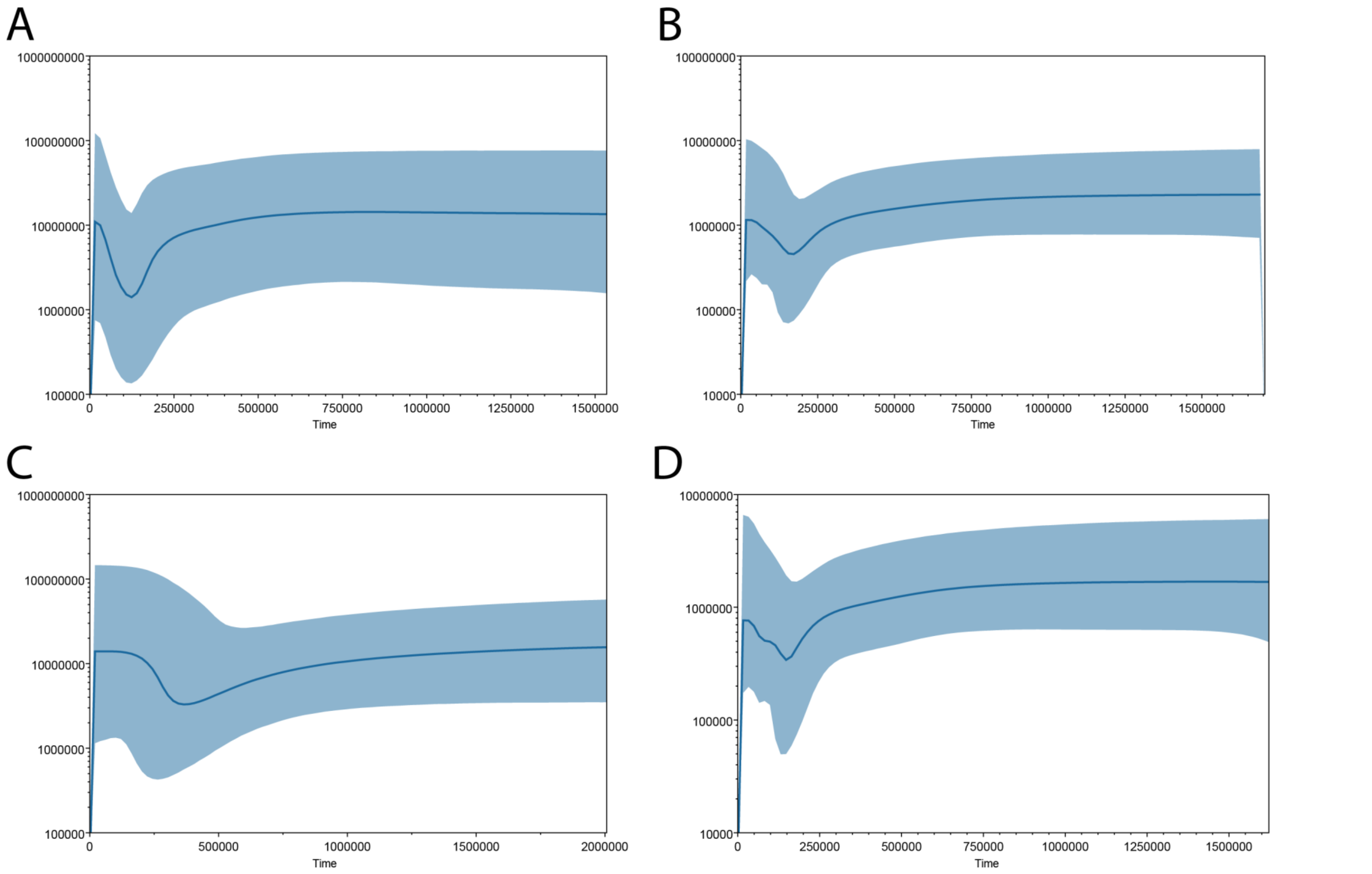

Supplementary References

1. Dabney, J. *et al.* Complete mitochondrial genome sequence of a Middle Pleistocene cave bear reconstructed from ultrashort DNA fragments. *Proc. Natl. Acad. Sci. U. S. A.* **110**, 15758–15763 (2013).
2. Glocke, I. & Meyer, M. Extending the spectrum of DNA sequences retrieved from ancient bones and teeth. *Genome Res.* **27**, 1–8 (2017).
3. Meyer, M. & Kircher, M. Illumina Sequencing Library Preparation for Highly Multiplexed Target Capture and Sequencing. *Cold Spring Harb. Protoc.* **2010**, 1–10 (2010).
4. Kircher, M., Sawyer, S. & Meyer, M. Double indexing overcomes inaccuracies in multiplex sequencing on the Illumina platform. *Nucleic Acids Res.* **40**, 1–8 (2012).
5. Gansauge, M.-T. & Meyer, M. Single-stranded DNA library preparation for the sequencing of ancient or damaged DNA. *Nat. Protoc.* **8**, 737–748 (2013).
6. Gansauge, M.-T. *et al.* Single-stranded DNA library preparation from highly degraded DNA using T4 DNA ligase. *Nucleic Acids Res.* **45**, 1–10 (2017).
7. Karpinski, E., Mead, J. I. & Poinar, H. N. Molecular identification of paleofeces from Bechan Cave, southeastern Utah, USA. *Quat. Int.* **443**, 140–146 (2016).
8. Enk, J. *et al.* Mammuthus population dynamics in Late Pleistocene North America: Divergence, Phylogeogrpaphy and Introgression. *Front. Ecol. Evol.* **4**, 1–13 (2016).
9. Renaud, G., Stenzel, U. & Kelso, J. leeHom: adaptor trimming and merging for Illumina sequencing reads. *Nucleic Acids Res.* 1–7 (2014). doi: https://doi.org/10.1093/nar/gku699
10. Li, H. & Durbin, R. Fast and accurate short read alignment with Burrows-Wheeler transform. *Bioinformatics* **25**, 1754–60 (2009).
11. Lehwark, P. & Greiner, S. GB2sequin - A file converter preparing custom GenBank files for database submission. *Genomics* **111**, 759–761 (2019).
12. Jónsson, H., Ginolhac, A., Schubert, M., Johnson, P. L. F. & Orlando, L. MapDamage2.0: Fast approximate Bayesian estimates of ancient DNA damage parameters. *Bioinformatics* **29**, 1682–1684 (2013).
13. Edgar, R. C. MUSCLE: Multiple sequence alignment with high accuracy and high throughput. *Nucleic Acids Res.* **32**, 1792–1797 (2004).
14. Darriba, D., Taboada, G. L., Doallo, R. & Posada, D. jModelTest 2: more models, new heuristics and parallel computing. *Nat. Methods* **9**, 772–772 (2012).
15. Nguyen, L. T., Schmidt, H. A., Von Haeseler, A. & Minh, B. Q. IQ-TREE: A fast and effective stochastic algorithm for estimating maximum-likelihood phylogenies. *Mol. Biol. Evol.* **32**, 268–274 (2015).
16. Drummond, A. J., Suchard, M. a, Xie, D. & Rambaut, A. Bayesian phylogenetics with BEAUti and the BEAST 1.7. *Mol. Biol. Evol.* **29**, 1969–73 (2012).
17. Huson, D. H. *et al.* MEGAN Community Edition - Interactive Exploration and Analysis of Large-Scale Microbiome Sequencing Data. *PLoS Comput. Biol.* **12**, 1–12 (2016).

18. Paradis, E., Claude, J. & Strimmer, K. APE: Analyses of phylogenetics and evolution in R language. *Bioinformatics* **20**, 289–290 (2004).

19. Shapiro, B. *et al.* A Bayesian Phylogenetic Method to Estimate Unknown Sequence Ages. *Mol. Biol. Evol.* **28**, 879–887 (2011).

20. Duchene, S. *et al.* Bayesian Evaluation of Temporal Signal in Measurably Evolving Populations. *Mol. Biol. Evol.* msaa163 (2020). doi: https://doi.org/10.1093/molbev/msaa163

21. Suchard, M. A. *et al.* Bayesian phylogenetic and phylodynamic data integration using BEAST 1.10. *Virus Evol.* **4**, 1–5 (2018).

22. Baele, G., Lemey, P. & Suchard, M. A. Genealogical Working Distributions for Bayesian Model Testing with Phylogenetic Uncertainty. *Syst. Biol.* **65**, 250–264 (2016).

23. Kass, R. E. & Raftery, A. E. Bayes Factors. *J. Am. Stat. Assoc.* **90**, 773–795 (1995).

24. Drummond, A. J. & Bouckaert, R. R. *Bayesian Evolutionary Analysis with BEAST*. *Bayesian Evolutionary Analysis with BEAST* (Cambridge University Press, 2015). doi: https://doi.org/10.1017/CBO9781139095112

25. Molak, M., Lorenzen, E. D., Shapiro, B. & Ho, S. Y. W. Phylogenetic Estimation of Timescales Using Ancient DNA: The Effects of Temporal Sampling Scheme and Uncertainty in Sample Ages. *Mol. Biol. Evol.* **30**, 253–262 (2013).

26. Wickham, H. *ggplot2: Elegant Graphics for Data Analysis*. (Springer-Verlag New York, 2016).

27. Bronk Ramsey, C., Higham, T., Bowles, A. & Hedges, R. Improvements to the Pretreatment of Bone at Oxford. *Radiocarbon* **46**, 155–163 (2004).

28. Bronk Ramsey, C., Higham, T. & Leach, P. Towards High-Precision AMS: Progress and Limitations. *Radiocarbon* **46**, 17–24 (2004).

29. Ramsey, C. B., Higham, T. F. G., Owen, D. C., Pike, A. W. G. & Hedges, R. E. M. Radiocarbon Dates from the Oxford Ams System: Archaeometry Datelist 31. *Archaeometry* **44**, 1–150 (2002).

30. Rambaut, A., Lam, T. T., Max Carvalho, L. & Pybus, O. G. Exploring the temporal structure of heterochronous sequences using TempEst (formerly Path-O-Gen). *Virus Evol.* **2**, 1–7 (2016).

31. Stuiver, M., Reimer, P. J. & Reimer, R. W. CALIB 7.1 [WWW program]. (2019).

32. Baele, G. *et al.* Improving the accuracy of demographic and molecular clock model comparison while accommodating phylogenetic uncertainty. *Mol. Biol. Evol.* **29**, 2157–67 (2012).

33. Rohland, N. *et al.* Proboscidean mitogenomics: Chronology and mode of elephant evolution using mastodon as outgroup. *PLoS Biol.* **5**, 1663–1671 (2007).
