# Supplementary material for: American mastodon mitochondrial genomes suggest multiple dispersal events in response to Pleistocene climate oscillations
Source: Nat Commun. 2020 Sep 1;11:4048. doi: 10.1038/s41467-020-17893-z (PMC7463256; doi:10.1038/s41467-020-17893-z)
Supplement: Supplementary file 3 — Reporting Summary [file 41467_2020_17893_MOESM3_ESM.pdf]

nature research

Corresponding author(s): Emil Karpinski, Hendrik Poinar

Last updated by author(s): Jul 15, 2020

Reporting Summary

Nature Research wishes to improve the reproducibility of the work that we publish. This form provides structure for consistency and transparency in reporting. For further information on Nature Research policies, see Authors & Referees and the Editorial Policy Checklist .

Statistics

For all statistical analyses, confirm that the following items are present in the figure legend, table legend, main text, or Methods section.

n/a	Confirmed	
☐	☒	The exact sample size (n) for each experimental group/condition, given as a discrete number and unit of measurement
☒	☐	A statement on whether measurements were taken from distinct samples or whether the same sample was measured repeatedly
☒	☐	The statistical test(s) used AND whether they are one- or two-sided<br>*Only common tests should be described solely by name; describe more complex techniques in the Methods section.*
☒	☐	A description of all covariates tested
☐	☒	A description of any assumptions or corrections, such as tests of normality and adjustment for multiple comparisons
☐	☒	A full description of the statistical parameters including central tendency (e.g. means) or other basic estimates (e.g. regression coefficient) AND variation (e.g. standard deviation) or associated estimates of uncertainty (e.g. confidence intervals)
☒	☐	For null hypothesis testing, the test statistic (e.g. F, t, r) with confidence intervals, effect sizes, degrees of freedom and P value noted<br>*Give P values as exact values whenever suitable.*
☐	☒	For Bayesian analysis, information on the choice of priors and Markov chain Monte Carlo settings
☒	☐	For hierarchical and complex designs, identification of the appropriate level for tests and full reporting of outcomes
☒	☐	Estimates of effect sizes (e.g. Cohen's d, Pearson's r), indicating how they were calculated

Our web collection on statistics for biologists contains articles on many of the points above.

Software and code

Policy information about availability of computer code

Data collection	No software used during data collection.
Data analysis	Software used: CASAVA (for sequencing runs conducted prior to 2016-08-09); bcl2fastq (for runs conducted on/after 2016-08-09); leeHom v1.1.5; network-aware BWA v0.5.10; samtools v0.1.18; libbam-retrieveMapped_single_and_ProperlyPair; biohazard-bam-rmdup v0.5.14; Geneious v6.1.5; mapDamage 2.0 v2.0.3; MUSCLE v3.8.31; jModelTest v2.1.4; IQ-TREE v1.6.6; BEAUti v1.8.0; BEAST v1.8.0; BLAST v2.9.0; MEGAN v6.12.3; R v3.5.1; TempEst v.1.5.1; CALIB v7.0.4; Tracer v1.7.1; TreeAnnotator v1.8.0; TreeStat v1.8.0; FigTree v1.4.3; ape v5.2. BEAUti v1.10.5 (Prerelease #23570d1); BEAST v1.10.5 (Prerelease #23570d1) . R scripts used to produce nucleotide diversity diagrams are available at: https://github.com/ekarpinski/MastoScripts.

For manuscripts utilizing custom algorithms or software that are central to the research but not yet described in published literature, software must be made available to editors/reviewers. We strongly encourage code deposition in a community repository (e.g. GitHub). See the Nature Research guidelines for submitting code & software for further information.

Data

Policy information about availability of data

All manuscripts must include a data availability statement. This statement should provide the following information, where applicable:

- Accession codes, unique identifiers, or web links for publicly available datasets
- A list of figures that have associated raw data
- A description of any restrictions on data availability

Final consensus sequences for all complete mastodon specimens have been uploaded to NCBI with GenBank accessions MN616941-MN616973. Raw sequencing reads for each complete mastodon were also uploaded to the SRA (BioProject: PRJNA578413). Two previously published American mastodon mitochondrial genomes (GenBank accessions NC_035800 and EF632344) were also analyzed in this study, as well as two mammoth mitochondrial genomes (NC_007596 and NC_015529) to root maximum-likelihood trees.

Field-specific reporting

Please select the one below that is the best fit for your research. If you are not sure, read the appropriate sections before making your selection.

☐ Life sciences ☐ Behavioural & social sciences ☒ Ecological, evolutionary & environmental sciences

For a reference copy of the document with all sections, see nature.com/documents/nr-reporting-summary-flat.pdf

Ecological, evolutionary & environmental sciences study design

All studies must disclose on these points even when the disclosure is negative.

Study description	Phylogeographic analysis of 33 new (+2 previously published) American mastodon (Mammut americnaum) mitochondrial genomes. Studies establishes a continental framework for American mastodon mitochondrial diversity, and sheds light on mastodon range expansion/contractions as a result of glacial/interglacial cycles throughout the Pleistocene.
Research sample	American mastodon samples from across Canada, USA, and Mexico. Specimens analyzed in this study were obtained from participating institutions depending on availability and access of material for destructive analysis. Specimens were chosen without any consideration for sex, age, or other life history variables.
Sampling strategy	Specimens analyzed in this study were obtained from participating institutions depending on availability and access of material for destructive analysis. All specimens which met a predefined completeness threshold (>80% coverage of the American mastodon mitochondrial reference at a minimum depth of 3x) where used in all bioinformatic analyses.
Data collection	Original subsamples of specimens were taken at the institutions housing the specimens, or were repurposed from subsamples taken for other experiments as part of other projects (e.g., radiocarbon analysis). All downstream work was conducted at the McMaster Ancient DNA Centre, with sequencing happening at the McMaster Genome Facility.
Timing and spatial scale	Sequencing data was collected over a span of 5 years as specimens were collected and screened throughout the project, or as methodology improvements allowed for the revisitation of earlier specimens.
Data exclusions	Specimens were removed from downstream wet lab procedures if they failed to produce strong positive results with a qPCR targeting a conserved proboscidean specific region of the 12S gene. Specimens which did not produce enough endogenous reads to reconstruct mitochondrial genomes that met our predefined inclusion threshold (80% coverage of the American mastodon mitochondrial reference at a minimum depth of 3x) were excluded from all bioinformatic analyses.
Reproducibility	No attempts were made to independently reproduce consensus sequences generated from complete American mastodon specimens. However, field standard ancient DNA authenticity criteria was used to ensure the validity of recovered data.
Randomization	Specimens were processed as they were collected over the course of this study. No comparisons were made or are being tested between how specimens were treated during wet lab procedures. Bioinformatic analysis of all specimens was done simultaneously to ensure consistency of procession, program versions, and databases.
Blinding	No direct comparisons or conclusions being drawn between how samples were treated.

Did the study involve field work? ☐ Yes ☒ No

Reporting for specific materials, systems and methods

We require information from authors about some types of materials, experimental systems and methods used in many studies. Here, indicate whether each material, system or method listed is relevant to your study. If you are not sure if a list item applies to your research, read the appropriate section before selecting a response.

Materials & experimental systems

n/a	Involved in the study	
☒	☐	Antibodies
☒	☐	Eukaryotic cell lines
☐	☒	Palaeontology
☒	☐	Animals and other organisms
☒	☐	Human research participants
☒	☐	Clinical data

Methods

n/a	Involved in the study	
☒	☐	ChIP-seq
☒	☐	Flow cytometry
☒	☐	MRI-based neuroimaging

Palaeontology

Specimen provenance	No new specimens were excavated for this study. All analyzed specimens are housed in institutions listed in Supplementary Data 1.
Specimen deposition	Specimens analyzed in this study are were obtained from institutions outlined in Supplementary Data 1 depending on availability and access of material for destructive analysis.
Dating methods	Specimens DP1296, DP885, DP234, DP3727, and DP5247 were sent to the Keck-CCAMS facility at the University of California, Irvine, where they were decalcified in 1 M HCl, gelatinized at 60°C and pH 2, and ultrafiltered to select a high molecular weight fraction (>30kDa).<br><br>Specimens AMNH 982, AMNH 988, AMNH 26834, AMNH 22728, and AMNH 983 were also sent for radiocarbon analysis to the Keck-CCAMS facility at the University of California, Irvine. Bone samples were decalcified using 0.5 M HCl, rinsed with Milli-Q water, hydrolyzed overnight at 60°C with 0.01 M HCl, and the high molecular weight fraction isolated. Cleaned bone samples were additionally sonicated with acetone, methanol, and water to remove unknown consolidants.<br><br>Specimens UAMES 7663 and CCM-1 were sent to the Oxford Radiocarbon Accelerator Unit for radiocarbon analysis. Specimens were prepared and measured as outlined using described in Ramsey et al. [see references 25-27 in SI].

☒ Tick this box to confirm that the raw and calibrated dates are available in the paper or in Supplementary Information.
