# Supplementary material for: American mastodon mitochondrial genomes suggest multiple dispersal events in response to Pleistocene climate oscillations
Source: Nat Commun. 2020 Sep 1;11:4048. doi: 10.1038/s41467-020-17893-z (PMC7463256; doi:10.1038/s41467-020-17893-z)
Supplement: Supplementary file 4 — Description of Additional Supplementary Files [file 41467_2020_17893_MOESM4_ESM.pdf]

Description of Additional Supplementary Files

File name: Supplementary Data 1
Description: Master table showing specimen information and processing steps. Certain specimens are identified using either Field ID or official repository numbers. In such cases both are provided here. Each step was done on the preceding extract/library (to the right) – e.g. libraries 215 and 230 (Rows 4 and 5) were both generated using library prep methodology A from the extract 512, whereas library L684 was generated from extract 512 ME. For specimens with both a collector field number and an official repository number, the official repository number is bolded. References for specimens were provided when the information was known and if the specimen has been formally described in the literature.
