# Supplementary material for: American mastodon mitochondrial genomes suggest multiple dispersal events in response to Pleistocene climate oscillations
Source: Nat Commun. 2020 Sep 1;11:4048. doi: 10.1038/s41467-020-17893-z (PMC7463256; doi:10.1038/s41467-020-17893-z)
Supplement: Supplementary file 2 — Peer Review File [file 41467_2020_17893_MOESM2_ESM.pdf]

Reviewers' comments:

Reviewer #1 (Remarks to the Author):

Karpinski et al present an analysis of Mastodon phylogeography through time based on 33 new, complete mitogenomes.

All experiments and analyses are conducted thoroughly, and I have little doubts about the data itself. Below are a few points that I feel could be improved, though.

Major points:
1. Most conclusions presented in this manuscript rely heavily on the ages estimated for the undated samples using molecular dating. The estimates produce broad 95% HPD intervals, which is not surprising, but which makes their interpretation somewhat speculative. The authors make a case for considering the reasonably tight peaks of the HPD distribution rather than the width of the complete 95% HPD intervals, and while this is a bit arbitrary, I feel it is a fair point to make. However, when so much relies on the molecular estimates, I would like to see how well this molecular age estimation actually performs with this specific dataset. This can for example be tested through "leave-one-out" cross validation of dated samples. I.e, take the dataset as is, but in each iteration, don't just estimate the age of the undated samples, but also the age of one sample for which we know the age. Repeat for all dated samples, and check how often the radiocarbon age of these samples
a) falls within the 95% HPD estimate for the respective sample, and
b) within the arbitrarily defined "peak" of the HPD distribution, and
c) how often the radiocarbon age is within the same MIS as the molecular estimate.
From my experience, this normally works reasonably well, and it really helps to lend some confidence to the results.

2. I find myself slightly skeptical as to how much can be said about the age distribution of clade A from two samples. The results are consistent with an earlier migration of clade A to Beringia compared to clade Y but with so few samples, this interpretation is standing on very shaky legs and is perhaps given a bit too much weight in the discussion and conclusion. And at least with relaxed clock settings, there is a significant overlap between the age of clade A and clade Y samples. Perhaps the above cross validation test will provide further support, though.

Minor points:
Page 6.: When mentioning the 5 major clades (line 87), it would be good to briefly list them, as Fig 1. shows 6 clades and it only becomes clear later that clade M is not stable.

Fig 1 and Fig A2: I am not quite sure what in Figure 1 is expanded compared to Fig A2, as per Figure 1 legend. They look similar to me. However, it is noticeable that Fig A2 only shows two haplotypes in Alberta, while Fig 1 shows three,

It would be good to indicate in Figure 1 which samples were radiocarbon dated or had a stratigraphic date. I can somewhat guess by their names, but it would be easier to have - for example - an asterisk by the respective name if there is a date for that sample.

Michael Knapp

Reviewer #2 (Remarks to the Author):

As I am not a geneticist, this review will not focus on the technical aspects of the DNA extraction and

genomic analysis. As a researcher primarily focused on Pleistocene megafauna and animal communities, my review comes from the perspective of a consumer of the genetic literature.

This work is of substantial value as the first large scale mitochondrial study of extinct North American mastodons, with analysis of 33 new mitogenomes. It addresses an existing model of Pleistocene mastodon distributions in North America: that populations repeatedly expanded northward into high latitudes regional during interglacial periods and were subsequently extirpated in these regions with the return to glacial conditions.

The identification of six previously unknown lineages of mastodons, including two distinct clades in eastern Beringia, is the most significant contribution of this paper. These data add to our growing knowledge of Pleistocene megafaunal demographics and responses to climate. As the authors correctly point out, the only phylogeographic studies of Pleistocene megafauna prior to this study were from bison, mammoth, horse, reindeer, and muskox—species adapted to either grassland or steppe-tundra environments. This study is one of the first to address megafaunal species adapted to forest or woodland habitats. As such there is a broad audience (including geneticists, paleontologists, ecologists, and archaeologists) that would be interested in these results. However, difficulties with precisely dating many of the samples from northern regions (either directly, using techniques such as radiocarbon or luminescence, or with their own attempts at molecular dating) limit definitive conclusions about mastodon responses to cyclical glacial-interglacial climate intervals. This manuscript has much to contribute to the discipline but requires some relatively minor reworking before publication.

It is fascinating and somewhat counter-intuitive that mastodons from northern latitudes (even in restricted geographic areas such as eastern Beringia and Alberta) contained specimens from distantly related clades. Genetic studies such as this are so valuable in part because they can identify complex ecological and evolutionary histories. That said, I would like a deeper discussion of these results in the methods and conclusion sections. How do these results—especially the ancient timing of splits in some of these clades, well before onset of glacial conditions –fit our larger understanding of mastodon origins and evolutionary history? They also diverge from my limited knowledge of the phylogenies of North American woolly mammoths and bison, which are arguably the best studied Pleistocene megafauna. On page 8, for example, the authors mention that prior work on bison (Shapiro et al, Heintzman et al) show a north-south separation of clades related to cyclical climate change. However, the changes in bison occurred within the last 100,000 years and are closely tied to clear MIS stages. The data presented here suggest much critical differentiation occurred prior to the beginning of the Pleistocene. It would be very useful to talk more about this larger patterning. For example, clade M contains a late surviving mastodon (30 kya) in Mexico with a lineage split from other north America mastodons over 6 mya ago. I would also like to hear more about clade G. Frankly, these are the samples with the best temporal control and probably represent a southern population which presumably would not undergo periodic extirpation. Because I would assume more genetic continuity in southern populations, it would be interesting to know more about this clade.

The section on mastodon age estimates is the most problematic, not because the median age estimates are unbelievable, but because the level of imprecision represented in the 95% HPD is very broad. For example, Clade Y's median age estimates of ~150,000 years have 95% HPD values ranging between 50,000-420,000 yrs. As shown in Figure 3b, even under a stricter clock model the best range is ~50,000-226,000 yrs. This span covers two complete glacial-interglacial cycles! Results from the Clade A analysis are even worse (95% HPD 50,000 – 685,000). Limited available data and the dating of individual specimens (rather than tree nodes) understandably increase imprecision. Given that level of uncertainty, however, I find it hard to accept some associations of these clades with specific MIS stages. The study results appear to be over-interpreted. One way to increase reader confidence would be to present the result of this analysis on clade G. These specimens are independently dated with radiocarbon dating, so it would be very interested to see how the age estimates from the molecular analysis compare to the independent dates.

Minor issues
• Not sure Table 1 contributes much. Lisiecki and Raymo's MIS stage descriptions are broadly available. As noted earlier, the temporal precision of the current analysis is not great and so such fine-scale focus on MIS stages may be problematic.
• Check the reference column in supplementary Table b. There appear to be some missing or incomplete references.

Matthew Hill
University of Iowa

Reviewer #3 (Remarks to the Author):

The paper by Karpinski et al. greatly expands the genetic dataset currently available for the American mastodon. Using robust phylogenetic and molecular dating methodology, the authors find multiple occupations of Eastern Beringia by this species, suggesting that the mastodon's evolutionary history was defined by a dynamics of range expansions and contractions.
I find the paper methodologically sound and clearly written. The conclusions are of interest to the community of researchers studying ancient population dynamics and phylogeography. I appreciate that the authors performed analyses to ensure quality of the generated data and that they do not overinterpret data. On the other hand, I am missing the link between the rather matter-of-factly description of results and their potential to address the general issues laid out in the beginning and at the end of the paper. I think that the paper would be improved by narrowing its scope and by a more careful discussion on the meaning and impact of the results.

Major comments:

I think that the generalization of the Abstract and Introduction is not matched by the interpretations and extrapolations in the Discussion and Conclusions. Although this study certainly improves our understanding of the Pleistocene megafauna range dynamics, I doubt that the spatial and temporal detail of the study is sufficient for it to markedly contribute to addressing issues like "how global climate change may affect the ecology and distribution of extant species" as indicated in the Abstract. I would suggest that the authors narrow down the focus of the paper, so that the areas in which it contributes to our understanding of population dynamics are clearer.

Specific comments:

Line 9: "large-scale phylogeographic study" - In light of previous publications on Proboscideans like Enk et al. 2016 and Chang et al. 2017, which contained 67 and 54 new mitogenomes, respectively, the term 'large-scale' becomes questionable and I think that it would be better to use something more relevant like 'continent-wide'.

Lines 17-19: Can the authors explain how? I think that they should either include more on this in the conclusions or the abstract should be phrased in a less generalized way.

Line 43: I would rephrase the sentence to "most Pleistocene phylogeographic studies on North America have focused on megafauna adapted to…" in order to not neglect the numerous studies focused e.g. on phylogenetics of Eurasian megafauna.

Lines 48-50: I'm not quite convinced about this statement. Doesn't climate-change pressure affect both warm-adapted and cold-adapted species in the same extant, just in different or opposite ways?

Line 78: Even though it comes later in the methods, it would be good to mention here at least a very

brief information about the samples, for example, where they come from (field-collected or museum specimen).

Line 87: Also, the listing of phylogenetic results comes abruptly. I know the methods are thoroughly discussed later, but it would be helpful to at least mention that a number of different extraction methods, library build methods, and phylogenetic inference methods were used. Especially, understanding what phylogenetic methods were used is important since these are directly compared in the main text and in the figure.

Line 112-122: I find the diverse genetic background of the Alberta samples, especially compared to the other samples, one of the most interesting results of the study. And although it is appropriate that the authors don't overinterpret these results because of the small sample size and uncertainty of the sample ages, I think it would be interesting to see more information on previously published work discussing the role of this region and whether anything is known about mastodon refugia in North America in general. I would like to know when was the Ice-Free corridor open and maybe also a bit more detail about the bison study by Heintzman et al. 2016 paper mentioned in the text, because there might be parallels to this study. Also, including a reference to Fig. 2 in this paragraph would be useful, I only noticed that it relates to it later.

Lines 146-177: I think that the section on age estimates and Fig. 3 could use a more thorough discussion. The main message of the paper is basically reduced to one sentence (lines 162 to 164).

Lines 178-184: I don't have a strong opinion about this, but the skyline plot could be moved to the supplementary since it doesn't deliver any important information.

Line 205: "represents a 16.5-fold increase in available molecular data for this species" - quantifying the contribution of this dataset seems redundant in the time period of big data being generated at an increasing speed.

Lines 251-259: At first reading, it wasn't clear to me what the authors meant and I think it would help to describe more clearly that a wide spectrum of extraction and library-build protocols was used and also to explain the reasoning.

Table 1:
I think this could go to the supplementary material since the glacial and interglacial cycles are already shown in Fig. 2A.

Fig. 1:
Would it be possible to use other colour than two shades of blue? My printer turned the dark blue on the tree, but not in the legend, into purple and it created confusion.
The clade Y star is not immediately visible if one doesn't look at the supplementary.
"Circles indicate locations" instead of "Large circles…." – As far as I can see, there is only one size of circles.

Fig. 2:
I find the depiction in Fig. 2F funny, but also more appropriate for a blog rather than a scientific publication. Maybe the authors could instead add another detail showing the progressing glacial conditions and absence of mastodons in eastern Beringia.

Fig. 3:
I think for consistency, the timeline of Fig. 2 and Fig. 3 should have the same direction.
I didn't quite understand which samples are shown. Why do the A and B figures list different samples in the legend?
For visualization purposes, the colours could correspond to the colours of clades in Fig. 2, with the text

boxes indicating clades highlighted in the same colour. The individual-colour figure could be then moved to supplementary.

Supplementary:
I think it would be very useful to include the reasoning behind using such a wide variety of laboratory methods and how these were piped into a protocol. It’s not immediately obvious if the extracts were pooled or which library build method was used on which extract.

Detailed Changelog:
Reviewer #1 (Remarks to the Author):
>Karpinski et al present an analysis of Mastodon phylogeography through time based on 33 new, complete mitogenomes.
>All experiments and analyses are conducted thoroughly, and I have little doubts about the data itself. Below are a few points that I feel could be improved, though.
>Major points:
>1. Most conclusions presented in this manuscript rely heavily on the ages estimated for the undated samples using molecular dating. The estimates produce broad 95% HPD intervals, which is not surprising, but which makes their interpretation somewhat speculative. The authors make a case for considering the reasonably tight peaks of the HPD distribution rather than the width of the complete 95% HPD intervals, and while this is a bit arbitrary, I feel it is a fair point to make. However, when so much relies on the molecular estimates, I would like to see how well this molecular age estimation actually performs with this specific dataset. This can for example be tested through "leave-one-out" cross validation of dated samples. I.e, take the dataset as is, but in each iteration, don't just estimate the age of the undated samples, but also the age of one sample for which we know the age. Repeat for all dated samples, and check how often the radiocarbon age of these samples
a) falls within the 95% HPD estimate for the respective sample, and
b) within the arbitrarily defined "peak" of the HPD distribution, and
c) how often the radiocarbon age is within the same MIS as the molecular estimate.
From my experience, this normally works reasonably well, and it really helps to lend some confidence to the results.
We recognize the concerns of the reviewer and have substantially reanalyzed the data using a variety of methods. The results of these additional analyses (Supplementary Methods) confirm that our date estimates have non-overlapping 95% HPD intervals that support the main thesis of our paper. We have also conducted a Leave-One-Out (LOO) analysis. The key results are:
- In 50 of the 56 (89%) LOO analyses, the 95% HPD intervals of the sample-age estimates contain the radiocarbon/ESR/stratigraphic dates.
- In 47 of 56 analyses (84%) the mode was closer than the median to the mean of the known age.
- The posterior distributions on specimen ages remain very broad.
- We identified an issue with the model when dealing with only dated specimens, where the clock rate estimate abutted the upper bound of the uniform prior that we specified. The model testing and age estimations have been revised with the assistance of Simon Ho, who has been added as a co-author.

>2. I find myself slightly skeptical as to how much can be said about the age distribution of clade A from two samples. The results are consistent with an earlier migration of clade A to Beringia compared to clade Y but with so few samples, this interpretation is standing on very shaky legs and is perhaps given a bit too much weight in the discussion and conclusion. And at least with relaxed clock settings, there is a significant overlap between the age of clade A and clade Y samples. Perhaps the above cross validation test will provide further support, though.
We agree that there are limitations to some aspects of the data; however, we have now redone our model selection using Bayes factors and found support for a strict clock over a relaxed clock. This suggests that our initial model was overparameterized. In our carefully constructed, new analyses using

a strict clock, the distributions of Clade A and Clade Y produce non-overlapping 95% HPD intervals.

> Minor points:
>Page 6.: When mentioning the 5 major clades (line 87), it would be good to briefly list them, as Fig 1. shows 6 clades and it only becomes clear later that clade M is not stable.

This is a good point and we have incorporated it into the text, although we note that Clade M is included in the 5 major clades and its monophyly is recovered in both midpoint-rooted maximum-likelihood and Bayesian phylogenies (see following paragraph). The sixth clade (Clade N) is not a true clade as it contains only a single specimen. However, this specimen is both geographically and temporally distinct from other clades and is likely to be the sole representative of a distinct group of mastodons from the east coast, for which we have not found any close relatives.

>Fig 1 and Fig A2: I am not quite sure what in Figure 1 is expanded compared to Fig A2, as per Figure 1 legend. They look similar to me. However, it is noticeable that Fig A2 only shows two haplotypes in Alberta, while Fig 1 shows three,

We have changed the word choice from “expanded” to “jittered”. Given the close geographic proximity of the Alberta specimens, we have artificially adjusted their latitude and longitude to clearly show the three clades present in Alberta. Only two clades are apparent in Figure A2, but this is due to the overlapping sampling locations of these specimens.

>It would be good to indicate in Figure 1 which samples were radiocarbon dated or had a stratigraphic date. I can somewhat guess by their names, but it would be easier to have - for example - an asterisk by the respective name if there is a date for that sample.

We have now placed an asterisk next to the dated specimens in the tree.

>Michael Knapp

Reviewer #2 (Remarks to the Author):

>As I am not a geneticist, this review will not focus on the technical aspects of the DNA extraction and genomic analysis. As a researcher primarily focused on Pleistocene megafauna and animal communities, my review comes from the perspective of a consumer of the genetic literature.

>This work is of substantial value as the first large scale mitochondrial study of extinct North American mastodons, with analysis of 33 new mitogenomes. It addresses an existing model of Pleistocene mastodon distributions in North America: that populations repeatedly expanded northward into high latitudes regional during interglacial periods and were subsequently extirpated in these regions with the return to glacial conditions.

>The identification of six previously unknown lineages of mastodons, including two distinct clades in eastern Beringia, is the most significant contribution of this paper. These data add to our growing knowledge of Pleistocene megafaunal demographics and responses to climate. As the authors correctly point out, the only phylogeographic studies of Pleistocene megafauna prior to this study were from bison, mammoth, horse, reindeer, and muskox—species adapted to either grassland or steppe-tundra environments. This study is one of the first to address megafaunal species adapted to forest or woodland habitats. As such there is a broad audience (including geneticists, paleontologists, ecologists, and archaeologists) that would be interested in these results. However, difficulties with precisely dating

many of the samples from northern regions (either directly, using techniques such as radiocarbon or luminescence, or with their own attempts at molecular dating) limit definitive conclusions about mastodon responses to cyclical glacial-interglacial climate intervals. This manuscript has much to contribute to the discipline but requires some relatively minor reworking before publication.
>It is fascinating and somewhat counter-intuitive that mastodons from northern latitudes (even in restricted geographic areas such as eastern Beringia and Alberta) contained specimens from distantly related clades. Genetic studies such as this are so valuable in part because they can identify complex ecological and evolutionary histories. That said, I would like a deeper discussion of these results in the methods and conclusion sections. How do these results—especially the ancient timing of splits in some of these clades, well before onset of glacial conditions –fit our larger understanding of mastodon origins and evolutionary history? They also diverge from my limited knowledge of the phylogenies of North American woolly mammoths and bison, which are arguably the best studied Pleistocene megafauna. On page 8, for example, the authors mention that prior work on bison (Shapiro et al, Heintzman et al) show a north-south separation of clades related to cyclical climate change.
>However, the changes in bison occurred within the last 100,000 years and are closely tied to clear MIS stages. The data presented here suggest much critical differentiation occurred prior to the beginning of the Pleistocene. It would be very useful to talk more about this larger patterning. For example, clade M contains a late surviving mastodon (30 kya) in Mexico with a lineage split from other north America mastodons over 6 mya ago.
We have modified the section on bison to clarify our meaning and apologize for perhaps misleading the reviewer. This section was not intended to as evidence that glacial/interglacial cycles are responsible for the structure of the phylogeny, but that these events were likely to have been very important for the biogeography of megafauna in North America. As mentioned in the paper, present-day Alberta (and surrounding areas) are very interesting in this respect, and in particular when examining cross-continental movement of populations in response to deglaciation, as it sits at the conflux of the Laurentide and Cordilleran ice sheets and would have been the first area to have been exposed as glaciers began to retreat. Both papers seek to characterize the recolonization of this area and the relative contributions of northern and southern populations.

With regards to identifying causes behind the deep divergences within our phylogeny, we have been cautious to avoid overinterpreting our date estimates, given the breadth of the 95% HPD intervals. It is worth noting that proboscideans, in general, display fairly deep divergences in mitochondrial phylogenies due to their matrilocal herd structure. Also unlike bison, mastodons (Mammutidae) have occupied North America for at least 18 million years, with American mastodons appearing ca. ~3.75 million years ago. Together this might provide some explanation for the long and very divergent lineages we have begun to capture.

>I would also like to hear more about clade G. Frankly, these are the samples with the best temporal control and probably represent a southern population which presumably would not undergo periodic extirpation. Because I would assume more genetic continuity in southern populations, it would be interesting to know more about this clade.
We now expand on this in the Results and Discussion in two sections: (1) addressing the ages of the two specimens without direct dates from this clade, and (2) expanding the section on their high diversity

(relative to northern clades). In line with the reviewer's expectations, we agree this clade is likely to have had greater continuity than northern clades, and think this probably explains the much higher diversity observed in this clade than in Clades Y and A.

>The section on mastodon age estimates is the most problematic, not because the median age estimates are unbelievable, but because the level of imprecision represented in the 95% HPD is very broad. For example, Clade Y's median age estimates of ~150,000 years have 95% HPD values ranging between 50,000-420,000 yrs. As shown in Figure 3b, even under a stricter clock model the best range is ~50,000-226,000 yrs. This span covers two complete glacial-interglacial cycles! Results from the Clade A analysis are even worse (95% HPD 50,000 – 685,000). Limited available data and the dating of individual specimens (rather than tree nodes) understandably increase imprecision. Given that level of uncertainty, however, I find it hard to accept some associations of these clades with specific MIS stages. The study results appear to be over-interpreted. One way to increase reader confidence would be to present the result of this analysis on clade G. These specimens are independently dated with radiocarbon dating, so it would be very interested to see how the age estimates from the molecular analysis compare to the independent dates.
We have now redone extensive analyses on specimens in clade G (see Supplementary Methods – Leave-One-Out Analysis, as well as our response to Reviewer #1), using our previous BEAST models, and completely redone model selection and dating to improve the reliability of our the results. We have also modified the Results and Discussion to clarify our interpretation and to shift the focus on the heterochronous nature of the Clades A and Y as opposed to the exact MIS stage of Clade A mastodons.

We would like to emphasize that estimates of sample ages made using a molecular clock are usually less precise than those based on direct methods of dating. However, molecular dating of samples remains a useful tool when other methods are unavailable.

>Minor issues
>Not sure Table 1 contributes much. Lisiecki and Raymo's MIS stage descriptions are broadly available. As noted earlier, the temporal precision of the current analysis is not great and so such fine-scale focus on MIS stages may be problematic.
We have removed Table 1.

>Check the reference column in supplementary Table b. There appear to be some missing or incomplete references.
We have added some references that were mistakenly omitted. However, references for specimens were provided when the information was known and if the specimen has been formally described in the literature. We have added a sentence stating this in the table caption.

>Matthew Hill
>University of Iowa

Reviewer #3 (Remarks to the Author):
>The paper by Karpinski et al. greatly expands the genetic dataset currently available for the American mastodon. Using robust phylogenetic and molecular dating methodology, the authors find multiple

occupations of Eastern Beringia by this species, suggesting that the mastodon's evolutionary history was defined by a dynamics of range expansions and contractions.

>I find the paper methodologically sound and clearly written. The conclusions are of interest to the community of researchers studying ancient population dynamics and phylogeography. I appreciate that the authors performed analyses to ensure quality of the generated data and that they do not overinterpret data. On the other hand, I am missing the link between the rather matter-of-factly description of results and their potential to address the general issues laid out in the beginning and at the end of the paper. I think that the paper would be improved by narrowing its scope and by a more careful discussion on the meaning and impact of the results.

>Major comments:

>I think that the generalization of the Abstract and Introduction is not matched by the interpretations and extrapolations in the Discussion and Conclusions. Although this study certainly improves our understanding of the Pleistocene megafauna range dynamics, I doubt that the spatial and temporal detail of the study is sufficient for it to markedly contribute to addressing issues like "how global climate change may affect the ecology and distribution of extant species" as indicated in the Abstract. I would suggest that the authors narrow down the focus of the paper, so that the areas in which it contributes to our understanding of population dynamics are clearer.

We have clarified our wording here in an attempt to better articulate the main goals. The argument we put forward is that the study of past species' responses can provide important information for predicting and testing how species might respond in the future, because these events have happened in the past multiple times. We also changed the wording to better tie in with the Conclusions and explain that these results offer important considerations and resources for identifying parallels in currently expanding species, in which case they might need to be monitored for conservation purposes.

>Specific comments:

>Line 9: "large-scale phylogeographic study" - In light of previous publications on Proboscideans like Enk et al. 2016 and Chang et al. 2017, which contained 67 and 54 new mitogenomes, respectively, the term 'large-scale' becomes questionable and I think that it would be better to use something more relevant like 'continent-wide'.

We have revised the abstract and this no longer appears.

>Lines 17-19: Can the authors explain how? I think that they should either include more on this in the conclusions or the abstract should be phrased in a less generalized way.

This point has been addressed above. In short, there are numerous comparisons that could be carried out between the population dynamics of past and present species (or even past expansions in long-term resident extant species), and any observed parallels would highlight the importance of also considering the diversity of the expanding subpopulations.

>Line 43: I would rephrase the sentence to "most Pleistocene phylogeographic studies on North America have focused on megafauna adapted to…" in order to not neglect the numerous studies focused e.g. on phylogenetics of Eurasian megafauna.

We have revised this accordingly.

>Lines 48-50: I'm not quite convinced about this statement. Doesn't climate-change pressure affect both

warm-adapted and cold-adapted species in the same extant, just in different or opposite ways?
We agree with the reviewer that the answer is more nuanced and complex than we originally conveyed. Accordingly, we have rephrased our sentence for clarity and have added a citation that shows ecosystem reconstructions throughout the Pleistocene. Our key takeaway here is that forest (and consequently forest-adapted species) occupied much of the area that became inaccessible due to advancing ice-sheets during glacials, and disappears completely in far northern environments even without glaciation. In comparison, steppe tundra and similar environments persist through both glacial and interglacial periods, although they decline in response to interglacial warming.

>Line 78: Even though it comes later in the methods, it would be good to mention here at least a very brief information about the samples, for example, where they come from (field-collected or museum specimen).
We have added a sentence here to explain that these were acquired from institutions and are not field samples.

>Line 87: Also, the listing of phylogenetic results comes abruptly. I know the methods are thoroughly discussed later, but it would be helpful to at least mention that a number of different extraction methods, library build methods, and phylogenetic inference methods were used. Especially, understanding what phylogenetic methods were used is important since these are directly compared in the main text and in the figure.
We have added a short mention of the phylogenetic methods in this section. We have also added a sentence at the top of the Methods sections (in DNA Extraction, Library Preparation, and Enrichment) to explain that a variety of techniques were used as they were developed and modified during the lifetime of the project.

>Line 112-122: I find the diverse genetic background of the Alberta samples, especially compared to the other samples, one of the most interesting results of the study. And although it is appropriate that the authors don't overinterpret these results because of the small sample size and uncertainty of the sample ages, I think it would be interesting to see more information on previously published work discussing the role of this region and whether anything is known about mastodon refugia in North America in general. I would like to know when was the Ice-Free corridor open and maybe also a bit more detail about the bison study by Heintzman et al. 2016 paper mentioned in the text, because there might be parallels to this study. Also, including a reference to Fig. 2 in this paragraph would be useful, I only noticed that it relates to it later.
We have added a reference to Figure 2 and have rephrased the section on bison (see also our response to reviewer #2).

We do not see the precise timing of the ice-free corridor as being of particular relevance to our study. The corridor opens ~13 kya and the study by Heintzman et al. (2016) specifically aimed to characterize the early colonization of this corridor, as a proxy for productive biological activity. As it relates to our dataset, we have no mastodon specimens that date to this time period from this region, and all dated mastodons in Alberta have so far returned non-finite dates (see Jass and Barron-Ortiz 2016 – A review of Quaternary proboscideans from Alberta, Canada).

Reconstructions of past glacial cycles and the exact closing and opening of the corridor are difficult to address, because most research has focused on the last glacial period. However, given that corridor moves from full coalescence to open in ~10 ky, individual fluctuations are not something that can be reasonably addressed given the uncertainty in the date estimates. It is also possible that the corridor functioned as a barrier to movement long before the Laurentide and Cordilleran ice sheets coalesced. We have added a citation in Line 49 when mentioning the changing biomes, and address this point in our proposed model in Fig. 3D and 3F. Even when the corridor opens at the end of the last glaciation, this region is primarily composed of various tundra environments (see Dyke 2005 - Late Quaternary Vegetation History of Northern North America Based on Pollen, Macrofossil, and Faunal Remains) which may explain the lack of finite-dated mastodons in this region. If the reverse process had also occurred (i.e., before closing the corridor was a long stretch of flat open tundra), it might have also presented an effective barrier to American mastodons. With that said, Dyke 2005 identified some forest biomes as already beginning to appear in the corridor within ~2 ky, showing the rapidly changing nature of this landscape.

>Lines 146-177: I think that the section on age estimates and Fig. 3 could use a more thorough discussion. The main message of the paper is basically reduced to one sentence (lines 162 to 164).
We have revised this section to provide more detail and in light of new dating methodology.

>Lines 178-184: I don't have a strong opinion about this, but the skyline plot could be moved to the supplementary since it doesn't deliver any important information.
We agree and have removed it, especially given that Bayes factors supported the constant-size model over the skyline plot.

>Line 205: "represents a 16.5-fold increase in available molecular data for this species" - quantifying the contribution of this dataset seems redundant in the time period of big data being generated at an increasing speed.
Agreed, this has been removed.

>Lines 251-259: At first reading, it wasn't clear to me what the authors meant and I think it would help to describe more clearly that a wide spectrum of extraction and library-build protocols was used and also to explain the reasoning.
We have cleaned up this section and added a sentence to clarify why so many methods were used here.

>Table 1:
I think this could go to the supplementary material since the glacial and interglacial cycles are already shown in Fig. 2A.
We have removed this entirely from the paper, following this comment and Reviewer #2's suggestion.

>Fig. 1:
Would it be possible to use other colour than two shades of blue? My printer turned the dark blue on the tree, but not in the legend, into purple and it created confusion.

We apologize for this and thank the reviewer for pointing it out. We have revised the colour scheme and have also changed the structure of the figure, to improve clarity.

>The clade Y star is not immediately visible if one doesn't look at the supplementary.
We have revised the caption so that it calls attention to the two stars.

>"Circles indicate locations" instead of "Large circles…." – As far as I can see, there is only one size of circles.
We apologize for this error, which was a relic from an older version of this figure.

>Fig. 2:
I find the depiction in Fig. 2F funny, but also more appropriate for a blog rather than a scientific publication. Maybe the authors could instead add another detail showing the progressing glacial conditions and absence of mastodons in eastern Beringia.
We would prefer to keep the image as we feel that it provides a clear depiction of what is happening in the region at each time point. We did consider using other symbols, but felt they over-anthropomorphized the animals, were not as clear, or perhaps too culturally specific.

>Fig. 3:
I think for consistency, the timeline of Fig. 2 and Fig. 3 should have the same direction.
I didn't quite understand which samples are shown. Why do the A and B figures list different samples in the legend?
For visualization purposes, the colours could correspond to the colours of clades in Fig. 2, with the text boxes indicating clades highlighted in the same colour. The individual-colour figure could be then moved to supplementary.
The dating analyses in our study have significantly changed, so we have now modified both figures. With respect to the coloration of Fig. 3, we agree this is a good change and incorporated it into our new designs.

>Supplementary:
I think it would be very useful to include the reasoning behind using such a wide variety of laboratory methods and how these were piped into a protocol. It's not immediately obvious if the extracts were pooled or which library build method was used on which extract.
Specimens were processed using established techniques available at the time of processing and as they were deemed appropriate for dealing with problematic specimens. A statement to this effect has been added to the main text as well as to the Supplementary Methods.

Supplementary Table B provides a full list of how each specimen was processed, which library was generated from which extract, whether indexed libraries from single specimens were generated independently or from the same source library, which were enriched and how, and how many times each library was sequenced.

REVIEWERS' COMMENTS:

Reviewer #1 (Remarks to the Author):

My previous comments have been thoroughly addressed by the authors and I have no further suggestions.

Reviewer #2 (Remarks to the Author):

I was a reviewer for the earlier version of this manuscript. I thought the previous submission had substantial value as it represented the first large scale mitochondrial study of extinct North American mastodons and provide interesting results that expanded our knowledge of understanding of mastodon origins and evolutionary history. My criticisms of the earlier version focused on issues related to 1) nature of the discussion of these results, and 2) what I interepretated as the the over interpretations of the mastodon age estimates, which had high very broad 95% HPD.

This new submission is a substantial improvement on the original manuscript. This version does a much better job in their discussion of clades and their revised methodologies (Bayesian age estimates and LOO analysis) resulted in improved reliability in their results discussion. The authors have adequately responded to my concerns and comments, and I have no further reservations for the publication of this manuscript. I expect this manuscript will be of great interest to a broad audience of Nature Communication readers.

Matthew Hill

Reviewer #3 (Remarks to the Author):

The authors made significant improvements to the manuscript. I believe that the new molecular dating analyses strengthen the argument for multiple re-expansions to Beringia and thanks to the changes in the text, results are now delivered more clearly and their potential to contribute to understanding the general issues outlined in the Introduction is more straightforward. I don't have any further comments.

Response to Reviewers

>REVIEWERS' COMMENTS:

>Reviewer #1 (Remarks to the Author):
>My previous comments have been thoroughly addressed by the authors and I have no further suggestions.

>Reviewer #2 (Remarks to the Author):
>I was a reviewer for the earlier version of this manuscript. I thought the previous submission had substantial value as it represented the first large scale mitochondrial study of extinct North American mastodons and provide interesting results that expanded our knowledge of understanding of mastodon origins and evolutionary history. My criticisms of the earlier version focused on issues related to 1) nature of the discussion of these results, and 2) what I interepretated as the the over interpretations of the mastodon age estimates, which had high very broad 95% HPD.
>This new submission is a substantial improvement on the original manuscript. This version does a much better job in their discussion of clades and their revised methodologies (Bayesian age estimates and LOO analysis) resulted in improved reliability in their results discussion. The authors have adequately responded to my concerns and comments, and I have no further reservations for the publication of this manuscript. I expect this manuscript will be of great interest to a broad audience of Nature Communication readers.
>Matthew Hill

>Reviewer #3 (Remarks to the Author):
>The authors made significant improvements to the manuscript. I believe that the new molecular dating analyses strengthen the argument for multiple re-expansions to Beringia and thanks to the changes in the text, results are now delivered more clearly and their potential to contribute to understanding the general issues outlined in the Introduction is more straightforward. I don't have any further comments.

We would like to deeply thank the reviewers of our paper for their input and direction during the peer review process. Their suggestions highlighted some key areas of weakness and additional tests which we could use to bolster our findings, and we feel the paper has been made stronger through their input. Thank you once again.

Sincerely,

Emil Karpinski and Hendrik N. Poinar
